# Enantioselective Synthesis of 1,12‐Disubstituted [4]Helicenes

**DOI:** 10.1002/anie.201915870

**Published:** 2020-02-20

**Authors:** Thierry Hartung, Rafael Machleid, Martin Simon, Christopher Golz, Manuel Alcarazo

**Affiliations:** ^1^ Institut für Organische und Biomolekulare Chemie, Georg-August-Universität Göttingen Tammannstr 2 37077 Göttingen Germany

**Keywords:** [4]helicenes, asymmetric catalysis, Au catalysis, enantioselective synthesis, ligand design

## Abstract

A highly enantioselective synthesis of 1,12‐disubstituted [4]carbohelicenes is reported. The key step for the developed synthetic route is a Au‐catalyzed intramolecular alkyne hydroarylation, which is achieved with good to excellent regio‐ and enantioselectivity by employing TADDOL‐derived (TADDOL=α,α,α,α‐tetraaryl‐1,3‐dioxolane‐4,5‐dimethanol) α‐cationic phosphonites as ancillary ligands. Moreover, an appropriate design of the substrate makes the assembly of [4]helicenes of different substitution patterns possible, thus demonstrating the synthetic utility of the method. The absolute stereochemistry of the newly prepared structures was determined by X‐ray crystallography and characterization of their photophysical properties is also reported.

## Graphical abstract

**Minimalistic helicenes**: The use of a chiral gold catalyst bearing an α‐cationic TADDOL‐derived phosphonite as an ancillary ligand allows the enantioselective assembly of 1,12‐disubstituted [4]helicenes—one of the smallest helical polyarene architectures.

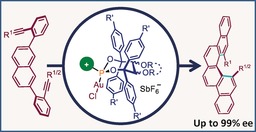

Carbohelicenes are screw‐shaped molecules formally derived from the *ortho*‐condensation of benzene rings.^1^ Even though no stereogenic centers are present in their structures, the twisted geometry imposed by their connectivity makes them chiral. The energetic barrier for the interconversion between the two enantiomeric forms is strongly dependent on the number of *ortho*‐fused benzene units. The first member of the carbohelicene family with a helicoidal structure is [4]helicene, which is configurationally unstable under ambient conditions.^2^ [5]helicene can be resolved, but the low activation energy of racemization (Δ*G* ^≠^=24.1 kcal mol^−1^) does not hamper the slow interconversion of the enantiomers. Hence, the racemization of these structures is typically complete after a couple of days at room temperature.^3^ The first configurationally stable member of the helicene family is [6]helicene. Racemization of [6]helicene only occurs after intensive heating (Δ*G* ^≠^=36.2 kcal mol^−1^; *t*_1/2_(*rac*)=48 min at 205 °C), making this scaffold the first carbohelicene intrinsically useful for the design of thermally stable chiral architectures.^4^

Nevertheless, the absolute configuration of low‐order [4] and [5]helicenes can be fixed either by installation of appropriate substituents at one or both termini of their fjord region, or by embedment of the helicene moiety into a more extended π‐conjugated scaffold.^5^ As an illustrative example, on the incorporation of a methyl substituent in the 1‐position of [5]helicene, the enantiomerization barrier increases up to Δ*G* ^≠^=39.1 kcal mol^−1^, making 1‐(methyl)[5]helicene even more reluctant to racemize than [6]helicene.^6^ The situation is analogous for [4]helicenes, but these structures require positions 1‐ and 12‐ to be simultaneously substituted to freeze the racemization process (Figure 1).^7^

**Figure 1:**
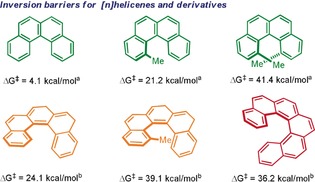
Inversion barriers for [4]‐, [5]‐, and [6]helicenes and their Me‐substituted derivatives; [a] Calculated values at the B3LYP/6‐31G(d) level of theory; [b] Experimental values.

Despite the impressive development already achieved in the synthesis of helicenes,^1^ and the number of applications that configurationally stable low‐order helicenes have found in diverse areas such as asymmetric catalysis,^8^ chiral recognition,^9^ or the design of molecular machines,^10^ highly enantioselective syntheses of [5]carbohelicene derivatives are scarce.^11^, ^12^ To the best of our knowledge, no enantioselective route is available for the preparation of 1,12‐disubstituted[4]helicenes.^13^

Being aware of the potential offered by Au‐catalyzed hydroarylation reactions for the assembly of conveniently designed alkynes into polyarenes,^14^ we conceived the enantiomeric synthesis of 1,12‐disubstituted [4]helicenes from substrates of general formulae **A** or **B** (Figure 2).

**Figure 2:**
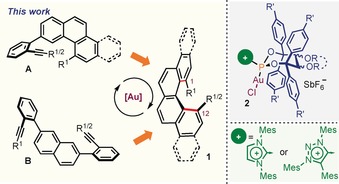
Synthetic approaches to the enantioselective synthesis of 1,12‐disubstituted [4]helicenes.

TADDOL‐derived α‐cationic phosphonites recently developed in our laboratory were chosen as ancillary ligands for the preparation of the necessary Au precatalysts **2** because of their high modularity, which allows easy tuning of the chiral pocket around the Au atom; and their cationic character, ultimately responsible for the enhanced activity of the actual catalytic species if compared with other Au catalysts of similar structure but derived from neutral ligands.^15^

We began our investigation by synthesizing dialkyne **3 a**, which was obtained by Suzuki coupling between known bistriflate **4** and boronic acid **5 a** (Scheme 1 a).^17^ Subsequently, a complete array of Au precatalysts **2 a**–**g** were screened for the successive double hydroarylation required to transform the model substrate into [4]helicene **1 a**. Our initial explorative conditions were set up as follows: catalyst loading of 10 mol %, fluorobenzene as solvent, and a working temperature of −20 °C. All reactions were allowed to proceed for 96 hours or until total consumption of the starting material.

**Scheme 1:**
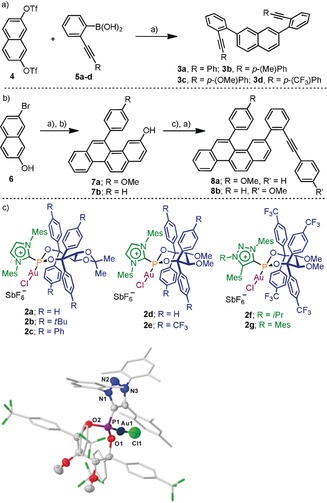
Synthesis of [4]helicene precursors and structures of the catalysts tested. Reagents and conditions: a) Pd_2_(dba)_3_ (5 mol %), SPhos (10 mol %), Cs_2_CO_3_ (4 equiv), THF/H_2_O (10:1), 80 °C, 24 h, **3 a**, 62 %; **3 b**, 67 %; **3 c**, 65 %; **3 d**, 89 %; b) **19** (9 mol %), AgSbF_6_ (9 mol %), DCM, **7 a**, 87 %; **7 b**, 42 %, both from **6** (two steps); c) Tf_2_O (1.5 equiv), pyridine (4.0 equiv), and then **5 a** or **5 c**, Pd_2_(dba)_3_ (5 mol %), SPhos (10 mol %), Cs_2_CO_3_ (2 equiv), THF/H_2_O (10:1), 80 °C, 24 h, **8 a**, 60 %; **8 b**, 25 % (two steps). X‐ray structure of **2 g**, H atoms, co‐crystallized solvents and SbF_6_ anions are removed for clarity. Arene moieties are drawn as reduced sticks, ellipsoids drawn at 50 % probability level.^16^

The performance of catalysts **2 a**–**c**, all containing an acetonide backbone, was modest in terms of regio‐ and enantioselectivity (Table 1, Entries 1–3). Interestingly, replacement of the acetonide motif by methoxy groups, such as in Au complexes **2 d**,**e**, improved the performance of the catalysts.^18^ Specifically, catalyst **2 e**, which contains four *p*‐(CF_3_)Ph substituents, was able to promote the desired double cyclization towards [4]helicene **1 a** with excellent enantio‐ (97 % *ee*) and regioselectivity (**1 a**:**9 a**; 98:2) (Table 1, Entry 5). The catalytic system suffers, however, from low reactivity, and reaction times of up to four days were necessary.

**Table 1:** Screening of chiral Au‐phosphonite complexes in the hydroarylation of mono‐ and diynes towards [4]helicenes.^[a]^ 
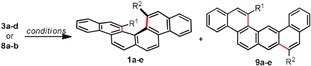

Entry	Au catalyst	Substrate	Yield [%]	**1:9**	**1** (*ee* [%])
1	**2 a**	**3 a**	50	75:25	**1 a** (−36)
2	**2 b**	**3 a**	13	50:50	**1 a** (−30)
3	**2 c**	**3 a**	91	94:6	**1 a** (−8)
4	**2 d**	**3 a**	52	68:32	**1 a** (28)
5	**2 e**	**3 a**	94	98:2	**1 a** (97)
6	**2 f**	**3 a**	97	99:1	**1 a** (98)
7^[b]^	**2 g**	**3 a**	98	98:2	**1 a** (99)
8^[b,c]^	**2 g**	**3 a**	95	96:4	**1 a** (98)
9^[b]^	**2 g**	**3 b**	94	97:3	**1 b** (97)
10^[b,d]^	**2 g**	**3 c**	84	93:7	**1 c** (89)
11^[b,c]^	**2 g**	**3 d**	4	>99:1	**1 d** (95)
12^[b,d]^	**2 g**	**8 a**	57	98:2	**1 e** (88)
13^[b,d]^	**2 g**	**8 b**	65	94:6	**1 e** (97)

[a] Reaction conditions: **3 a**–**d** (0.02 mmol), catalysts **2 a**–**g**, 10 mol %, AgSbF_6_ 10 mol %, FC_6_H_5_ (0.05 m), −20 °C, 96 h. Yields are of the isolated **1**:**9** mixtures; regioisomer ratios were determined by ^1^H NMR spectroscopy and *ee* values by chiral HPLC. [b] CH_2_Cl_2_ was used as solvent (0.05 m). [c] Reaction carried out at 0 °C. [d] Catalyst loading of 5 mol %.

In an attempt to solve this issue, the imidazolium moiety in ligand **2 e** was exchanged by more‐electron‐withdrawing 1,2,3‐triazolium units, and the already optimal chiral environment around the Au atom was maintained. Both resulting catalysts, **2 f** and **2 g**, were able to match, or even slightly overtake, the already outstanding regio‐ and enantioselectivities of **2 e** (Table 1, Entries 6 and 7), and by employing **2 g** the reaction times were shortened to only two days. No product derived from the 5‐*exo*‐dig cyclization of the substrate was observed in any of these experiments. A final solvent screening indicated that **2 g** does not require the employment of fluorobenzene to maintain excellent levels of enantioinduction; highly competitive results were also obtained working in dichloromethane (Table 1, Entry 8).15a Finally, crystals of precatalyst **2 g** were obtained, and its molecular connectivity was confirmed by X‐ray diffraction (see Scheme 1 c and the Supporting Information).

By using the conditions already optimized, the respective cyclization reactions of diynes **3 b**,**c** into **1 b**,**c** took place with high levels of regio‐ and enantioselectivity (Table 1, Entries 9 and 10). Interestingly, the more reactive nature of **3 c**, decorated with terminal *p*‐anisyl substituents allowed a reduction of the catalyst loading to only 5 mol % without significant erosion of the yield. On the other hand, the cyclization of diyne **3 d**, bearing strong electron‐withdrawing CF_3_ substituents proved to be difficult and only traces of the desired [4]helicene **1 d** was obtained under the standard reaction conditions, although with outstanding *ee* (4 % yield of isolated product, 95 % *ee*). The mono‐cyclized intermediate is the main species present in the mixture (Table 1, Entry 11).

The hydroarylation of substrates **8 a** and **8 b** towards non C_2_‐symmetrically substituted **1 e** proceeded in both cases with acceptable yields. The same major enantiomer was obtained from both reactions, albeit the enantioselectivity of the cyclization is slightly eroded when using **8 a** as the substrate (Table 1, Entries 12 and 13). Considering that only one hydroarylation event is required to obtain **1 e** from these substrates, it is not surprising that only 5 mol % of precatalyst **2 g** is required for the reactions to conclude in 48 h. The X‐ray structure of substrate **8 a** and precursor **7 a** are depicted in the Supporting Information.

Encouraged by these results, and seeking to further explore the viability of our cycloisomerization protocol towards other [4]helicene structures, additional alkyne precursors were evaluated. Thus, phenanthrene derivatives **18 a**–**j** were prepared following a multistep route, which is described in detail in Scheme 2.

**Scheme 2:**
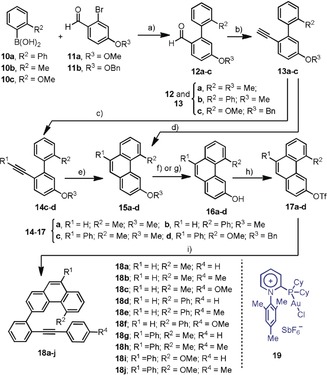
Synthesis of non‐symmetric [4]helicene precursors. Reagents and conditions: a) Pd(AcO)_2_ (3 mol %), SPhos, (3 mol %), K_3_PO_4_ (2.0 equiv), toluene/dioxane (10:1), **12 a**, 92 %, **12 b**, 91 %, **12 c**, 87 %; b) Ohira–Bestmann reagent (3.0 equiv), K_2_CO_3_ (3.0 equiv), MeOH, **13 a**, 95 %, **13 b**, 99 %, **13 c**, 87 %; c) PhI (1.0 equiv), CuI (3 mol %), PdCl_2_(PPh_3_)_2_ (2 mol %), Et_3_N (5 mL), **14 c**, 89 %, **14 d**, 71 %; d) PtCl_2_ (10 mol %), toluene, 85 °C, **15 a**, 60 %; **15 b**, 89 %; e) **19** (10 mol %), AgSbF_6_ (10 mol %), DCM, **15 c**, 73 %; **15 d**, not isolated; f) BBr_3_, DCM, **16 a**, 99 %; **16 b**, 85 %; **16 c**, 73 %; g) BCl_3_ (2 equiv), C_6_HMe_5_ (3 equiv),**16 d**, 67 % (two steps); h) Tf_2_O (1.2 equiv), Et_3_N, **17 a**, 79 %; **17 b**, 75 %; **17 c**, 98 %, **17 d**, 95 %; i) **5 a**–**c**, Pd_2_(dba)_3_ (5 mol %), SPhos (10 mol %), Cs_2_CO_3_ (2 equiv), THF/H_2_O (10:1), 80 °C, 24 h, **18 a**, 89 %; **18 b**, 92 %; **18 c**, 68 %; **18 d**, 51 %; **18 e**, 43 %; **18 f**, 38 %; **18 g**, 67 %; **18 h**, 75 %; **18 i**, 62 %; **18 j**, 81 %.

Key for the success of this synthetic plan was the effective preparation of phenanthrene intermediates **15 a**–**d** by intramolecular hydroarylation of the corresponding alkynes. Although for substrates **13 a**,**b**, containing a terminal alkyne, the cycloisomerization proceeded successfully in the presence of catalytic amounts of PtCl_2_,^19^ neither PtCl_2_ or Ph_3_PAuCl/AgSbF_6_ were able to satisfactorily promote the hydroarylation step for internal alkynes **14 c**,**d**. Only the employment of Au precatalyst **19**, containing a strong π‐acceptor *N*‐arylpyridinio phosphine as an ancillary ligand, induced the formation of **15 c**,**d** in synthetically practical yields.^20^

For substrates **18 a**–**c** (R^2^=Me), the performance of **2 g** (5 mol %) is mediocre in terms of regio‐ and enantioselectivity (Table 2, Entries 1–3), and significant amounts of undesired benzo[m]tetraphenes **21 a**–**c** were obtained as side products. Interestingly, complete control over the enantioselectivity (97–99 % *ee*) is achieved by formal exchange of the methyl groups at position R^2^ by phenyl groups (substrates **18 d**–**f**); however, the regioselectivity of the cyclization for these substrates is still far from ideal (Table 2, Entries 4–6). Note that **8 a**,**b** only differs from **18 d**–**f** in a remote benzannulation, but this seems to be crucial to effectively direct the hydroarylation to the inner position of the phenanthrene (Table 1, Entries 11–12).

**Table 2:** Scope of the Au‐catalyzed hydroarylation of **18 a**–**j** towards [4]helicenes.^[a]^ 
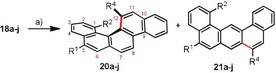

Entry	Substrate	Yield [%]	**20:21**	**20** (*ee* [%])
1	**18 a**	93	63:37	**20 a** (60)
2	**18 b**	43	42:58	**20 b** (75)
3	**18 c**	85	44:56	**20 c** (70)
4	**18 d**	93	69:31	**20 d** (97)
5	**18 e**	93	54:46	**20 e** (99)
6	**18 f**	75	38:62	**20 f** (98)
7	**18 g**	87	90:10	**20 g** (67)
8	**18 h**	93	98:2	**20 h** (79)
9	**18 i**	93	99:1	**20 i** (92)
10	**18 j**	94	95:5	**20 j** (90)

[a] Reaction conditions: **18 a**–**j** (0.02 mmol), catalyst **2 g**, 5 mol %, AgSbF_6_ 10 mol %, CH_2_Cl_2_ (0.05 m), −20 °C, 96 h. Yields are of the isolated **20**:**21** mixtures; regioisomer ratios were determined by ^1^H NMR spectroscopy and *ee* values by chiral HPLC.

Similarly, alkynes **18 g**,**h** only differs from **18 a**,**b** in a phenyl substituent from the outer rim of the phenanthrene; however, for these structures the cyclization is again selectively directed to the desired position affording **20 g**,**h** with high regioselectivity (Table 2, Entries 7–8). The best results of the series were obtained for substrates **18 i**,**j** (R^1^=Ph, R^2^=OMe), which were transformed into **20 i**,**j** with excellent regio‐ and enantioselectivities. Further scrutiny is ongoing to fully understand the effect of remote substitutions on the hydroarylation site, but it seems to be consistent that catalyst **2 g** promotes higher enantioselectivites for π‐extended structures.^15^

The connectivity of parent [4]helicene **1 a** was unambiguously confirmed by X‐ray crystallography employing a racemic single crystal (Figure 3). In this compound the vertical distance between the overlapping C1 and C7 carbon atoms is 3.243(1) Å, which is basically identical to the value in [6]helicene (3.215 Å). On the other hand, the torsion angles along the inner rim in **1 a** (from C1 to C7, *ϕ*=20.3, 27.6, 27.6, 20.3°) vary significantly if compared with those in [6]helicene (*ϕ*=11.2, 30.1, 31.0, 15.2°), in particular for rings A and D. This difference is likely to be caused by the higher tolerance to geometrical distortion of the one‐point‐connected phenyl groups.^21^ To examine the chiral stability of the [4]helicenes prepared, enantioenriched **1 a** (98 % *ee*) was heated at 180 °C in 1,2‐dichlorobenzene for 24 h and later monitored by chiral HPLC. No erosion of the *ee* value was observed, highlighting the configurational stability of 1,12‐disubstituted [4]helicenes.

**Figure 3:**
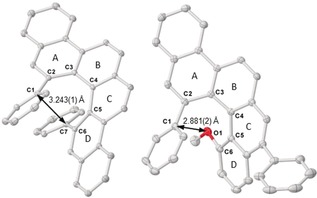
X‐ray structure of *rac*‐**1 a** (left) and **20 j** (right). H atoms are removed for clarity and ellipsoids drawn at 50 % probability level.^16^

The absolute configuration of the newly prepared helicenes was determined to be *P* from the X‐ray analyses of single crystals of **20 i** and **20 j** (See Figure 3 and the Supporting Information).^22^ For **20 i**, both the Flack and Hooft parameters (0.01(4) and 0.04(3), respectively) unambiguously support this assignment. For **20 j** an identical conclusion can be reached (0.01(12) and 0.09(5) for the Flack and Hooft parameters, respectively). The higher estimated standard deviation for the latter case is attributed to lower data redundancy for this measurement. Importantly, both independent results point to the same absolute configuration. Additionally, the circular dichroism spectra for [4]helicenes **1 a**–**e** are shown in Figure 4. Comparison of these EDC spectra with that reported by Yamaguchi and co‐workers for a 1,12‐dimethyl substituted [4]helicene further confirms the assignment of the helicity made by crystallographic techniques.^23^

**Figure 4:**
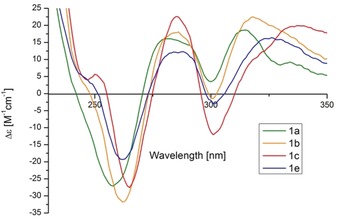
Circular dichroism spectra of **1 a**–**e** in dichloromethane.

The absorption and fluorescence spectra of the [4]helicenes synthesized can be found in the Supporting Information. Interestingly, compounds **1 a**–**e** display blue fluorescence, with the emission maximum being located at 438–440 nm. The emission bands for **20 a**–**j**, architectures characterized by only one benzannulation of the parent [4]helicene, are slightly blue‐shifted and appear at 426–433 nm.

In summary, we report herein the first highly enantioselective synthesis of 1,12‐disubstituted [4]carbohelicenes, achieved through the Au‐catalyzed intramolecular hydroarylation of appropriate alkynes and employing a TADDOL‐derived α‐cationic phosphinite as an ancillary ligand. Single‐crystal X‐ray analysis unambiguously determined the connectivity of the new structures obtained and established their absolute configuration. Ongoing work in our laboratory is focused on the further optimization of the catalytic system developed towards the enantioselective synthesis of higher‐order carbohelicenes and other polyhelical scaffolds.

## Conflict of interest

The authors declare no conflict of interest.

## Supporting information

As a service to our authors and readers, this journal provides supporting information supplied by the authors. Such materials are peer reviewed and may be re‐organized for online delivery, but are not copy‐edited or typeset. Technical support issues arising from supporting information (other than missing files) should be addressed to the authors.

Supplementary
