# Supplementary material for: Enantioselective Synthesis of 1,12‐Disubstituted [4]Helicenes
Source: Angew Chem Int Ed Engl. 2020 Feb 20;59(14):5660–4. doi: 10.1002/anie.201915870 (PMC7154633; doi:10.1002/anie.201915870)
Supplement: Supplementary file 1 — Supplementary [file ANIE-59-5660-s001.pdf]

Angewandte Chemie
Eine Zeitschrift der Gesellschaft Deutscher Chemiker

Supporting Information

Enantioselective Synthesis of 1,12-Disubstituted [4]Helicenes

*Thierry Hartung, Rafael Machleid, Martin Simon, Christopher Golz, and Manuel Alcarazo**

anie_201915870_sm_miscellaneous_information.pdf

Table of Contents

Experimental Procedures 3
General working methods 3
Starting materials 3
General analytical methods 3
Synthesis of Compounds **3a-d** 5
General Procedure A for the preparation of boronic acids **5a-d** 5
General Procedure B for Suzuki cross-couplings 5
Synthesis of the Compounds **8a** and **8b** 8
General procedure C for racemic cycloisomerisations 9
General procedure D for aromatic triflates 10
Synthesis of compounds **18a-j** 12
General procedure E for the Ohira Bestmann variation of the Seyferth-Gilbert Homologisation 14
General Procedure F for the Sonogashira couplings 15
General Procedure G for $PtCl_2$ catalysed cyclo-isomerisation 16
General Procedure H for the cleavage of methyl ethers 17
General Procedure I for the cleavage of benzyl ethers 18
Synthesis of tetrahelicenes 24
General Procedure J for the enantioselective cycloisomerisation 24
Photophysical properties of helicenes 32
Circular dichroism 32
Absorption and Fluorescence 36
NMR-Spectra 40
Single crystal X-ray diffraction analysis 101
HPLC-chromatograms of helicenes 110
References 128
Author contributions 128

Experimental Procedures

General working methods

Unless otherwise stated, all reactions were carried out in dried glassware under an inert atmosphere (nitrogen or argon if specifically noted) using standard Schlenk techniques, or under an atmosphere of nitrogen in a MBraun UNIlab plus glovebox. Dry and degassed solvents were obtained by distillation over the appropriate drying agents and stored under nitrogen. Alternatively, dry solvents were obtained using an MBraun MB-SPS-800 solvent purification system (tetrahydrofuran, diethyl ether, toluene, pentane, dichloromethane, acetonitrile). Flash chromatography was performed on Macherey Nagel 60 (40-63 μm) silica gel. Reactions were controlled by thin-layer chromatography (TLC) analysis, performed using polygram SIL G/UV254 TLC-plates from Macherey Nagel, and visualized by UV irradiation (λ = 254 nm).

Starting materials

Unless otherwise stated, all reagents were used as received from commercial suppliers (ABCR, Acros Organics, Alfa Aesar, Chempur GmbH, J and K Scientific, Sigma Aldrich, Thermo Fisher Scientific, Tokyo Chemical Industry). Compounds synthesized according to literature procedures: Naphthalene-2,7-diyl bis(trifluoromethanesulfonate) **4**[1], 7-bromonaphthalen-2-ol **6**[2], 1-bromo-2-(phenylethynyl)benzene[3], 1-bromo-2-(p-tolylethynyl)benzene[3], 1-bromo-2-((4-methoxyphenyl)ethynyl)benzene[3], 1-bromo-2-((4-(trifluoromethyl)phenyl)ethynyl)benzene[3], catalysts **2g**[4], **19**[5] and **19a**[5].

General analytical methods

NMR: spectra were recorded on Bruker AV600, AV500, AV400, or Varian Inova 500 spectrometers; ^{1}H and ^{13}C chemical shifts (δ) are given in ppm relative to TMS, using the solvent signals as references and converting the chemical shifts to the TMS scale. ^{31}P and ^{19}F chemical shifts (δ) are given in ppm relative to H_3PO_4 and $CFCl_3$ respectively (external standard). Coupling constants (J) are given in Hz. Solvents for NMR spectroscopy used as received from Eurisotop.
HRMS: Finnigan MAT 95 (70 eV, EI), Finnigan LCQ (ESI) and APEX IV 7T FTICR, Bruker Daltonic (HRMS).
IR: FT/IR-4100 (Jasco), wavenumbers ($\tilde{\nu}$) in cm^{-1}.
HPLC: the relative ratios of products in catalysis reactions were determined by analytical HPLC using either Shimadzu Nexera-*i* LC 2040 3D with integrated downstream UV/Vis PDA detector (detection at 254 nm, reverse phase) or a Shimadzu Prominence-I LC-2030C 3D Plus with integrated downstream UV/Vis PDA detector (detection at 254 nm, normal phase). Separation was carried out on Agilent ZORBAX SB-C18, 4.6x250 mm, 3.5µm column. Specific conditions such as eluent mixtures, flow rates and temperatures are stated for each case. System control and chromatogram analysis were carried out with LabSolutions (Shimadzu) software.
Chiral HPLC: the enantiomeric excesses of the products in catalysis reactions was determined using either a Shimadzu Nexera-*i* LC 2040 3D with integrated downstream UV/Vis PDA detector (detection at 254 nm, reverse phase) or a Shimadzu Prominence-*i* LC-2030C 3D Plus with integrated downstream UV/Vis PDA detector (detection at 254 nm, normal phase). Separation was carried out on Daicel Chiral Technologies IC-3, 4.6x150 mmL, 3µm column. Specific conditions eluent mixtures, flow rates and temperatures are stated for each compound. System control and chromatogram analysis were carried out with LabSolutions (Shimadzu) software.

Preparative HPLC: preparative separations were carried out on an Interchim PuriFlash 4250 using either an Agilent ZORBAX SB-C18, 21.2x250 mm, 7 µm column (up to 50 mg) or an Agilent ZORBAX 7 SB-C18, 50.0x250 mm, 7µm column (>50 mg).
SFC: Waters Acquity UPC² modular system, consisting of an Acquity UPC² Binary Solvent Manager, Acquity UPC² Sample Manager, Acquity UPLC Column Manager, Acquity UPC² PDA Detector and Acquity UPC² Convergence Manager. Detection was carried out using downstream PDA/UV-vis detector with pressure resistant SFC-suitable flow cell, sold with the UPC² system. Software used was Empower 3, Revision 5.
Specific rotations: were collected using a Jasco P-2000 polarimeters at the stated temperature under a Na/Hg lamp, λ = 589 nm (*c* in g/100 ml).

Synthesis of Compounds 3a-d

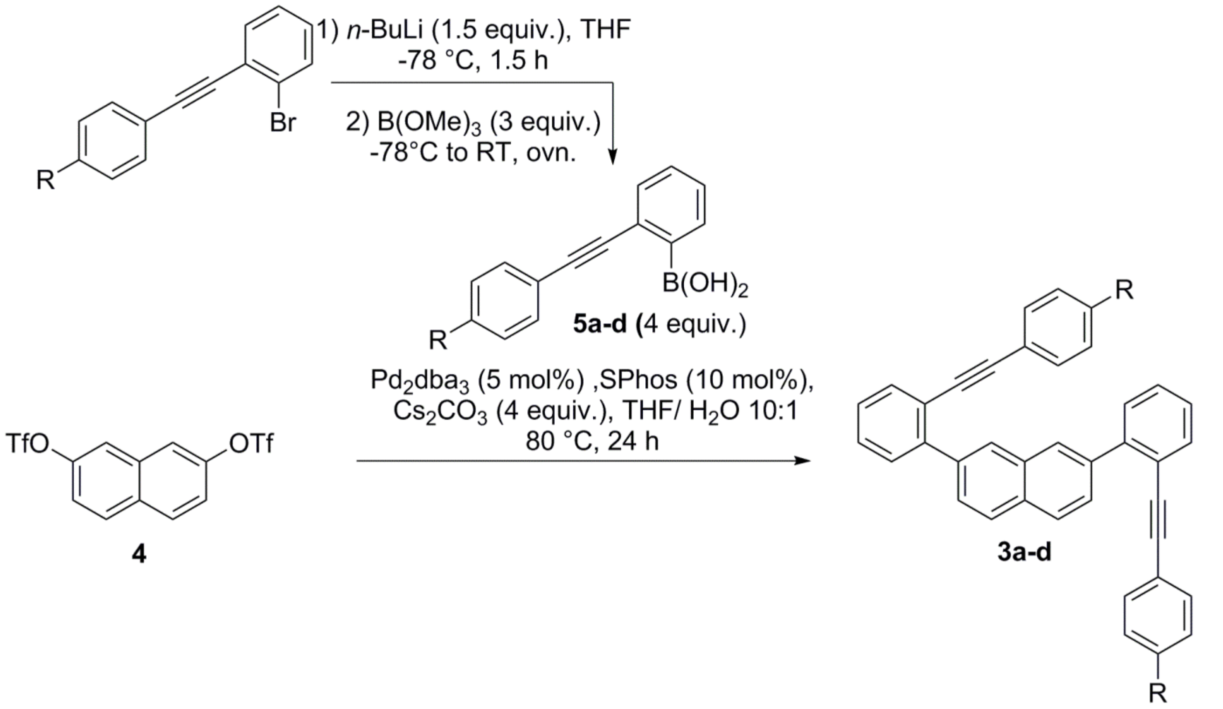

Scheme 1 Synthesis of symmetrical Tetrahelicene Precursors

General Procedure A for the preparation of boronic acids 5a-d

A dried Schlenk flask equipped with a magnetic stirring bar was charged with the respective arylbromide (1.0 equiv.), which was then dissolved in THF and cooled to -78 °C. *n*-Butyllithium (1.5 equiv.) was added dropwise over 10 min. The mixture was stirred at -78 °C for 1.5 h. Trimethylborate (3.0 equiv.) was added and the reaction mixture was allowed to reach room temperature overnight, while maintaining vigorous stirring. The reaction was quenched by the addition of aqueous HCl (2M). The phases were separated and the aqueous phase was extracted with ether (3 x 50 mL). The combined organic phases were dried over Na_2SO_4 and the solvents removed *in vacuo*. The obtained boronic acids **5a-d** were used without further purification.

General procedure B for Suzuki Cross-couplings

A Schlenk flask equipped with a magnetic stirring bar was loaded with the respective bromide or triflate (1 equiv. (per reactive moiety), the respective boronic acid **5a-d** (2 equiv.), the base (Cs_2CO_3 (1.5 equiv.) or K_3PO_4 (2 equiv.)), Pd_2dba_3 (5 mol%) and SPhos (10 mol%). A thoroughly degassed mixture of THF and H_2O (10:1, 0.04 M) was added. The solution was placed in a pre-heated oil bath and stirred at 80 °C for 24 h. After cooling to room temperature the mixture was eluted through a plug of silica gel using DCM. Column chromatography (SiO_2, EtOAc in pentane) yielded the desired alkynes **3a-d.**

Compound 3a

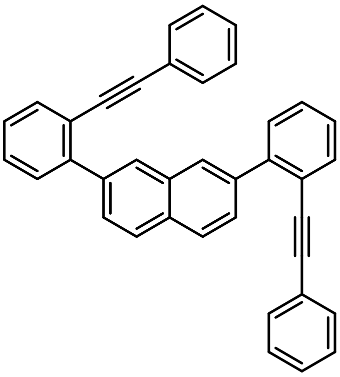

Prepared from **4** (701 mg, 1.65 mmol, 1.0 equiv), **5a** (1.47 g, 6.61 mmol, 4.0 equiv), Pd_2dba_3 (60.52 mg, 0.66 mmol, 5 mol%), SPhos (54.26 mg, 0.13 mmol, 10 mol%), Cs_2CO_3 (2.19 g, 4.96 mmol, 3.0 equiv.) and the specified solvent mixture (40 mL) according to general procedure **B**. Column chromatography (SiO_2, 0 to 10% EtOAc in pentane) yielded the product as a white solid (494 mg, 1.03 mmol, 62%).

^{1}H NMR: (400 MHz, $CDCl_3$) δ = 8.18 – 8.14 (m, 1H), 7.98 (d, *J* = 8.5 Hz, 1H), 7.91 (dd, *J* = 8.5, 1.7 Hz, 1H), 7.70 (dd, *J* = 7.6, 1.5 Hz, 1H), 7.55 (dd, *J* = 7.6, 1.5 Hz, 1H), 7.45 (td, *J* = 7.6, 1.5 Hz, 1H), 7.38 (td, *J* = 7.5, 1.4 Hz, 1H), 7.33 – 7.27 (m, 2H), 7.25-7.19 (m, 3H). **^{13}C{H} NMR:** (101 MHz, $CDCl_3$) δ = 143.8, 138.5, 133.2, 133.0, 132.0, 131.4, 129.8, 128.6, 128.5, 128.2, 128.1, 128.1, 127.2, 127.0, 123.4, 121.9, 92.6, 89.4. **IR:** (neat) ṽ = 3059, 3022, 2161, 1891, 1806, 1753, 1675, 1629, 1597, 1572, 1530, 1491, 1476, 1454, 1444, 1388, 1364, 1336, 1309, 1270, 1190, 1174, 1161, 1154, 1141, 1099, 1070, 1049, 1025, 996, 977, 968, 958, 947, 913, 880, 862, 839, 799, 770, 749, 716, 689, 666, 631, 617, 591, 566, 558, 536, 524, 506, 496, 484, 473, 448, 435, 419, 412, 405 cm^{-1}. **HRMS:** calcd m/z. for; $C_{38}H_{24}^+$ $[M+H]^+$: 481.1951 ; found (ESI) 481.1942.

Compound 3b

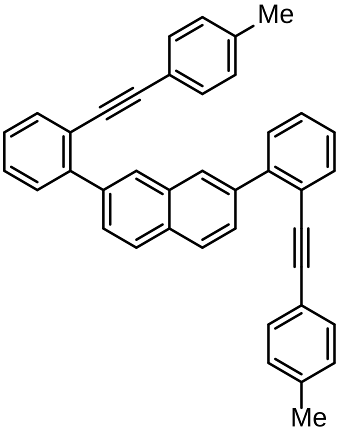

Prepared from **4** (100 mg, 0.24 mmol, 1.0 equiv), **5b** (223 g, 0.94 mmol, 4.0 equiv), Pd_2dba_3 (10.8 mg, 0.012 mmol, 5 mol%), SPhos (9.68 mg, 0.023 mmol, 10 mol%), K_3PO_4 (200 mg, 0.94 mmol, 4.0 equiv.) and the specified solvent mixture (15 mL) according to general procedure **B**. Column chromatography (SiO_2, 0 to 10% EtOAc in pentane) yielded the product as a yellow solid (81 mg, 0.16 mmol, 67%).

^{1}H NMR: (300 MHz, $CDCl_3$) δ = 8.16 (s, 2H), 7.98 (d, *J* = 8.5 Hz, 2H), 7.92 (dd, *J* = 8.5, 1.6 Hz, 2H), 7.70 (dd, *J* = 7.6, 1.4 Hz, 2H), 7.56 (dd, *J* = 7.7, 1.3 Hz, 2H), 7.44 (td, *J* = 7.5, 1.4 Hz, 2H), 7.38 (td, *J* = 7.5, 1.3 Hz, 2H), 7.21 (d, *J* = 7.9 Hz, 4H), 7.04 (d, *J* = 7.8 Hz, 4H), 2.30 (s, 6H). **^{13}C{H} NMR:** (101 MHz, $CDCl_3$) δ = 143.7, 138.5, 138.3, 133.2, 132.9, 131.9, 131.3, 129.8, 129.0, 128.5, 128.4, 128.0, 127.2, 126.9, 122.1, 120.3, 92.8, 88.8, 21.5. **IR:** (neat) ṽ = 3058, 3023, 2961, 2913, 2860, 2363, 2331, 1907, 1684, 1652, 1628, 1592, 1559, 1508, 1476, 1439, 1406, 1307, 1260, 1180, 1159, 1141, 1100, 1017, 945, 908, 874, 843, 813, 753, 716, 706, 692 ,653, 631, 619, 588, 562, 538, 524, 512, 504, 496, 484, 473, 457, 442, 430, 417, 408 cm^{-1}. **HRMS:** calcd m/z. for; $C_{40}H_{28}^+$ $[M+Na]^+$: 531.2081; found (ESI) 531.2070.

Compound 3c

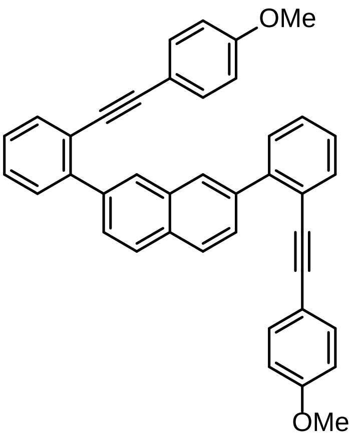

Prepared from **4** (800 mg, 1.99 mmol, 1.0 equiv), **5c** (2.02 g, 7.99 mmol, 4.0 equiv), Pd_2dba_3 (73.1 mg, 0.80 mmol, 5 mol%), SPhos (65.5 mg, 0.16 mmol, 10 mol%), Cs_2CO_3 (1.95 g, 4.96 mmol, 3.0 equiv.) and the specified solvent mixture (20 mL) according to general procedure **B**. Column chromatography (SiO_2, 0 to 10% EtOAc in pentane) yielded the product as a yellow solid (704 mg, 1.29 mmol, 65%).

^{1}H NMR: (300 MHz, $CDCl_3$) δ = 8.15 (s, 2H), 7.97 (d, *J* = 8.5 Hz, 2H), 7.89 (dd, *J* = 8.6, 1.6 Hz, 2H), 7.67 (dd, *J* = 7.5, 1.2 Hz, 2H), 7.54 (d, *J* = 7.5 Hz, 2H), 7.42 (td, *J* = 7.5, 1.6 Hz, 2H), 7.36 (td, *J* = 7.9, 1.7 Hz, 2H), 7.22 (d, *J* = 8.7 Hz, 4H), 6.74 (d, *J* = 8.7 Hz, 4H), 3.74 (s, 6H). **^{13}C{H} NMR:** (101 MHz, $CDCl_3$) δ = 159.8, 143.6, 138.7, 132.8, 132.6, 129.7,

128.5, 128.2, 128.0, 127.2, 126.8, 122.2, 113.9, 92.6, 88.0, 55.1. **IR:** (neat) ṽ = 3055, 3014, 2954, 2931 ,2906 ,2834, 2538, 2210, 1605, 1592, 1568, 1508, 1477, 1462, 1439, 1416, 1363, 1302, 1286, 1245, 1174, 1146, 1104, 1028, 946, 910, 875, 843, 828, 784, 754, 716.4, 688, 666, 652, 641, 631, 619, 587, 560, 528, 513, 478, 458, 448 cm^{-1}. **HRMS:** calcd m/z. for; $C_{40}H_{28}O^+$ $[M]^+$: 540.2089; found (EI) 540.2087.

Compound 3d

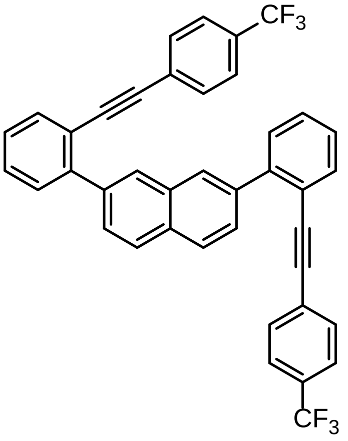

Prepared from **4** (100 mg, 0.24 mmol, 1.0 equiv), **5d** (273 mg, 0.94 mmol, 4.0 equiv), Pd_2dba_3 (10.8 mg, 0.012 mmol, 5 mol%), SPhos (9.68 mg, 0.023 mmol, 10 mol%), K_3PO_4 (200 mg, 0.94 mmol, 4.0 equiv.) and the specified solvent mixture (20 mL) according to general procedure **B**. Column chromatography (SiO_2, 0 to 10% EtOAc in pentane) yielded the product as a white solid (120 mg, 0.21 mmol, 88%).
^{1}H NMR: (300 MHz, $CDCl_3$) δ = 8.14 (s, 1H), 8.00 (d, *J* = 8.5 Hz, 1H), 7.88 (dd, *J* = 8.5, 1.7 Hz, 1H), 7.72 (dd, *J* = 7.5, 1.4 Hz, 1H), 7.54 (td, *J* = 7.5, 1.6 Hz, 1H), 7.50 – 7.44 (m, 3H), 7.41 (td, *J* = 7.5, 1.6 Hz, 1H), 7.35 (d, *J* = 8.1 Hz, 2H). **^{19}F{H} NMR:** (282 MHz, $CDCl_3$) δ = -62.8. **^{13}C{H} NMR:** (101 MHz, $CDCl_3$) δ = 144.1, 138.4, 133.1, 133.1, 132.0, 131.5, 131.5, 130.1 (q, J_{CF} = 32.7 Hz), 129.8, 129.2, 128.5, 128.0, 127.9, 127.4, 127.1, 127.1, 125.1 (q, J_{CF} = 3.8 Hz), 123.9 (q, J_{CF} = 272.2 Hz), 122.5, 121.2, 91.8, 91.1. **IR:** (neat) ṽ = 3059, 3026, 2214, 2048, 1914, 1786, 1666, 1614, 1572, 1516, 1478, 1440, 1404, 1321, 1229, 1185, 1170, 1156, 1116, 1104, 1065, 1015, 978, 964, 948, 911, 901, 872, 856, 835, 769, 754, 710, 683, 669, 643, 631, 620, 594, 553, 541, 510, 493, 478, 462, 453, 445, 438, 428, 419 cm^{-1}. **HRMS:** calcd m/z. for; $C_{40}H_{22}F_6^+$ $[M]^+$: 616.1626; found (EI) 616.1624.

Synthesis of the compounds 8a and 8b

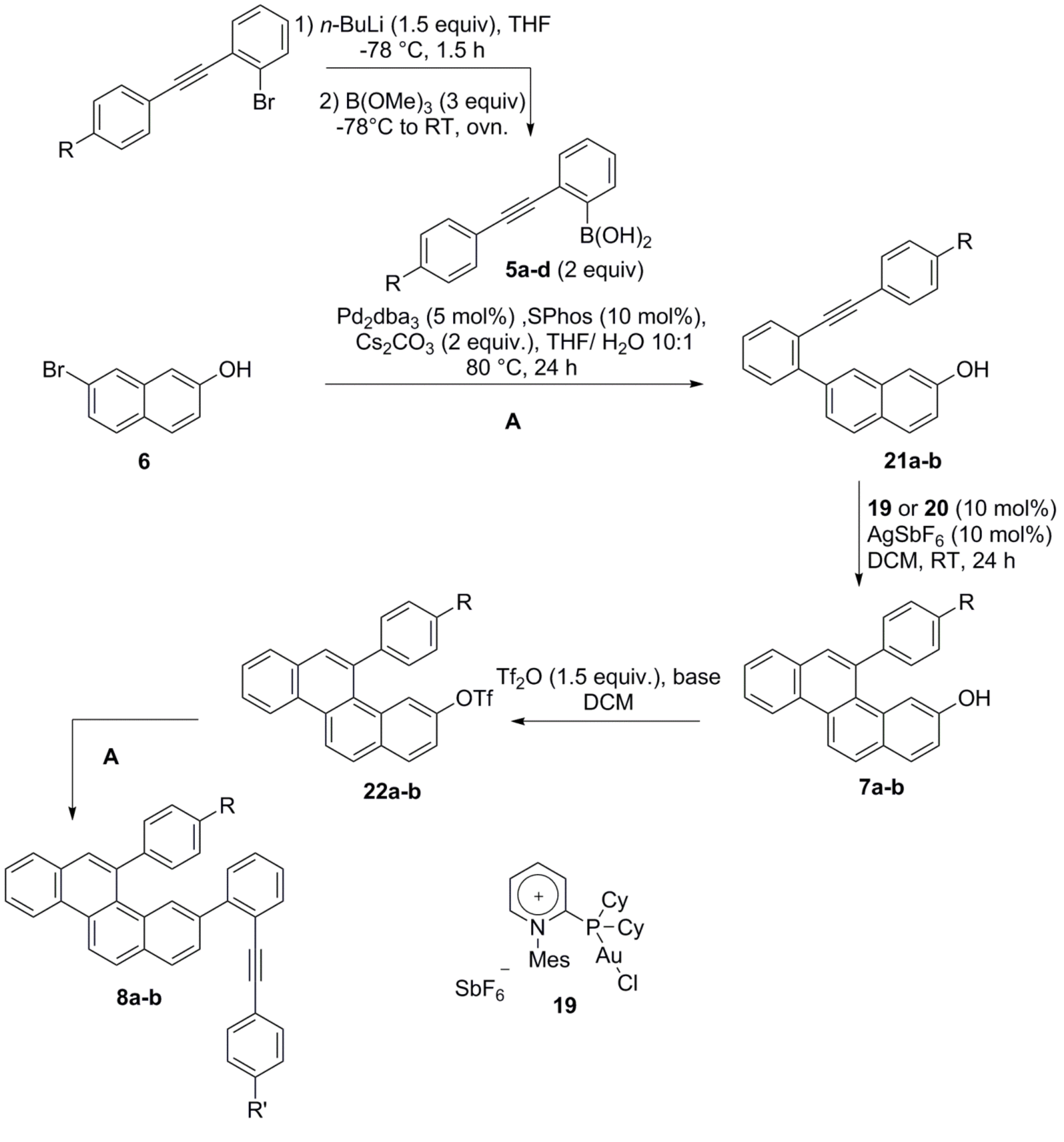

Scheme 2 Synthesis of unsymmetrical Tetrahelicene Precursors

Compound 21a

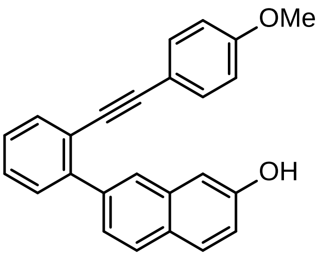

Prepared from **6** (517 mg, 2.32 mmol, 1.0 equiv), **5c** (996 mg, 7.32 mmol, 1.5 equiv), Pd_2dba_3 (106 mg, 116 µmol, 5 mol%,), SPhos (95.2 mg, 232 µmol, 10 mol%,) and according to general procedure **B**. Two column chromatographies (SiO_2, 0 to 20% EtOAc in pentane) yielded the product as a brown oil (613 mg, 1.75 mmol, 76%).

^{1}H NMR (400 MHz, $CDCl_3$) δ = 7.96 (d, *J* = 1.6 Hz, 1H), 7.82 (dd, *J* = 13.6, 8.6 Hz, 2H), 7.71 – 7.63 (m, 2H), 7.51 (dd, *J* = 7.7, 1.4 Hz, 1H), 7.41 (tt, *J* = 7.7, 1.2 Hz, 1H), 7.35 (tt, *J* = 7.5, 1.2 Hz, 1H), 7.25 – 7.19 (m, 3H),

7.13 (dd, *J* = 8.8, 2.5 Hz, 1H), 6.78 (d, *J* = 8.6 Hz, 2H), 4.96 (s, 1H), 3.78 (s, 2H). **^{13}C NMR** (101 MHz, $CDCl_3$) δ = 159.6, 153.7, 143.6, 139.1, 134.5, 132.9, 132.9, 129.8, 129.7, 128.4, 128.3, 127.3, 127.2, 126.9, 125.8, 122.2, 118.0, 115.7, 114.1, 109.9, 92.7, 88.3, 55.4. **IR** (neat): $\tilde{\nu}$ (cm^{-1}) = 3429, 3060, 2971, 2833, 1738, 1605, 1510, 1443, 1366, 1288, 1250, 1175, 1108, 1030, 832, 756, 637, 532. **HRMS** calc. for $C_{25}H_{19}O_2^+$: 351.1385; found: 351.1378 $[M+H]^+$ (ESI).

Compound 21b

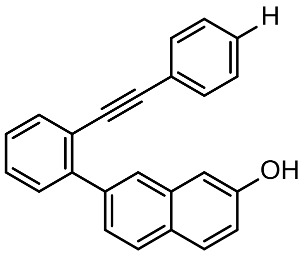

Prepared from **6** (400 mg, 1.79 mmol, 1.0 equiv), **5a** (597 mg, 2.69 mmol, 1.5 equiv), Pd_2dba_3 (82.0 mg, 89.7 µmol, 5 mol%), SPhos (73.6 mg, 179 µmol, 10 mol%) and according to general procedure **B**. Column chromatography (SiO_2, 0 to 10% EtOAc in pentane) yielded the product as a yellow oil (485 mg, 1.51 mmol,85%).

^{1}H NMR (400 MHz, $CDCl_3$) δ = 7.99 (s, 1H), 7.86 (dd, *J* = 14.7, 8.5 Hz, 2H), 7.71 (d, *J* = 8.8 Hz, 2H), 7.57 – 7.54 (t, *J* = 8.1 Hz, 1H), 7.46 (t, *J* = 7.6 Hz, 1H), 7.39 (t, *J* = 7.5 Hz, 1H), 7.35 – 7.21 (m, 6H), 7.16 (dd, *J* = 8.8, 2.2 Hz, 1H), 4.93 (s, 1H). **^{13}C NMR** (101 MHz, $CDCl_3$) δ = 153.6, 143.8, 138.8, 134.4, 133.0, 131.4, 129.7, 129.6, 128.6, 128.2, 128.2, 128.1, 127.2, 127.1, 126.8, 125.6, 123.4, 121.8, 117.9, 109.8, 92.4, 89.4. **IR:** (neat) ṽ = 3374, 3056, 3017, 2925, 2848, 2360, 2342, 2255, 2218, 2204, 2193, 2181, 2163, 2007, 1990, 1696, 1631, 1595, 1576, 1557, 1539, 1512, 1494, 1479, 1461, 1442, 1389, 1349, 1302, 1272, 1206, 1169, 1127, 1099, 1067, 1021, 961, 915, 897, 839, 794, 755, 706, 690, 674, 641, 631, 609, 586, 536, 511, 484, 472, 462, 451, 437, 415, 406. **HRMS:** calcd m/z. for; $C_{24}H_{16}O^+$ $[M+H]^+$: 321.1274; found (ESI) 321.1276.

General procedure C for racemic cycloisomerisations[4]

A dried Schlenk equipped with a magnetic stirring bar flask was loaded with the respective alkyne (1 equiv.) and the gold precatalyst **19** (10 mol%). After drying *in vacuo* for 1 h, DCM (0.05 M) was added. To the stirred mixture $AgSbF_6$ (10 mol%, 0.05 M in DCM) was added dropwise. After stirring overnight at room temperature the mixture was filtered through a silica plug eluting with DCM. Evaporation of the solvent yielded a crude product which was purified by column chromatography (SiO_2, EtOAc in pentane) or recrystallization.

Compound 7a

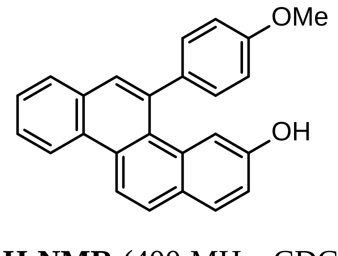

Prepared using **21** (231 mg, 660 µmol, 1.0 equiv.), gold pre-catalyst **19** (9 mol%), DCM (13 mL) and $AgSbF_6$ (9 mol%, 19.3 mg, 56.1 µmol, 0.05 M in DCM) according to general procedure **C**. The crude compound was filtered through a short pad of silica, eluting with DCM and EtOAc to afford the product without further purification as a yellow solid (201 mg, 573 µmol, 87% yield). Colourless needles were obtained by recrystallization from chloroform.

^{1}H NMR (400 MHz, $CDCl_3$) δ = 8.76 (d, *J* = 8.3 Hz, 1H), 8.64 (d, *J* = 9.1 Hz, 1H), 7.94 (t, *J* = 9.1 Hz, 2H), 7.83 (d, *J* = 8.6 Hz, 1H), 7.77 (s, 1H), 7.68 (ddd, *J* = 8.4, 6.9, 1.4 Hz, 1H), 7.63 (ddd, *J* = 8.0, 6.9, 1.3 Hz, 1H), 7.44 – 7.36 (m, 2H), 7.14 (d, *J* = 2.4 Hz, 1H), 7.09 (dd, *J* = 8.6, 2.3 Hz, 1H), 7.07 – 7.00 (m, 2H), 4.52 (bs, 1H), 3.92 (s, 3H). **^{13}C NMR** (101 MHz, $CDCl_3$) δ = 159.0, 152.5, 138.1, 137.9, 132.2, 131.7, 130.7, 130.6, 130.3, 130.2, 130.1, 128.5, 128.4, 127.9, 126.9, 126.7, 126.6, 123.4, 119.2, 116.5, 114.4, 112.8, 55.6. **IR** (neat): $\tilde{\nu}$ (cm^{-1}) = 3012, 2970, 2948, 1738, 1558, 1541, 1507, 1473, 1457, 1436, 1365, 1228, 1217, 527. **HRMS** calc. for $C_{25}H_{19}O_2^+$: 351.1385; found: 351.1370 $[M+H]^+$ (ESI).

Compound 7b

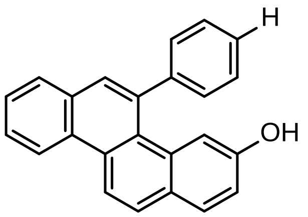

Prepared from **22** (485 mg, 1.51 mmol, 1.0 equiv.), **19** (10 mol%), $AgSbF_6$ (52.0 mg, 151 µmol, 10 mol%) in DCM according to general procedure **B**, stirring at RT for 24 h. Column chromatography (SiO_2, 0 to 5% EtOAc in pentane) yielded a yellow solid (240 mg, 750 µmol, 50 % yield). **^{1}H NMR** (400 MHz, $CDCl_3$) δ = 8.77 (d, *J* = 8.3 Hz, 1H), 8.65 (d, *J* = 9.0 Hz, 1H), 7.99 – 7.92 (m, 2H), 7.83 (d, *J* = 8.6 Hz, 1H), 7.80 (s, 1H), 7.73 – 7.67 (m, 1H), 7.64 (ddd, *J* = 7.9, 6.9, 1.2 Hz, 1H), 7.50 (d, *J* = 4.2 Hz, 5H), 7.08 (dd, *J* = 8.6, 2.4 Hz, 1H), 7.02 (d, *J* = 2.4 Hz, 1H), 4.46 (s, 1H). **^{13}C NMR** (101 MHz, $CDCl_3$) δ = 152.3, 145.3, 138.3, 131.9, 131.5, 130.5, 130.4, 130.1, 130.0, 129.2, 128.9, 128.4, 128.3, 127.8, 127.1, 126.9, 126.7, 126.2, 123.3, 119.0, 116.3, 112.8. **IR:** (neat) ṽ = 3415, 3090, 3046, 3030, 2958, 2924, 2853, 2361, 2334, 2323, 2158, 2009, 1979, 1944, 1910, 1884, 1820, 1776, 1736, 1618, 1604, 1553, 1535, 1519, 1493, 1476, 1441, 1421, 1366, 1353, 1325, 1286, 1253, 1243, 1210, 1155, 1093, 1071, 1027, 973, 953, 908, 891, 867, 849, 833, 813, 784, 762, 744, 696, 661, 632, 610, 590, 567, 553, 528, 511, 476, 461, 446, 438, 420, 405 cm^{-1}. **HRMS:** calcd m/z. for; $C_{24}H_{16}O^+$ $[M+H]^+$: 321.1274; found (ESI) 321.1272.

General procedure D for the preparation of aromatic triflates[1]

Adapted from a literature procedure. A dried Schlenk flask equipped with a magnetic stirring bar was charged with the respective aryl alcohol (1.0 equiv.). DCM (0.05 M) and the appropriate base (Et_3N (0.1 M) or pyridine (4 equiv.)) were added and the stirred mixture cooled to 0 °C. Triflic anhydride (1.5 equiv.) was added dropwise, afterwards the reaction mixture warmed to room temperature and left to stir for 16 h. The reaction was quenched by the addition of water. The phases were separated, the aqueous phase was extracted with DCM (3 x 25 mL) and the combined organic phases dried over Na_2SO_4. The solvent was removed *in vacuo* and the crude mixture purified by column chromatography (SiO_2, EtOAc in pentane).

Compound 22a

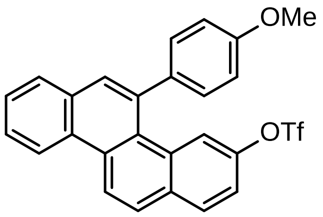

Prepared using **7a** (95 mg, 272 µmol, 1.0 equiv.), triflic anhydride (115 mg, 408 µmol, 1.5 equiv), DCM (7 mL) and pyridine (97 mg, 1.24 mmol, 4 equiv.) according to general procedure **D**. The crude compound was purified by column chromatography (SiO_2, 5% EtOAc in pentane) to afford the product as a white solid (111 mg, 231 µmol, 85%). **^{1}H NMR** (400 MHz, $CDCl_3$) δ = 8.85 (d, *J* = 9.1 Hz, 1H), 8.77 (dd, *J* = 8.0, 1.0 Hz, 1H), 8.02 (d, *J* = 9.0 Hz, 1H), 8.00 – 7.94 (m, 2H), 7.87 (s, 1H), 7.76 (d, *J* = 2.5 Hz, 1H), 7.75 – 7.65 (m, 2H), 7.39 – 7.34 (m, 3H), 7.12 – 7.03 (m, 2H), 3.93 (s, 3H). **^{13}C NMR** (101 MHz, $CDCl_3$) δ = 159.5, 146.2, 137.9, 136.6, 132.7, 131.9, 131.7, 131.5, 130.8, 130.4, 130.0, 129.9, 128.5, 127.5, 127.5, 127.3, 127.1, 123.3, 122.9, 120.9, 119.3, 118.7 (q, *J* = 320.8 MHz), 115.1, 55.6. **^{19}F NMR** (377 MHz, $CDCl_3$) δ = -73.3 **IR** (neat): $\tilde{\nu}$ (cm^{-1}) = 3104, 3015, 2971, 2937, 2842, 1739, 1607, 1509, 1474, 1456, 1419, 1364, 1284, 1245, 1203, 1174, 1138, 1030, 945, 924, 888, 873, 837, 824, 753, 726, 647, 634, 592, 563, 514, 504. **HRMS** calc. for $C_{26}H_{18}F_3O_4S^+$: 483.0878; found: 483.0878 $[M+H]^+$ (ESI).

Compound 22b

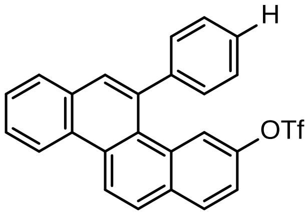

Prepared using **7b** (240 mg, 749 µmol, 1.0 equiv.), triflic anhydride (232 mg, 824 µmol, 1.5 equiv), DCM (7 mL) and pyridine (237 mg, 2.99 mmol, 4 equiv.) according to general procedure **D**. The crude compound was purified by column chromatography (SiO_2, 5% EtOAc in pentane) to afford the product as a white solid (170 mg, 375 µmol, 50%).

^{1}H NMR (400 MHz, $CDCl_3$) δ = 8.90 (d, *J* = 9.0 Hz, 1H), 8.82 (d, *J* = 8.4 Hz, 1H), 8.07 (d, *J* = 9.1 Hz, 1H), 8.03 – 7.97 (m, 2H), 7.91 (s, 1H), 7.77 (ddd, *J* = 8.4, 6.9, 1.6 Hz, 1H), 7.74 – 7.69 (m, 2H), 7.59 – 7.50 (m, 3H), 7.48 (dt, *J* = 7.3, 1.4 Hz, 2H), 7.39 (dd, *J* = 8.7, 2.3 Hz, 1H).**^{13}C NMR** (101 MHz, $CDCl_3$) δ = 146.1, 144.2, 138.1, 132.5, 131.7, 131.4, 131.4, 130.6, 130.3, 129.8, 129.4, 128.8, 128.5, 127.6, 127.4, 127.2, 127.1, 127.0, 123.2, 122.8, 120.7, 119.2, 118.5 (q, J_{CF}= 321 Hz). **IR:** (neat) ṽ = 3108, 3069, 3027, 2961, 2925, 2851, 2660, 2549, 2354, 2342, 2319, 2172, 2018, 1982, 1960, 1928, 1899, 1799, 1779, 1765, 1741, 1700, 1678, 1620, 1598, 1588, 1516, 1489, 1473, 1427, 1419, 1403, 1364, 1345, 1307, 1242, 1214, 1168, 1157, 1141, 1131, 1072, 1044, 1028, 1002, 988, 978, 964, 947, 932, 914, 890, 873, 855, 846, 840, 816, 786, 768, 750, 732, 704, 679, 661, 634, 621, 596, 584, 560, 535, 522, 512, 496, 475, 456, 435, 418, 408. **HRMS:** calcd m/z. for; $C_{25}H_{15}O_3SF_3^+$ $[M]^+$: 453.0767; found (EI) 453.0750.

Compound 8a

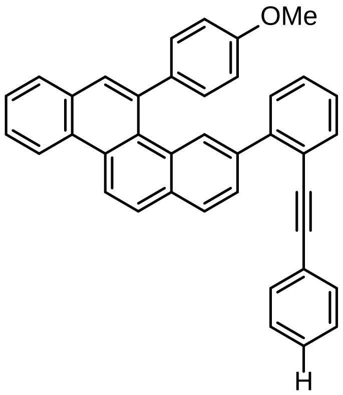

Prepared using **23** (97 mg, 200 µmol, 1.0 equiv.), boronic acid **5a** (89 mg, 401 µmol, 2 equiv.), $CsCO_3$ (77 mg, 401 µmol, 2.0 equiv), $Pd_2(dba)_3$ (9.2 mg, 10.0 µmol, 5 mol%), SPhos (8.2 mg, 20.0 µmol, 10 mol%) and THF/H_2O (10:1, 7 mL) according to general procedure **B**. The crude compound was filtered through a pad of silica, eluting with DCM and EtOAc. The crude mixture was further purified by column chromatography (SiO_2, 0 to 1% EtOAc in pentane), followed by a crystallization from a dichloromethane/pentane bilayer to afford the product as green needles (71 mg, 139 µmol, 70% yield).

^{1}H NMR (300 MHz, $CDCl_3$) δ = 8.80 (dd, *J* = 8.7, 3.4 Hz, 2H), 8.10 – 8.03 (m, 4H), 8.00 (s, 1H), 7.98 – 7.92 (m, 1H), 7.84 (s, 1H), 7.75 – 7.55 (m, 3H), 7.44 – 7.37 (m, 2H), 7.32 – 7.16 (m, 6H), 7.08 – 7.00 (m, 2H), 6.95 – 6.87 (m, 1H), 3.91 (s, 3H). **^{13}C NMR** (101 MHz, $CDCl_3$) δ = 159.1, 143.9, 138.4, 138.2, 136.8, 132.9, 132.7, 131.7, 131.4, 130.7, 130.7, 130.3, 130.2, 130.2, 129.9, 128.5, 128.3, 128.2, 127.8, 127.6, 127.1, 127.1, 126.9, 126.7, 123.6, 123.3, 121.6, 121.6, 114.8, 92.3, 89.7, 55.6. **IR** (neat): $\tilde{\nu}$ (cm^{-1}) = 3057, 1606, 1510, 1471, 1282, 1244, 1175, 1033, 907, 838, 754, 733, 690, 588. **HRMS** calc. for $C_{39}H_{27}O^+$: 511.2062; found: 511.2051 $[M+H]^+$ (ESI).

Compound 8b

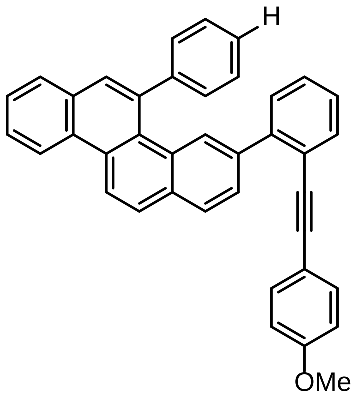

Prepared using **23** (170 mg, 375 µmol, 1.0 equiv.), boronic acid **5a** (189 mg, 751 µmol, 2 equiv.), $CsCO_3$ (245 mg, 751 µmol, 2.0 equiv), $Pd_2(dba)_3$ (17.2 mg, 18.8 µmol, 5 mol%), SPhos (15.4 mg, 37.6 µmol, 10 mol%) and THF/H_2O (10:1, 15 mL) according to general procedure **B**. The crude compound was filtered through a pad of silica, eluting with DCM. Purification by column chromatography (SiO_2, 0 to 1% EtOAc in pentane) and subsequent preparative HPLC (20% to 0% H_2O in MeCN over 20 min, 255 K, 1.0mL/min) yielded the product as an off-white solid (94 mg, 184 µmol, 50%).

^{1}H NMR (400 MHz, $CDCl_3$) δ = 8.83 (dd, *J* = 8.8, 3.5 Hz, 2H), 8.14 – 8.06 (m, 3H), 8.04 – 7.95 (m, 2H), 7.86 (s, 1H), 7.74 (t, *J* = 7.6 Hz, 1H), 7.67 (t, *J* = 7.5 Hz, 1H), 7.56 (d, *J* = 7.5 Hz, 1H), 7.54 – 7.44 (m, 5H), 7.32 – 7.20 (m, 4H), 6.81 (d, *J* = 7.5 Hz, 1H), 6.77 (d, *J* = 8.7 Hz, 2H), 3.78 (s, 3H). **^{13}C NMR** (101 MHz, $CDCl_3$) δ = 159.4, 145.9, 143.3, 138.4, 136.8, 132.7, 132.5, 131.5, 130.7, 130.4, 130.1, 130.0, 129.9, 129.8, 129.2, 129.1, 128.4, 127.9, 127.7, 127.7, 127.6, 126.9, 126.9, 126.8, 126.7, 126.7, 123.1, 121.7, 121.4, 115.6, 113.9, 113.9, 92.2, 88.3, 55.2. **IR:** (neat) ṽ = 3055, 3021, 2953, 2924, 2853, 2836, 2536, 2360, 2342, 2212, 2181, 2172, 2161, 2025, 1977, 1960, 1949, 1899, 1605, 1594, 1568, 1510, 1493, 1469, 1440, 1413, 1394, 1303, 1287, 1249, 1208, 1174, 1147, 1105, 1073, 1031, 980, 949, 932, 892, 832, 814, 786, 753, 722, 703, 661, 639, 610, 597, 589, 573, 553, 535, 518, 499, 490, 475, 462, 448, 427, 418, 402. **HRMS:** calcd m/z. for; $C_{39}H_{26}O^+$ $[M]^+$: 511.2056; found (EI) 511.2050.

Synthesis of Compounds 18a-j

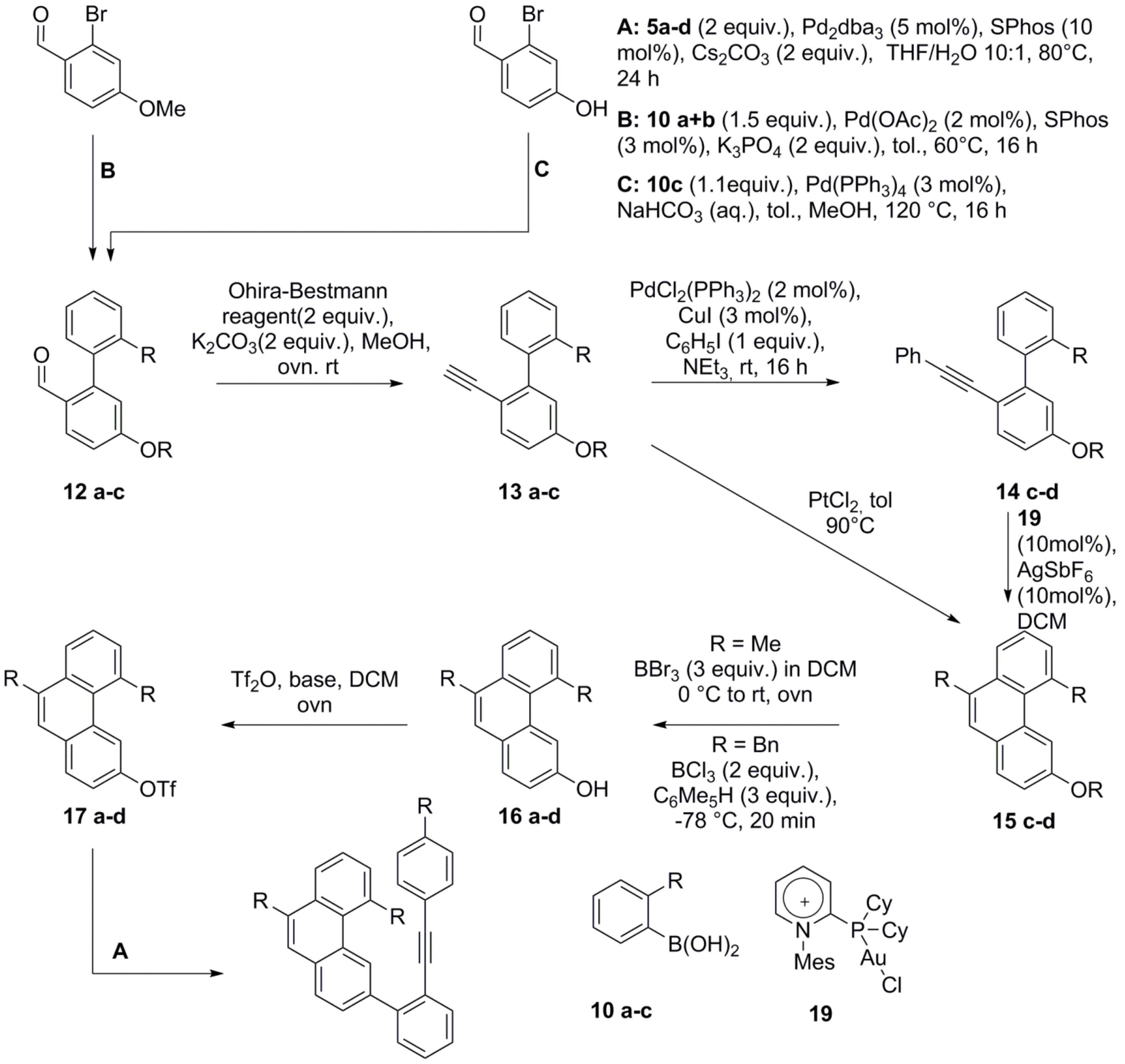

Scheme 3 Synthesis of unsymmetrical phenanthrene-based Tetrahelicene Precursors

Compound 12a

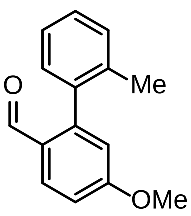

Adapted from a literature procedure.[6] A dried Schlenk was charged with 2-bromo-4-methoxybenzaldehyd (1.050 g, 4.88 mmol, 1.0 equiv), followed by (2-methylphenyl)boronic acid (996 mg, 7.32 mmol, 1.5 equiv.), SPhos (60.1 mg, 146.5 µmol, 3 mol%), K_3PO_4 (2.070 g, 9.77 mmol, 2.0 equiv.) and $Pd(OAc)_2$ (21.9 mg, 97.7 µmol, 2 mol%). Toluene (21 mL) was added *via* syringe and the mixture heated to 60 °C for 16 h. When the reaction had reached completion, it was allowed to cool to room temperature and filtered through a pad of celite, eluting with EtOAc. The solvents were removed *in vacuo* and the crude compound purified by column chromatography (SiO_2, 0 to 5% EtOAc in pentane) to afford the product as a yellow solid (1.010 g, 4.47 mmol, 92%).

^{1}H NMR (300 MHz, $CDCl_3$) δ = 9.58 (d, *J* = 0.8 Hz, 1H), 8.01 (d, *J* = 8.7 Hz, 1H), 7.38 – 7.26 (m, 3H), 7.21 (td, *J* = 8.2, 7.6, 1.5 Hz, 1H), 7.00 (dt, *J* = 8.8, 1.6 Hz, 1H), 6.76 (d, *J* = 2.5 Hz, 1H), 3.89 (s, 3H), 2.12 (s, 3H). **^{13}C NMR** (101 MHz, $CDCl_3$) δ = 191.0, 163.9, 148.3, 137.7, 136.2, 130.2, 130.0, 129.6, 128.4, 127.7, 125.8, 115.3, 114.1, 55.8, 20.4. **IR** (neat): $\tilde{\nu}$ (cm^{-1}) = 3063, 3017, 2968, 2943, 2840, 2755, 1739, 1682, 1594, 1561, 1485, 1457, 1440, 1394, 1365, 1330, 1300, 1279, 1254, 1224, 1177, 1126, 1103, 1031, 1018, 885, 814, 781, 765, 730, 653, 565. **HRMS** calc. for $C_{15}H_{15}O_2^+$: 227.1072; found: 227.1067 $[M+H]^+$ (ESI).

Compound 12b

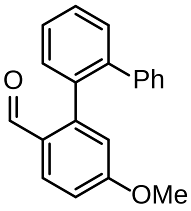

Adapted from a literature procedure.[6] A dried Schlenk was charged with 2-bromo-4-methoxybenzaldehyde (1.00 g, 4.65 mmol. 1.0 equiv.), 2-biphenylboronicacid (1.38 g, 6.98 mmol, 1.5 equiv,), $Pd(OAc)_2$ (20.8 mg, 93.0 µmol, 2 mol%), SPhos (57.3 mg, 140 µmol, 3 mol%), K_3PO_4 (1.97 g, 9.30 mmol, 2.0 equiv.). Toluene (24 mL) was added and the mixture was heated to 60 °C for 16 h. After cooling to room temperature the mixture was filtered through a pad of celite eluting with EtOAc. The solvents were removed *in vacuo* and the crude product was purified by column chromatography (SiO_2, 5% EtOAc in pentane), yielding the product as a white solid (1.22 g, 91 % yield).

^{1}H NMR (300 MHz, $CDCl_3$) δ = 9.67 (s, 1H), 7.82 (d, *J* = 8.7 Hz, 1H), 7.58 – 7.42 (m, 3H), 7.41 (ddd, *J* = 6.8, 6.1, 1.9 Hz, 1H), 7.29 – 7.10 (m, 3H), 7.16 – 7.04 (m, 2H), 6.88 (ddd, *J* = 8.7, 2.6, 0.8 Hz, 1H), 6.75 (d, *J* = 2.5 Hz, 1H), 3.77 (s, 3H). **^{13}C NMR** (101 MHz, $CDCl_3$) δ = 190.3, 163.4, 147.8, 141.9, 140.4, 136.4, 131.2, 130.2, 129.7, 129.6, 129.5, 128.7, 128.2, 128.1, 127.4, 127.4, 127.0, 116.3, 113.9, 55.6. **IR:** (neat) ṽ = 3351, 3057, 3020, 2939, 2838, 2756, 2618, 2251, 1958, 1739, 1675, 1591, 1561, 1490, 1475, 1448, 1432, 1396, 1330, 1297, 1241, 1212, 1175, 1125, 1103, 1074, 1057, 1031, 1015, 953, 910, 878, 861, 819, 801, 768, 743, 730, 699, 659, 633, 613, 567, 555, 523, 472, 417 cm^{-1}. **HRMS:** calcd m/z. for; $C_{20}H_{16}O_2^+$ $[M+H]^+$: 289.1223; found (ESI) 289.1223.

Compound 23

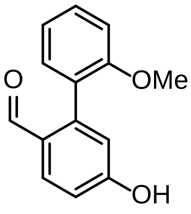

Adapted from a literature procedure.[7] A pressure tube was charged with 2-bromo-4-hydroxybenzaldehyde (500 mg, 2.49 mmol, 1.0 equiv.), (2-methoxyphenyl)boronic acid (416 mg, 2.74 mol, 1.1 equiv.) and $Pd(PPh_3)_4$ (86.2 mg, 74.6 µmol, 3 mol%). Sat. aq. sodium bicarbonate solution (8.5 mL), methanol (20 mL) and toluene (10 mL) were added and the mixture heated to 120 °C for 17 h. When the reaction had reached completion, it was allowed to cool to room temperature. EtOAc and water were added, and the layers separated. The aqueous phase was extracted with EtOAc and the combined organic layers were dried over Na_2SO_4. The solvents were removed *in vacuo* and the crude compound was purified by column chromatography (SiO_2, 0 to 5% EtOAc in pentane) to afford the product as a yellow solid (568 mg, 2.49 mmol, 99%).

^{1}H NMR (300 MHz, $CDCl_3$) δ = 9.63 (d, *J* = 0.7 Hz, 1H), 7.95 (d, *J* = 8.5 Hz, 1H), 7.45 – 7.38 (m, 1H), 7.25 (dd, *J* = 7.5, 1.8 Hz, 1H), 7.07 (td, *J* = 7.5, 1.0 Hz, 1H), 6.97 (d, *J* = 8.3 Hz, 1H), 6.91 (dd, *J* = 8.5, 2.6 Hz, 1H), 6.78 (d, *J* = 2.6 Hz, 1H), 5.47 (bs, 1H), 3.75 (s, 3H). **^{13}C NMR** (101 MHz, $CDCl_3$) δ = 191.5, 160.3, 156.6, 144.9, 131.4, 130.2, 129.6, 128.6, 128.0, 126.6, 121.0, 117.7, 115.2, 110.8, 55.6. **IR** (neat): $\tilde{\nu}$ (cm^{-1}) = 3248, 2954, 2929, 1668, 1591, 1492, 1463, 1434, 1396, 1319, 1245, 1200, 1125, 1107, 1053, 1022, 905, 824, 754. **HRMS** calc. for $C_{14}H_{13}O_3^+$: 229.0865; found: 229.0859 $[M+H]^+$ (ESI).

Compound 12c

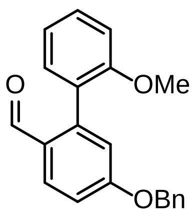

Adapted from a literature procedure.[8] A dried Schlenk flask was charged with **23** (601 mg, 2.63 mmol, 1.0 equiv.) and K_2CO_3 (364 mg, 2.63 mmol, 1.0 equiv.). Benzyl bromide (313 µL, 2.63 mmol, 1.0 equiv.) and acetonitrile (8 mL) were added and the mixture refluxed for 17 h. The solvent was removed *in vacuo* and a mixture of DCM/water (1:1) was added while stirring. The phases were separated, and the aqueous layer extracted with DCM (3 x 50 mL). The combined organic phases were dried over $MgSO_4$ and the solvent removed *in vacuo*. The crude compound was purified by column chromatography (SiO_2, 0 to 5% EtOAc in pentane) to afford the product as a white solid (728 mg, 2.29 mmol, 87%).
^{1}H NMR (300 MHz, $CDCl_3$) δ = 9.67 (s, 1H), 8.01 (d, *J* = 8.7 Hz, 1H), 7.45 – 7.34 (m, 6H), 7.26 (dd, J = 7.3, 1.9 Hz, 1H), 7.09 (t, *J* = 7.7 Hz, 2H), 7.00 (d, *J* = 8.3 Hz, 1H), 6.94 (d, *J* = 2.6 Hz, 1H), 5.17 (s, 2H), 3.77 (s, 3H). **^{13}C NMR** (101 MHz, $CDCl_3$) δ = 191.5, 163.1, 156.6, 144.5, 136.3, 131.5, 130.1, 129.2, 128.8, 128.4, 128.0, 127.7, 126.9, 121.0, 116.9, 114.5, 110.8, 70.4, 55.6. **IR** (neat): $\tilde{\nu}$ (cm^{-1}) = 3458, 3015, 2970, 2946, 2358, 2338, 1738, 1436, 1365, 1228, 1217, 1206, 904, 538, 527, 515. **HRMS** calc. for $C_{21}H_{19}O_3^+$: 319.1334; found: 319.1329 $[M+H]^+$ (ESI).

General procedure E for the synthesis of 2-(alkynyl)biphenylenes via Seyferth-Gilbert Homologation

Adapted from a literature procedure.[9] A dried Schlenk flask equipped with a magnetic stirring bar was loaded with the respective aldehyde (1 equiv.), pre-dried K_2CO_3 (3 equiv.) and dissolved in dry methanol (0.06 M) (for methanol insoluble aldehydes, THF was added until the aldehydes dissolved). The Ohira Bestmann reagent (3 equiv.) was added and the mixture stirred overnight. Dilution with Et_2O, washing with aq. $NaHCO_3$ solution and drying over $MgSO_4$ yielded a crude product. Column chromatography (SiO_2, EtOAc in pentane) yielded the desired alkynes.

Compound 13a

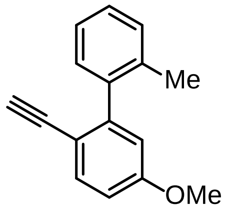

Prepared using **12a** (1.01 g, 4.46 mmol, 1.0 equiv.), K_2CO_3 (1.230 g, 8.93 mmol, 2.0 equiv.), the Ohira-Bestmann reagent (1.57 g, 8.93 mmol, 2.0 equiv.) and methanol (67 mL) according to general procedure **E**. The crude compound was purified by column chromatography (SiO_2, 0 to 1% EtOAc in pentane) to afford the product as yellow oil (941 mg, 4.23 mmol, 95%).
^{1}H NMR (300 MHz, $CDCl_3$) δ = 7.52 (d, *J* = 8.5 Hz, 1H), 7.35 – 7.27 (m, 2H), 7.21 (td, *J* = 7.5, 7.0, 2.2 Hz, 2H), 6.86 (dd, *J* = 8.6, 2.7 Hz, 1H), 6.76 (d, *J* = 2.7 Hz, 1H), 3.83 (s, 3H), 2.82 (s, 1H), 2.20 (s, 3H). **^{13}C NMR** (101 MHz, $CDCl_3$) δ = 159.7, 146.7, 140.6, 136.2, 134.4, 129.9, 129.5, 127.8, 125.5, 115.1, 114.1, 113.1, 82.8, 78.3, 55.5, 20.0. **IR** (neat): $\tilde{\nu}$ (cm^{-1}) = 3015, 2970, 2946, 1738, 1558, 1541, 1522, 1507, 1473, 1456, 1436, 1366, 1228, 1216, 901, 539, 527, 517. **HRMS** calc. for $C_{16}H_{15}O^+$: 223.1123; found: 223.1117 $[M+H]^+$ (ESI).

Compound 13b

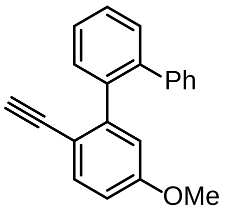

Prepared using **12b** (1.2 g, 4.16 mmol, 1.0 equiv.), K_2CO_3 (1.73 g, 12.5 mmol, 3.0 equiv.), the Ohira-Bestmann reagent (2.40 g, 12.5 mmol, 3.0 equiv.) and methanol (50 mL) according to general procedure **E**. The crude compound was purified by column chromatography (SiO_2, 0 to 1% EtOAc in pentane) to afford the product as colourless oil (1.18 g, 4.16 mmol, quant.).
^{1}H NMR (400 MHz, $CDCl_3$) δ = 7.47 (m, 3H), 7.44 – 7.37 (m, 2H), 7.22 – 7.13 (m, 5H), 6.72 (dd, *J* = 8.6, 2.7 Hz, 1H), 6.51 (d, *J* = 2.7 Hz, 1H), 3.59 (s, 3H), 2.86 (s, 1H). **^{13}C NMR** (101 MHz, $CDCl_3$) δ = 159.1, 146.1, 141.3, 141.2, 139.0, 134.2, 130.9, 130.0, 129.7, 129.7, 128.0, 127.7, 127.7, 126.7, 126.5, 115.8, 114.2, 113.3, 83.0, 78.7, 55.2. **IR:** (neat) $\tilde{\nu}$ = 3284, 3054, 3021, 2957, 2936,2835, 2363, 2333, 2105, 1604, 1557, 1489, 1474, 1447, 1434, 1415, 1320, 1301, 1288, 1235, 1218, 1175, 1132, 1111, 1072, 1035, 1016, 1007, 966, 952, 913, 883, 860, 814, 773, 744, 701, 664, 650 ,611, 553, 533, 514, 500, 482, 480 ,465, 455, 442, 430, 409. **HRMS:** calcd m/z. for; $C_{21}H_{16}O^+$ $[M+H]^+$: 285.1274; found (ESI) 285.1276.

Compound 13c

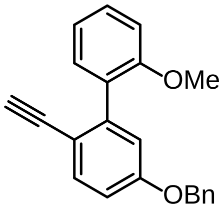

Prepared using 1**2c** (728 mg, 2.29 mmol, 1.0 equiv.), K_2CO_3 (632 mg, 4.58 mmol, 2 equiv.), the Ohira-Bestmann reagent (806 mg, 4.58 mmol, 2.0 equiv.) and methanol (34 mL) according to general procedure **E**. The crude compound was purified by column chromatography (SiO_2, 0 to 1% EtOAc in pentane) to afford the product as a yellow oil (622 mg, 1.98 mmol, 87%).
^{1}H NMR (400 MHz, $CDCl_3$) δ = 7.52 (d, *J* = 8.5 Hz, 1H), 7.47 – 7.30 (m, 6H), 7.28 (dd, *J* = 7.5, 1.8 Hz, 1H), 7.05 – 6.94 (m, 3H), 6.91 (dd, *J* = 8.5, 2.7 Hz, 1H), 5.08 (s, 2H), 3.78 (s, 3H), 2.85 (s, 1H). **^{13}C NMR** (101 MHz, $CDCl_3$) δ = 158.8, 156.8, 143.4, 136.8, 134.5, 131.3, 129.6, 129.3, 128.7, 128.2, 127.7, 120.4, 116.7, 114.8, 114.0, 111.2, 83.2, 77.8, 70.2, 55.7. **IR** (neat): $\tilde{\nu}$ (cm^{-1}) 3280, 2919, 2851, 1599, 1560, 1497, 1480, 1454, 1439, 1376, 1318, 1302, 1251, 1236, 1199, 1112, 1051, 1015, 907, 792, 752, 735, 710, 695, 648, 610. **HRMS** calc. for $C_{22}H_{19}O_2^+$: 315.1385; found: 315.1380 $[M+H]^+$ (ESI).

General Procedure F for the Sonogashira Couplings

Adapted from a literature procedure.[3] A dried Schlenk flask equipped with a magnetic stirring bar was charged with the alkyne (1.0 equiv.), $PdCl_2(PPh_3)_2$ (2 mol%), CuI (3 mol%) and Et_3N (0.38 m). The reaction mixture was degassed for several minutes under nitrogen flow. The appropriate Aryl halide (1.0 equiv.) was added dropwise via syringe and the reaction mixture left to stir for 16 h. When the reaction had reached completion, the mixture was filtered through a pad of celite, eluting with EtOAc. The organic layer was washed with a saturated aqueous solution of NH_4Cl, water and dried over Na_2SO_4 and the solvent removed in vacuo. The crude mixture was purified by column chromatography (SiO_2, EtOAc in pentane).

Compound 14c

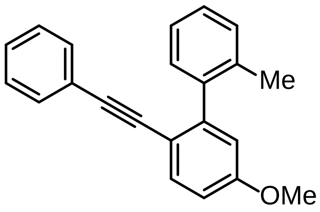

Prepared using **3a** (430 mg, 1.93 mmol, 1.0 equiv.), 1-iodobenzene (395 mg, 1.93 mmol, 1.0 equiv.), CuI (11.1 mg, 58.0 µmol, 3 mol%), $PdCl_2(PPh_3)_4$ (27.2 mg, 38.7 µmol, 2 mol%) and triethylamine (5 mL) according to general procedure **F**. The crude compound was purified by column chromatography (SiO_2, 0 to 5% EtOAc in pentane) to afford the product as a yellow oil (511 mg, 1.71 mmol, 89%).

^{1}H NMR (400 MHz, $CDCl_3$) δ = 7.58 (d, *J* = 8.5 Hz, 1H), 7.38 – 7.27 (m, 4H), 7.26 – 7.22 (m, 3H), 7.14 (dd, *J* = 6.7, 3.0 Hz, 2H), 6.92 (dd, *J* = 8.6, 2.7 Hz, 1H), 6.87 (d, *J* = 2.6 Hz, 1H), 3.86 (s, 3H), 2.28 (s, 3H). **^{13}C NMR** (101 MHz, $CDCl_3$) δ = 159.5, 146.5, 140.9, 136.4, 133.1, 131.2, 129.8, 129.8, 128.2, 127.7, 125.4, 123.8, 115.4, 115.1, 113.1, 91.0, 89.1, 55.5, 77.2, 20.1. **IR** (neat): $\tilde{\nu}$ (cm^{-1}) = 3016, 2970, 1738, 1595, 1557, 1441, 1366, 1300, 1227, 1217, 1035, 887, 757, 691, 527. **HRMS** calc. for $C_{22}H_{19}O^+$: 299.1436; found: 299.1430 $[M+H]^+$ (ESI).

Compound 14d

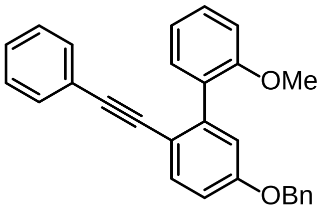

Prepared using 1**3c** (603 mg, 1.92 mmol, 1.0 equiv.), 1-iodobenzene (391 mg, 1.92 mmol, 1.0 equiv.), CuI (11.0 mg, 57.5 µmol, 3 mol%), $PdCl_2(PPh_3)_4$ (26.9 mg, 38.3 µmol, 2 mol%) and Et_3N (4.9 mL) according to general procedure **F**. The crude compound was purified by column chromatography (SiO_2, 0 to 5% EtOAc in pentane) to afford the product as a yellow oil (528 mg, 1.35 mmol, 71%).

^{1}H NMR (400 MHz, $CDCl_3$) δ = 7.55 (d, *J* = 8.5 Hz, 1H), 7.48 – 7.29 (m, 7H), 7.25 – 7.14 (m, 5H), 7.10 – 6.98 (m, 3H), 6.95 (dd, *J* = 8.6, 2.7 Hz, 1H), 5.10 (s, 2H), 3.77 (s, 3H). **^{13}C NMR** (101 MHz, $CDCl_3$) δ = 158.6, 157.0, 143.1, 136.9, 133.4, 131.5, 131.3, 129.9, 129.2, 128.7, 128.3, 128.2, 127.7, 127.7, 124.1, 120.3, 116.7, 115.9, 114.0, 111.1, 90.5, 89.6, 70.2, 55.8. **IR** (neat): $\tilde{\nu}$ (cm^{-1}) = 3028, 2970, 2946, 1738, 1593, 1557, 1541, 1506, 1491, 1473, 1455, 1435, 1366, 1301, 1274, 1229, 1217, 1204, 1125, 1109, 1093, 1051, 1027, 906, 808, 752, 733, 691, 540, 527, 516. **HRMS** calc. for $C_{28}H_{23}O_2^+$: 391.1698; found: 391.1693 $[M+H]^+$ (ESI).

General Procedure G for $PtCl_2$ catalysed cycloisomerisation

Adapted from a literature procedure.[10] The respective alkyne was dissolved in toluene (0.02) and $PtCl_2$ (10 mol%) was added. The mixture was stirred at 90 °C overnight. Filtration through a pad of celite, evaporation of the solvent and column chromatography (SiO_2, EtOAc in pentane) yielded the desired phenanthrenes.

Compound 15a

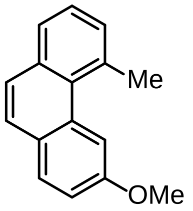

Prepared from **13a** (1.59 g, 7.16 mmol, 1.0 equiv.), $PtCl_2$ (191 mg, 0.72 mmol, 10 mol%) in toluene (30 mL) according to general procedure **G**, stirring at 90 °C for 16 h, column chromatography (SiO_2, 5% EtOAc in pentane) yielded the product as an off-white solid (0.96 g, 60 % yield).

^{1}H NMR (300 MHz, $CDCl_3$) δ = 8.39 (d, *J* = 2.4 Hz, 1H), 7.84 (d, *J* = 8.7 Hz, 1H), 7.75 (d, *J* = 5.6 Hz, 1H), 7.66 (d, *J* = 8.7 Hz, 1H), 7.59 (d, *J* = 8.7 Hz, 1H), 7.52 – 7.44 (m, 2H), 7.29 – 7.23 (m, 2H), 4.00 (s, 3H), 3.18 (s, 3H). **^{13}C NMR** (101 MHz, $CDCl_3$) δ = 157.4, 135.4, 134.1, 132.9, 130.9, 129.8, 129.7, 128.1, 127.5, 126.7, 126.0, 125.7, 115.0, 110.3, 55.5, 27.3. **IR:** (neat) ṽ = 3044, 2959, 2927, 2898, 2828, 2158, 2034, 1728, 1615, 1595, 1572, 1555, 1524, 1505, 1454, 1437, 1399, 1346, 1313, 1292, 1261, 1235, 1224, 1174, 1122, 1097, 1042, 1015, 961, 911, 883, 832, 817, 794, 760, 709, 699, 676, 653, 619, 518, 497, 483, 464, 445, 433, 423, 407. **HRMS:** calcd m/z. for; $C_{16}H_{14}O^+$ $[M]^+$: 222.1039; found (EI) 222.1038.

Compound 15b

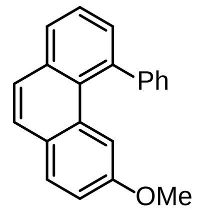

Prepared from **13b** (1.13 g, 4.19 mmol, 1.0 equiv.), $PtCl_2$ (112 mg, 0.42 mmol, 10 mol%) in toluene (30 mL) according to general procedure **G**, stirring at 90 °C for 16 h, column chromatography (SiO_2, 5% EtOAc in pentane) yielded the product as a white solid (1.06 g, 94 % yield).
^{1}H NMR (400 MHz, $CDCl_3$) δ = 7.92 (dd, *J* = 7.9, 1.5 Hz, 1H), 7.79 – 7.67 (m, 3H), 7.65 – 7.57 (m, 1H), 7.58 – 7.40 (m, 6H), 7.38 (d, *J* = 2.5 Hz, 1H), 7.11 (dd, *J* = 8.7, 2.5 Hz, 1H), 3.28 (s, 3H). **^{13}C NMR** (101 MHz, $CDCl_3$) δ = 156.8, 145.8, 140.5, 134.0, 131.7, 130.4, 129.7, 129.4, 129.1, 128.7, 128.0, 127.3, 127.1, 125.6, 125.2, 117.4, 109.2, 54.3. **IR:** (neat) ṽ = 3050, 3025, 3001, 2953, 2924, 2853, 1616, 1593, 1571, 1521, 1503, 1491, 1455, 1439, 1402, 1378, 1352, 1304, 1277, 1226, 1180, 1134, 1090, 1071, 1037, 964, 907, 887, 857, 836, 760, 719, 699, 617, 568, 523, 512, 448, 427 cm^{-1}. **HRMS:** calcd m/z. for; $C_{21}H_{16}O^+$ $[M]^+$: 284.1196; found (EI) 284.1194.

Compound 15c

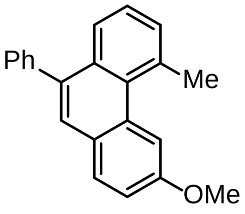

Prepared using **4a** (200 mg, 670 µmol, 1.0 equiv.), gold pre-catalyst **19** (57.8 mg, 67.0 µmol, 10 mol%) and $AgSbF_6$ (23.0 mg, 67.0 µmol, 10 mol%, 0.05 M in DCM) in DCM (3 mL) according to general procedure **C**. The crude compound was purified by column chromatography (SiO_2, 0 to 1% EtOAc in pentane) to afford the product as a green solid (146 mg, 488 µmol, 73%).
^{1}H NMR (500 MHz, $CDCl_3$) δ = 8.36 (d, *J* = 2.4 Hz, 1H), 7.83 (dt, *J* = 8.7, 0.4 Hz, 1H), 7.80 – 7.74 (m, 1H), 7.60 (s, 1H), 7.52 – 7.47 (m, 5H), 7.47 – 7.42 (m, 1H), 7.40 (dd, *J* = 8.2, 7.2 Hz, 1H), 7.28 (dd, *J* = 8.7, 2.4 Hz, 1H), 4.02 (s, 3H), 3.21 (s, 3H). **^{13}C NMR** (126 MHz, $CDCl_3$) δ = 157.5, 141.8, 137.1, 135.6, 133.3, 132.4, 130.9, 130.3, 130.1, 128.6, 128.4, 127.7, 127.6, 127.2, 125.9, 125.7, 115.6, 110.5, 55.7, 27.6. **IR** (neat): $\tilde{\nu}$ (cm^{-1}) = 3016, 2970, 1739, 1614, 1597, 1525, 1504, 1438, 1365, 1217, 891, 801, 770, 703, 527. **HRMS** calc. for $C_{22}H_{19}O^+$: 299.1436; found: 299.1430 $[M+H]^+$ (ESI).

Compound 15d

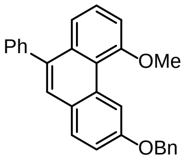

Prepared using 14d (603 mg, 1.54 mmol, 1.0 equiv.) 19 (133.2 mg, 154.4 µmol, 10 mol%), AgSbF6 (0.05 M in DCM, 53.0 mg, 154.4 µmol, 10 mol%,) in DCM (30 mL) according to general procedure C. The crude compound was directly used in the next step.

General Procedure H for the cleavage of methyl ethers

Adapted from a literature procedure.[11] In a dried Schlenk Flask equipped with a magnetic stirring bar the methyl-ether (1.0 equiv.) was dissolved in DCM (0.05 M) and cooled to 0 °C. A solution of BBr_3 (1M in DCM, 3 equiv.) was added dropwise. While maintaining stirring the solution was allowed to reach room temperature, stirring was continued over 16 h. The reaction mixture was poured into ice-cold water (40 mL), the phases separated and the organic phase extracted with DCM (3 x 40 mL). The combined organic phases were dried over Na_2SO_4 and the solvent was removed *in vacuo*. Column chromatography of the crude mixture (SiO_2, EtOAc in pentane) yielded the desired alcohols.

General Procedure I for the cleavage of benzyl ethers

Adapted from a literature procedure.[12] A dried Schlenk flask was charged with the respective benzyl-ether (1.0 equiv.) and pentamethylbenzene (3.0 equiv.). The mixture was dissolved in CH_2Cl_2 and cooled to -78 °C. BCl_3 (1M in DCM, 2 equiv.) was added dropwise. After 30 min the reddish solution was quenched with a DCM/MeOH-mixture(10:1). Evaporation of the solvents and column chromatography (SiO_2, EtOAc in pentane) yielded the desired alcohols.

Compound 16a

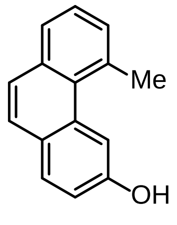

Prepared from **15a** (0.96 g, 4.32 mmol, 1.0 equiv.) and BBr_3 (1 M in DCM, 13 mL, 13 mmol, 3 equiv.) in DCM (20 mL) according to general procedure **H**. Column chromatography (SiO_2, 5% EtOAc in pentane) yielded the product as an off-white solid (0.89 g, 4.27 mmol, 99%).
^{1}H NMR (300 MHz, $CDCl_3$) δ = 8.35 (d, *J* = 2.4 Hz, 1H), 7.81 (d, *J* = 8.5 Hz, 1H), 7.78 – 7.71 (m, 1H), 7.70 – 7.54 (m, 2H), 7.52 – 7.42 (m, 2H), 7.17 (dt, *J* = 8.6, 1.6 Hz, 1H), 5.05 (s, 1H), 3.14 (s, 3H). **^{13}C NMR** (101 MHz, $CDCl_3$) δ = 153.4, 135.8, 134.3, 133.2, 131.0, 130.3, 129.5, 128.4, 127.7, 126.9, 126.2, 125.9, 115.6, 112.5, 27.5. **IR:** (neat) ṽ = 3344, 3049, 2959, 2925, 2871, 2846, 2016, 1653, 1615, 1558, 1530, 1506, 1447, 1418, 1396, 1312, 1261, 1209, 1144, 1125, 1097, 1033, 897, 834, 756, 669, 654, 618, 519, 472, 458, 418. **HRMS:** calcd m/z. for; $C_{15}H_{11}O^+$ $[M]^+$: 208.0883; found (EI) 208.0882.

Compound 16b

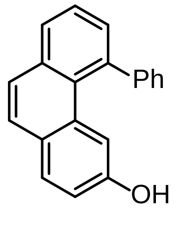

Prepared from **15b** (1.06 g, 3.73 mmol, 1.0 equiv.) and BBr_3 (1 M in DCM, 11 mL, 11 mmol, 3 equiv.) in DCM (18 mL) according to general procedure **H**. Column chromatography (SiO_2, 5% EtOAc in pentane) yielded the product as an off-white solid (0.86 g, 3.18 mmol, 85%).
^{1}H NMR (400 MHz, $CDCl_3$) δ = 7.89 (dd, *J* = 7.9, 1.5 Hz, 1H), 7.75 (d, *J* = 8.3 Hz, 1H), 7.68 (q, *J* = 8.7 Hz, 2H), 7.60 (t, *J* = 7.6 Hz, 1H), 7.54 – 7.42 (m, 6H), 7.08 – 7.02 (m, 2H), 4.75 (s, 1H). **^{13}C NMR** (101 MHz, $CDCl_3$) δ = 152.5, 145.4, 140.6, 134.1, 131.8, 130.5, 130.2, 129.3, 129.1, 128.7, 128.2, 127.7, 127.3, 127.2, 125.9, 125.2, 116.1, 112.9. **IR:** (neat) ṽ = 2253, 1739, 1615, 1501, 1439, 1345, 1208, 903, 838, 722, 649, 617, 564, 514, 463, 444, 404 cm^{-1}. **HRMS:** calcd m/z. for; $C_{20}H_{14}O^+$ $[M]^+$: 270.1045; found (EI) 270.1043.

Compound 16c

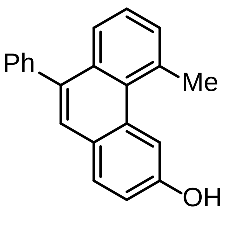

Prepared from compound **15c** (174 mg, 528 µmol, 1.0 equiv.) and BBr_3 (1 M in DCM, 1.75 mL, 1.75 mmol, 3.0 equiv.) in DCM (10 mL) according to general procedure **H**. Purification by column chromatography (SiO_2, 20% EtOAc in pentane), followed by crystallization from a dichloromethane/pentane bilayer afforded the product as green needles (145 mg, 511 µmol, 88%).
^{1}H NMR (400 MHz, $CDCl_3$) δ = 8.33 (d, *J* = 2.3 Hz, 1H), 7.81 (d, *J* = 8.5 Hz, 1H), 7.76 (dd, *J* = 8.1, 1.5 Hz, 1H), 7.59 (s, 1H), 7.53 – 7.37 (m, 8H), 7.19 (dd, *J* = 8.5, 2.4 Hz, 1H), 3.18 (s, 3H). **^{13}C NMR** (101 MHz, $CDCl_3$) δ = 153.3, 141.7, 137.2, 135.8, 133.4, 132.7, 130.9, 130.4, 130.3, 130.0, 128.4, 127.8, 127.7, 127.3, 126.0, 125.8, 116.0, 112.6, 27.8. **IR** (neat): $\tilde{\nu}$ (cm^{-1}) = 3458, 3016, 2970, 2946, 1738, 1443, 1366, 1228, 1216, 1092, 895, 527, 516. **HRMS** calc. for $C_{21}H_{15}O^-$: 283.1123; found: 283.1128 $[M-H]^-$ (ESI).

Compound 16d

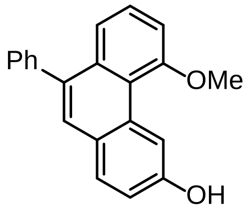

Prepared from **15d** (304 mg, 778 µmol, 1.0 equiv.), BCl_3 (1.56 mL, 1.56 mmol, 2.0 equiv.) and pentamethylbenzene (346 mg, 2.33 mmol, 3.0 equiv.) in DCM (5 mL) according to general procedure **I**. The crude compound was purified by column chromatography (SiO_2, 0 to 20% EtOAc in pentane) and prep. HPLC (4.6 x 250 mm Zorbax SB-C18 column, 3.5 µm, CH_3CN/H2O = 70/30, gradient to 90/10 over 20 min, 1.0 mL/min, 295 K, 254 nm) to afford the product as a yellow solid (85 mg, 284 µmol, 367%).
^{1}H NMR (400 MHz, $CDCl_3$) δ = 9.17 (d, J = 2.5 Hz, 1H), 7.78 (d, J = 8.5 Hz, 1H), 7.62 (s, 1H), 7.52 – 7.41 (m, 7H), 7.20 (dd, J = 8.5, 2.6 Hz, 1H), 7.14 (dd, J = 7.8, 1.3 Hz, 1H), 5.16 (s, 1H), 4.15 (s, 3H). **^{13}C NMR** (101 MHz, $CDCl_3$) δ = 159.2, 154.0, 141.7, 136.4, 134.3, 131.2, 130.3, 130.1, 128.6, 128.4, 127.3, 127.1, 126.6, 120.7, 120.0, 116.1, 113.2, 108.0, 55.9. **IR** (neat): $\tilde{\nu}$ (cm^{-1}) = 3015, 2970, 1738, 1685, 1597, 1575, 1558, 1541, 1522, 1507, 1490, 1455, 1434, 1365, 1267, 1229, 1216, 1073, 901, 704, 527, 516. **HRMS** calc. for $C_{21}H_{16}O_2Na^+$: 323.1048; found: 323.1043 $[M+Na]^+$ (ESI).

Compound 17a

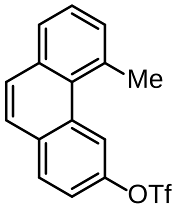

Prepared from **16a** (0.89 g, 4.28 mmol, 1.0 equiv.), triflic anhydride (1.33 g, 4.71 mmol, 1.1 equiv.) and pyridine (1.36 g, 17.1 mmol, 4.0 equiv.) in DCM (50 mL) according to general procedure **D**. Column chromatography (SiO_2, 5% EtOAc in pentane) yielded a white solid (1.15 g, 79 % yield).
^{1}H NMR (400 MHz, $CDCl_3$) δ = 8.87 (d, *J* = 2.3 Hz, 1H), 7.98 (d, *J* = 8.8 Hz, 1H), 7.87 – 7.77 (m, 2H), 7.73 (d, *J* = 8.7 Hz, 1H), 7.62 – 7.48 (m, 3H), 3.13 (s, 3H). **^{13}C NMR** (101 MHz, $CDCl_3$) δ = 147.1, 135.7, 134.0, 132.9, 132.5, 132.1, 130.6, 129.7, 129.5, 128.0, 127.2, 126.2, 120.6, 119.9, 119.0, 117.5, 77.5, 77.2, 76.8, 27.0. **^{19}F NMR** (377 MHz, $CDCl_3$) δ = -72.6. **IR:** (neat) ṽ = 3171, 3056, 2966, 2878, 2269, 1608, 1597, 1576, 1526, 1500, 1446, 1421, 1380, 1339, 1316, 1247, 1208, 1140, 1033, 1022, 965, 906, 876, 853, 838, 810, 762, 737, 709, 669, 631, 602, 573, 559, 520, 499. **HRMS:** calcd m/z. for; $C_{16}H_{11}F_3O_3S^+$ $[M]^+$: 340.0376; found (EI) 340.0375.

Compound 17b

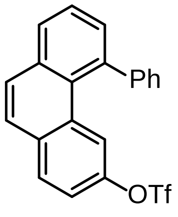

Prepared from **16b** (0.42 g, 1.55 mmol, 1.0 equiv.), triflic anhydride (0.48 g, 1.71 mmol, 1.1 equiv.) and pyridine (0.49 mg, 6.21 mmol, 4.0 equiv.) in DCM (30 mL) according to general procedure **D**. Column chromatography (SiO_2, 5% EtOAc in pentane) yielded a white solid (1.22 g, 75 % yield).
^{1}H NMR (400 MHz, $CDCl_3$) δ = 7.92 (d, *J* = 1.5 Hz, 1H), 7.87 (dd, *J* = 11.1, 8.8 Hz, 4H), 7.76 (d, *J* = 8.8 Hz, 2H), 7.66 (t, *J* = 7.6 Hz, 2H), 7.59 (d, *J* = 2.4 Hz, 2H), 7.58 – 7.46 (m, 4H), 7.45 – 7.38 (m, 3H), 7.35 (dd, *J* = 8.7, 2.5 Hz, 2H). **^{13}C NMR** (101 MHz, $CDCl_3$) δ = 146.5, 144.1, 140.9, 133.8, 132.9, 131.6, 131.4, 130.4, 129.6, 129.1, 128.9, 128.8, 128.0, 127.8, 126.8, 126.7, 120.4, 120.3, 119.5, 117.1. **^{19}F NMR** (377 MHz, $CDCl_3$) δ = -73.1. **IR:** (neat) ṽ = 3054, 2252, 1593, 1496, 1444, 1420, 1245, 1214, 1141, 1076, 1029, 903, 851, 840, 791, 725, 649, 625, 600, 563, 523, 499, 473, 433 cm^{-1}. **HRMS:** calcd m/z. for; $C_{21}H_{13}O_3F_3S^+$ $[M+Na]^+$: 425.0430; found (ESI) 425.0431.

Compound 17c

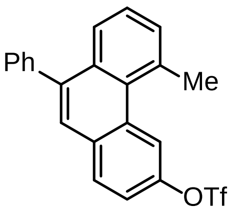

Prepared using **16c** (106 mg, 373 µmol, 1.0 equiv.), triflic anhydride (126 mg, 448 µmol, 1.2 equiv.) in DCM (10 mL) and Et_3N (3.3 mL) according to general procedure **D**. The crude compound was purified by column chromatography (SiO_2, 0 to 20% EtOAc in pentane) to afford the product as a white solid (155 mg, 372 µmol, quantitative yield).

^{1}H NMR (400 MHz, $CDCl_3$) δ = 8.86 (d, *J* = 2.2 Hz, 1H), 7.98 (d, *J* = 8.8 Hz, 1H), 7.83 – 7.79 (m, 1H), 7.67 (s, 1H), 7.58 (d, *J* = 7.1 Hz, 1H), 7.55 – 7.44 (m, 7H), 3.18 (s, 3H). **^{13}C NMR** (101 MHz, $CDCl_3$) δ = 147.0, 141.1, 140.9, 135.8, 133.3, 132.3, 132.0, 131.9, 130.6, 130.1, 130.0, 128.5, 127.8, 126.9, 126.9, 126.2, 120.0, 119.3, 119.1 (q, *J* = 320.8 MHz), 27.3. **^{19}F NMR** (377 MHz, $CDCl_3$) δ = -72.5. **IR** (neat): $\tilde{\nu}$ (cm^{-1}) = 3025, 2970, 2949, 1738, 1445, 1424, 1366, 1216, 1142, 1026, 906, 802, 703, 604, 527. **HRMS** calc. for $C_{22}H_{15}F_3O_3S^+$: 416.0694; found: 416.0692 $[M]^+$ (EI).

Compound 17d

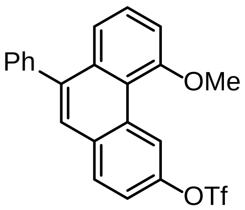

Prepared using **16d** (85 mg, 284 µmol, 1.0 equiv.), triflic anhydride (96 mg, 341 µmol, 1.2 equiv.) in DCM (10 mL) and Et_3N (3.3 mL) according to general procedure **D**. The crude compound was purified by column chromatography (SiO_2, 0 to 20% EtOAc in pentane) to afford the product as a yellow solid (117 mg, 271 µmol, 95 %).

^{1}H NMR (300 MHz, $CDCl_3$) δ = 9.73 (d, *J* = 2.5 Hz, 1H), 7.93 (d, *J* = 8.8 Hz, 1H), 7.69 (s, 1H), 7.58 – 7.42 (m, 8H), 7.25 – 7.21 (m, 1H), 4.18 (s, 3H). **^{13}C NMR** (126MHz, $CDCl_3$) δ = 159.0, 147.7, 140.9, 140.3, 134.1, 131.6, 130.6, 130.1, 128.5, 127.8, 127.7, 127.6, 121.2, 120.6, 120.2, 119.4, 119.1 (q, *J* = 320.9 Hz), 108.8, 56.0. **^{19}F NMR** (377 MHz, $CDCl_3$) δ = -72.61. **IR** (neat): $\tilde{\nu}$ (cm^{-1}) = 3006, 2970, 2943, 2358, 2338, 1738, 1575, 1495, 1454, 1421, 1365, 1216, 1141, 1072, 1019, 908, 866, 805, 761, 704, 600, 514. **HRMS** calc. for $C_{22}H_{16}F_3O_4S^+$: 433.0721; found: 433.0716 $[M+H]^+$ (ESI).

Compound 18a

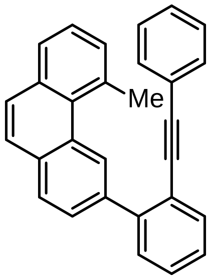

Prepared from **17a** (250 mg, 0.73 mmol, 1 equiv.), **5a** (326 mg, 1.47 mmol, 2 equiv,), $Pd_2(dba)_3$ (33.6 mg, 36.7 µmol, 5 mol%,), SPhos (30.2 mg, 73.5 µmol, 10 mol%,), Cs_2CO_3 (283 mg, 1.47 mmol, 2 equiv.,) in THF/H_2O (10/1, 20 mL) according to general procedure **B**. Column chromatography (SiO_2, 0 to 5% EtOAc in pentane), yellow solid (241 mg, 0.65 mmol 89%).

^{1}H NMR: (400 MHz, $CDCl_3$) δ = 9.28 (s, 1H), 8.01 (d, *J* = 8.2 Hz, 1H), 7.93 (dd, *J* = 8.2, 1.6 Hz, 1H), 7.86 – 7.69 (m, 4H), 7.61 (dd, *J* = 7.6, 1.3 Hz, 1H), 7.54 – 7.44 (m, 3H), 7.40 (td, *J* = 7.4, 1.3 Hz, 1H), 7.24 – 7.10 (m, 5H), 3.16 (s, 3H). **^{13}C{H} NMR:** (101 MHz, $CDCl_3$) δ = 144.4, 137.8, 135.7, 133.9, 133.3, 133.3, 132.7, 131.4, 131.3, 130.3, 129.9, 128.7, 128.3, 128.3, 128.2, 128.2, 128.0, 127.6, 127.2, 127.1, 126.8, 125.9, 123.3, 121.8, 92.4, 89.7, 27.5. **IR:** (neat) ṽ = 3079, 3047, 3018, 2959, 2876, 1595, 1568, 1490, 1466, 1441, 1428, 1400, 1391, 1376, 1301, 1259, 1237, 1215, 1162, 1152, 1138, 1092, 1070, 1023, 1002, 974, 962, 910, 891, 875, 866, 841, 810, 795, 767, 755, 716, 686, 665, 634, 590, 567, 548, 523, 515, 494, 480, 464, 446, 428, 419, 412 cm^{-1}. **HRMS:** calcd m/z. for; $C_{29}H_{20}^+$ $[M]^+$: 368.1565; found (EI) 368.1568.

Compound 18b

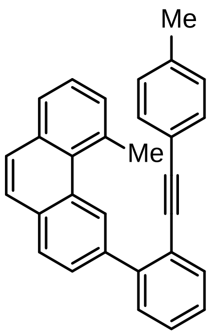

Prepared from **17a** (290 mg, 0.85 mmol, 1 equiv.), **5b** (603 mg, 2.56 mmol, 2 equiv,), $Pd_2(dba)_3$ (39.0 mg, 42.6 µmol, 5 mol%,), SPhos (34.9 mg, 85.2 µmol, 10 mol%,), Cs_2CO_3 (329 mg, 1.70 mmol, 2 equiv.,) in THF/H_2O (10/1, 20 mL) according to general procedure **B**. Column chromatography (SiO_2, 0 to 5% EtOAc in pentane), yellow solid (301 mg, 0.79 mmol, 92%).
^{1}H NMR (400 MHz, $CDCl_3$) δ = 9.28 (d, *J* = 1.6 Hz, 1H), 8.00 (d, *J* = 8.1 Hz, 1H), 7.93 (dd, *J* = 8.1, 1.5 Hz, 1H), 7.85 – 7.75 (m, 3H), 7.72 (dd, *J* = 7.8, 1.5 Hz, 1H), 7.59 (dd, *J* = 7.7, 1.4 Hz, 1H), 7.53-7.42 (m, 3H), 7.39 (td, *J* = 7.5, 1.4 Hz, 1H), 7.09 (dd, *J* = 8.0, 1.5 Hz, 2H), 6.95 (d, *J* = 7.8 Hz, 2H), 3.16 (s, 3H), 2.27 (s, 3H). **^{13}C NMR** (101 MHz, $CDCl_3$) δ = 144.4, 138.3, 138.0, 135.9, 134.0, 133.3, 132.8, 131.5, 131.5, 131.4, 130.4, 130.1, 129.1, 128.6, 128.5, 128.4, 128.4, 127.7, 127.3, 127.2, 126.9, 126.0, 122.2, 120.3, 92.8, 89.2, 27.6, 21.6. **IR:** (neat) ṽ = 3155, 3048, 3029, 2961, 2915, 2873, 2725, 2211, 1949, 1915, 1855, 1822, 1806, 1791, 1769, 1732, 1714, 1658, 1644, 1613, 1596, 1567, 1510, 1477, 1465, 1450, 1436, 1399, 1389, 1376, 1314, 1307, 1283, 1259, 1238, 1213, 1184, 1169, 1158, 1100, 1063, 1028, 1018, 964, 952, 890, 875, 867, 848, 716, 707, 696, 679, 656, 647, 634, 592, 558, 528, 514, 502, 483, 469, 445, 433, 421, 412. **HRMS:** calcd m/z. for; $C_{30}H_{22}^{+}$ $[M]^{+}$: 382.1721; found (EI) 382.1713.

Compound 18c

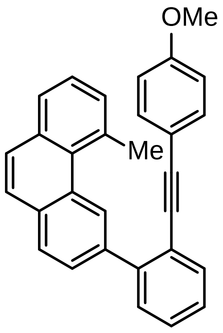

Prepared from **17a** (200 mg, 0.59 mmol, 1 equiv.), **5c** (297 mg, 1.18 mmol, 2 equiv,), $Pd_2(dba)_3$ (26.9 mg, 29.4 µmol, 5 mol%,), SPhos (24.1 mg, 58.8 µmol, 10 mol%,), Cs_2CO_3 (226 mg, 1.18 mmol, 2 equiv.,) in THF/H_2O (10/1, 15 mL) according to general procedure **B**. Column chromatography (SiO_2, 0 to 5% EtOAc in pentane), yellow solid (159 mg, 0.39 mmol, 68%).
^{1}H NMR: (400 MHz, $CDCl_3$) δ = 9.29 (s, 1H), 8.01 (d, *J* = 8.2 Hz, 1H), 7.93 (dd, *J* = 8.2, 1.6 Hz, 1H), 7.84 – 7.74 (m, 3H), 7.71 (dd, *J* = 7.8, 1.4 Hz, 1H), 7.59 (dd, *J* = 7.6, 1.1 Hz, 1H), 7.54 – 7.42 (m, 3H), 7.38 (td, *J* = 7.5, 1.5 Hz, 1H), 7.13 (d, *J* = 9.0 Hz, 2H), 6.67 (d, *J* = 8.8 Hz, 2H), 3.74 (s, 3H), 3.16 (s, 3H). **^{13}C{H} NMR:** (101 MHz, $CDCl_3$) δ = 159.6, 144.3, 138.0, 135.9, 134.0, 133.2, 133.0, 132.8, 131.5, 131.5, 130.4, 130.0, 128.5, 128.4, 128.4, 128.4, 127.7, 127.3, 127.2, 127.0, 126.0, 122.3, 115.6, 114.0, 92.7, 88.6, 55.4, 27.6. **IR:** (neat) ṽ = 3047, 3007, 2961, 2933, 2907, 2876, 2836, 2734, 2539, 2288, 2207, 2163, 2025 ,1979, 1926, 1855, 1791, 1604, 1565, 1510, 1475, 1451, 1436, 1416, 1398, 1389, 1372, 1314, 1306, 1288, 1248, 1174, 1160, 1141, 1101, 1062, 1032, 981, 961, 948, 896, 890, 874, 867, 849, 835, 820, 814, 805, 782, 754, 716, 695, 679, 652, 634, 590, 558, 545, 529, 519, 509, 481, 454, 445, 437, 430, 423, 415, 409 cm^{-1}. **HRMS:** calcd m/z. for; $C_{30}H_{22}O^{+}$ $[M]^{+}$: 398.1671; found (EI) 398.1661.

Compound 18d

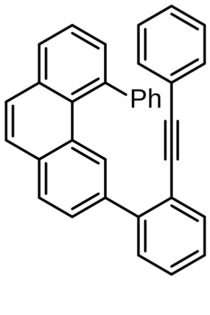

Prepared from **17b** (150 mg, 0.37 mmol, 1 equiv.), **5a** (166 mg, 0.75 mmol, 2 equiv,), $Pd_2(dba)_3$ (17.1 mg, 18.8 µmol, 5 mol%,), SPhos (15.3 mg, 37.3 µmol, 10 mol%,), Cs_2CO_3 (144 mg, 0.75 mmol, 2.0 equiv.,) in THF/H_2O (10/1, 10 mL) according to general procedure **B**. Column chromatography (SiO_2, 0 to 5% EtOAc in pentane), yellow solid (82 mg, 0.19 mmol 51 %).
^{1}H NMR: (400 MHz, $CDCl_3$) δ = 8.07 (dd, *J* = 8.2, 1.7 Hz, 1H), 7.99 (d, *J* = 1.7 Hz, 1H), 7.93 (dd, *J* = 8.0, 1.6 Hz, 2H), 7.83 (s, 2H), 7.65 – 7.56 (m, 2H), 7.53 – 7.41 (m, 7H), 7.34 – 7.20 (m, 8H), 6.91 – 6.85 (m, 1H). **^{13}C{H} NMR:** (101 MHz, $CDCl_3$) δ = 145.7, 143.7, 140.7, 137.0, 133.8, 132.9, 132.7, 131.4, 130.7, 130.4,

130.0, 129.5, 129.3, 129.2, 129.0, 128.6, 128.4, 128.3, 128.1, 127.7, 127.7, 127.3, 127.3, 127.0, 127.0, 125.8, 123.6, 121.4, 92.2, 89.8. **IR:** (neat) ṽ = 3053, 2962, 2926, 2851, 2359, 2341, 2214, 1982, 1935, 1884, 1869, 1811, 1758, 1675, 1640, 1613, 1597, 1569, 1537, 1490, 1472, 1440, 1400, 1386, 1308, 1260, 1192, 1178, 1149, 1092, 1070, 1024, 971, 952, 926, 915, 900, 876, 848, 798, 768, 757, 727, 689, 669, 647, 619, 609, 586, 568, 556, 539, 522, 513, 498, 483, 470, 457, 437 cm^{-1}. **HRMS:** calcd m/z. for; $C_{34}H_{22}^{+}$ $[M]^{+}$: 430.1721; found (EI) 430.1725.

Compound 18e

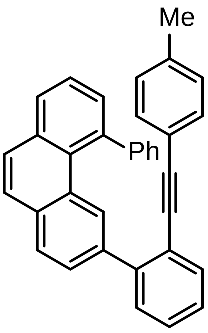

Prepared from **17b** (150 mg, 0.37 mmol, 1 equiv.), **5b** (176 mg, 0.75 mmol, 2 equiv,), $Pd_2(dba)_3$ (17.1 mg, 18.8 μmol, 5 mol%,), SPhos (15.3 mg, 37.3 μmol, 10 mol%,), Cs_2CO_3 (144 mg, 0.75 mmol, 2.0 equiv.,) in THF/H_2O (10/1, 10 mL) according to general procedure **B**. Column chromatography (SiO_2, 0 to 5% EtOAc in pentane), yellow solid (72 mg, 0.16 mmol 43 %).

^{1}H NMR: (400 MHz, $CDCl_3$) δ = 8.04 (dd, *J* = 8.2, 1.7 Hz, 1H), 7.95 (d, *J* = 1.6 Hz, 1H), 7.93 – 7.86 (m, 2H), 7.80 (s, 2H), 7.60 (t, *J* = 7.6 Hz, 1H), 7.54 (dd, *J* = 6.8, 2.3 Hz, 1H), 7.51 – 7.37 (m, 6H), 7.25 – 7.19 (m, 2H), 7.19 – 7.14 (m, 2H), 7.04 (d, *J* = 7.9 Hz, 2H), 6.87 – 6.81 (m, 1H), 2.31 (s, 3H). **^{13}C{H} NMR:** (101 MHz, $CDCl_3$) δ = 145.7, 143.6, 140.7, 138.3, 137.1, 133.8, 132.8, 132.7, 131.3, 130.7, 130.4, 130.0, 129.4, 129.3, 129.2, 129.1, 129.0, 128.6, 128.2, 127.8, 127.6, 127.3, 127.3, 127.0, 126.9, 125.7, 121.6, 120.5, 92.4, 89.1, 21.6. **IR:** (neat) ṽ = 3049, 3022, 2962, 2917, 2858, 2209, 1905, 1799, 1592, 1565, 1509, 1492, 1477, 1438, 1404, 1388, 1307, 1259, 1180, 1144, 1092, 1070, 1018, 951, 926, 904, 868, 842, 814, 795, 755, 724, 701, 680, 664, 645, 618, 607, 588, 566, 548, 528, 519, 508, 495, 482, 459, 428, 410 cm^{-1}. **HRMS:** calcd m/z. for; $C_{35}H_{24}^{+}$ $[M]^{+}$: 444.1878; found (EI) 444.1866.

Compound 18f

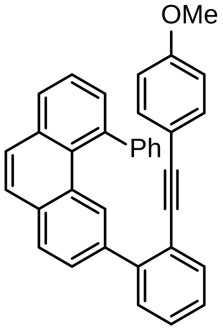

Prepared from **17b** (150 mg, 0.37 mmol, 1 equiv.), **5c** (188 mg, 0.75 mmol, 2 equiv,), $Pd_2(dba)_3$ (17.1 mg, 18.8 μmol, 5 mol%,), SPhos (15.3 mg, 37.3 μmol, 10 mol%,), Cs_2CO_3 (144 mg, 0.75 mmol, 2.0 equiv.,) in THF/H_2O (10/1, 10 mL) according to general procedure **B**. Column chromatography (SiO_2, 0 to 5% EtOAc in pentane), yellow solid (66 mg, 0.14 mmol 38 %).

^{1}H NMR: (400 MHz, $CDCl_3$) δ = 8.04 (dd, *J* = 8.2, 1.7 Hz, 1H), 7.95 (d, *J* = 1.6 Hz, 1H), 7.94 – 7.86 (m, 2H), 7.81 (s, 2H), 7.60 (t, *J* = 7.5 Hz, 1H), 7.52 (dd, *J* = 7.0, 2.0 Hz, 1H), 7.53 – 7.37 (m, 6H), 7.25 – 7.16 (m, 4H), 6.83 (dd, *J* = 7.5, 1.6 Hz, 1H), 6.76 (d, *J* = 8.8 Hz, 2H), 3.78 (s, 3H). **^{13}C{H} NMR:** (101 MHz, $CDCl_3$) δ = 159.4, 145.6, 143.3, 140.6, 137.0, 133.7, 132.7, 132.6, 132.5, 130.6, 130.3, 129.9, 129.3, 129.2, 129.1, 128.8, 128.5, 127.9, 127.6, 127.5, 127.2, 127.1, 126.9, 126.8, 125.6, 121.6, 115.6, 113.9, 92.1, 88.3, 55.3. **IR:** (neat) ṽ = 3049, 3020, 2956, 2927, 2835, 2753, 2620, 2595, 2537, 2349, 2289, 2214, 2062, 2045, 1940, 1927, 1888, 1808, 1731, 1695, 1682, 1651, 1644, 1634, 1604, 1593, 1568, 1509, 1478, 1463, 1452, 1439, 1388, 1336, 1303, 1286, 1246, 1173, 1146, 1106, 1071, 1028, 1001, 967, 952, 926, 904, 870, 842, 829, 795, 779, 724, 701, 680, 667, 643, 619, 607, 588, 566, 532, 513, 500, 481, 460, 451, 442, 427, 406 cm^{-1}. **HRMS:** calcd m/z. for; $C_{35}H_{24}O^{+}$ $[M]^{+}$: 460.1827; found (EI) 460.1829.

Compound 18g

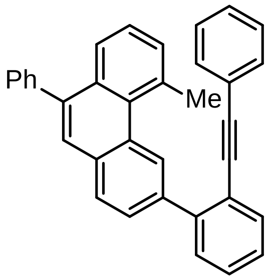

Prepared using **17c** (42 mg, 101 µmol, 1.0 equiv.), **5a** (45 mg, 203 µmol, 2.0 equiv.), Cs_2CO_3 (39 mg, 203 µmol, 2.0 equiv.), $Pd_2(dba)_3$ (4.6 mg, 5.1 µmol, 5 mol%), SPhos (4.2 mg, 10.1 µmol, 10 mol%) in THF/H_2O (10:1, 7 mL) according to general procedure **B**. The crude compound was filtered through a pad of silica, eluting with DCM and EtOAc. The crude was further purified by column chromatography (SiO_2, pentane) and prep. HPLC to afford the product as a yellow solid (30 mg, 68 µmol, 67% yield).

^{1}H NMR (500 MHz, $CDCl_3$) δ = 9.25 (t, *J* = 1.1 Hz, 1H), 8.02 – 7.94 (m, 2H), 7.80 (dd, *J* = 8.2, 1.5 Hz, 1H), 7.75 (ddd, *J* = 7.7, 1.5, 0.5 Hz, 1H), 7.72 (s, 1H), 7.61 (ddd, *J* = 7.7, 1.4, 0.5 Hz, 1H), 7.58 – 7.51 (m, 4H), 7.51 – 7.44 (m, 3H), 7.45 – 7.38 (m, 2H), 7.25 – 7.19 (m, 3H), 7.19 – 7.14 (m, 2H), 3.20 (s, 3H). **^{13}C NMR** (126 MHz, $CDCl_3$) = δ = 144.5, 141.6, 139.7, 137.8, 135.9, 133.5, 133.1, 132.2, 131.5, 131.5, 131.4, 131.0, 130.9, 130.3, 130.1, 129.0, 128.8, 128.5, 128.4, 128.3, 128.2, 127.8, 127.7, 127.4, 127.2, 125.8, 123.4, 122.0, 92.6, 89.8, 27.8. **IR** (neat): $\tilde{\nu}$ (cm^{-1}) = 3055, 3023, 2970, 1738, 1598, 1492, 1441, 1366, 1228, 1217, 1069, 1029, 906, 808, 756, 731, 702, 690, 528. **HRMS** calc. for $C_{35}H_{24}^+$: 444.1878; found: 444.1873 $[M]^+$ (EI). **Prep. HPLC** conducted on an Interchim Puriflash 4250, 4.6 x 250 mm Zorbax SB-C18 column, 3.5 µm, CH_3CN/H2O = 95/5, 30 min isocratic, 1.0 mL/min, 295 K, 254 nm.

Compound 18h

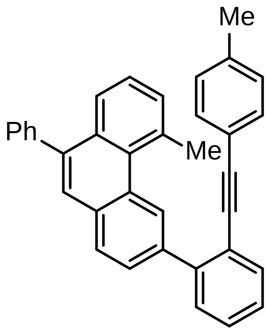

Prepared using 1**7c** (80 mg, 192 µmol, 1.0 equiv.), **5b** (91 mg, 384 µmol, 2.0 equiv.), Cs_2CO_3 (74 mg, 384 µmol, 2.0 equiv.), $Pd_2(dba)_3$ (8.8 mg, 9.6 µmol, 5 mol%), SPhos (7.9 mg, 19.2 µmol, 10 mol%) in THF/H_2O (10:1, 24 mL) according to general procedure **B**. The crude compound was filtered through a pad of silica, eluting with DCM. The crude was further purified by column chromatography (SiO_2, 0 to 1% EtOAc in pentane) and HPLC to afford the product as a yellow solid (66 mg, 143 µmol, 75% yield).

^{1}H NMR (400 MHz, $CDCl_3$) δ = 9.29 (s, 1H), 8.10 – 7.92 (m, 2H), 7.91 – 7.72 (m, 3H), 7.64 (dd, *J* = 7.7, 1.3 Hz, 1H), 7.61 – 7.40 (m, 9H), 7.17 (d, *J* = 8.0 Hz, 2H), 7.01 (d, *J* = 7.9 Hz, 2H), 3.23 (s, 3H), 2.31 (s, 3H). **^{13}C NMR** (101 MHz, $CDCl_3$) δ = 144.4, 141.7, 139.7, 138.4, 137.9, 135.9, 133.4, 133.1, 132.2, 131.4, 131.4, 131.0, 130.9, 130.3, 130.1, 129.1, 128.6, 128.5, 128.4, 127.8, 127.7, 127.4, 127.2, 125.8, 125.8, 122.2, 120.4, 100.5, 92.8, 89.2, 27.8x, 21.6. **IR** (neat): $\tilde{\nu}$ (cm^{-1}) = 3055, 3025, 2970, 2919, 1738, 1594, 1510, 1491, 1475, 1440, 1405, 1377, 1228, 1217, 1103, 1036, 1022, 906, 817, 775, 764, 731, 702, 688, 649, 635, 534, 515. **HRMS** calc. for $C_{36}H_{27}^+$: 459.2113; found: 459.2107 $[M+H]^+$ (ESI). **Prep. HPLC** conducted on an Interchim Puriflash 4250, 4.6 x 250 mm Zorbax SB-C18 column, 3.5 µm, CH_3CN/H2O = 90/10, gradient to 100/0 over 10 min, 1 mL/min, 295 K, 254 nm.

Compound 18i

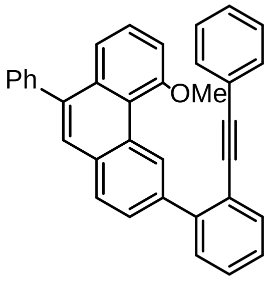

Prepared using **17d** (57 mg, 131 µmol, 1.0 equiv.), **5a** (58 mg, 261 µmol, 2.0 equiv.), Cs_2CO_3 (50 mg, 261 µmol, 2.0 equiv.), $Pd_2(dba)_3$ (6.0 mg, 6.5 µmol, 5 mol%), SPhos (5.4 mg, 13.1 µmol, 10 mol%,) in THF/H_2O (10:1, 20 mL) according to general procedure **B**. The crude compound was filtered through a pad of silica, eluting with DCM. The crude mixture was further purified by column chromatography (SiO_2, 0 to 1% EtOAc in pentane) and HPLC to afford the product as a green oil (37 mg, 81 µmol, 62% yield).

^{1}H NMR (400 MHz, $CDCl_3$) δ = 10.20 (s, 1H), 7.95 (q, *J* = 8.1 Hz, 2H), 7.75 (t, *J* = 3.9 Hz, 2H), 7.65 (d, *J* = 7.7 Hz, 1H), 7.59 – 7.43 (m, 9H), 7.39 (t, *J* = 7.5

Hz, 1H), 7.28 (s, 1H), 7.23 – 7.08 (m, 4H), 3.93 (s, 3H). **^{13}C NMR** (101 MHz, $CDCl_3$) δ = 159.1, 144.9, 141.7, 139.0, 138.4, 134.1, 133.4, 131.6, 131.6, 130.3, 130.2, 129.8, 129.7, 128.7, 128.4, 128.2, 128.2, 128.0, 128.0, 127.7, 127.4, 127.0, 126.5, 123.7, 122.0, 121.5, 120.0, 108.2, 92.5, 89.9, 55.6. **IR** (neat): $\tilde{\nu}$ (cm^{-1}) = 3465, 3016, 2970, 2360, 2339, 1738, 1602, 1568, 1491, 1454, 1434, 1365, 1263, 1229, 1217, 1077, 908, 812, 757, 729, 703, 690, 539, 527, 514. **HRMS** calc. for $C_{35}H_{25}O^+$: 461.1905; found: 461.1900 $[M+H]^+$ (ESI). **Prep. HPLC** conducted on an Interchim Puriflash 4250, 4.6 x 250 mm Zorbax SB-C18 column, 3.5 µm, CH_3CN/H2O = 95/5, 20 min isocratic, 1.0 mL/min, 10.4 MPa, 295 K, 254 nm.

Compound 18j

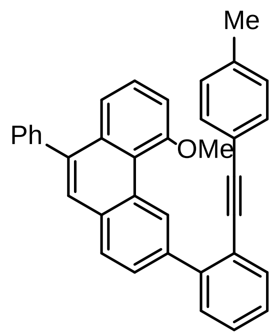

Prepared using **17d** (60 mg, 138 µmol, 1.0 equiv.), **5b** (65 mg, 276 µmol, 2.0 equiv.), Cs_2CO_3 (53 mg, 276 µmol, 2.0 equiv.), $Pd_2(dba)_3$ (6.3 mg, 6.9 µmol, 5 mol%), SPhos (5.7 mg, 13.8 µmol, 10 mol%) in THF/H_2O (10:1, 7 mL) according to general procedure **B**. The crude compound was filtered through a pad of silica, eluting with DCM and EtOAc. The crude mixture was further purified by column chromatography (SiO_2, 0 to 1% EtOAc in pentane) and HPLC to afford the product as a yellow oil (53 mg, 111 µmol, 81% yield).
^{1}H NMR (400 MHz, $CDCl_3$) δ = 10.21 (d, J = 1.6 Hz, 1H), 7.99 – 7.91 (m, 2H), 7.78 – 7.61 (m, 3H), 7.60 – 7.43 (m, 8H), 7.39 (td, J = 7.6, 1.4 Hz, 1H), 7.20 – 7.14 (m, 2H), 7.12 (dd, J = 7.9, 1.2 Hz, 1H), 6.96 (d, J = 7.9 Hz, 2H), 3.95 (s, 3H), 2.28 (s, 3H). **^{13}C NMR** (101 MHz, $CDCl_3$) δ = 159.2, 144.7, 141.7, 138.9, 138.5, 138.1, 134.1, 133.3, 131.5, 131.5, 130.3, 130.2, 129.8, 129.7, 129.0, 128.7, 128.5, 128.4, 128.0, 127.7, 127.4, 127.0, 126.4, 122.2, 121.5, 120.6, 119.9, 108.2, 92.7, 89.3, 77.2, 55.7, 21.6. **IR** (neat): $\tilde{\nu}$ (cm^{-1}) = 3057, 3023, 2971, 2932, 1738, 1605, 1566, 1510, 1491, 1476, 1454, 1434, 1397, 1383, 1264, 1235, 1217, 1077, 978, 907, 813, 765, 730, 703, 526, 507. **HRMS** calc. for $C_{36}H_{27}O^+$: 475.2062; found: 475.2050 $[M+H]^+$ (ESI). **Prep. HPLC** conducted on an Interchim Puriflash 4250, 4.6 x 250 mm Zorbax SB-C18 column, 3.5 µm, CH_3CN/H2O = 95/5, 30 min isocratic, 1.0 mL/min, 295 K, 254 nm.

Synthesis of Tetrahelicenes

General Procedure J for the enantioselective cycloisomerisation

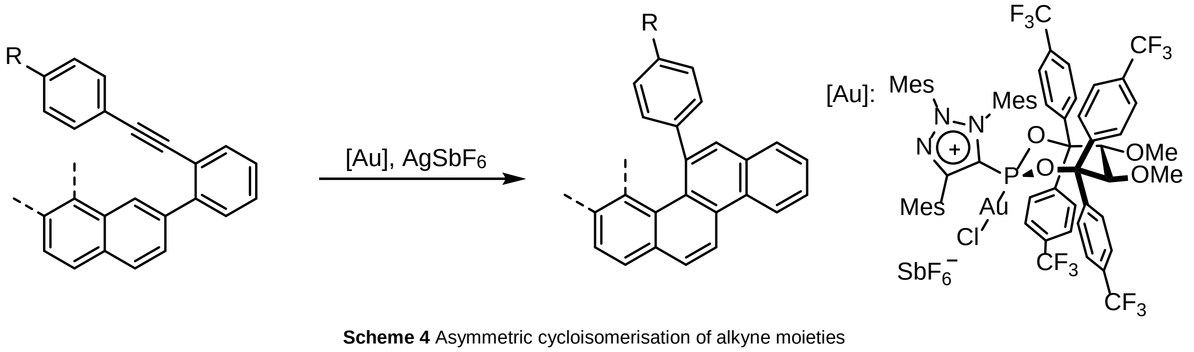

Scheme 4 Asymmetric cycloisomerisation of alkyne moieties

Adapted from a literature procedure.[4] A dried Schlenk equipped with a magnetic stirring bar flask was loaded with the respective alkyne (25 µmol, 1.0 equiv.) and the gold precatalyst **2g** (5 mol% per alkyne moiety). After drying *in vacuo* for 1 h, DCM (0.05 M) was added. To the stirred mixture $AgSbF_6$ (5 mol% per alkyne moiety, 0.05 M in DCM) was added dropwise. After stirring for 48 h at -20 °C the mixture was filtered through a silica plug eluting with DCM. Evaporation of the solvent yielded a crude product mixture which was purified by preparative HPLC. Product ratios are determined by NMR and HPLC, enantiomeric excesses by chiral HPLC.

Compound 1a

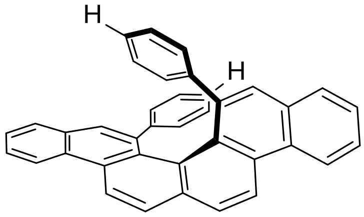

Prepared from **3a** (12 mg, 25 µmol, 1.0 equiv.), **2g** (4.1 mg, 2.5 µmol, 10 mol%) and $AgSbF_6$ (0.86 mg, 10 mol% 0.05 M in DCM) in DCM (0.5 mL) according to general procedure **J**. The crude mixture could be obtained as a yellow solid (11.4 mg, 95%), **1a:9a**: 96:4, >95% conversion (determined by NMR), +98% enantiomeric excess.

In order to obtain a purer sample for characterization, the mixture was purified by semi-preparative HPLC, obtaining 7.8 mg of **1a** (65% yield). Separation conditions: $MeCN/H_2O$ = 90/10 to 100/0 over 20min, 1.0 ml min^{-1}, 295 K; helicene tR= 15.557 min, isomer tR= 28.037 min. The *ee* was determined by chiral HPLC: $MeCN/H_2O$ = 75/25, 45 min, 1.0 ml min^{-1}, 298 K, 210 nm; major enantiomer tR= 10.330 min, minor enantiomer tR= 15.255 min.

^{1}H NMR: (300 MHz, $CDCl_3$) δ = 8.79 (d, *J* = 8.6 Hz, 2H), 8.70 (d, *J* = 8.3 Hz, 2H), 8.13 (d, *J* = 8.5 Hz, 2H), 7.71 (dd, *J* = 8.0, 1.4 Hz, 2H), 7.63 (ddd, *J* = 8.3, 6.9, 1.5 Hz, 2H), 7.52 (t, *J* = 7.2 Hz, 2H), 7.31 (s, 2H), 6.69 (d, *J* = 5.6 Hz, 10H). **^{13}C{H} NMR:** (101 MHz, $CDCl_3$) δ = 140.9, 137.8, 132.2, 131.1, 129.8, 129.1, 128.9, 128.5, 128.3, 128.2, 126.9, 126.8, 126.4, 126.3, 126.0, 126.0, 122.9, 122.0. **IR:** (neat) ṽ = 3081, 3060, 3027, 2250, 2199, 2150, 2036, 1987, 1939, 1595, 1492, 1474, 1443, 1343, 1264, 1239, 1210, 1177, 1162, 1073, 1034, 905, 851, 829, 779, 729, 698, 667, 649, 616, 579, 557, 536, 523, 504, 472, 438, 415, 405 cm^{-1}. **HRMS:** calcd m/z. for; $C_{38}H_{24}^+$ $[M]^+$: 481.1878 ; found (EI) 480.1873. $[\alpha]_{24}^{D}$: (98% *ee*) +954.71 (c = 0.39, DCM).

Compound 1b

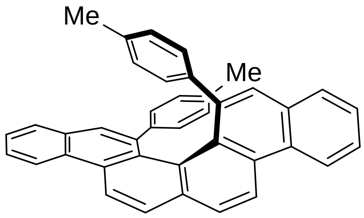

Prepared from **3b** (12.7 mg, 25 µmol, 1.0 equiv.), **2g** (4.1 mg, 2.5 µmol, 10 mol%) and $AgSbF_6$ (0.86 mg, 10 mol% 0.05 M in DCM) in DCM (0.5 mL) according to general procedure **J**. The crude mixture could be obtained as a yellow solid (12.0 mg, 94%), **1b:9b**: 97:3, >95% conversion. +97% enantiomeric excess.

In order to obtain a purer sample for characterization, the mixture was purified by semi-preparative HPLC, obtaining 9.2 mg of **1b** (72% yield). Separation conditions: $MeCN/H_2O$ = 90/10 to 100/0 over 20min, 1.0 ml min^{-1}, 295 K; helicene tR= 22.201 min. The *ee* was determined by chiral HPLC: $MeCN/H_2O$ = 75/25, 45 min, 1.0 ml min^{-1}, 298 K, 210 nm; major enantiomer tR= 16.207 min, minor enantiomer tR= 20.046 min.

^{1}H NMR: (400 MHz, $CDCl_3$) δ = 8.76 (d, *J* = 8.5 Hz, 2H), 8.69 (d, *J* = 8.3 Hz, 2H), 8.10 (d, *J* = 8.5 Hz, 2H), 7.71 (dd, *J* = 8.0, 1.3 Hz, 2H), 7.62 (ddd, *J* = 8.3, 6.9, 1.4 Hz, 2H), 7.52 (ddd, *J* = 8.0, 6.9, 1.1 Hz, 2H), 7.29 (s, 2H), 6.67 (s, 4H), 6.50 (s, 4H), 1.90 (s, 6H). **^{13}C{H} NMR:** (101 MHz, $CDCl_3$) δ = 137.7, 137.3, 135.5, 132.2, 130.8, 129.6, 128.9, 128.8, 128.5, 127.8, 127.3, 127.2, 126.6, 126.1, 125.9, 125.8, 122.8, 121.7, 20.5. **IR:** (neat) ṽ = 3051, 3024, 2915, 2862, 2364, 2247, 2209, 2183, 2170, 2153, 2135, 2114, 2046, 2033, 2007, 1969, 1945, 1910, 1737, 1613, 1589, 1563, 1510, 1475, 1440, 1373, 1343, 1306, 1271, 1242, 1207, 1186, 1159, 1143, 1114, 1039, 1017, 943, 930, 908, 886, 862, 823, 813, 793, 769, 747, 734, 669, 649, 606, 584, 568, 542, 529, 521, 488, 470, 446, 420 cm^{-1}. **HRMS:** calcd m/z. for; $C_{40}H_{28}^+$ $[M]^+$: 508.2191 ; found (EI) 508.2176. $[\alpha]_{24}^{D}$: (97% *ee*) +860.71 (c = 0.51, DCM).

Compound 1c

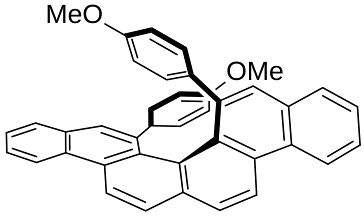

Prepared from **3c** (13.5 mg, 25 µmol, 1.0 equiv.), **2g** (4.1 mg, 2.5 µmol, 10 mol%) and $AgSbF_6$ (0.86 mg, 10 mol% 0.05 M in DCM) in DCM (0.5 mL) according to general procedure **J.** The crude mixture could be obtained as a yellow solid (11.1 mg, 84%), **1c:9c**: 93:7, >95% conversion. +88 % enantiomeric excess.

In order to obtain a purer sample for characterization, the mixture was purified by semi-preparative HPLC, obtaining 10.2 mg of **1c** (76% yield).
Separation conditions: MeCN/H_2O = 90/10 to 100/0 over 20min, 1.0 ml min^{-1}, 295 K; helicene tR= 13.858 min, isomer tR= 23.206 min. The *ee* was determined by chiral HPLC: MeCN/H_2O = 75/25, 45 min, 1.0 ml min^{-1}, 298 K, 210 nm; major enantiomer tR= 12.857 min, minor enantiomer tR= 14.657 min.
^{1}H NMR: (400 MHz, $CDCl_3$) δ = 8.78 (d, *J* = 8.6 Hz, 2H), 8.70 (d, *J* = 8.3 Hz, 2H), 8.12 (d, *J* = 8.5 Hz, 2H), 7.74 (dd, *J* = 7.9, 1.4 Hz, 2H), 7.63 (ddd, *J* = 8.4, 6.9, 1.5 Hz, 2H), 7.54 (ddd, *J* = 8.0, 6.9, 1.2 Hz, 2H), 7.29 (d, *J* = 6.4 Hz, 2H), 6.72 (s,4H), 6.25 (s, 4H), 3.32 (s, 6H). **^{13}C{H} NMR:** (101 MHz, $CDCl_3$) δ = 157.8, 136.9, 133.1, 132.2, 130.8, 130.1, 129.6, 128.9, 128.5, 127.8, 126.6, 126.5, 126.2, 125.8, 125.8, 122.8, 121.7, 112.3, 110.0, 55.1. **IR:** (neat) ṽ = 3059, 3038, 3004, 2950, 2931, 2907, 2833, 2357, 2340, 2322, 2250, 2156, 2049, 2036, 2025, 1974, 1961, 1606, 1574, 1512, 1475, 1461, 1439, 1341, 1304, 1282, 1245, 1207, 1177, 1112, 1034, 948, 930, 908, 889, 860, 827, 785, 769, 750, 733, 669, 646, 608, 587, 570, 552, 507, 474, 461, 442, 420 cm^{-1}. **HRMS:** calcd m/z. for; $C_{40}H_{28}O_2^+$ $[M]^+$: 540.2089 ; found (EI) 540.2076. $[\boldsymbol{\alpha}]_{\boldsymbol{24}}^{\boldsymbol{D}}$: (88% *ee*) +860.71 (c = 0.51, DCM).

Compound 1d

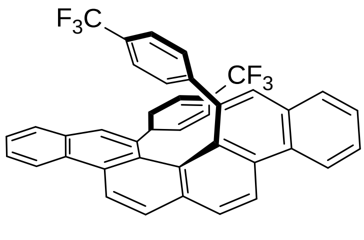

Prepared from **3d** (15.4 mg, 25 µmol, 1.0 equiv.), **2g** (4.1 mg, 2.5 µmol, 10 mol%) and $AgSbF_6$ (0.86 mg, 10 mol% 0.05 M in DCM) in DCM (0.5 mL) according to general procedure **J**. The crude mixture could be obtained as a yellow solid (15.2 mg, 24.7 µmol, 95%), **1d:9d**: 37:63, >95% conversion. +95 % enantiomeric excess. In addition a mono cyclized product could be detected (70%, ratio determined by NMR).
Separation conditions: MeCN/H_2O = 90/10 to 100/0 over 20min, 1.0 ml min^{-1}, 295 K; helicene tR= 18.9 min, mono-cyclisation tR= 24.5 min, isomer tR= 29.1 min. The *ee* was determined by chiral HPLC: MeCN/H_2O = 75/25, 45 min, 1.0 ml min^{-1}, 298 K, 210 nm; major enantiomer tR= 9.4 min, minor enantiomer tR= 13.6 min.
^{1}H NMR: (400 MHz, $CDCl_3$) δ = 8.81 (d, *J* = 8.6 Hz, 2H), 8.70 (d, *J* = 8.4 Hz, 2H), 8.17 (d, *J* = 8.7 Hz, 2H), 7.76 – 7.63 (m, 5H), 7.61 – 7.37 (m, 4H), 7.33 (d, *J* = 5.0 Hz, 3H), 6.98 (d, *J* = 17.0 Hz, 4H). **^{19}F NMR**: (377 MHz, $CDCl_3$) δ -62.9. **IR:** (neat) ṽ = 2958, 2893, 2357, 1772, 1723, 1613, 1460, 1440, 1408, 1374, 1323, 1280, 1240, 1165, 1123, 1109, 1065, 1037, 1014, 991, 956, 930, 844, 800, 760, 724, 713, 677 ,617 , 491, 472, 437, 418, 410. **HRMS** calc. for $C_{40}H_{22}F_6^+$: 616.1620; found: 616.1618 $[M]^+$ (EI). Additional analytics impossible to small isolated yield of **1d**.

Compound 1e

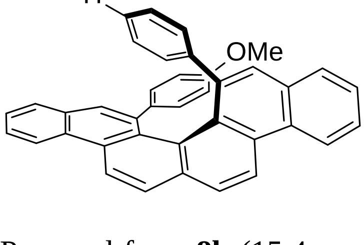

Prepared using **8a** (1.0 equiv., 12.8 mg, 25.1 µmol), gold pre-catalyst **2g** (5 mol%, 2.1 mg, 1.3 µmol), DCM (0.5 mL) and $AgSbF_6$ (5 mol%, 431 µg, 1.3 µmol, 0.05 M in DCM) according to general procedure **F**. The mixture was stirred for 48 h at -20 °C. The crude mixture was purified by HPLC to afford the product as a green oil (7.3 mg, 14.3 µmol, 57% yield) **1e:9e** 98:2, +88% enantiomeric excess.

Prepared from **8b** (15.4 mg, 25 µmol, 1.0 equiv**2g** (4.1 mg, 2.5 µmol, 10 mol%) and $AgSbF_6$ (0.86 mg, 10 mol% 0.05 M in DCM) in DCM (0.5 mL) according to general procedure **F.** In order to obtain a purer sample for characterization, the mixture was purified by semi-preparative HPLC, obtaining 8.4 mg of **1e** (65% yield), **1e:9e**: 94:6, >95% conversion. +97 % enantiomeric excess.
Separation conditions: MeCN/H_2O = 80/20 for 5min, than gradient to 100/0 over 10min, 1.0 ml min^{-1}, 295 K; helicene tR= 18.757 min, isomer tR= 27.379 min. The *ee* was determined by chiral HPLC:

MeCN/H_2O = 70/30, 45 min, 1.0 ml min^{-1}, 298 K, 210 nm; major enantiomer tR= 16.631 min, minor enantiomer tR= 18.995 min.
^{1}H NMR (500 MHz, $CDCl_3$) δ = 8.77 (ddd, *J* = 10.0, 8.5, 0.7 Hz, 2H), 8.69 (ddd, *J* = 8.4, 1.3, 0.6 Hz, 2H), 8.11 (d, *J* = 8.5 Hz, 2H), 7.71 (dddt, *J* = 22.0, 7.9, 1.3, 0.6 Hz, 2H), 7.62 (dddd, *J* = 10.5, 8.3, 6.9, 1.4 Hz, 2H), 7.52 (dddd, *J* = 9.0, 7.9, 6.9, 1.1 Hz, 2H), 7.29 (d, *J* = 5.1 Hz, 2H), 6.77 – 6.64 (m, 3H), 6.25 (bs, 2H), 3.29 (s, 3H). **^{13}C NMR** (126 MHz, $CDCl_3$) δ = 157.9, 140.7, 137.5, 137.2, 133.4, 132.3, 132.3, 131.0, 130.2, 129.8, 129.7, 129.2, 128.9, 128.9, 128.7, 128.4, 128.1, 128.1, 127.7, 127.3, 126.8, 126.7, 126.4, 126.3, 126.2, 126.1, 126.0, 125.9, 122.9, 122.9, 122.0, 121.8, 112.4, 55.2. 157.9, 140.7, 137.5, 137.2, 133.4, 132.3, 132.3, 131.0, 130.2, 129.8, 129.7, 129.2, 128.9, 128.9, 128.7, 128.4, 128.1, 128.1, 127.7, 127.3, 126.8, 126.7, 126.4, 126.3, 126.2, 126.1, 126.0, 125.9, 122.9, 122.9, 122.0, 121.8, 55.2. **IR** (ATR): $\tilde{\nu}$ (cm^{-1}) = 3029, 2970, 2953, 1738, 1606, 1509, 1440, 1366, 1229, 1217, 1034, 907, 829, 776, 751, 703, 528. **Specific rotation** $[\alpha]_{589}^{25}$:(88% *ee*) +717.98° (in DCM).

Compound 20a

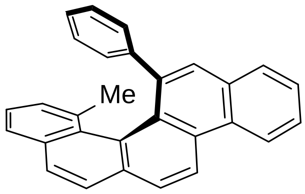

Prepared from **18a** (9.2 mg, 25 µmol, 1.0 equiv), **2g** (2.1 mg, 1.25 µmol, 5 mol%) and $AgSbF_6$ (0.43 mg, 5 mol% 0.05 M in DCM) in DCM (0.5 mL) according to general procedure **J**. The crude mixture could be obtained as a yellow solid (8.6 mg, 23.4 mg, 93%) **20a:21a**: 63:37, 95% conversion. +60% enantiomeric excess.

In order to obtain a purer sample for characterization, the mixture was purified by semi-preparative HPLC, obtaining 2.3 mg of **20a** (6.3 µmol, 25%). Separation conditions: MeCN/H_2O = 90/10, 30min, 1.0 ml min^{-1}, 295 K; helicene tR= 15.8 min. The *ee* was determined by chiral HPLC: MeCN/H_2O = 65/35, 40 min, 1.0 ml min^{-1}, 298 K; major enantiomer tR= 14.6 min, minor enantiomer tR= 16.2 min. The crude sample was furthermore analysed by chiral SFC: IC-3 SFC 3.0x100mm, 3µm; CO_2/MeOH = 90/10, 12 min, 1.7 ml min^{-1}, 310 K; major enantiomer tR= 3.1 min, minor enantiomer tR= 3.5 min.
^{1}H NMR: (400 MHz, $CDCl_3$) δ = 8.88 (d, *J* = 8.5 Hz, 1H), 8.84 (d, *J* = 8.4 Hz, 1H), 8.10 (dd, *J* = 8.4, 1.6 Hz, 1H), 8.03 (d, *J* = 7.8 Hz, 1H), 7.81 (d, *J* = 23.0 Hz, 3H), 7.74 (tt, *J* = 6.9, 1.4 Hz, 1H), 7.67 (ddd, *J* = 8.1, 6.9, 1.4 Hz, 1H), 7.56 (d, *J* = 7.8 Hz, 1H), 7.26 (s, 1H), 7.14 (t, *J* = 7.5 Hz, 1H), 7.10-6.55 (s, 2H), 6.91 – 6.84 (m, 2H), 6.75 (d, *J* = 7.2 Hz, 1H), 2.05 (s, 3H). **^{13}C{H} NMR:** (101 MHz, $CDCl_3$) δ = 141.9, 140.1, 135.8, 132.4, 132.1, 132.1, 132.1, 130.1, 129.9, 129.2, 128.6, 128.3, 128.0, 127.8, 126.8, 126.8, 126.3, 126.2, 126.0, 125.9, 124.9, 124.0, 123.4, 121.8, 23.8. **IR:** (neat) ṽ = 3047, 2927, 2851, 2221, 2206, 2193, 2179, 2151, 2103, 2094, 2063, 2020, 1992, 1965, 1847, 1681, 1491, 1444, 1339, 1318, 1274, 1178, 1157, 1077, 1033, 913, 883, 830, 805, 746, 722, 699, 669, 651, 623, 603, 581, 534, 509, 493, 477, 469, 455, 445, 433, 418. **HRMS** calc. for $C_{29}H_{20}^+$: 368.1560; found: 368.1561 $[M]^+$ (EI). $[\boldsymbol{a}]_{\boldsymbol{24}}^{\boldsymbol{D}}$: (60% *ee*) +349.62 (c = 0.12, DCM).

Compound 20b

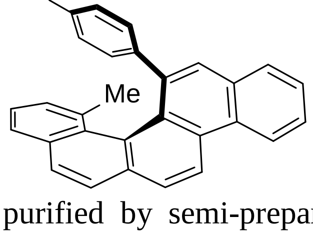

Prepared from **18b** (9.6 mg, 25 µmol, 1.0 equiv), **2g** (2.1 mg, 1.25 µmol, 5 mol%) and $AgSbF_6$ (0.43 mg, 5 mol% 0.05 M in DCM) in DCM (0.5 mL) according to general procedure **J**. . The crude mixture could be obtained as a yellow solid (4.1 mg, 10.7 µmol, 43%) **20b:21b**: 42:58, 52% conversion. +75% enantiomeric excess.

In order to obtain a purer sample for characterization, the mixture was purified by semi-preparative HPLC, obtaining 4.0 mg of **20a** (10.3 µmol, 41% yield). Separation conditions: MeCN/H_2O = 90/10, 45 min, 1.0 ml min^{-1}, 295 K; helicene tR= 20.8 min. The *ee* was determined by chiral HPLC: MeCN/H_2O = 65/35, 40 min, 1.0 ml min^{-1}, 298 K; major enantiomer tR= 17.5 min, minor enantiomer tR= 19.74 min. The crude sample was furthermore analyzed by chiral SFC:

IA-3 SFC 3.0x100mm, 3µm; CO_2/*i*PrOH = 75/25, 15 min, 1.7 ml min^{-1}, 310 K; major enantiomer tR= 2.0 min, minor enantiomer tR= 3.6 min.
^{1}H NMR: (400 MHz, $CDCl_3$) δ = 8.87 (dd, *J* = 8.6, 1.6 Hz, 1H), 8.83 (d, *J* = 8.3 Hz, 1H), 8.09 (dd, *J* = 8.5, 1.6 Hz, 1H), 8.02 (d, *J* = 7.75 Hz, 1H), 7.80 (d, *J* = 19.8, 3H), 7.76 – 7.69 (m, 1H), 7.69 – 7.63 (m, 1H), 7.56 (d, *J* = 7.8 Hz, 1H), 7.26 (s, 2H), 7.14 (td, *J* = 7.5, 1.6 Hz, 1H), 6.73 (d, *J* = 7.2 Hz, 1H), 6.64 (s, 2H), 2.13 (s, *J* = 1.6 Hz, 3H), 2.04 (s, *J* = 1.6 Hz, 3H). **^{13}C{H} NMR:** (101 MHz, $CDCl_3$) δ = 140.1, 139.0, 135.8, 135.6, 132.4, 132.2, 132.0, 132.0, 130.0, 129.9, 128.8, 128.6, 128.5, 128.2, 128.0, 128.0, 127.9, 126.7, 126.6, 126.3, 126.2, 125.6, 124.9, 124.0, 123.4, 121.7, 23.7, 20.9. **IR:** (neat) ṽ = 3039, 2961, 2924, 2853, 2359, 2016, 1738, 1663, 1613, 1592, 1509, 1486, 1466,1444, 1377, 1343, 1303, 1261, 1233, 1215, 1205, 1183, 1162, 1144, 1114, 1077, 1031, 946, 913, 885, 830, 820, 809, 745, 715, 683, 670, 644, 630, 602, 558, 533, 520, 506, 486, 471, 460, 422, 406. **HRMS** calc. for $C_{30}H_{22}^+$: 382.1716; found: 382.1714 $[M]^+$ (EI). $[\alpha]_{24}^{D}$: (75% *ee*) +209.50 (c = 0.21, DCM).

Compound 20c

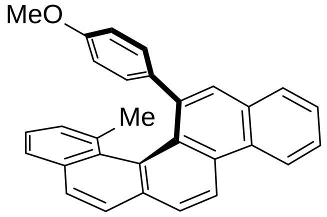

Prepared from **18c** (10.0 mg, 25 µmol, 1.0 equiv), **2g** (2.1 mg, 1.25 µmol, 5 mol%) and $AgSbF_6$ (0.43 mg, 5 mol% 0.05 M in DCM) in DCM (0.5 mL) according to general procedure **J**. The crude mixture could be obtained as a yellow solid (8.5 mg, 21.3 µmol, 85%) **20c:21c**: 44:56, 87% conversion. +70% enantiomeric excess.
In order to obtain a purer sample for characterization, the mixture was purified by semi-preparative HPLC, obtaining 4.6 mg of **20a** (11.5 µmol, 46% yield). Separation conditions: MeCN/H_2O = 90/10, 45 min, 1.0 ml min^{-1}, 295 K; helicene tR= 13.3 min. The *ee* was determined by chiral HPLC: MeCN/H_2O = 65/35, 40 min, 1.0 ml min^{-1}, 298 K; major enantiomer tR= 15.3 min, minor enantiomer tR= 16.8 min. The crude sample was furthermore analyzed by chiral SFC: IC-3 SFC 3.0x100mm, 3µm; CO_2/MeOH = 90/10, 10 min, 1.7 ml min^{-1}, 310 K; major enantiomer tR= 4.7 min, minor enantiomer tR= 5.3 min.
1H NMR: (400 MHz, CDCl3) δ = 8.87 (d, J = 8.5 Hz, 1H), 8.83 (d, J = 8.2 Hz, 1H), 8.08 (d, J = 8.4 Hz, 1H), 8.01 (d, J = 7.8 Hz, 1H), 7.79 (d, J = 10.86 Hz, 3H), 7.72 (t, J = 7.57 Hz, 1H), 7.66 (t, J = 7.67 Hz, 1H), 7.56 (d, J = 7.8 Hz, 1H), 7.17 (t, J = 7.5 Hz, 1H), 6.76 (d, J = 7.2 Hz, 1H), 3.66 (d, J = 0.8 Hz, 3H), 2.03 (s, 3H). 13C{H} NMR: (101 MHz, CDCl3) δ = 157.8, 139.9, 139.5, 135.7, 134.4, 132.1, 132.1, 131.9, 131.9, 129.9, 129.7, 128.3, 128.3, 128.3, 128.0, 127.9, 127.7, 126.6, 126.4, 126.1, 126.0, 125.7, 124.8, 123.9, 123.3, 121.6, 55.3, 23.6 IR: (neat) ṽ = 3035, 2996, 2959, 2925, 2853, 2833, 2360, 1983, 1663, 1606, 1572, 1508, 1464, 1439, 1376, 1341, 1320, 1302, 1280, 1244, 1205, 1177, 1141, 1092, 1033, 947, 907, 886, 863, 829, 805, 740, 687, 669, 647, 626, 602, 580, 561, 547, 536, 525, 512, 486, 476, 466, 454, 422, 412. HRMS calc. for C30H22O+: 398.1665; found: 398.1663 [M]+ (EI). $[\alpha]_{24}^{D}$: (70% ee) +192.48 (c = 0.23, DCM).

Compound 20d

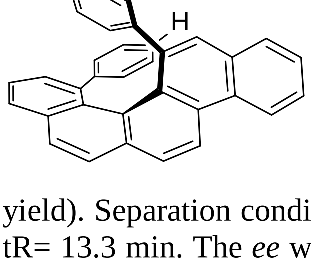

Prepared from **3d** (10.8 mg, 25 µmol, 1.0 equiv**2g** (4.1 mg, 2.5 µmol, 10 mol%) and $AgSbF_6$ (0.86 mg, 10 mol% 0.05 M in DCM) in DCM (0.5 mL) according to general procedure **X**. The crude mixture could be obtained as a yellow solid (10.1 mg, 23.4 µmol 93%) **20d:21d**: 69:31, 95% conversion. +97% enantiomeric excess.
In order to obtain a purer sample for characterization, the mixture was purified by semi-preparative HPLC, obtaining 3 mg of **20a** (7 µmol, 28% yield). Separation conditions: MeCN/H_2O = 90/10 to 100/0 over 10 min, 1.0 ml min^{-1}, 295 K; helicene tR= 13.3 min. The *ee* was determined by chiral HPLC: MeCN/H_2O = 65/35, 40 min, 1.0 ml min^{-1}, 298 K; major enantiomer tR= 16.5 min, minor enantiomer tR= 18.6 min.

^{1}H NMR: (400 MHz, $CDCl_3$) δ = 8.77 (d, *J* = 8.5 Hz, 1H), 8.68 (d, *J* = 8.3 Hz, 1H), 8.05 (d, *J* = 8.5 Hz, 1H), 7.86 (d, *J* = 0.9 Hz, 2H), 7.68 (dd, *J* = 7.9, 1.3 Hz, 2H), 7.68 – 7.56 (m, 1H), 7.57 – 7.45 (m, 1H), 7.29 (d, *J* = 7.5 Hz, 2H), 7.24 (s, 2H), 7.06 (d, *J* = 1.4 Hz, 1H), 6.89 (s, 4H), 6.70 (s, 4H). **^{13}C NMR** (126 MHz, $CDCl_3$) δ = 140.9, 140.9, 140.1, 137.2, 133.2, 132.1, 131.5, 129.8, 129.4, 129.1, 129.1, 128.8, 128.7, 128.4, 128.4, 128.2, 127.7, 126.9, 126.4, 126.4, 126.3, 126.2, 126.0, 126.0, 125.6, 122.9, 122.0. **IR:** (neat) ṽ = 3074, 3052, 2961, 2923, 2852, 2365, 2342, 2223, 2176, 2158, 2119, 2060, 2030, 2000, 1952, 1889, 1658, 1631, 1598, 1572, 1493, 1464, 1446, 1411, 1305, 1260, 1222, 1210, 1178, 1157, 1077, 1020, 913, 881, 832, 800, 766, 749, 733, 722, 698, 671, 623, 609, 586, 559, 529, 508, 497, 485, 472, 447, 427, 418, 406. **HRMS** calc. for $C_{34}H_{22}^+$: 430.1716; found: 430.1714 $[M]^+$ (EI). $[\boldsymbol{a}]_{\boldsymbol{24}}^{\boldsymbol{D}}$: (97% *ee*) +1025.75 (c = 0.15, DCM).

Compound 20e

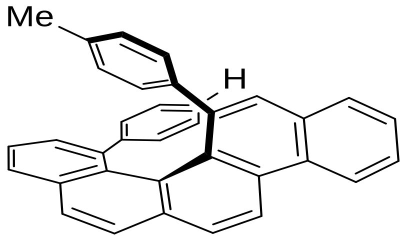

Prepared from **3d** (11.1 mg, 25 µmol, 1.0 equiv**2g** (4.1 mg, 2.5 µmol, 10 mol%) and $AgSbF_6$ (0.86 mg, 10 mol% 0.05 M in DCM) in DCM (0.5 mL) according to general procedure **X**. The crude mixture could be obtained as a yellow solid (10.4 mg, 23 µmol, 93%) **20e:21e** 54:46, >95% conversion. +99% enantiomeric excess.
In order to obtain an analytically purer sample for characterization, the mixture was purified by semi-preparative HPLC, obtaining 6.4 mg of **20a** (14.4 µmol, 57% yield). Separation conditions: MeCN/H_2O = 90/10 to 100/0 over 10 min, 1.0 ml min^{-1}, 295 K; helicene tR= 12.4 min. The *ee* was determined by chiral HPLC: MeCN/H_2O = 65/35, 40 min, 1.0 ml min^{-1}, 298 K; major enantiomer tR= 20.6 min, minor enantiomer tR= 22.9 min. The crude sample was furthermore analyzed by chiral SFC: IC-3 SFC 3.0x100mm, 3µm; CO_2/*i*PrOH = 85/15, 8 min, 1.7 ml min^{-1}, 310 K; major enantiomer tR= 4.5 min, minor enantiomer tR= 5.1 min.
^{1}H NMR: (400 MHz, $CDCl_3$) δ = 8.76 (d, *J* = 8.4 Hz, 1H), 8.67 (d, *J* = 8.3 Hz, 1H), 8.04 (d, *J* = 8.4 Hz, 1H), 7.86 (s, 2H), 7.72 – 7.64 (m, 2H), 7.64 – 7.57 (m, 1H), 7.50 (t, *J* = 7.0 Hz, 1H), 7.29 (m, 1H), 7.22 (s, 1H), 7.04 (dd, *J* = 7.3, 1.3 Hz, 1H), 6.68 (s, 8H), 2.16 (s, 3H). **^{13}C{H} NMR:** (101 MHz, $CDCl_3$) δ = 141.0, 140.0, 138.0, 137.2, 135.6, 133.1, 132.2, 131.4, 129.7, 129.4, 129.1, 129.0, 128.7, 128.6, 128.5, 128.1, 128.0, 127.6, 127.6, 126.9, 126.5, 126.3, 126.2, 126.0, 126.0, 125.9, 125.9, 125.6, 122.8, 122.0, 21.0. **IR:** (neat) ṽ = 3051, 2957, 2922, 2853, 2359, 2251, 2212, 2170, 2137, 2085, 2034, 2004, 1949, 1733, 1666, 1602, 1509, 1464, 1444 ,1364, 1307, 1261, 1222, 1114, 1018, 973, 941, 910, 832, 819, 796, 750, 736, 699,, 667, 648, 628, 614, 605, 584, 559, 526, 506, 495, 480, 469, 449, 440, 427, 416. **HRMS** calc. for $C_{35}H_{24}^+$: 444.1873; found: 444.1872 $[M]^+$ (EI). $[\boldsymbol{a}]_{\boldsymbol{24}}^{\boldsymbol{D}}$: (99% *ee*) +591.62 (c = 0.21, DCM).

Compound 20f

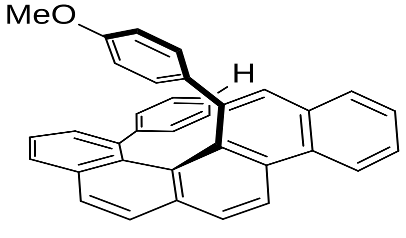

Prepared from **18f** (11.5 mg, 25 µmol, 1.0 equiv), **2g** (2.1 mg, 1.25 µmol, 5 mol%) and $AgSbF_6$ (0.43 mg, 5 mol% 0.05 M in DCM) in DCM (0.5 mL) according to general procedure **X**. The crude mixture could be obtained as a yellow solid (7.5 mg, 16.3 µmol, 65%) **20f:21f** 38:62, 66% conversion. +98% enantiomeric excess.
In order to obtain an analytically purer sample for characterization, the mixture was purified by semi-preparative HPLC, obtaining 3.1 mg of **20a** (6.75 µmol, 27% yield). Separation conditions: MeCN/H_2O = 90/10 to 100/0 over 20 min, 1.0 ml min^{-1}, 295 K; helicene tR= 11.2 min. The *ee* was determined by chiral HPLC: MeCN/H_2O = 60/40, 40 min, 1.0 ml min^{-1}, 298 K; major enantiomer tR= 28.7 min, minor enantiomer tR= 30.8 min.
^{1}H NMR (400 MHz, $CDCl_3$) δ = 8.76 (d, *J* = 8.6 Hz, 1H), 8.66 (d, *J* = 8.3 Hz, 1H), 8.03 (d, *J* = 8.5 Hz, 1H), 7.86 (d, *J* = 1.4 Hz, 2H), 7.67 (ddd, *J* = 8.7, 7.7, 1.4 Hz, 2H), 7.60 (ddd, *J* = 8.3, 6.9, 1.4 Hz, 1H), 7.53 – 7.47 (m, 1H), 7.30 (t, *J* = 7.6 Hz, 1H), 7.18 (s, 1H), 7.06 (dd, *J* = 7.3, 1.3 Hz, 1H), 6.68 (s, 3H),

6.43 (s, 2H), 3.68 (d, *J* = 1.3 Hz, 3H). **^{13}C NMR** (101 MHz, $CDCl_3$) δ = 140.8, 139.9, 136.6, 133.4, 133.0, 132.1, 131.3, 129.8, 129.6, 129.1, 128.9, 128.8, 128.5, 128.4, 127.9, 127.7, 127.4, 127.4, 126.8, 126.3, 126.2, 126.2, 125.9, 125.8, 125.7, 125.5, 124.9, 122.7, 121.8, 112.3, 55. **IR:** (neat) ṽ = 3050, 3001, 2959, 2925, 2853, 2362, 1898, 1661, 1633, 1605, 1574, 1508, 1464, 1441, 1415, 1341, 1305, 1261, 1246, 1205, 1177, 1141, 1092, 1032, 919, 883, 830, 803, 750, 739, 702, 671, 626, 608, 585, 556, 533, 519, 489, 470, 449, 421. **HRMS** calc. for $C_{35}H_{24}O^+$: 461.1900; found: 461.1888 $[M+H]^+$ (ESI). $[\boldsymbol{\alpha}]^{\boldsymbol{D}}_{\mathbf{24}}$: (98% *ee*) +1042.75 (c = 0.08, DCM).

Compound 20g

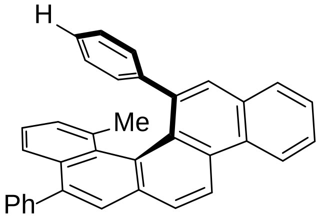

Prepared using **18g** (1.0 equiv., 11.6 mg, 25.3 µmol), gold pre-catalyst **2g** (5 mol%, 2.1 mg, 1.3 µmol), DCM (0.5 mL) and $AgSbF_6$ (5 mol%, 435 µg, 1.3 µmol, 0.05 M in DCM) according to general procedure **F**. The product could not be isolated (37% NMR yield), **20g:21g:** 90:10, +67%.enantiomeric excess.
Separation conditions: MeCN/ H_2O = 85/15, 60 min, 1.0 ml min^{-1}, 295 K; helicene tR= 31.2 min. The *ee* was determined by chiral HPLC: MeCN/H_2O = 70/30, 45 min, 0.8 ml min^{-1}, 303 K; major enantiomer tR= 15.4 min, minor enantiomer tR= 19.9 min.

Compound 20h

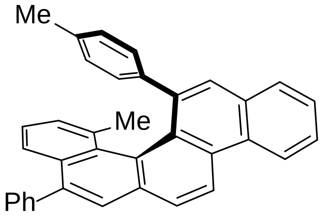

Prepared using **18h** (1.0 equiv., 11.1 mg, 25 µmol), gold pre-catalyst **2g** (5 mol%, 2.1 mg, 1.3 µmol), DCM (0.5 mL) and $AgSbF_6$ (5 mol%, 429 µg, 1.3 µmol, 0.05 M in DCM) according to general procedure **F**. The crude mixture could be obtained as a yellow oil **20h:21h**: 98:2, >95% conversion. +79% enantiomeric excess.
In order to obtain a purer sample for characterization, the mixture was purified by semi-preparative HPLC, obtaining 10.7 mg of **1b** (23.6 µmol, 93% yield). Separation conditions: MeCN/H_2O = 90/10 to 100/0 over 20min, 1.0 ml min^{-1}, 295 K; helicene tR= 21.7 min. The *ee* was determined by chiral HPLC: MeCN/H_2O = 68/32, 45 min, 0.8 ml min^{-1}, 303 K; major enantiomer tR= 27.4 min, minor enantiomer tR= 42.3 min.
^{1}H NMR (500 MHz, $CDCl_3$) δ = 8.89 (dd, *J* = 8.4, 0.7 Hz, 1H), 8.84 (ddd, *J* = 8.3, 1.3, 0.7 Hz, 1H), 8.09 (d, *J* = 8.5 Hz, 1H), 8.03 (ddt, *J* = 7.7, 1.2, 0.5 Hz, 1H), 7.84 (d, *J* = 0.7 Hz, 1H), 7.76 – 7.70 (m, 2H), 7.67 (ddd, *J* = 8.0, 6.9, 1.1 Hz, 1H), 7.64 – 7.59 (m, 2H), 7.58 – 7.45 (m, 5H), 7.18 – 6.94 (m, 2H), 6.74 (dt, *J* = 7.0, 1.0 Hz, 3H), 2.15 (s, 3H), 2.05 (d, *J* = 0.7 Hz, 3H). **^{13}C NMR** (126 MHz, $CDCl_3$) δ = 140.9, 140.0, 139.5, 139.0, 135.8, 135.7, 132.8, 132.2, 131.4, 130.9, 130.3, 130.2, 129.9, 128.9, 128.5, 128.4, 128.4, 128.1, 128.0, 127.4, 126.7, 126.6, 126.1, 125.7, 125.5, 125.5, 123.4, 122.3, 122.1, 116.5, 24.1, 21.0. **IR** (ATR): $\tilde{\nu}$ (cm^{-1}) = 3058, 3024, 2969, 2923, 2864, 2246, 1738, 1598, 1510, 1492, 1442, 1374, 1260, 1228, 1216, 1206, 1093, 1030, 906, 887, 866, 820, 810, 788, 771, 727, 701, 675, 660, 648, 601, 587, 533. **HR-MS** calc. for $C_{36}H_{27}^+$: 459.2113; found: 459.2107 $[M+H]^+$ (ESI-HRMS). $[\boldsymbol{\alpha}]^{\mathbf{24}}_{\mathbf{589}}$: (92% *ee*) 235.27° (in DCM).

Compound 20i

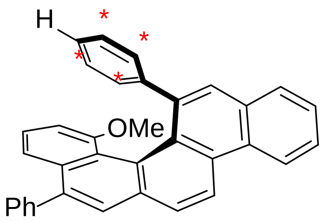

Prepared using **18i** (1.0 equiv., 11.6 mg, 25.2 µmol), gold pre-catalyst **2g** (5 mol%, 2.1 mg, 1.3 µmol), DCM (0.5 mL) and $AgSbF_6$ (5 mol%, 432 µg, 1.3 µmol, 0.05 M in DCM) according to general procedure **F**.
The crude mixture could be obtained as a yellow oil **20i:21i**: 99:1, >95% conversion. +92% enantiomeric excess.
In order to obtain a purer sample for characterization, the mixture was purified by semi-preparative HPLC, obtaining 10.7 mg of **1b** (23.2 µmol, 92% yield). The red designated C and H atoms are not visible in NMR. Separation conditions: MeCN/H_2O = 90/10 to 100/0 over 20min, 1.0 ml min^{-1}, 295 K; helicene tR= 13.9 min. The *ee* was determined by chiral HPLC: MeCN/H_2O = 70/30, 45 min, 1.0 ml min^{-1}, 295 K; major enantiomer tR= 11.28 min, minor enantiomer tR= 15.8 min.
^{1}H NMR (500 MHz, $CDCl_3$) δ = 8.89 – 8.86 (m, 1H), 8.82 (ddd, *J* = 8.2, 1.3, 0.6 Hz, 1H), 8.06 – 8.03 (m, 1H), 8.01 (ddt, *J* = 7.7, 1.5, 0.6 Hz, 1H), 7.79 (s, 1H), 7.77 (d, *J* = 0.6 Hz, 1H), 7.71 (ddd, *J* = 8.3, 6.9, 1.5 Hz, 1H), 7.66 (ddd, *J* = 8.0, 6.9, 1.2 Hz, 1H), 7.64 – 7.60 (m, 2H), 7.57 – 7.51 (m, 2H), 7.49 – 7.43 (m, 1H), 7.33 (dd, *J* = 8.2, 1.0 Hz, 1H), 7.10 (dd, *J* = 7.8 Hz, 1H), 6.93 (tt, *J* = 7.4, 1.2 Hz, 1H), 6.27 (dd, *J* = 8.0, 1.0 Hz, 1H), 3.52 (s, 3H). **^{13}C NMR** (126 MHz, $CDCl_3$) δ = 155.3, 142.6, 141.2, 140.9, 139.0, 132.2, 131.7, 131.2, 130.1, 130.0, 129.9, 128.4, 128.4, 128.3, 127.4, 126.9, 126.7, 126.6, 126.5, 126.3, 126.0, 125.9, 123.9, 123.5, 123.3, 122.1, 117.3, 105.0, 54.7. **IR** (ATR): $\tilde{\nu}$ (cm^{-1}) = 3457, 3026, 2970, 2831, 2360, 2244, 1738, 1597, 1560, 1526, 1492, 1448, 1425, 1395, 1365, 1259, 1230, 1217, 1089, 1033, 1021, 905, 886, 819, 788, 764, 723, 700, 674, 661, 649, 602, 587, 571, 538, 510. **HR-MS** calc. for $C_{35}H_{25}O^+$: 461.1905; found: 461.1900 $[M+H]^+$ (ESI-HRMS). $[\alpha]^{23}_{589}$: (92% *ee*) 433.55° (in DCM).

Compound 20j

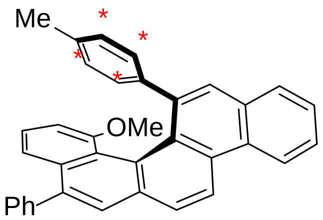

Prepared using **18j** (1.0 equiv., 11.9 mg, 25.1 µmol), gold pre-catalyst **2g** (5 mol%, 2.1 mg, 1.3 µmol), DCM (0.5 mL) and $AgSbF_6$ (5 mol%, 431 µg, 1.3 µmol, 0.05 M in DCM) according to general procedure **F**. The crude mixture could be obtained as a yellow solid **20j:21j**: 95:5, >95% conversion. +90% enantiomeric excess.
In order to obtain a purer sample for characterization, the mixture was purified by semi-preparative HPLC, obtaining 5.1 mg of **1b** (43% yield). The red designated C and H atoms are not visible in NMR. Separation conditions: MeCN/H_2O = 90/10 to 100/0 over 20min, 1.0 ml min^{-1}, 295 K; helicene tR= 16.267 min. The *ee* was determined by chiral HPLC: MeCN/H_2O = 70/30, 45 min, 1.0 ml min^{-1}, 303 K, 210 nm; major enantiomer tR= 13.57 min, minor enantiomer tR= 23.09 min.
^{1}H NMR (500 MHz, $CDCl_3$) δ = 8.88 – 8.84 (m, 1H), 8.81 (dd, *J* = 8.3, 1.1 Hz, 1H), 8.03 (d, *J* = 8.5 Hz, 1H), 8.00 (dd, *J* = 7.8, 1.5 Hz, 1H), 7.77 (s, 1H), 7.74 (s, 1H), 7.69 (ddd, *J* = 8.3, 6.9, 1.5 Hz, 1H), 7.66 – 7.60 (m, 3H), 7.57 – 7.51 (m, 2H), 7.49 – 7.43 (m, 1H), 7.33 (dd, *J* = 8.2, 1.0 Hz, 1H), 7.09 (t, *J* = 8.0 Hz, 1H), 6.26 (dd, *J* = 7.9, 1.0 Hz, 1H), 3.51 (s, 3H), 2.16 (s, 3H). **^{13}C NMR** (126 MHz, $CDCl_3$) δ = 155.3, 141.2, 141.0, 139.7, 138.9, 135.5, 132.3, 131.6, 131.1, 130.1, 129.9, 129.8, 128.5, 128.4, 128.3, 127.4, 126.7, 126.6, 126.5, 126.3, 126.1, 126.0, 123.9, 123.4, 123.3, 122.1, 117.3, 105.0, 54.6, 21.0. **IR** (ATR): $\tilde{\nu}$ (cm^{-1}) = 3024, 2969, 1738, 1597, 1563, 1525, 1510, 1492, 1446, 1425, 1396, 1364, 1260, 1230, 1216, 1089, 1026, 961, 907, 887, 866, 812, 786, 771, 727, 701, 675, 661, 647, 588, 528, 511. **HR-MS** calc. for $C_{36}H_{27}O^+$: 475.2062; found: 475.2056 $[M+H]^+$ (ESI-HRMS). $[\alpha]^{23}_{589}$: (90% *ee*) 424.53° (in DCM).

Photophysical properties of helicenes

Circular dichroism spectra were measured on a Jasco J-1500 spectrometer using a 10 mm quartz sample cell. UV/Vis spectra were conducted using either a Jasco V-630 or Jasco J-1500 spectrometer using a 1 cm quartz sample cell. Fluorescence spectra were conducted using a Jasco FP-8500 spectrofluorometer using a 10x2 mm quartz sample cell. Concentrations are stated in each case.

Circular Dichroism

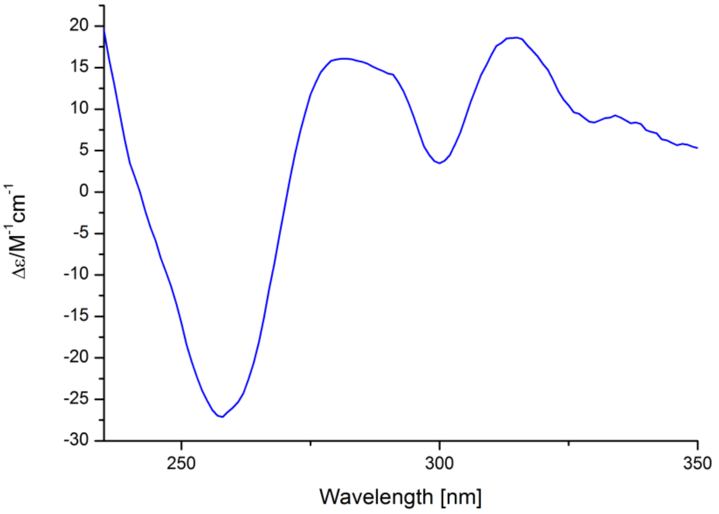

Figure S1. Circular dichroism spectrum of **1a** (98% *ee*), 10µM DCM

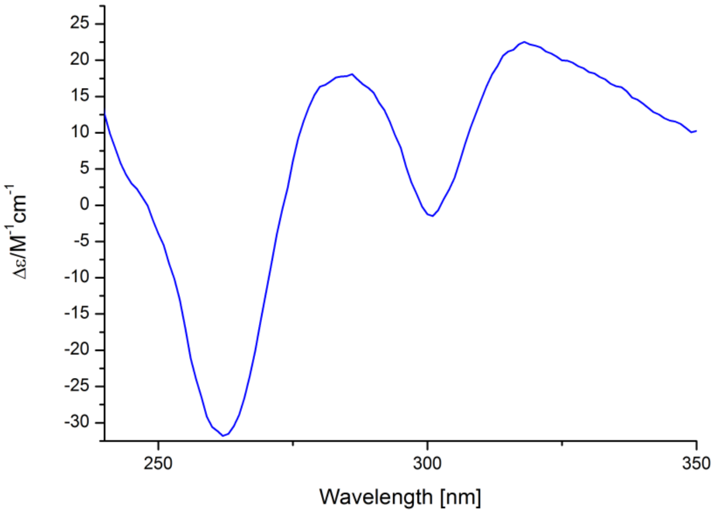

Figure S2. Circular dichroism spectrum of **1b** (97% *ee*), 10µM DCM

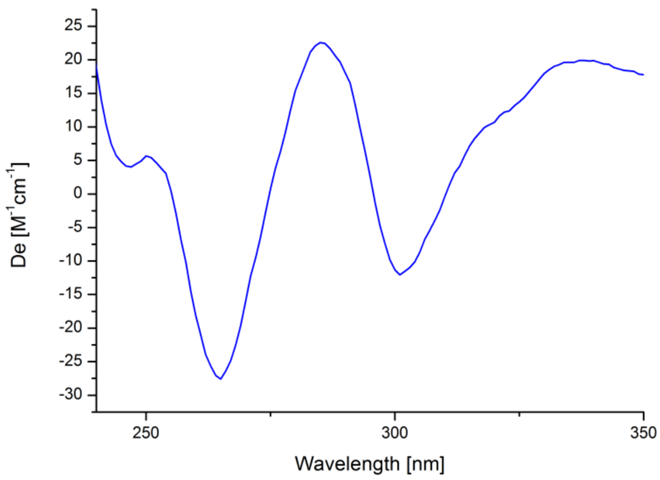

Figure S3. Circular dichroism spectrum of **1c** (88% *ee*), 10µM DCM

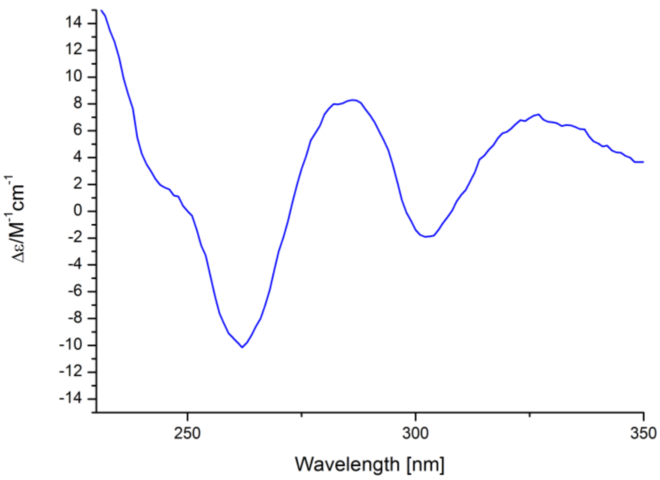

Figure S4. Circular dichroism spectrum of **1e** from **8a** (88% *ee*), 10µM DCM

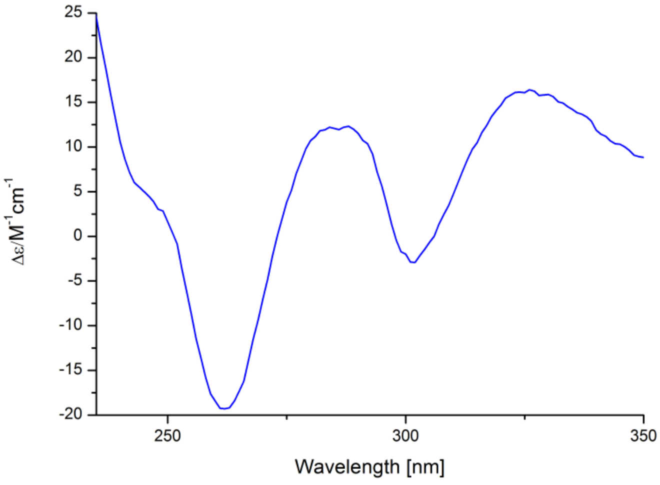

Figure S5. Circular dichroism spectrum of **1e** from **8b** (97% *ee*), 10µM DCM

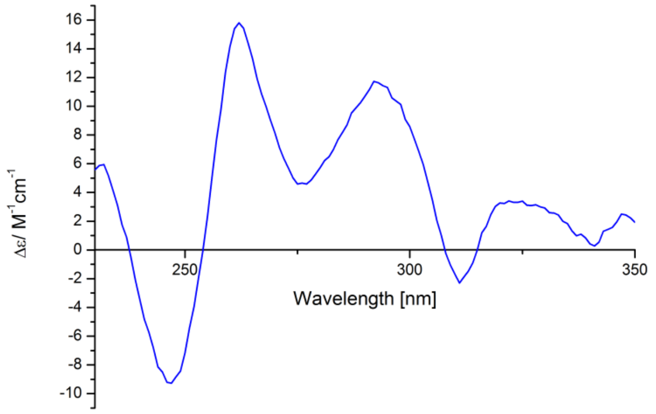

Figure S6. Circular dichroism spectrum of **20b** (75% *ee*), 10μM DCM

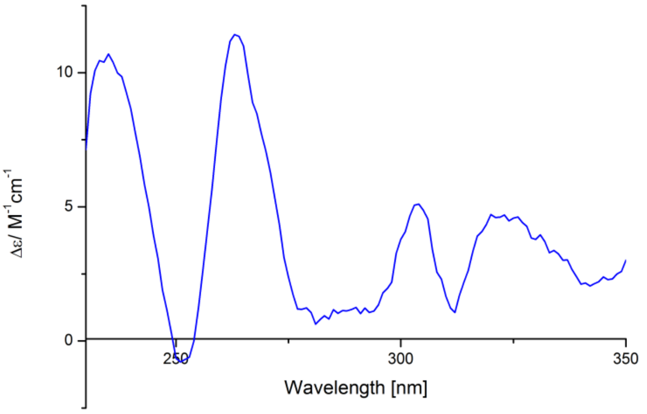

Figure S7. Circular dichroism spectrum of **20c** (70% *ee*), 10μM DCM

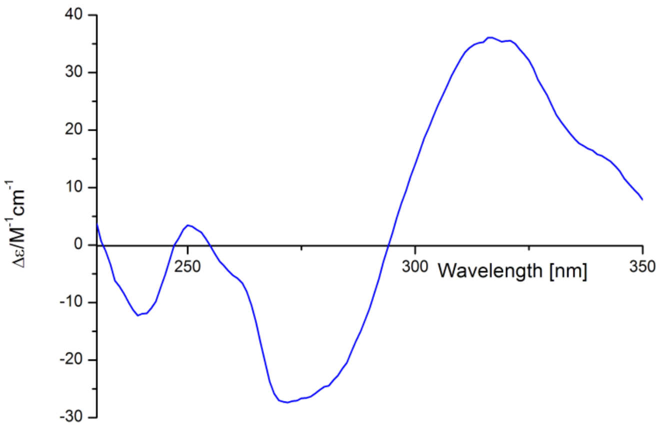

Figure S8. Circular dichroism spectrum of **20d** (97% *ee*), 10μM DCM

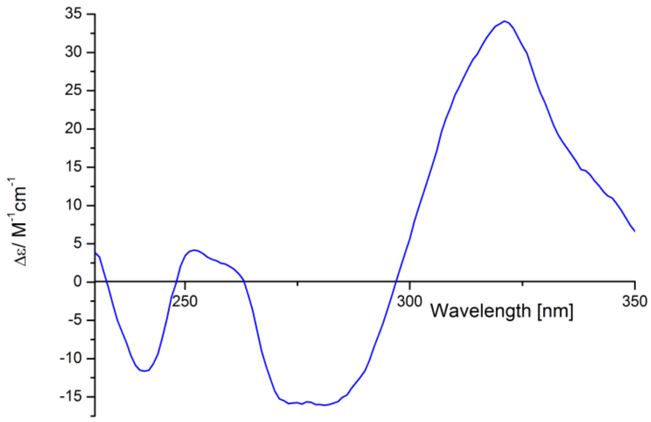

Figure S9. Circular dichroism spectrum of **20e** (99% *ee*), 10μM DCM

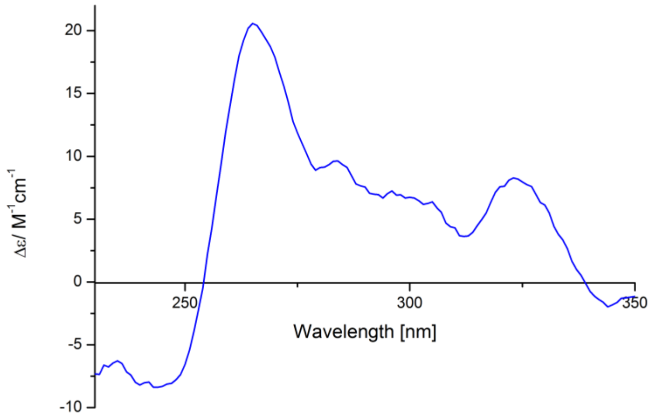

Figure S10. Circular dichroism spectrum of **20h** (92% *ee*), 10μM DCM

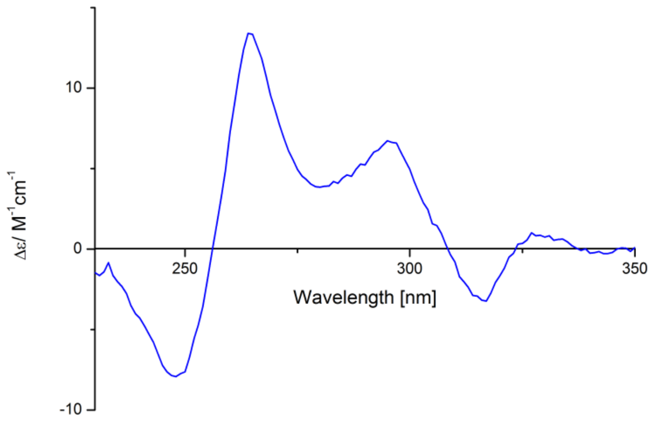

Figure S11. Circular dichroism spectrum of **20j** (90% *ee*), 10μM DCM

Absorption and Fluorescence

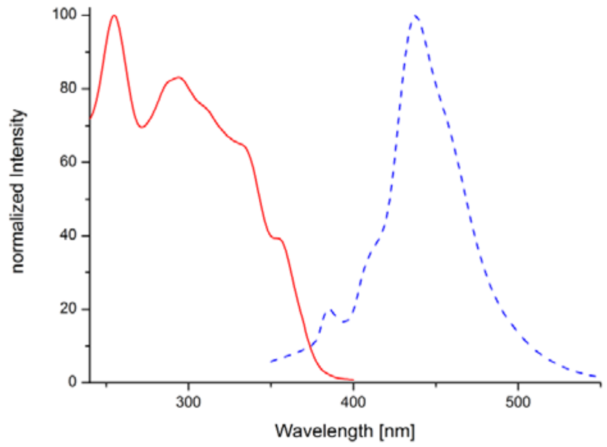

Figure S12. Normalized absorbtion (red, 10 µM, DCM) and fluorescence (blue, 0.1 µM, DCM) spectra of **1a.**

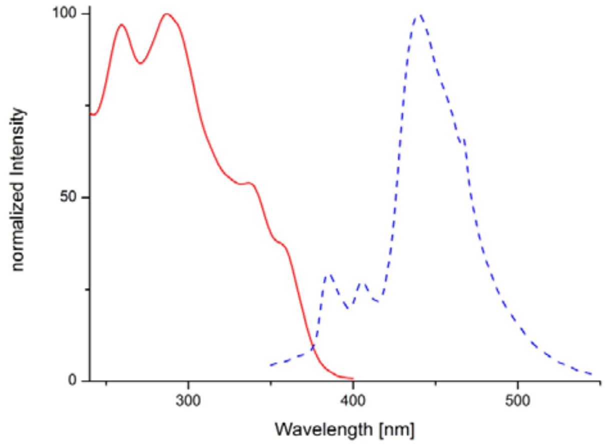

Figure S13. Normalized absorbtion (red, 10 µM, DCM) and fluorescence (blue, 0.1 µM, DCM) spectra of **1b.**

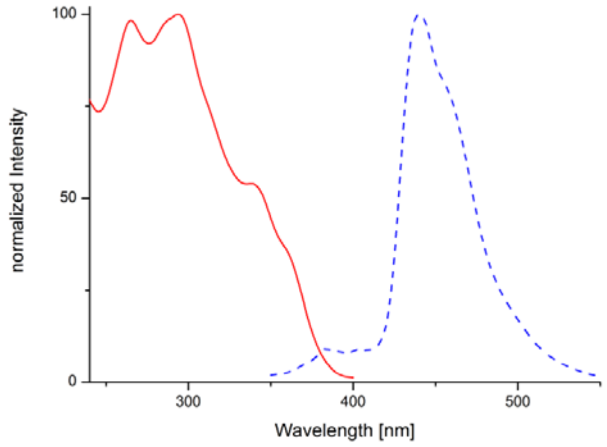

Figure S14. Normalized absorbtion (red, 10 µM, DCM) and fluorescence (blue, 0.1 µM, DCM) spectra of **1c.**

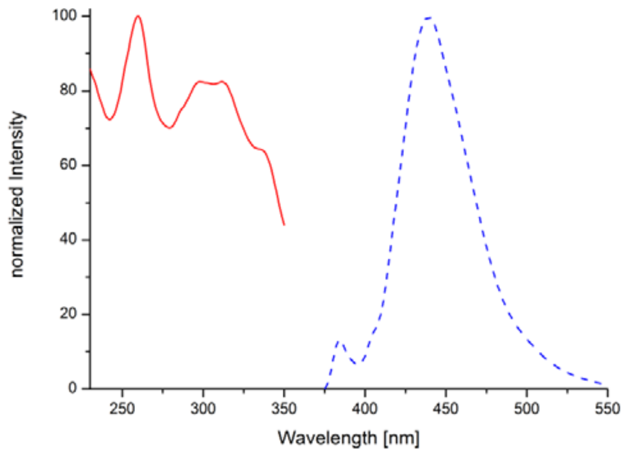

Figure S15. Normalized absorbtion (red, 10 µM, DCM) and fluorescence (blue, 0.1 µM, DCM) spectra of **1e** from **8a**.

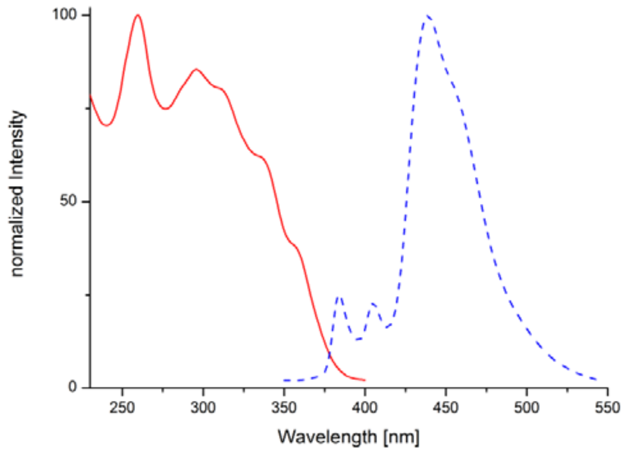

Figure S16. Normalized absorbtion (red, 10 µM, DCM) and fluorescence (blue, 0.25 µM, DCM) spectra of **1e.**

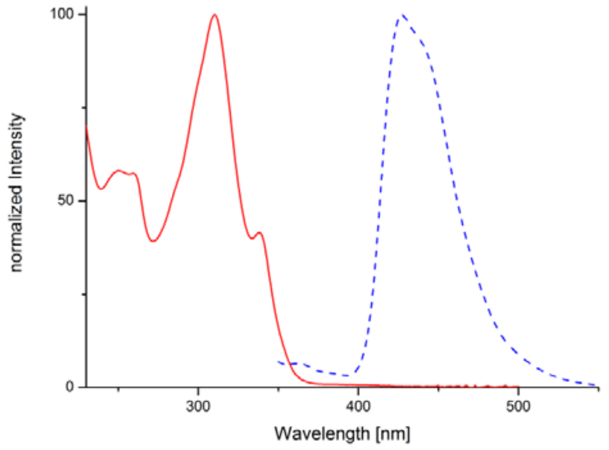

Figure S17. Normalized absorbtion (red, 10 µM, DCM) and fluorescence (blue, 0.08 µM, DCM) spectra of **20b**.

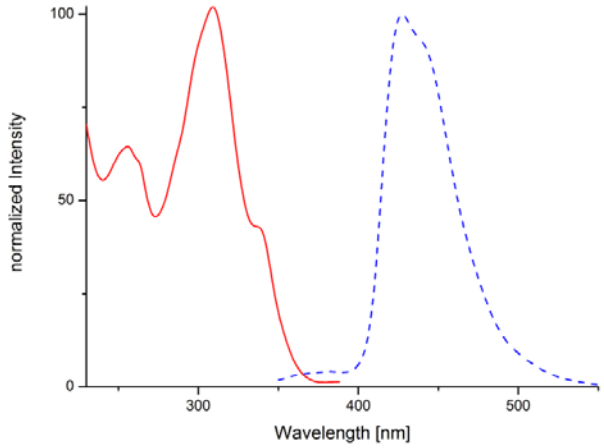

Figure S18. Normalized absorbtion (red, 10 µM, DCM) and fluorescence (blue, 0.07 µM, DCM) spectra of **20c**.

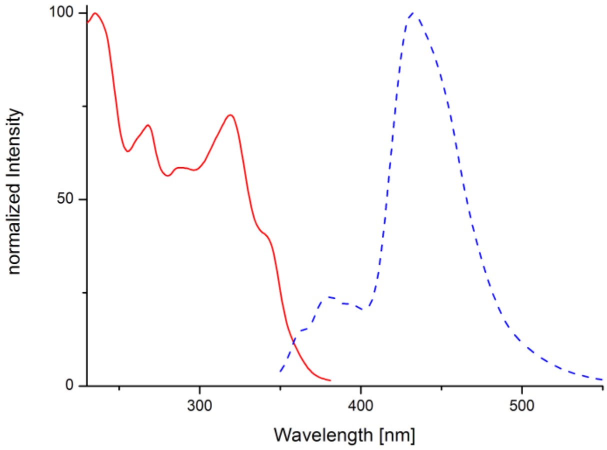

Figure S19. Normalized absorbtion (red, 10 µM, DCM) and fluorescence (blue, 0.25 µM, DCM) spectra of **20d**.

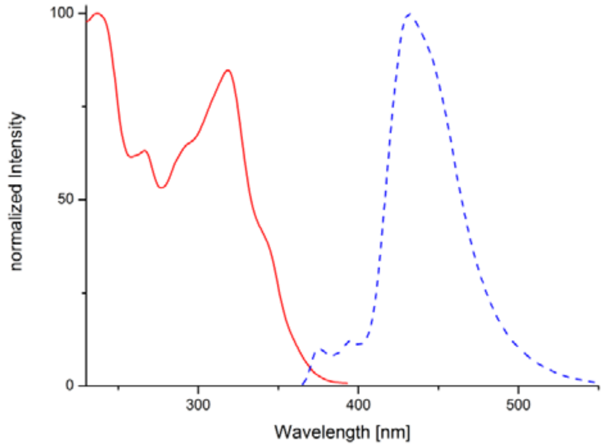

Figure S20. Normalized absorbtion (red, 10 µM, DCM) and fluorescence (blue, 0.08 µM, DCM) spectra of **20e**.

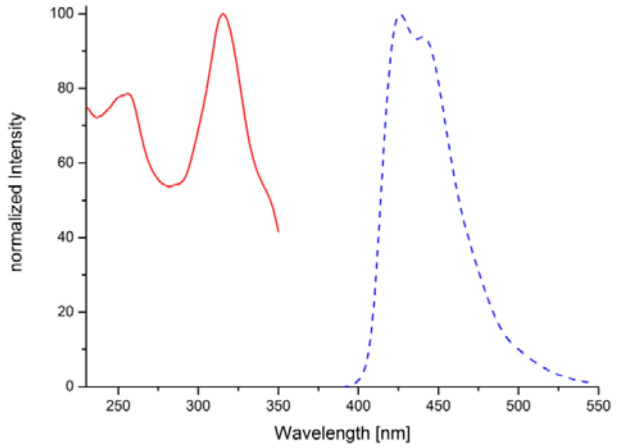

Figure S21. Normalized absorbtion (red, 10 µM, DCM) and fluorescence (blue, 0.1 µM, DCM) spectra of **20h**.

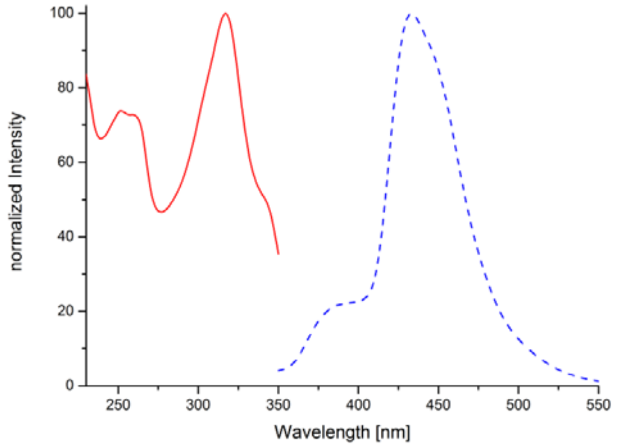

Figure S22. Normalized absorbtion (red, 10 µM, DCM) and fluorescence (blue, 0.1 µM, DCM) spectra of **20j**.

NMR-Spectra

1H NMR: (400 MHz, $CDCl_3$) **3a**

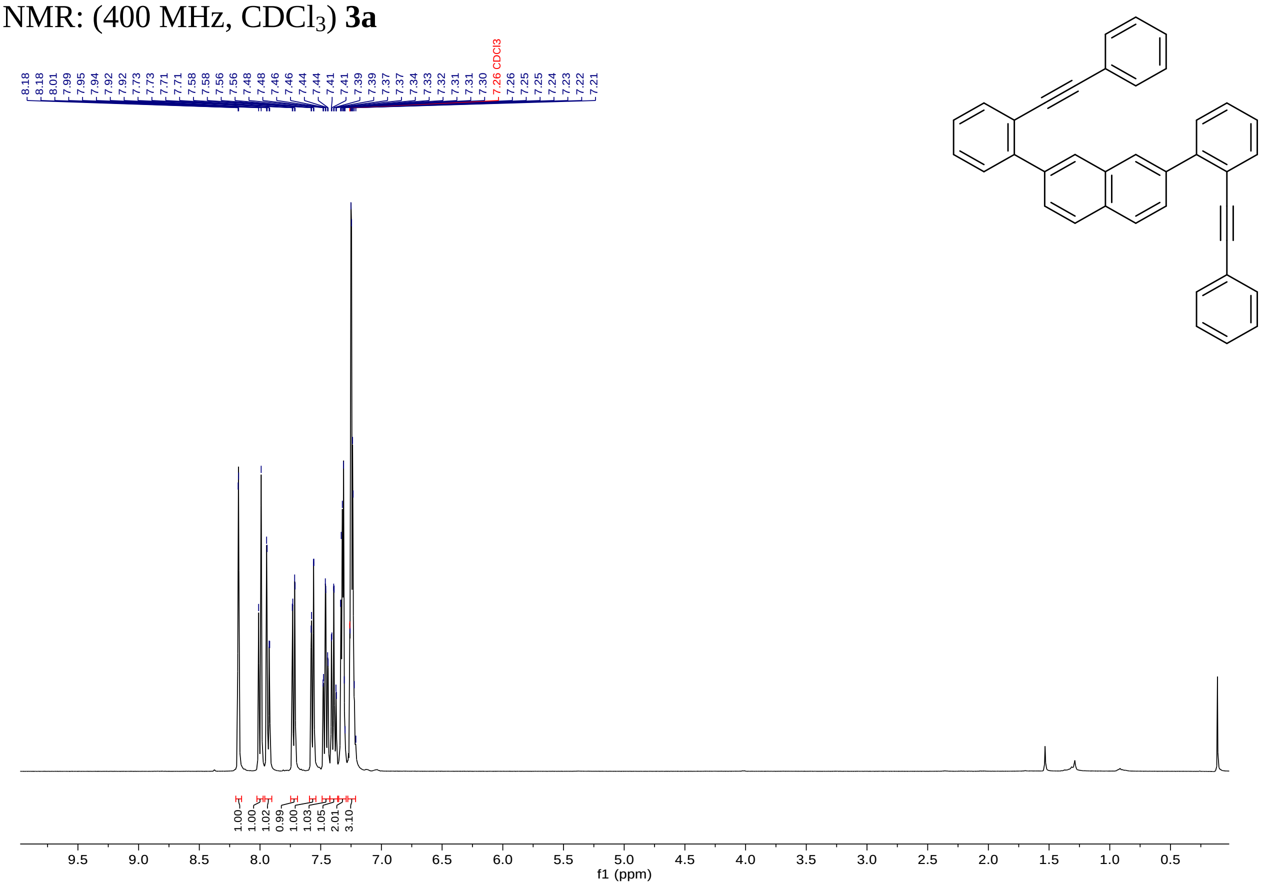

13C{H} NMR: (101 MHz, $CDCl_3$) **3a**

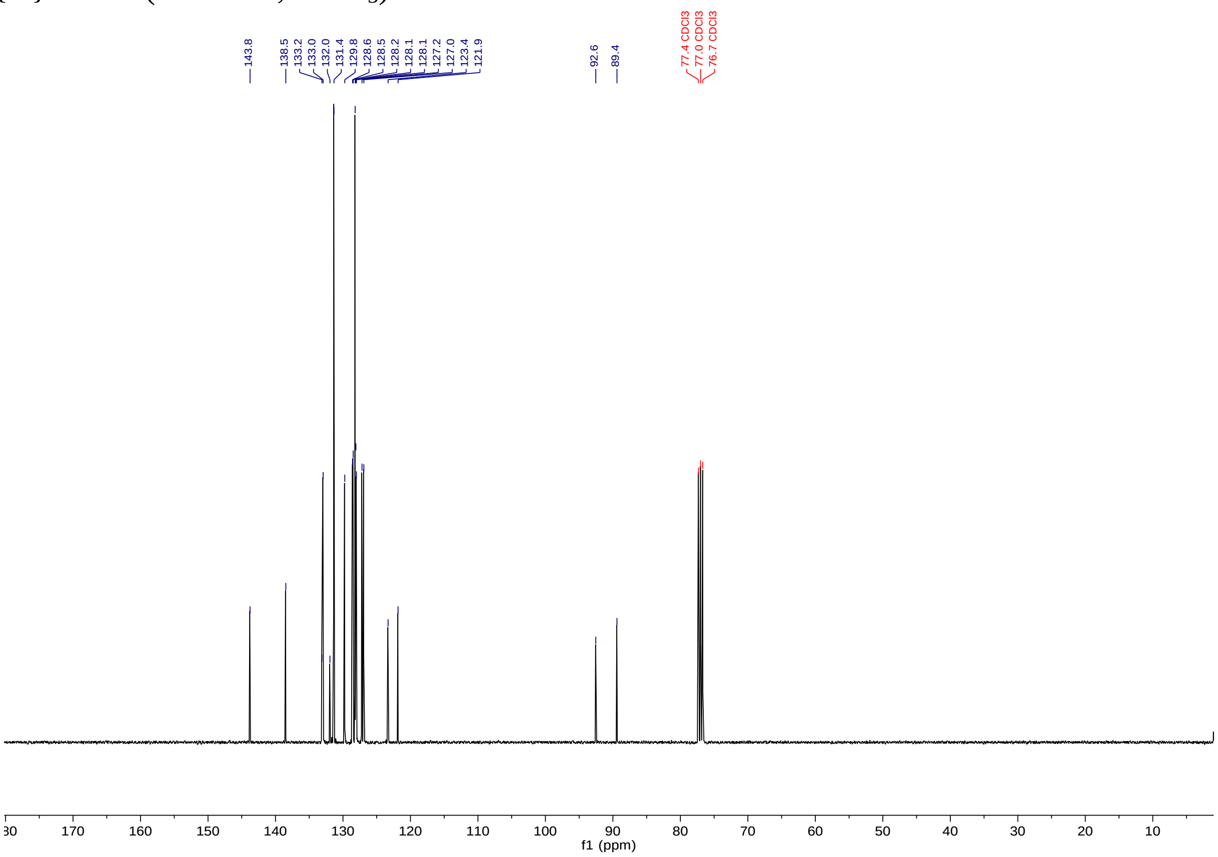

1H NMR: (400 MHz, $CDCl_3$) **3b**

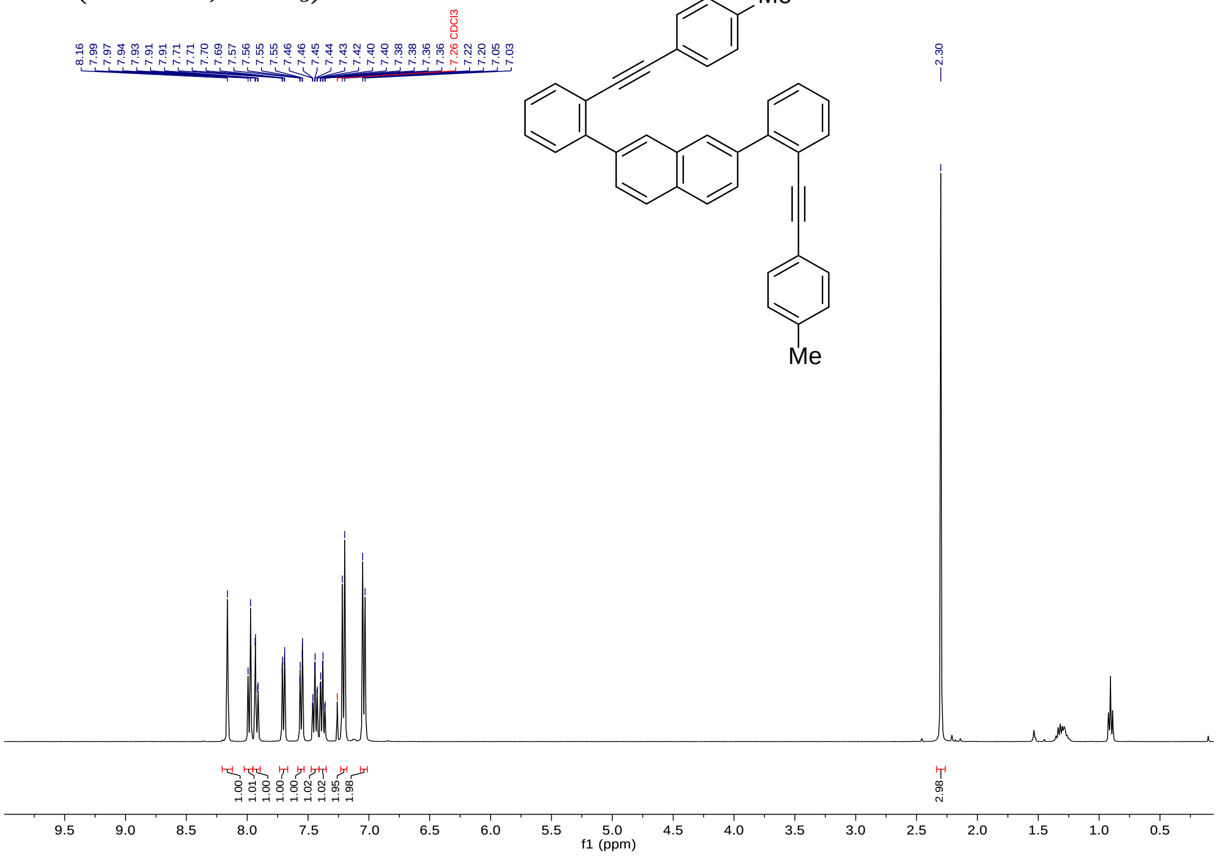

13C{H} NMR: (101 MHz, $CDCl_3$) **3b**

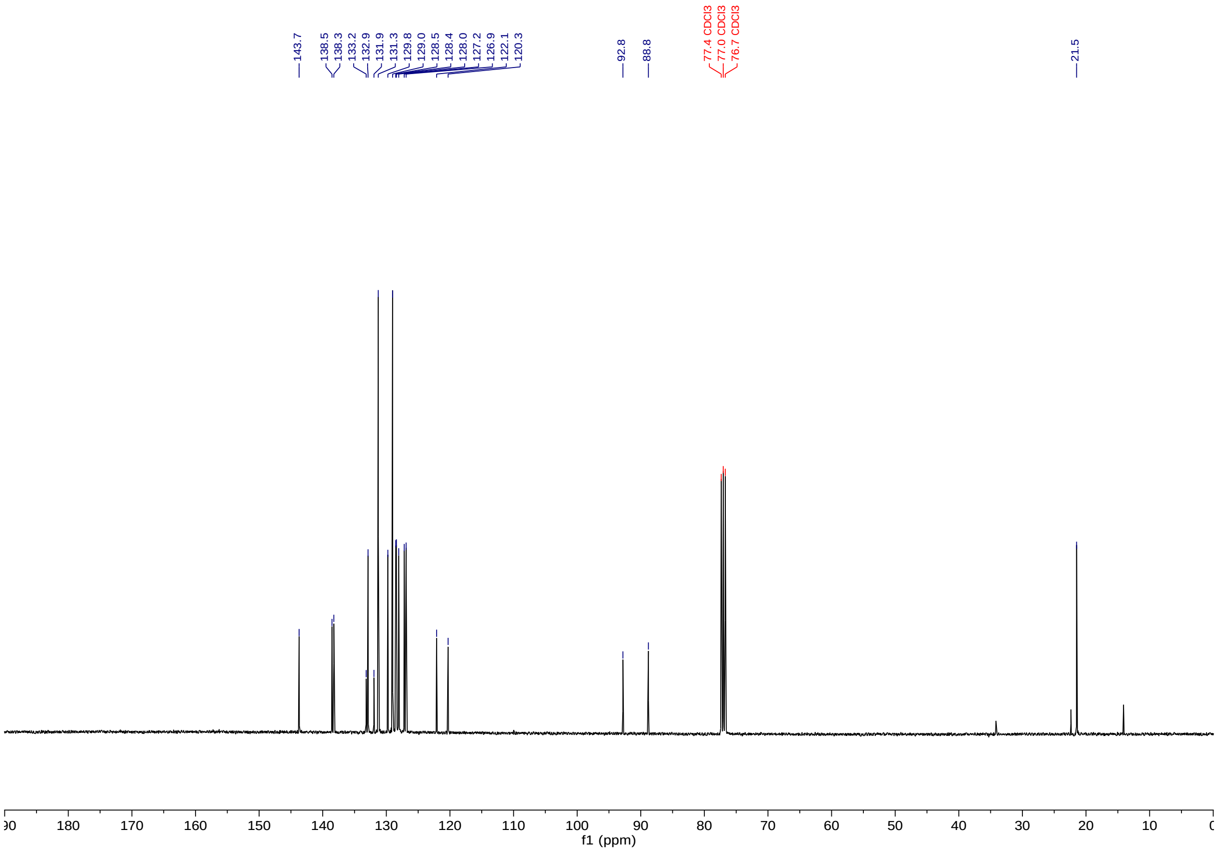

1H NMR: (400 MHz, $CDCl_3$) **3c**

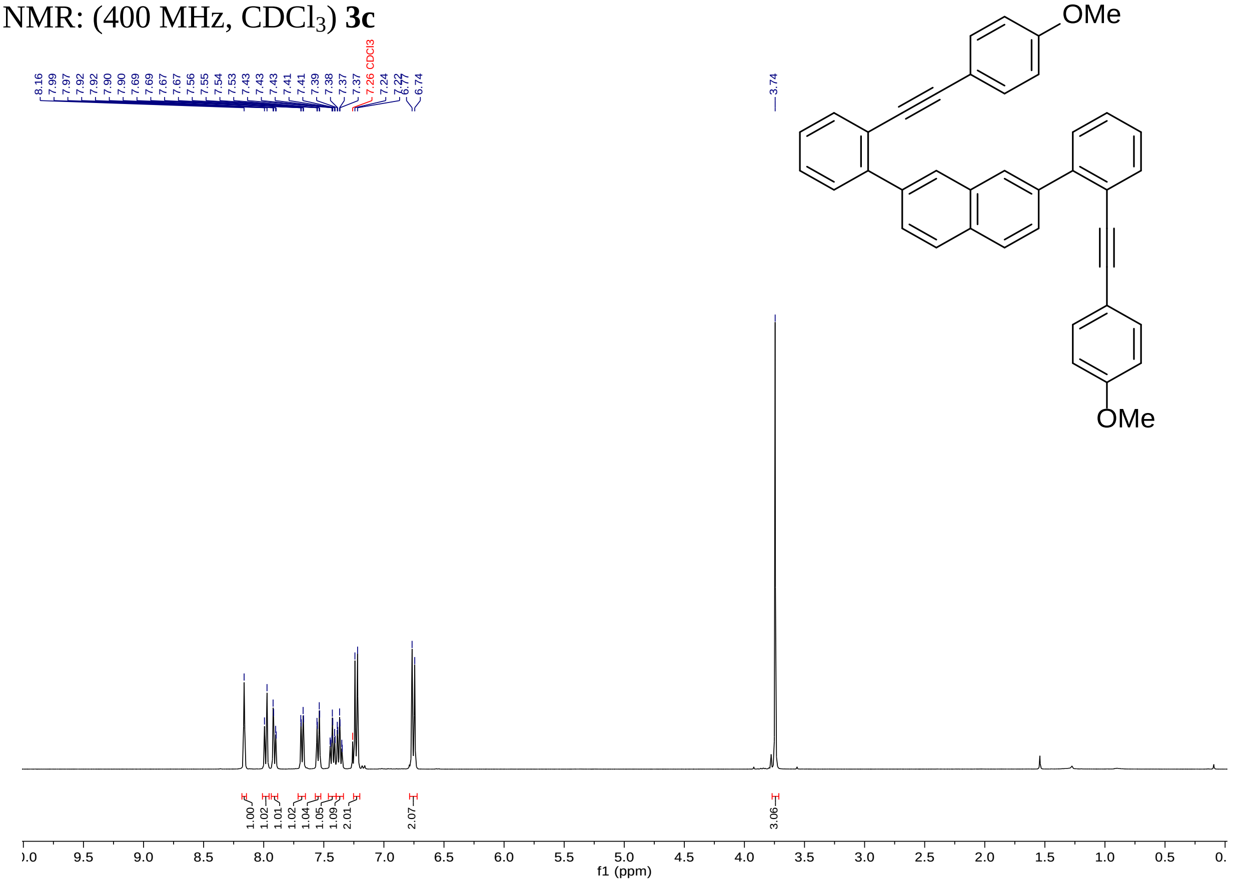

13C{H} NMR: (101 MHz, $CDCl_3$) **3c**

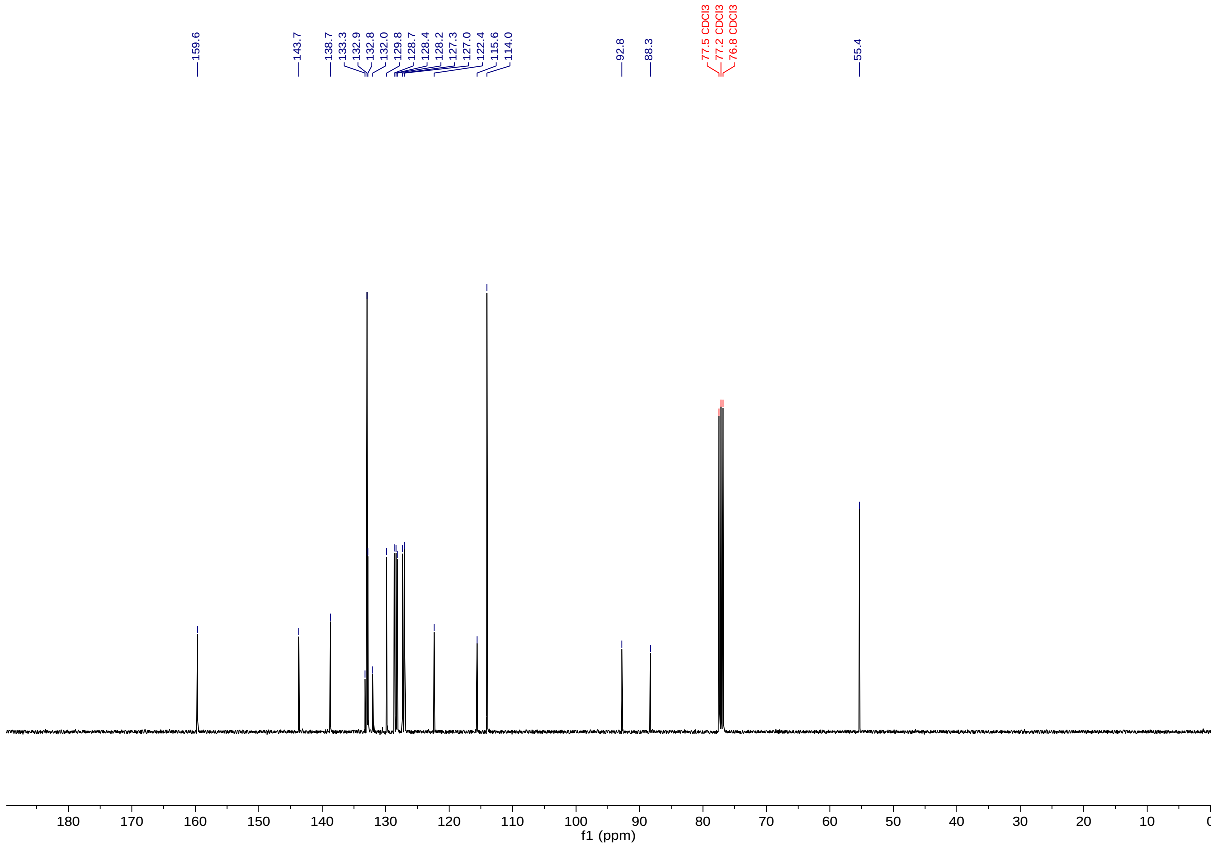

1H NMR: (400 MHz, $CDCl_3$) **3d**

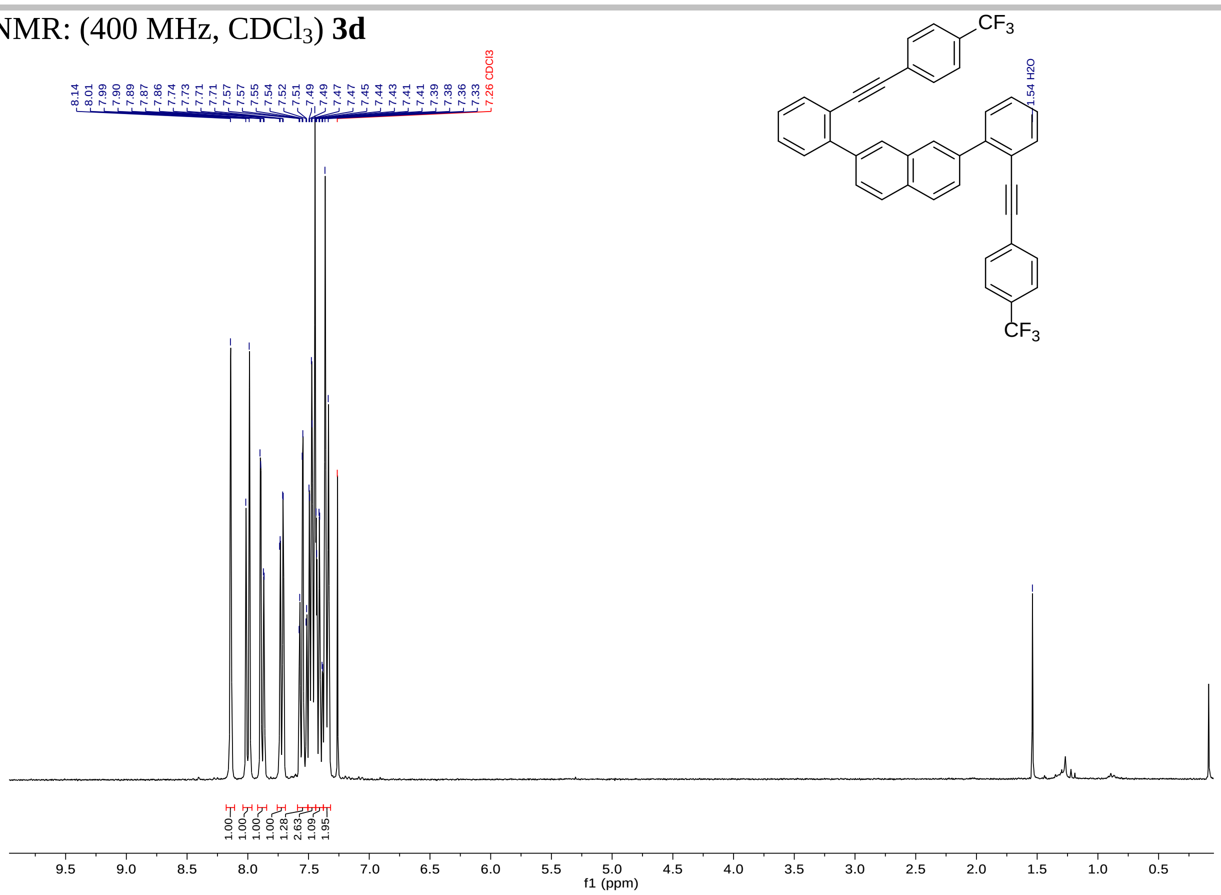

13C{H} NMR: (101 MHz, $CDCl_3$) **3d**

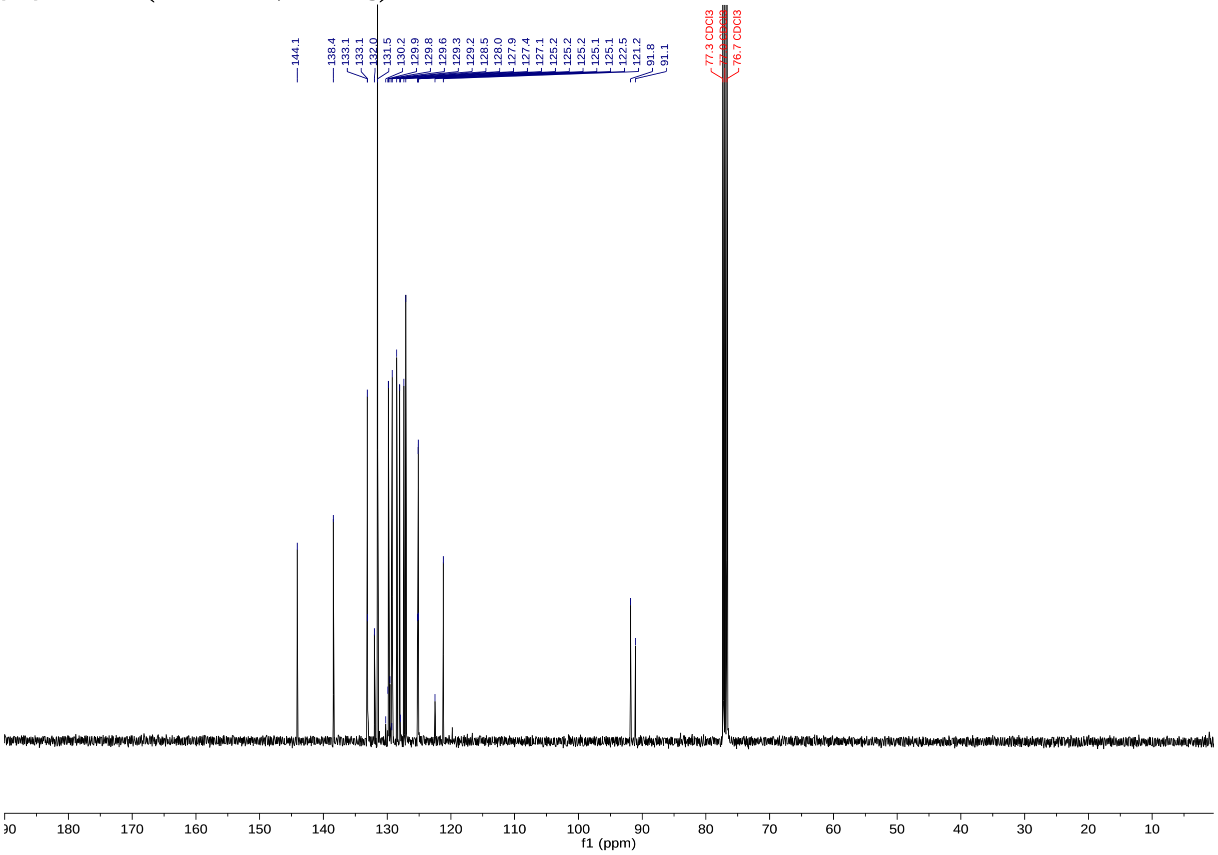

19F{H} NMR: (282 MHz, $CDCl_3$) **3d**

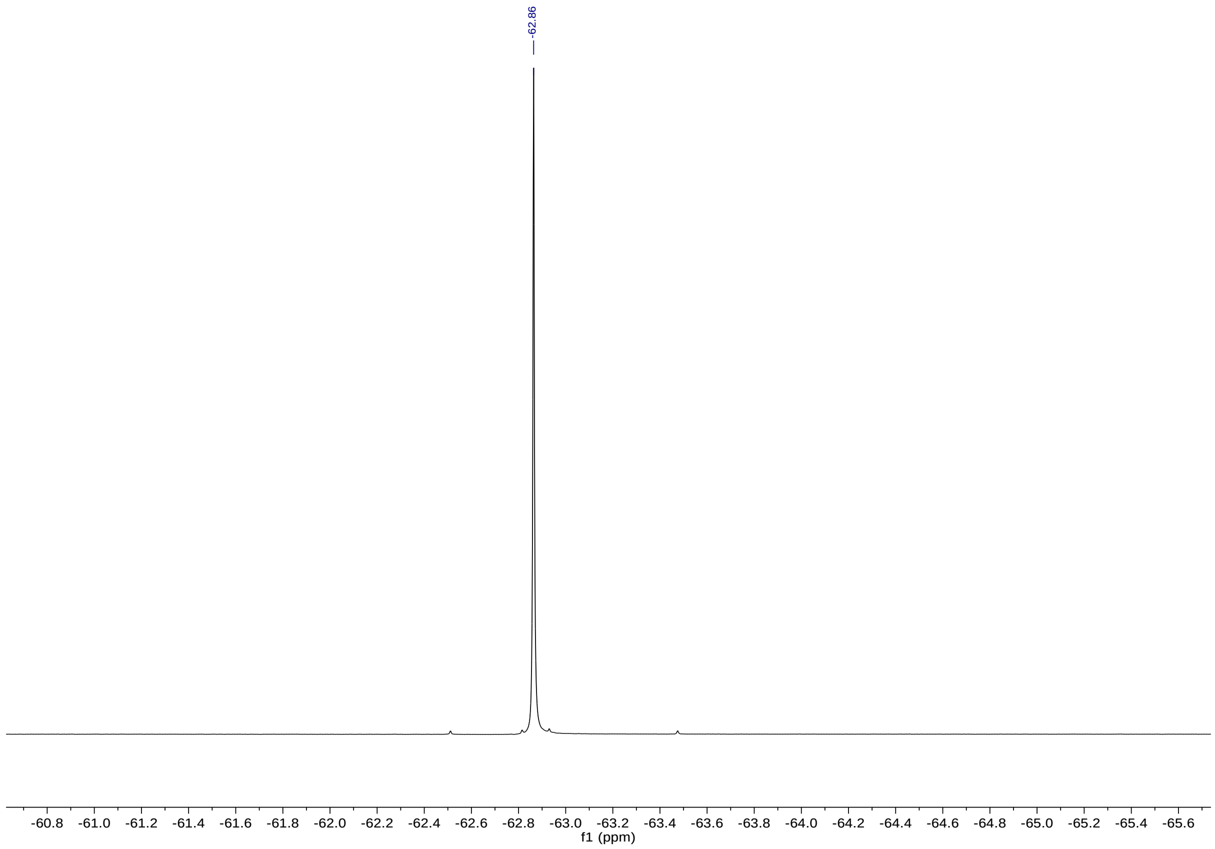

1H NMR: (400 MHz, $CDCl_3$) **1a**

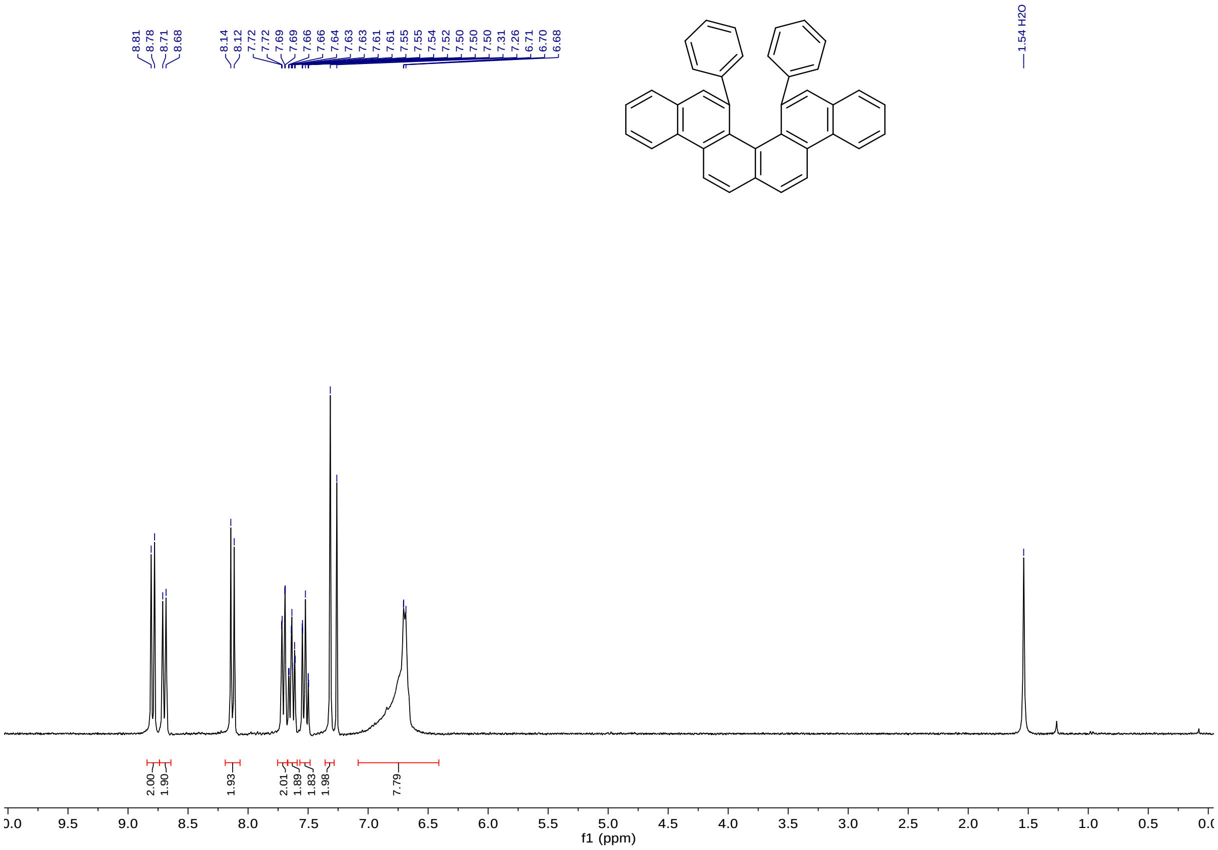

13C{H} NMR: (101 MHz, $CDCl_3$) **1a**

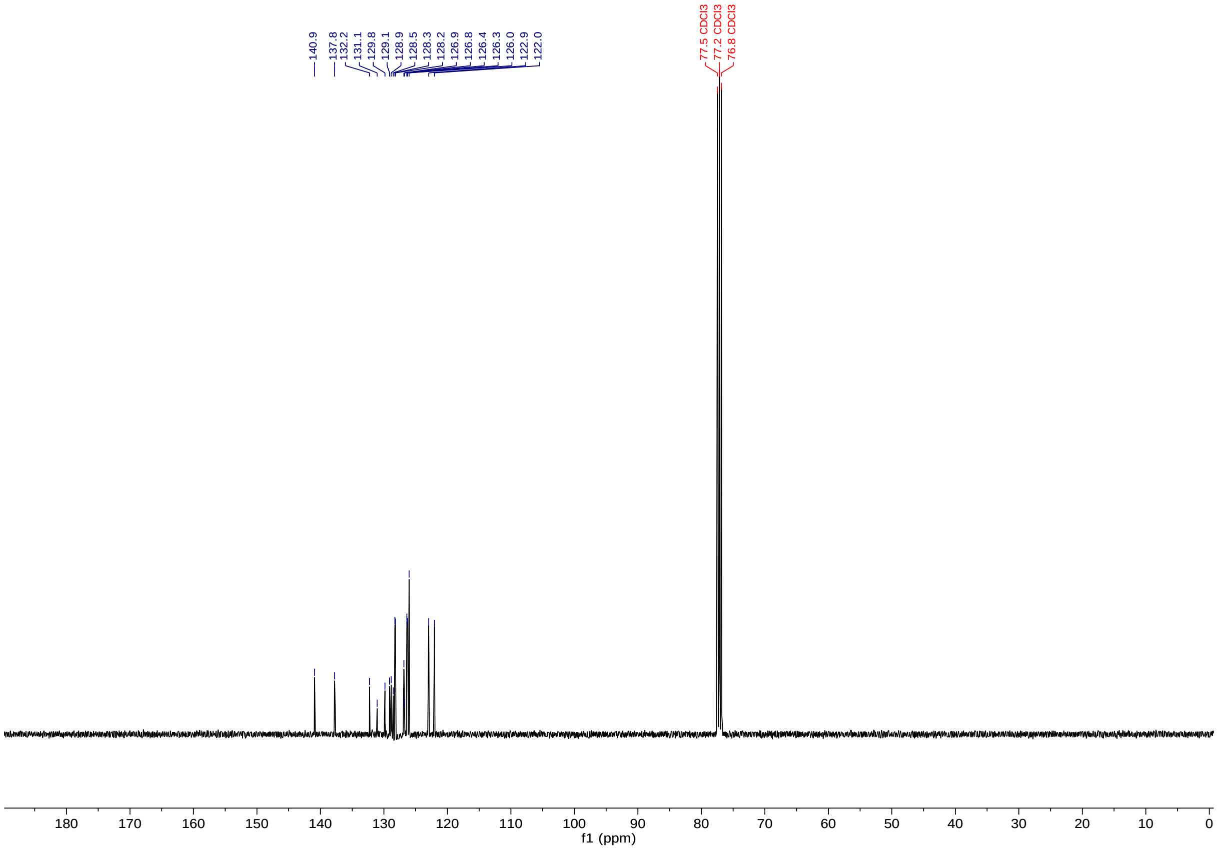

1H NMR: (400 MHz, $CDCl_3$) **1b**

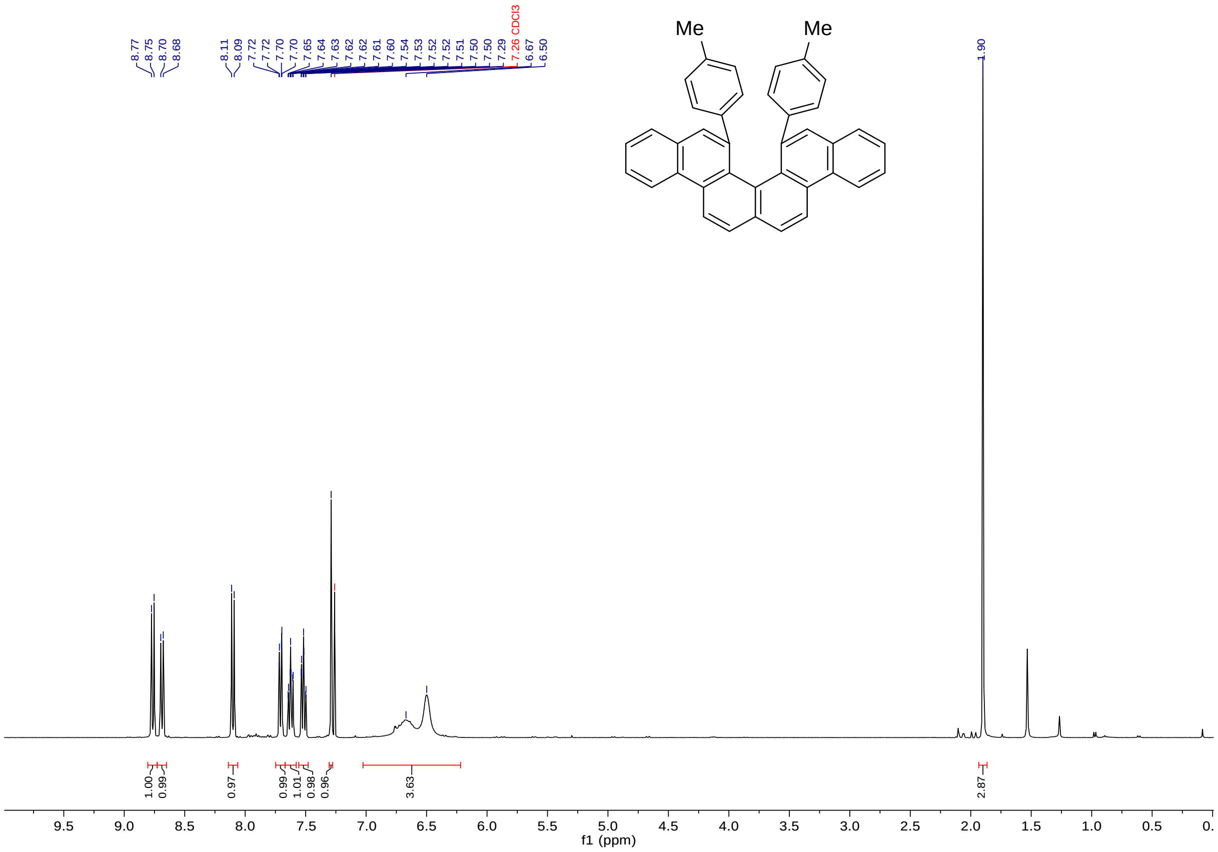

13C{H} NMR: (101 MHz, $CDCl_3$) **1b**

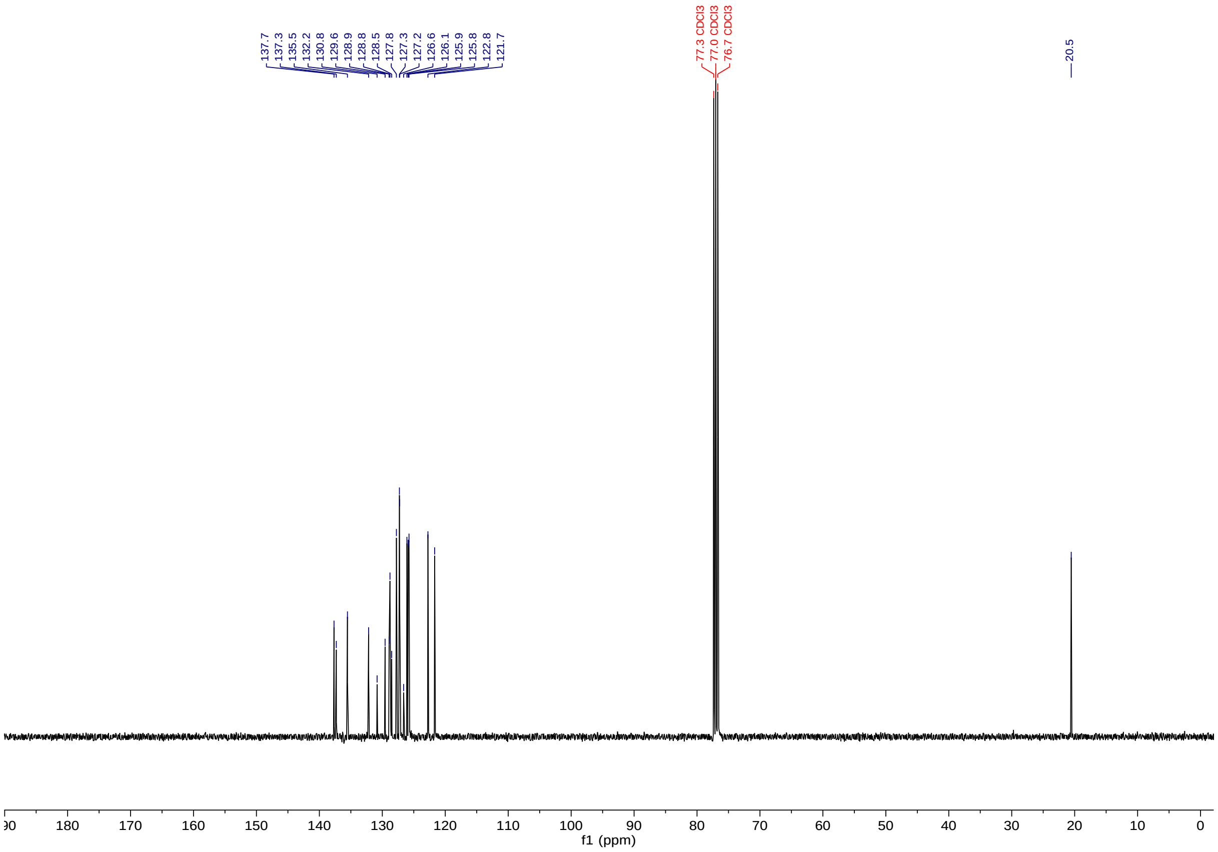

1H NMR: (400 MHz, $CDCl_3$) **1c**

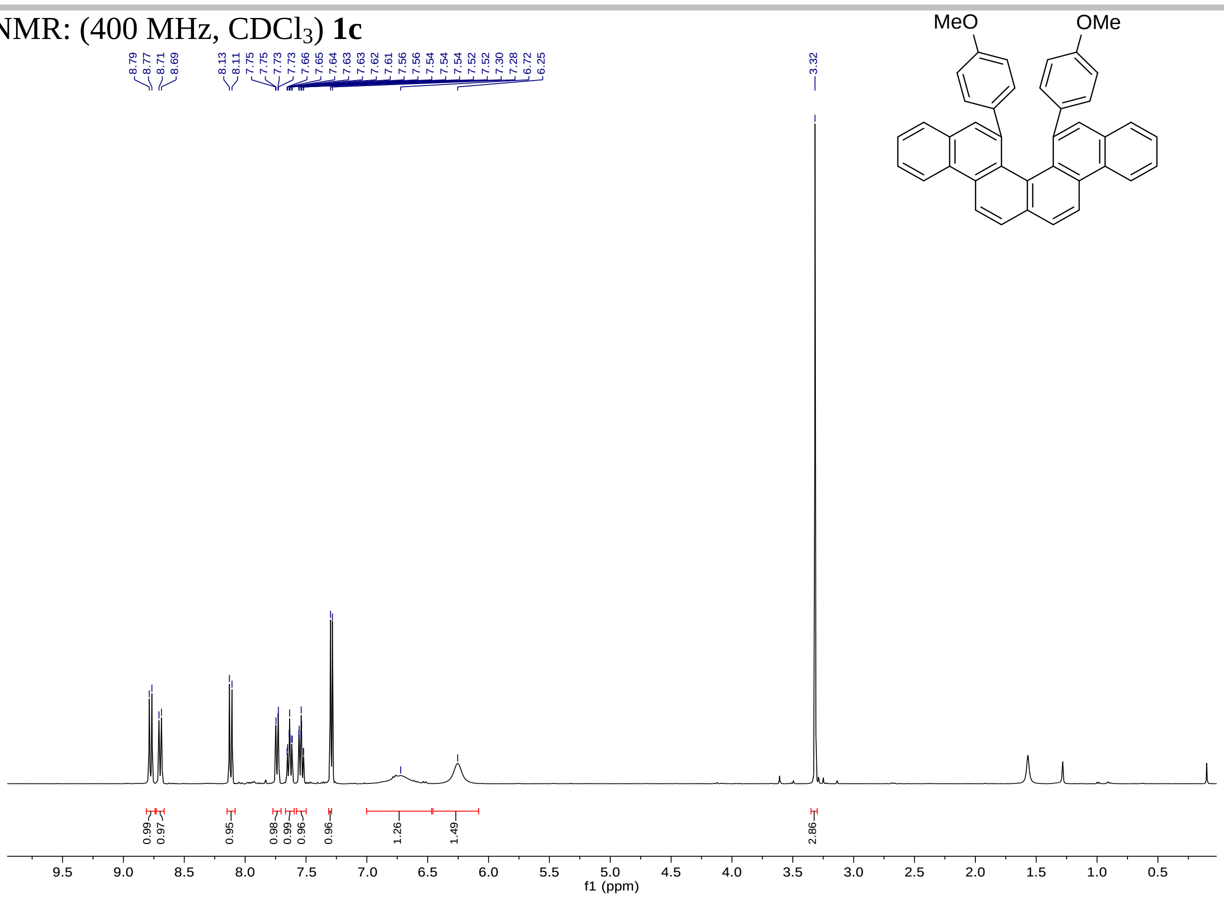

13C{H} NMR: (101 MHz, $CDCl_3$) **1c**

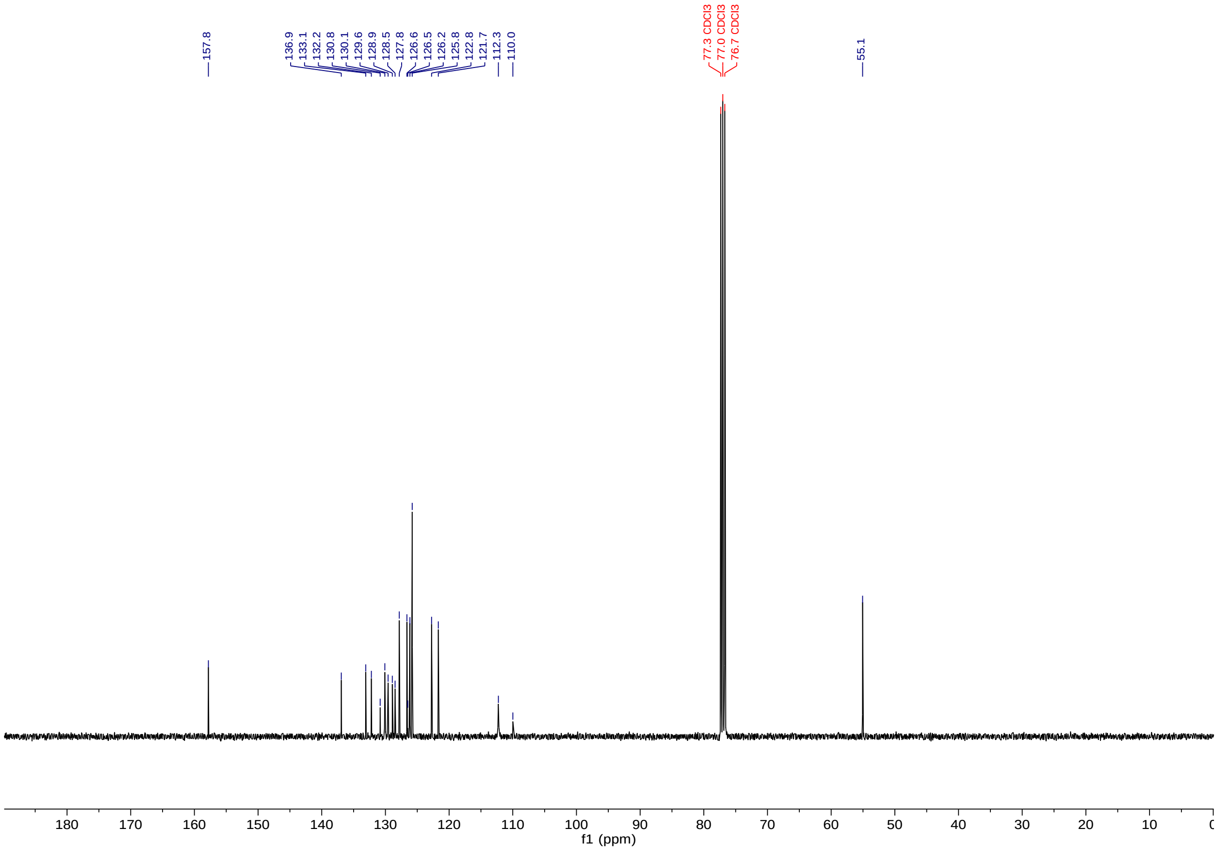

1H NMR: (400 MHz, $CDCl_3$) **1d**

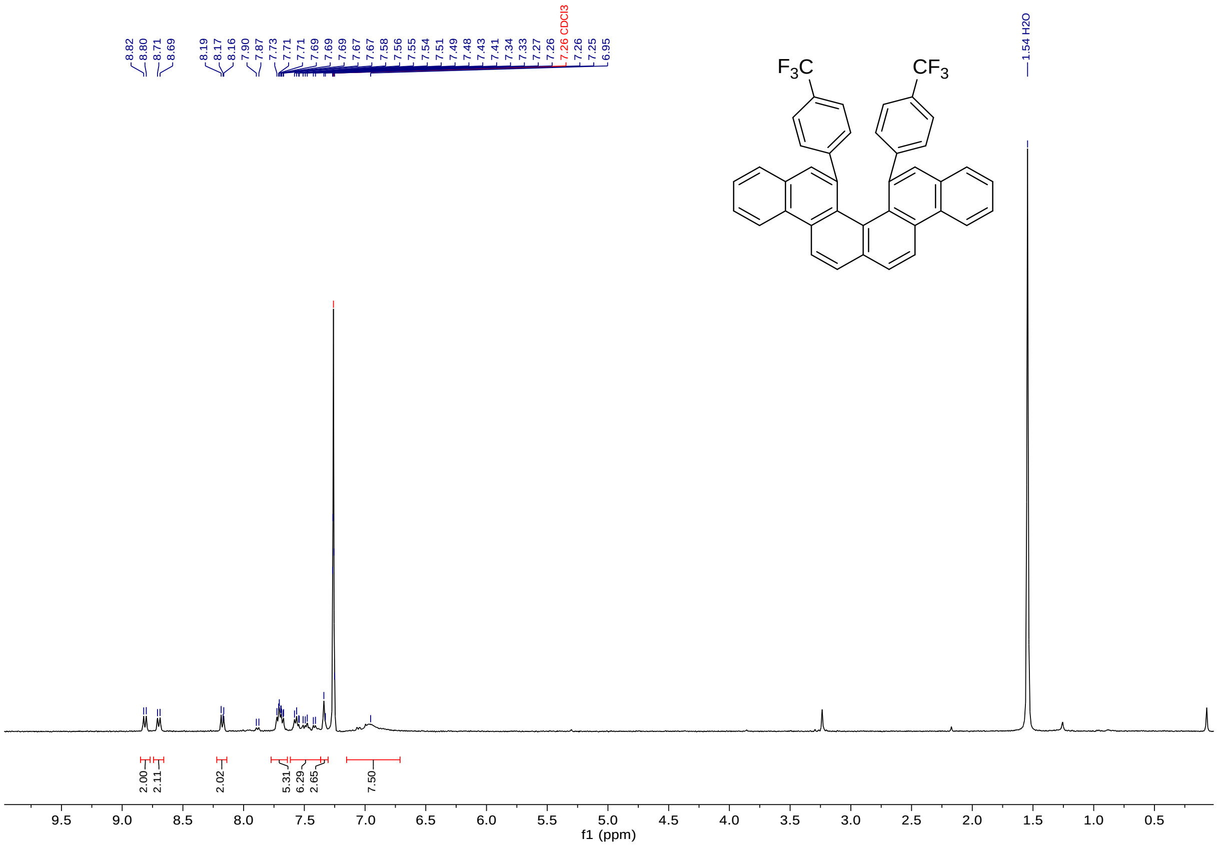

19F NMR: (377 MHz, $CDCl_3$) **1d**

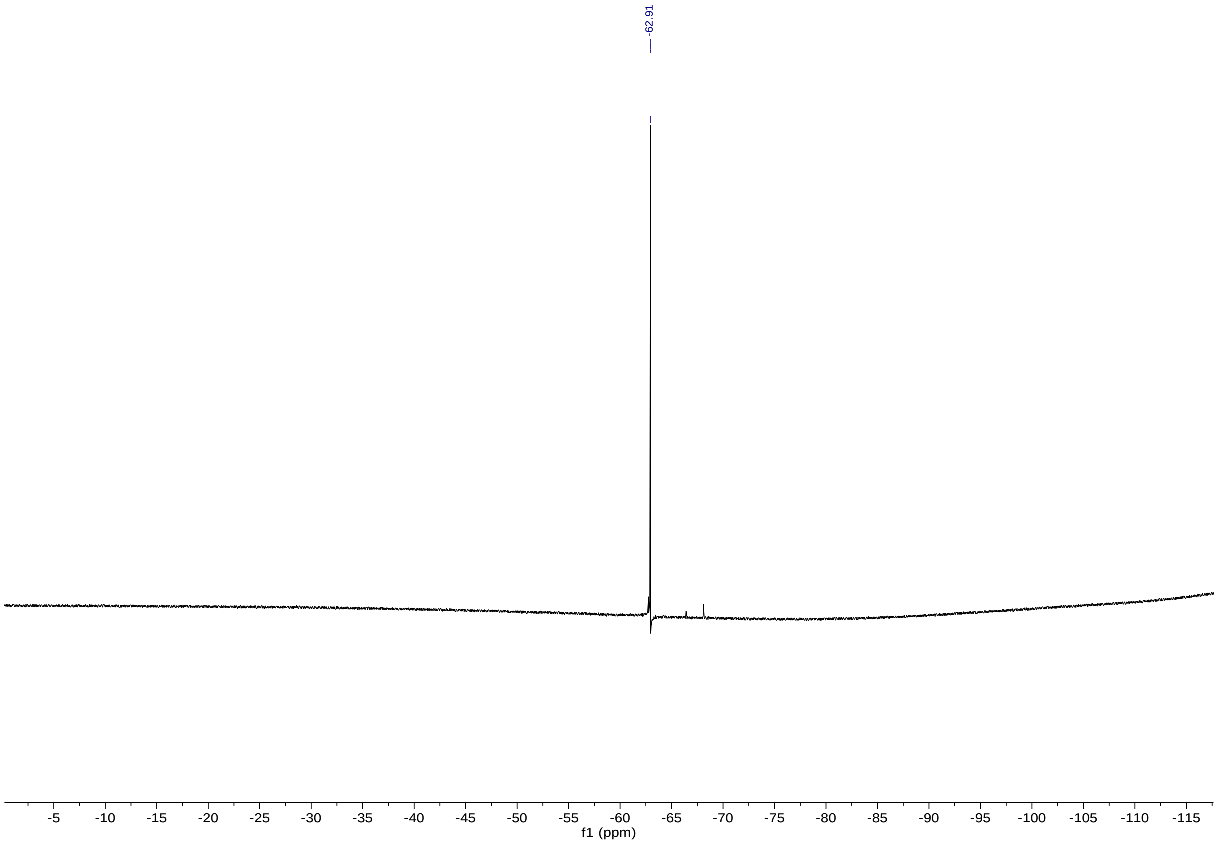

1H NMR: (400 MHz, $CDCl_3$) **21a**

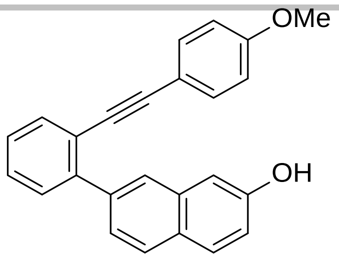

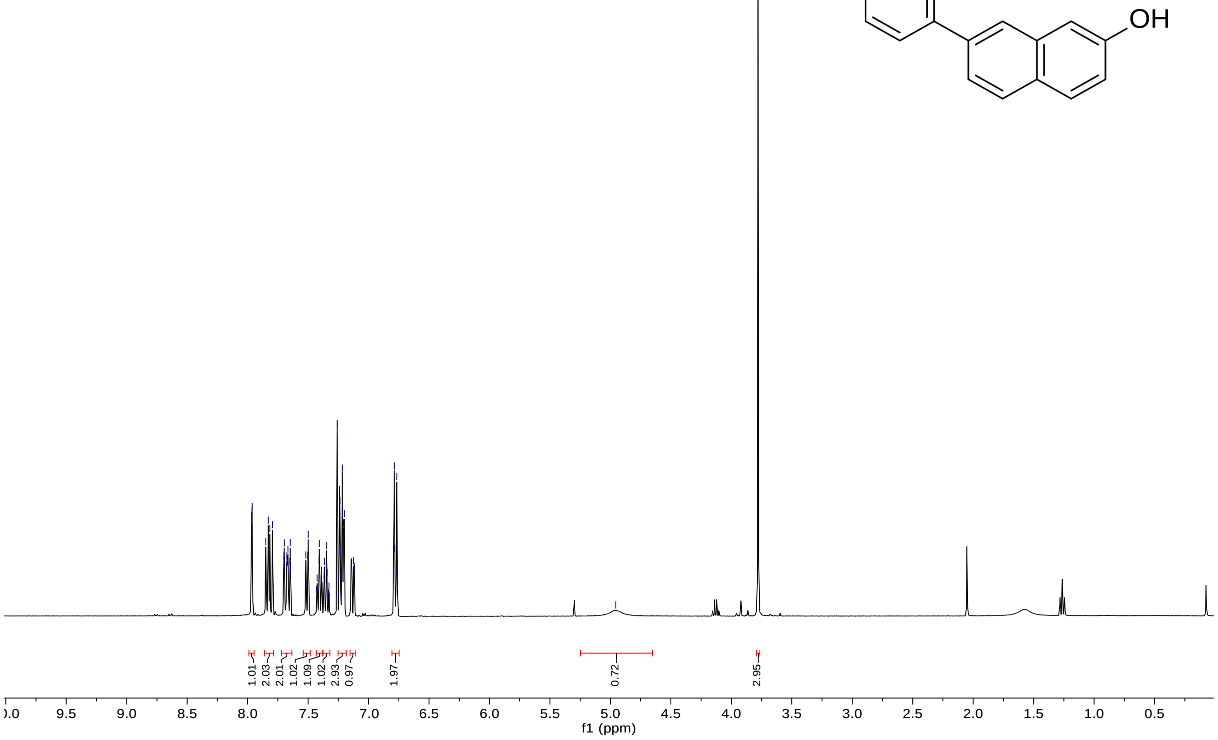

13C{H} NMR: (101 MHz, $CDCl_3$)

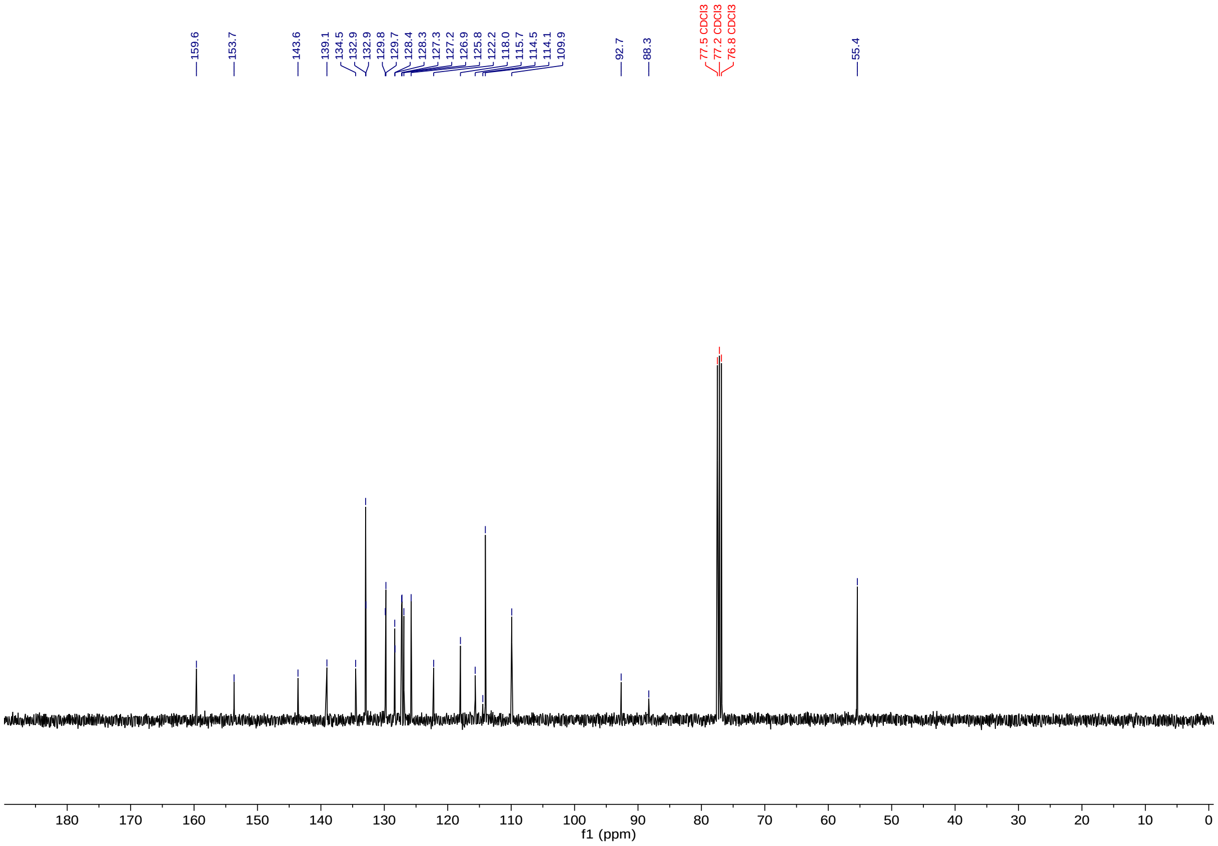

1H NMR: (400 MHz, $CDCl_3$) **21b**

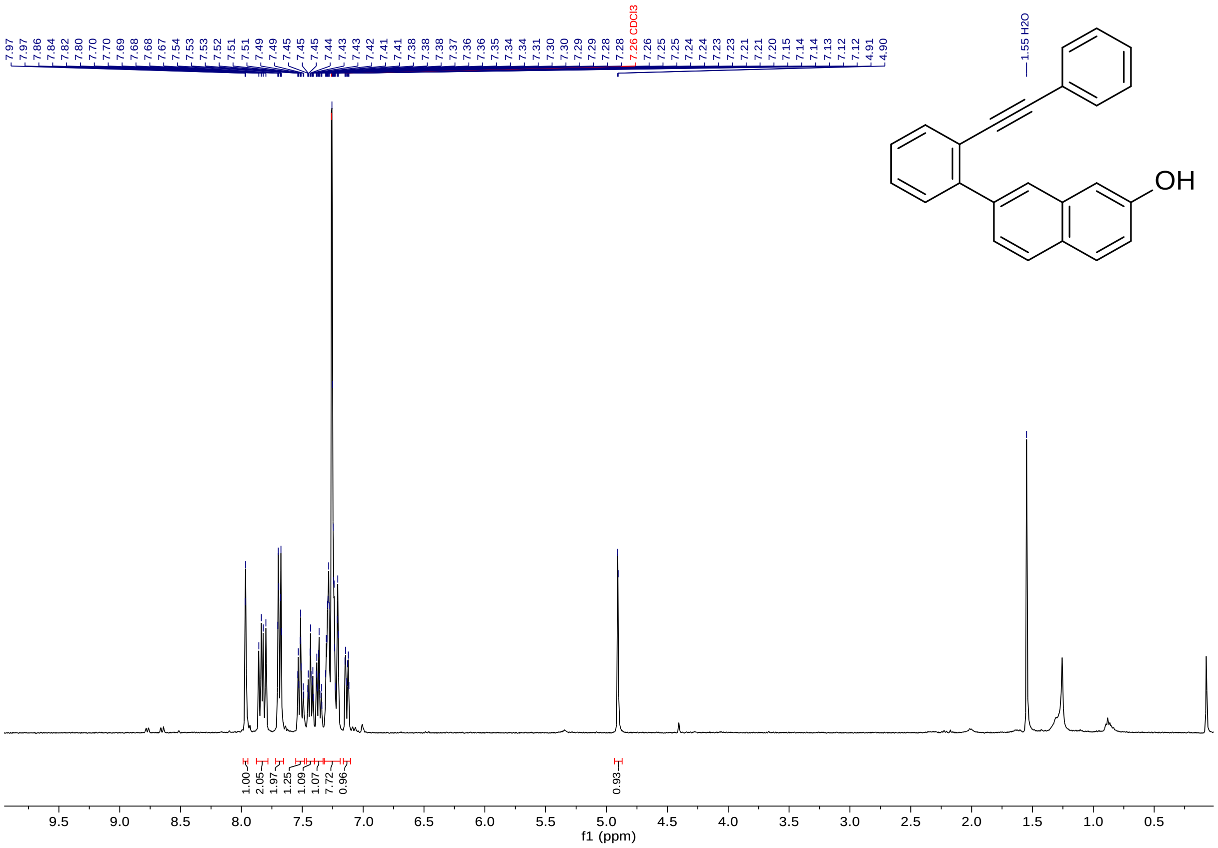

13C{H} NMR: (101 MHz, $CDCl_3$)

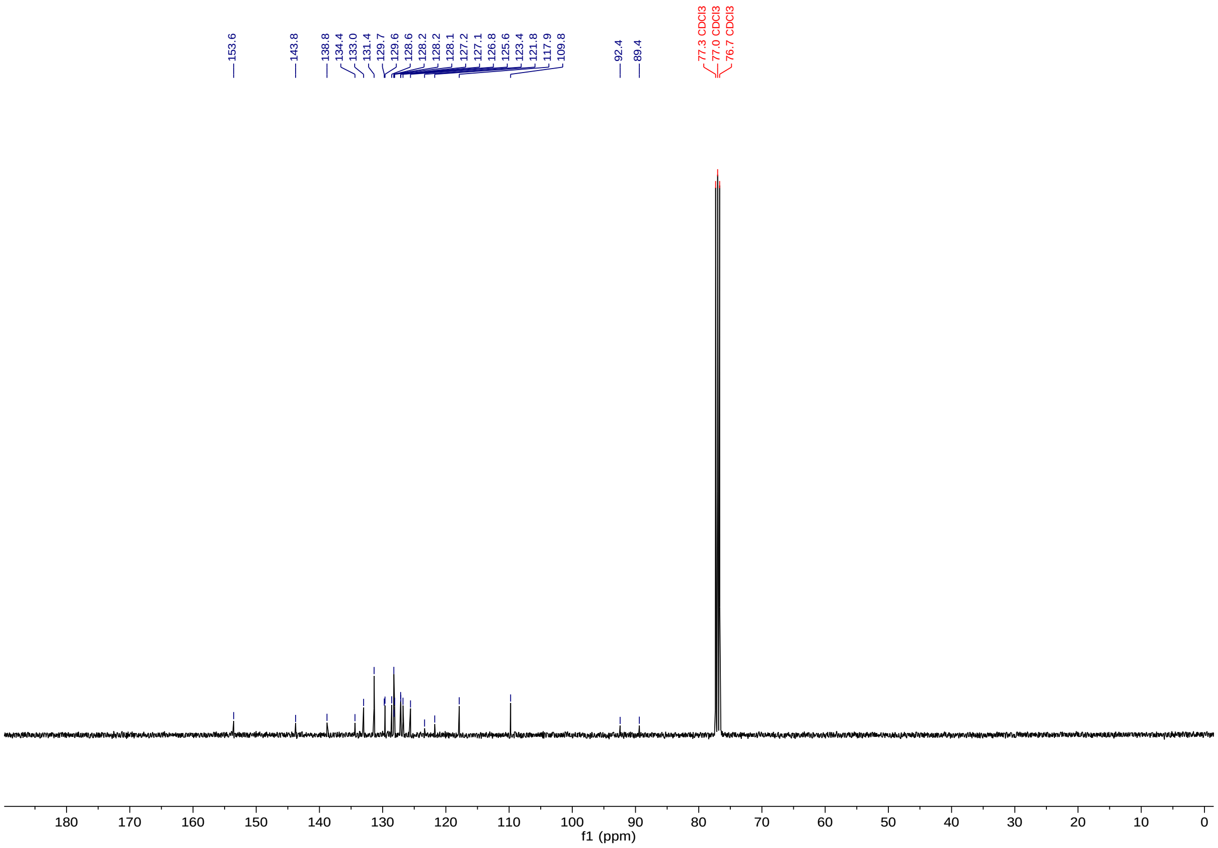

1H NMR: (400 MHz, $CDCl_3$) **7a**

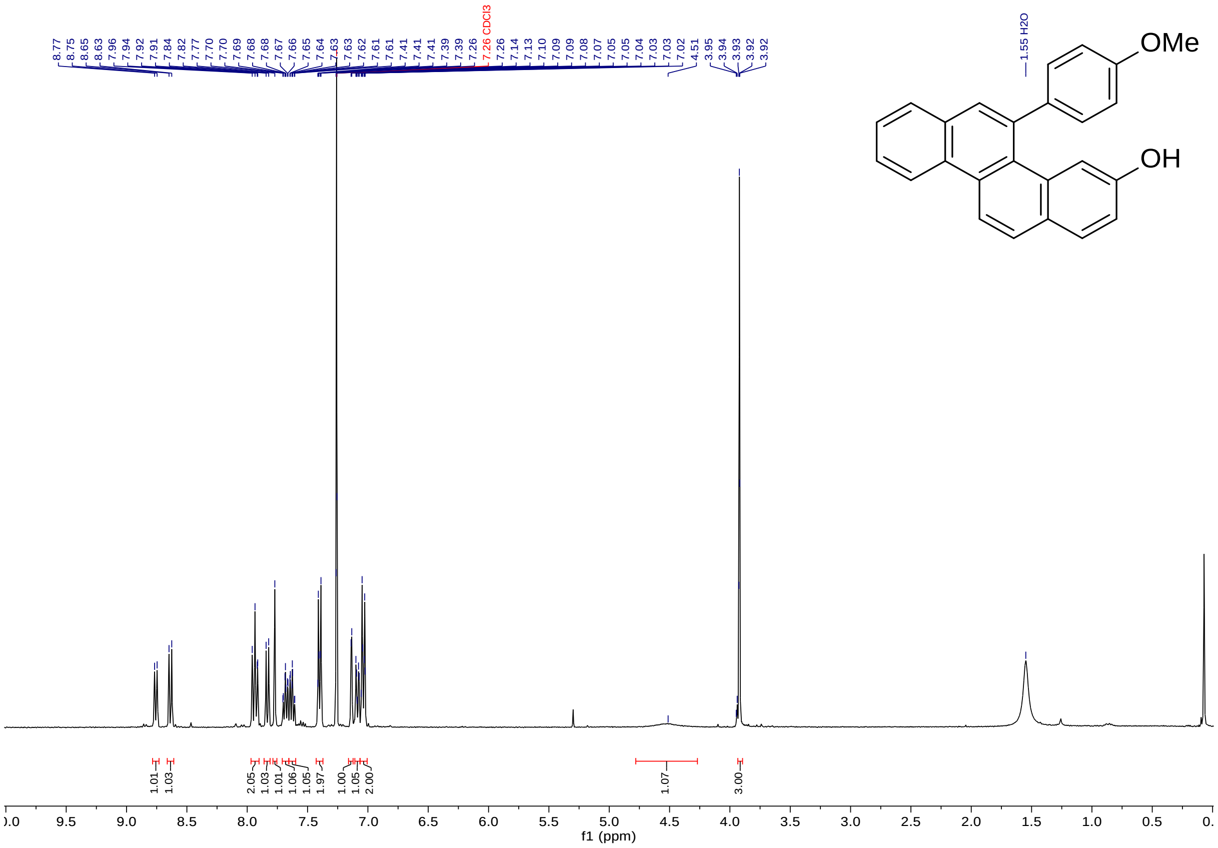

13C{H} NMR: (101 MHz, $CDCl_3$) **7a**

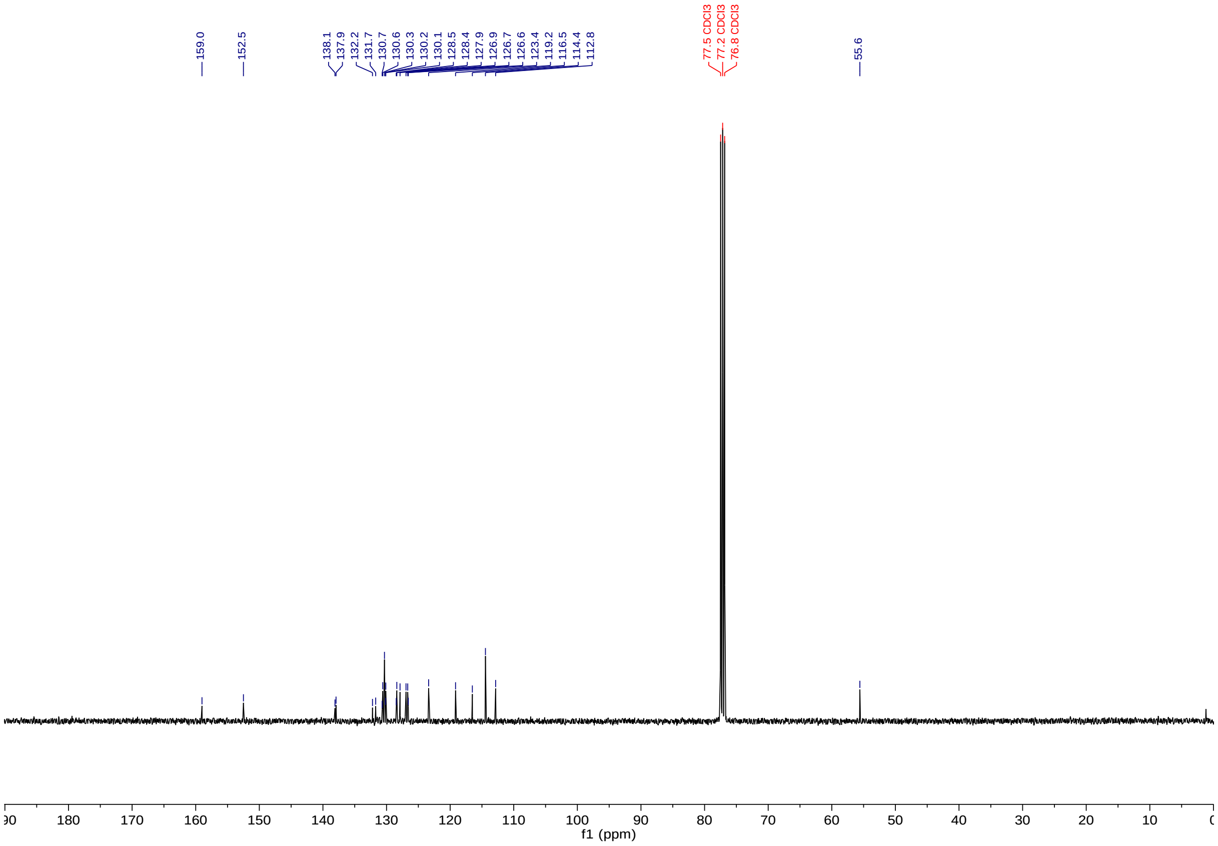

1H NMR: (400 MHz, $CDCl_3$) **7b**

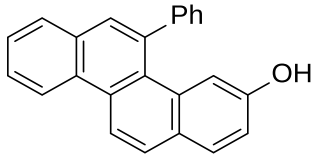

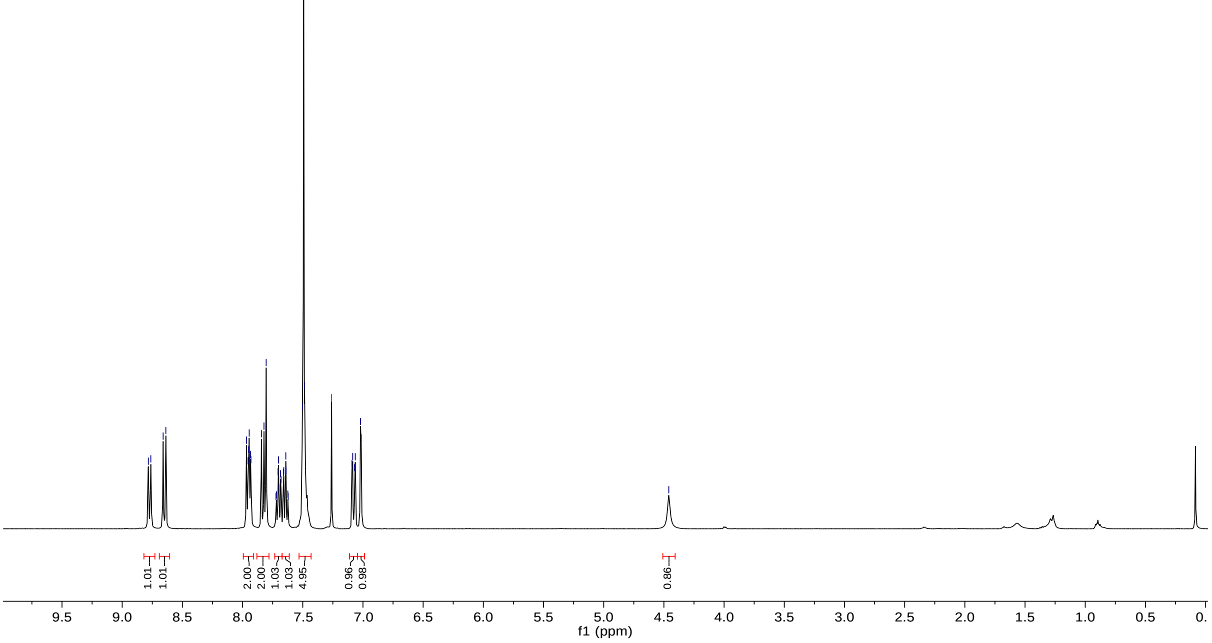

13C{H} NMR: (101 MHz, $CDCl_3$) **7b**

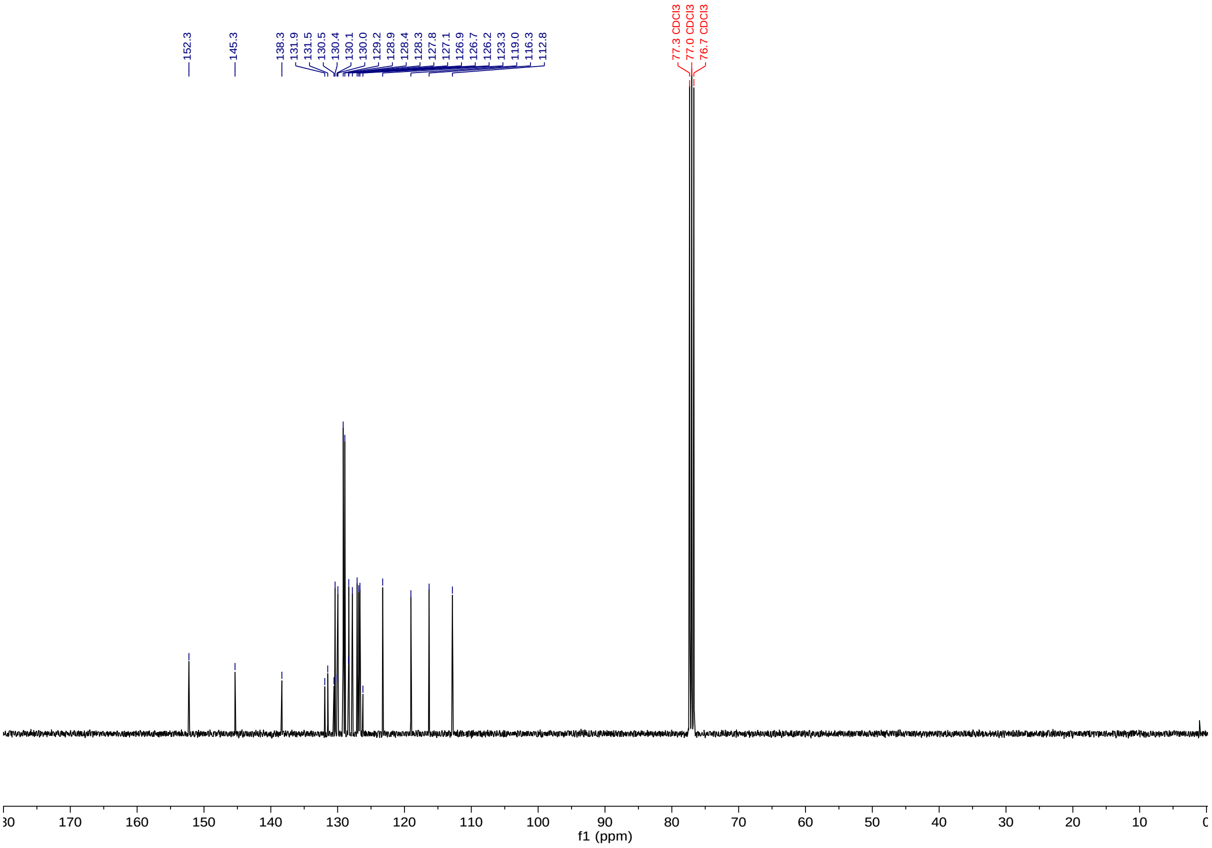

1H NMR: (400 MHz, $CDCl_3$) **22a**

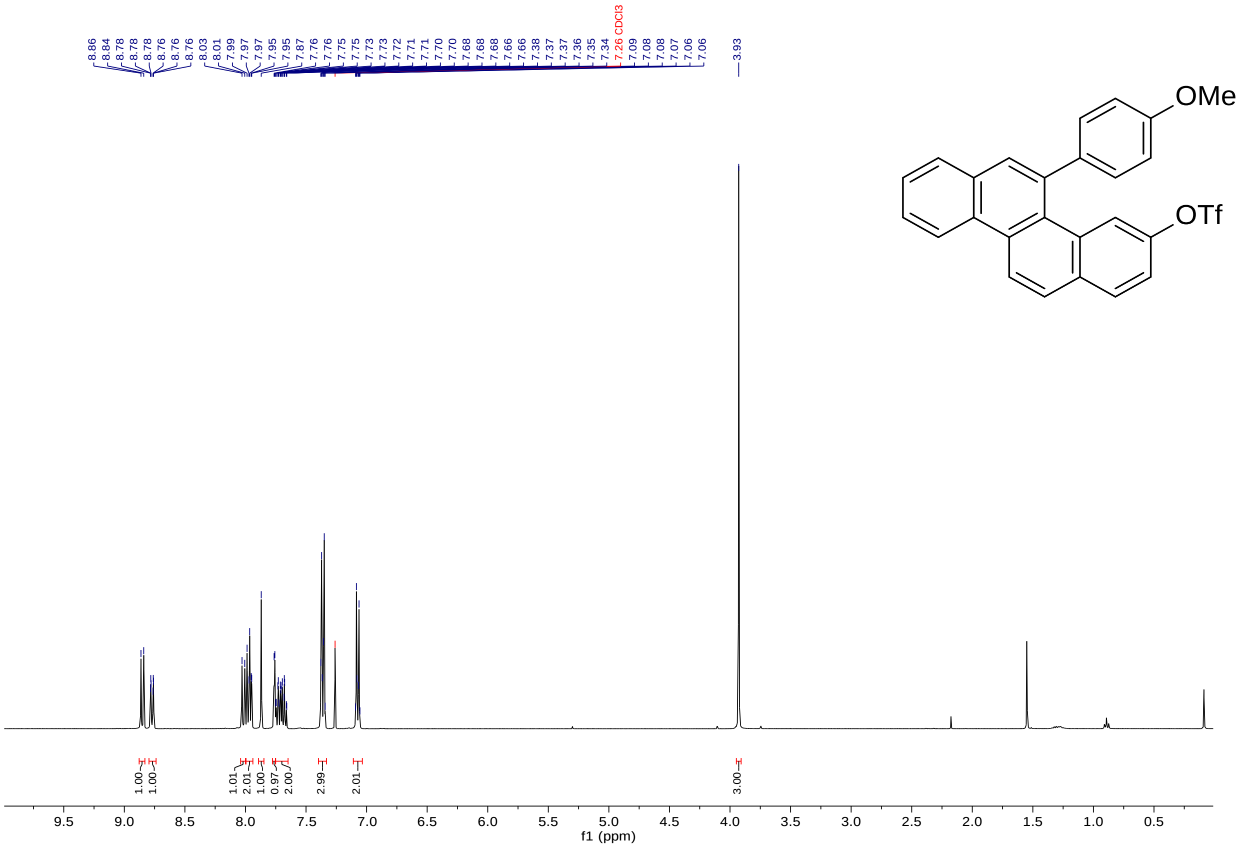

13C{H} NMR: (101 MHz, $CDCl_3$) **22a**

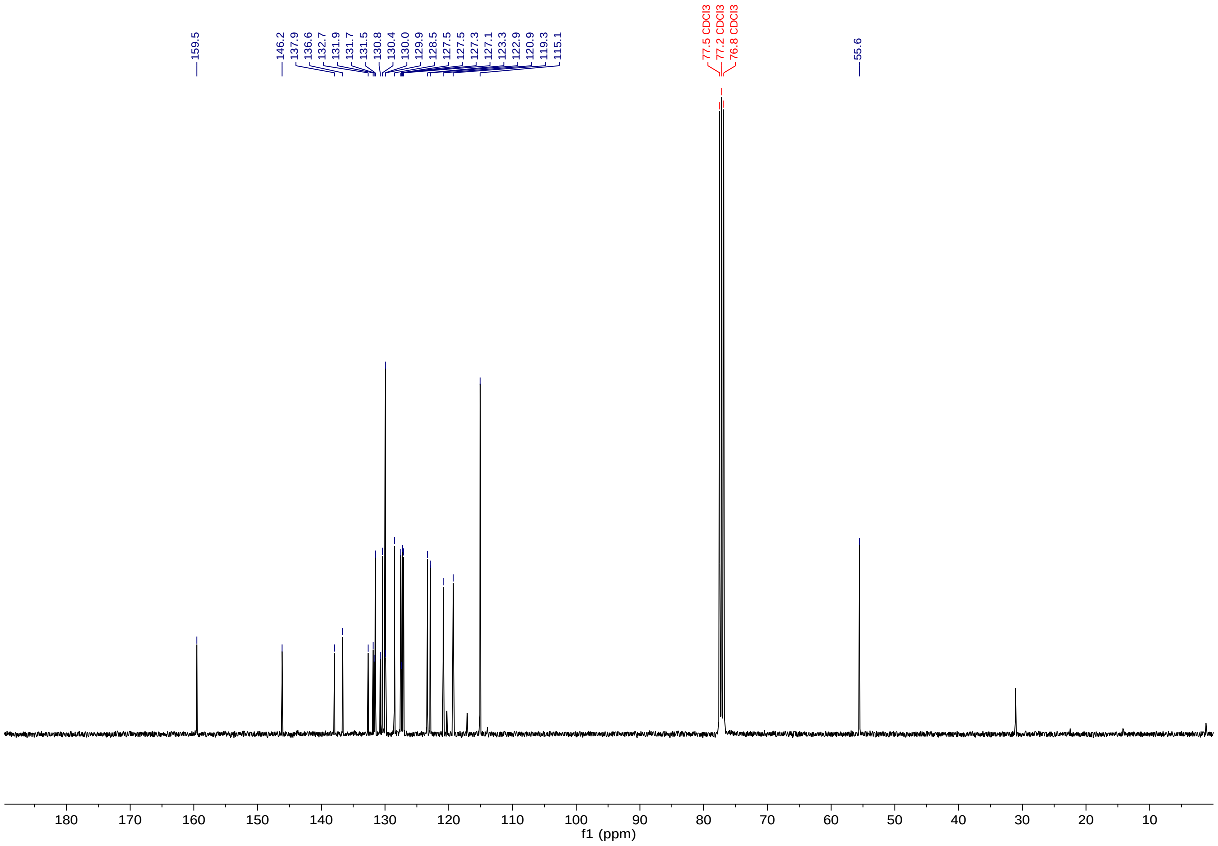

19F{H} NMR: (377 MHz, $CDCl_3$) **22a**

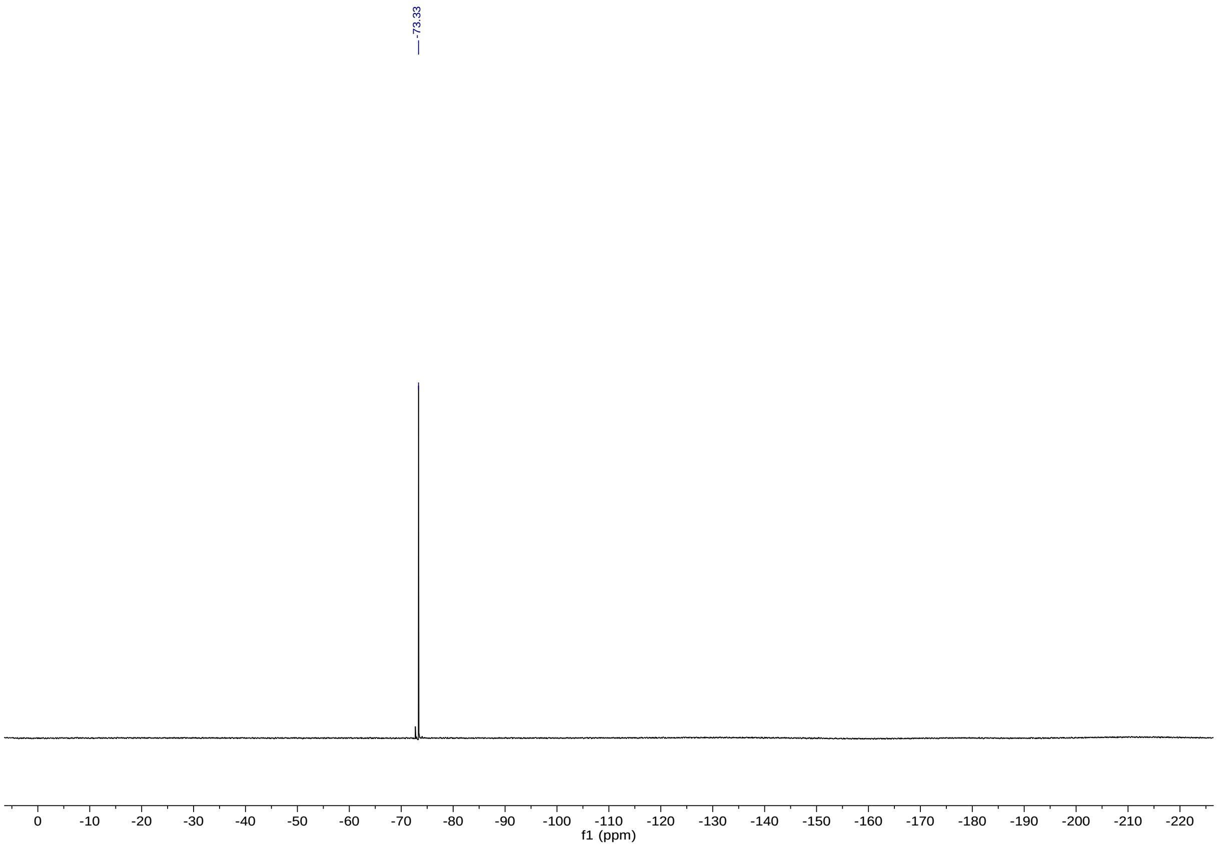

1H NMR: (400 MHz, $CDCl_3$) **22b**

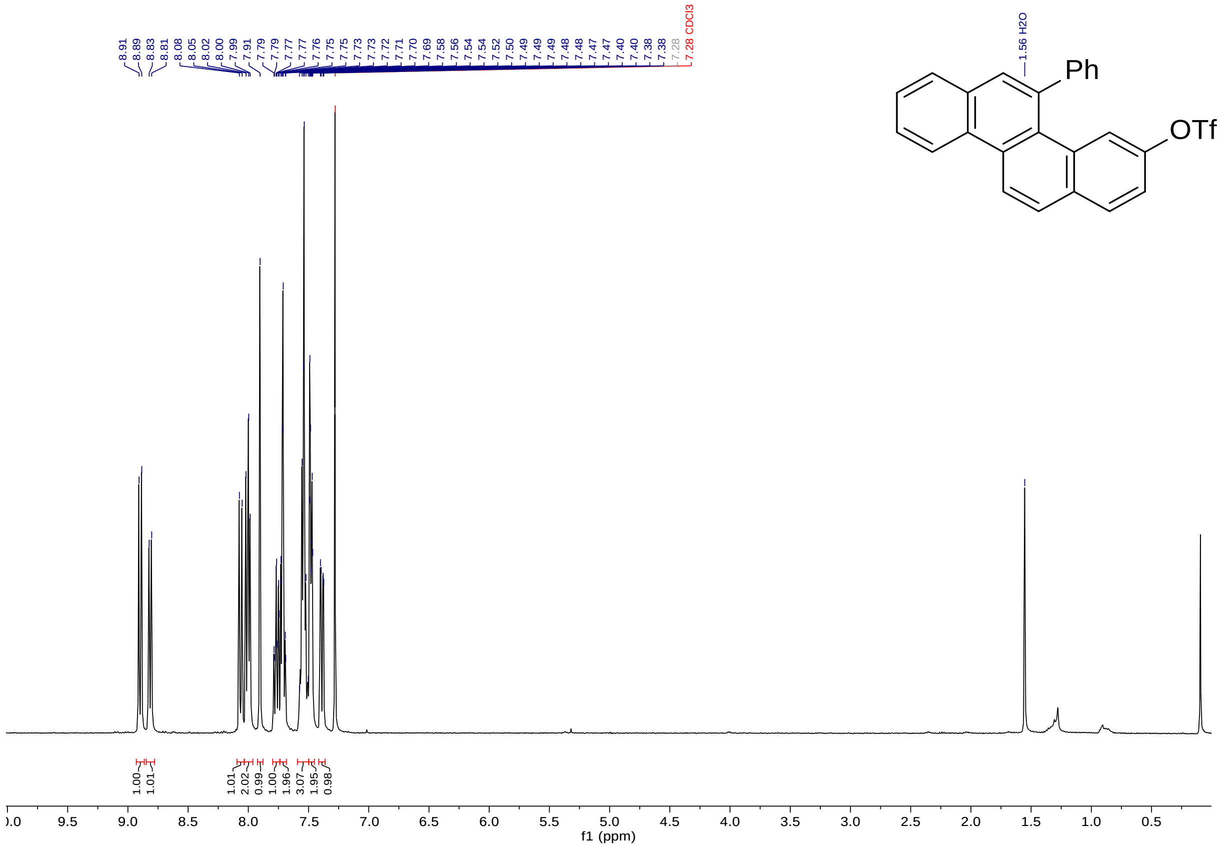

13C{H} NMR: (101 MHz, $CDCl_3$) **22b**

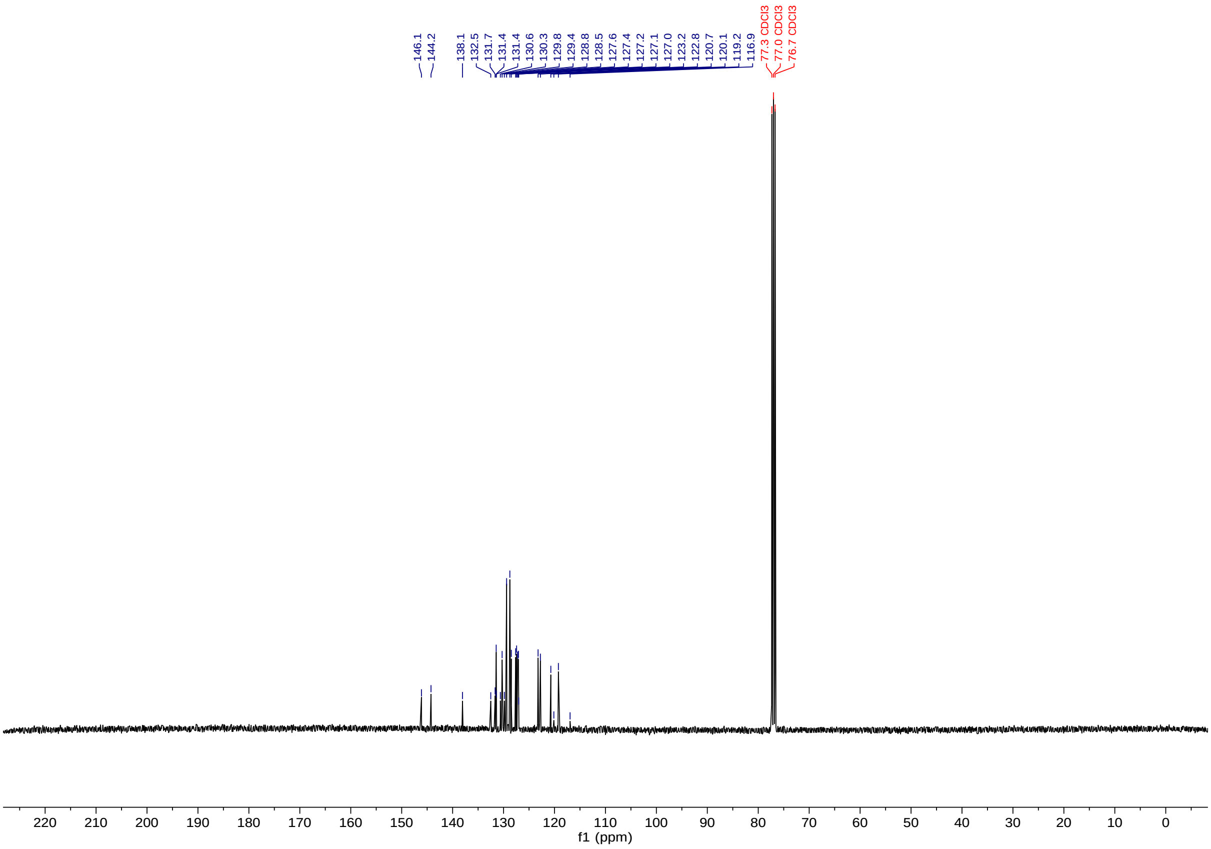

19F{H} NMR: (377 MHz, $CDCl_3$) **22b**

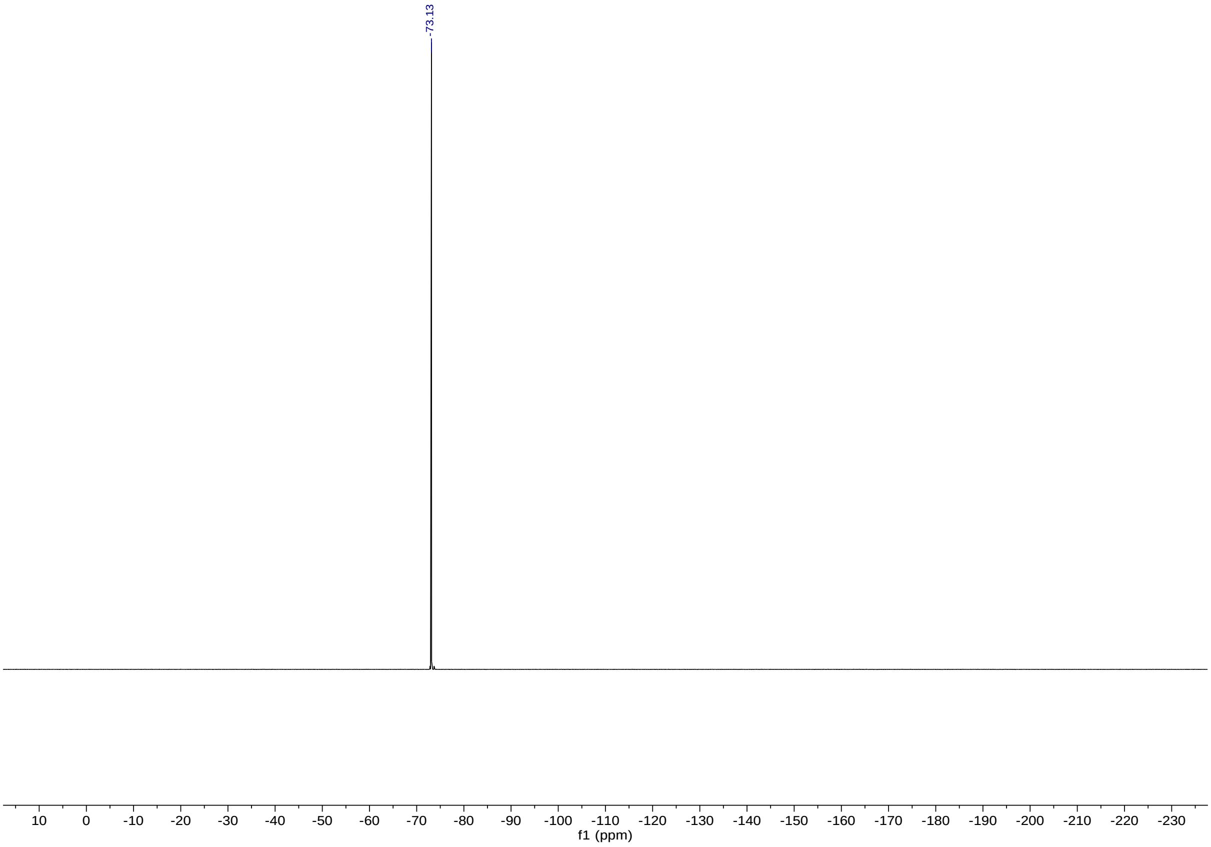

1H NMR: (300 MHz, $CDCl_3$) **8a**

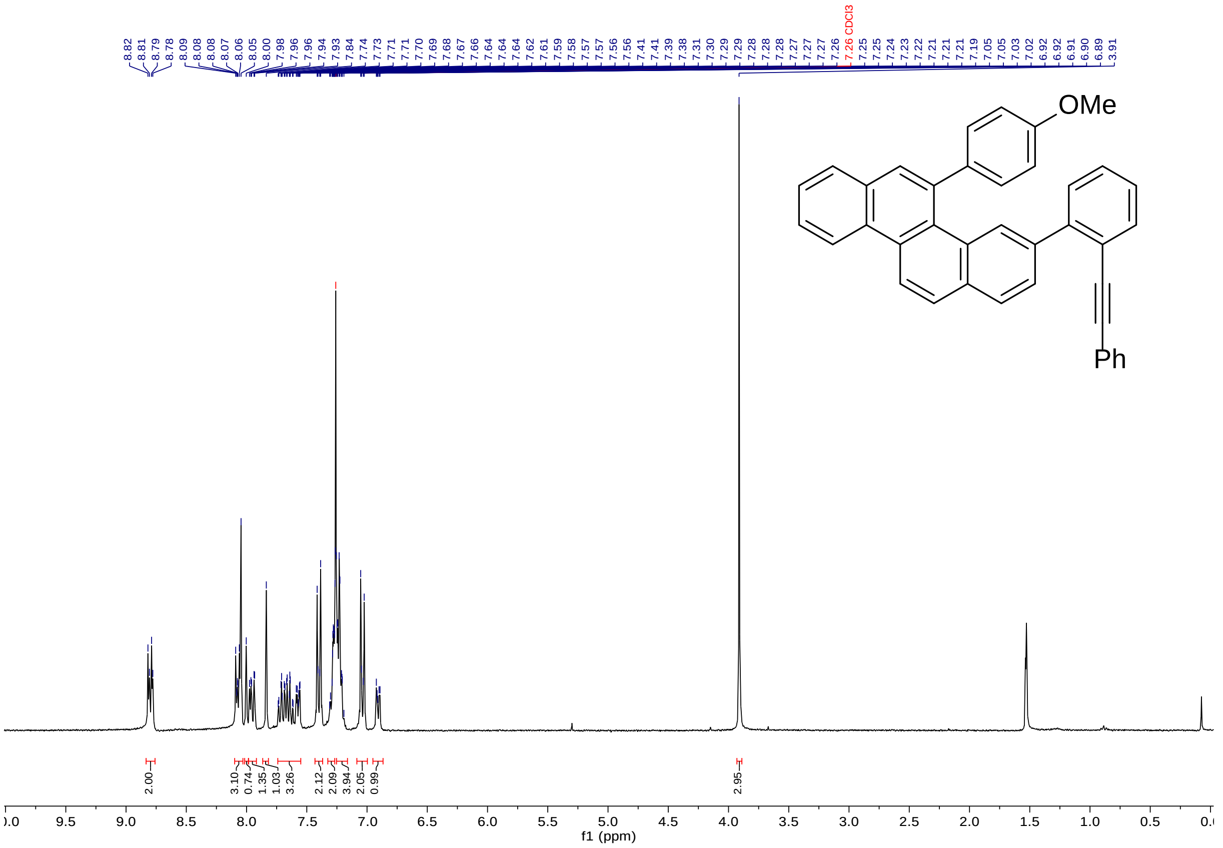

13C{H} NMR: (101 MHz, $CDCl_3$) **8a**

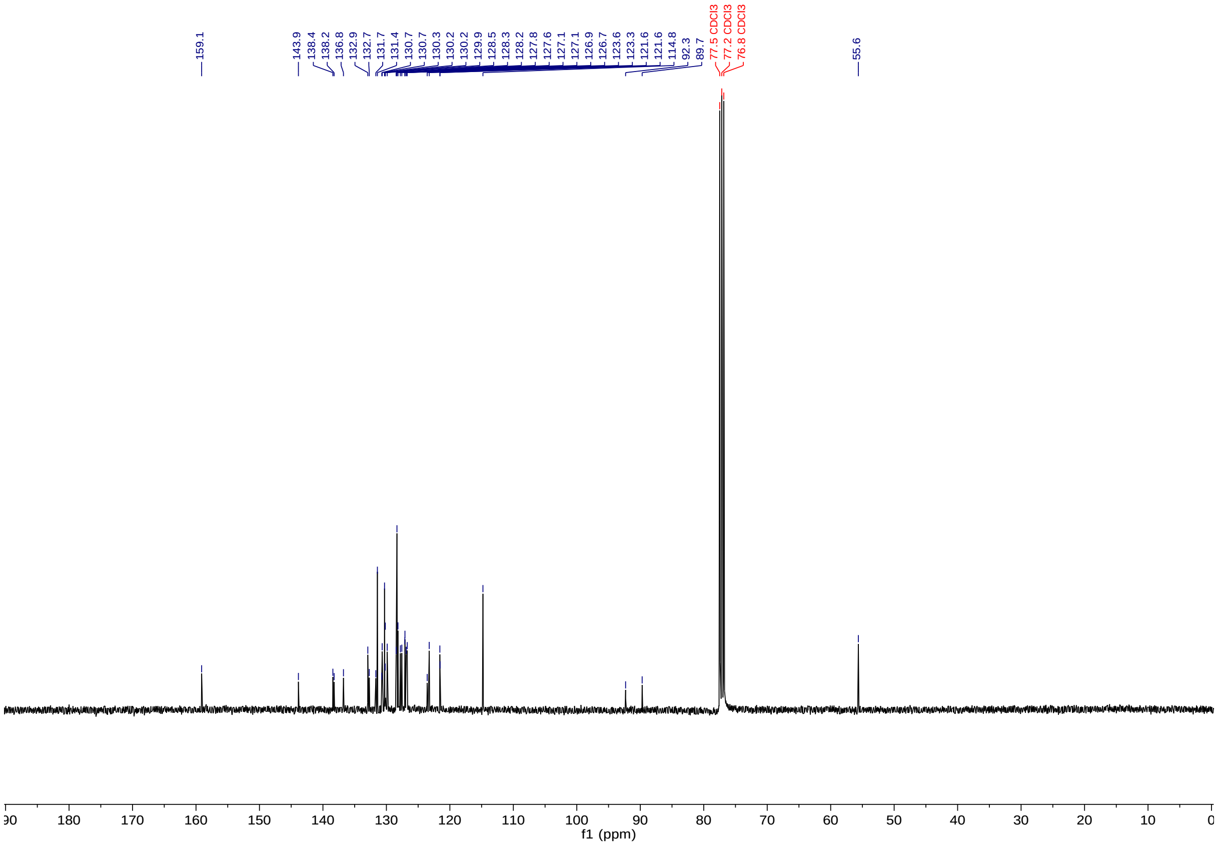

1H NMR: (400 MHz, $CDCl_3$) **8b**

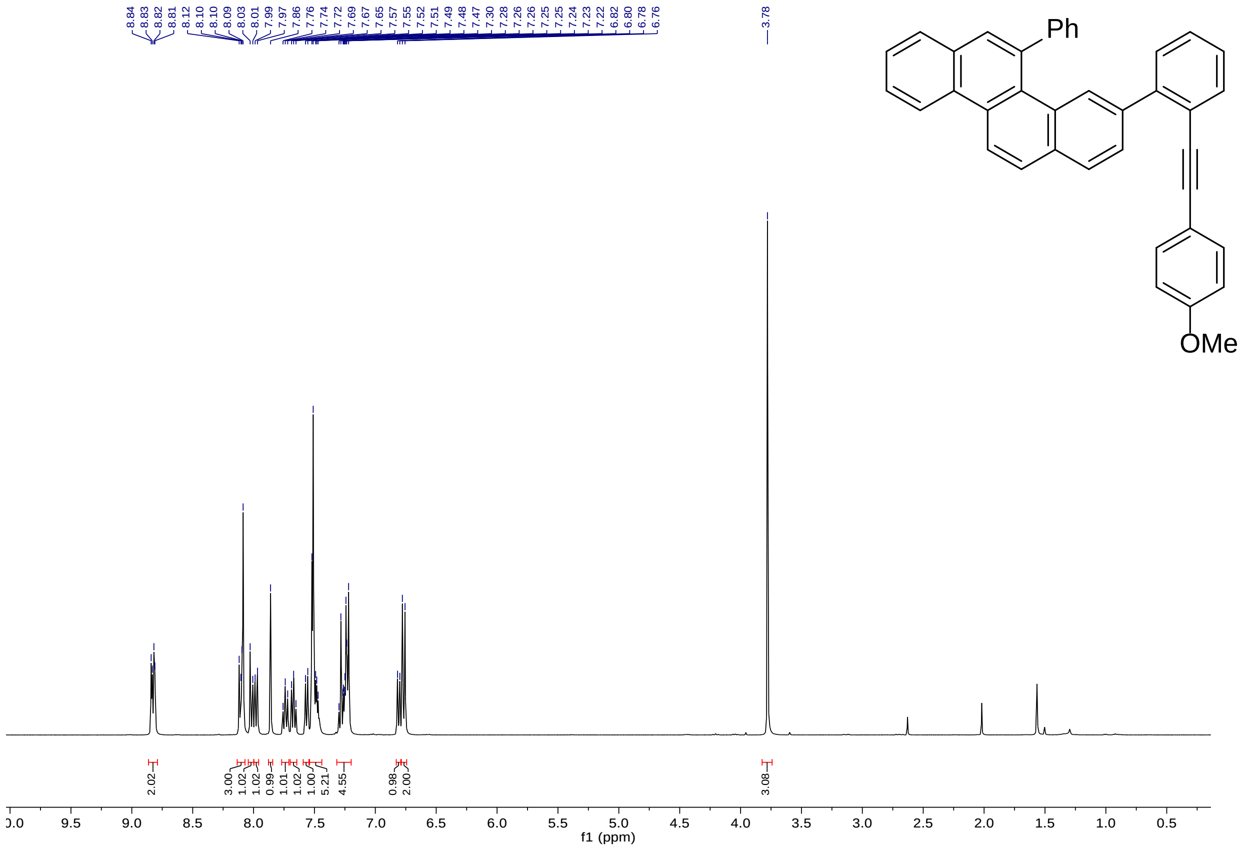

13C{H} NMR: (101 MHz, $CDCl_3$) **8b**

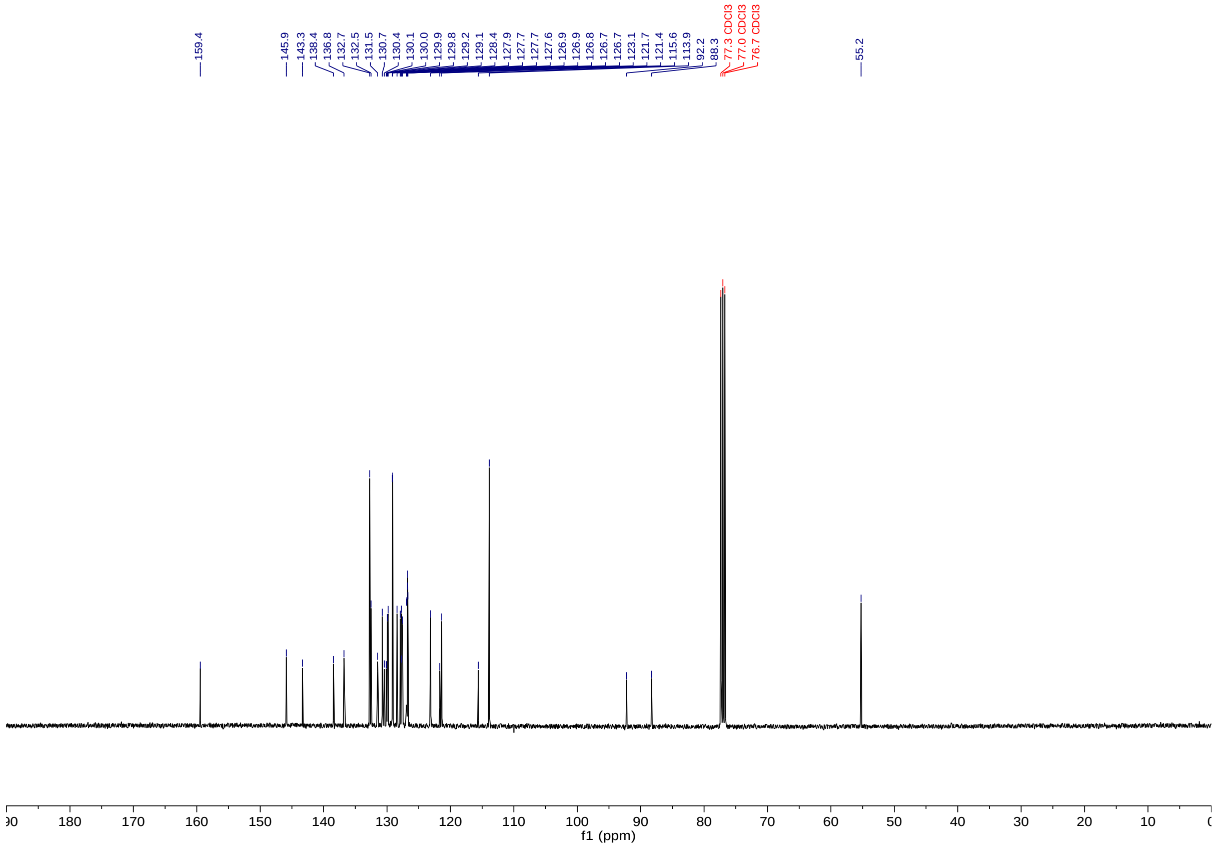

1H NMR: (500 MHz, $CDCl_3$) **1e**

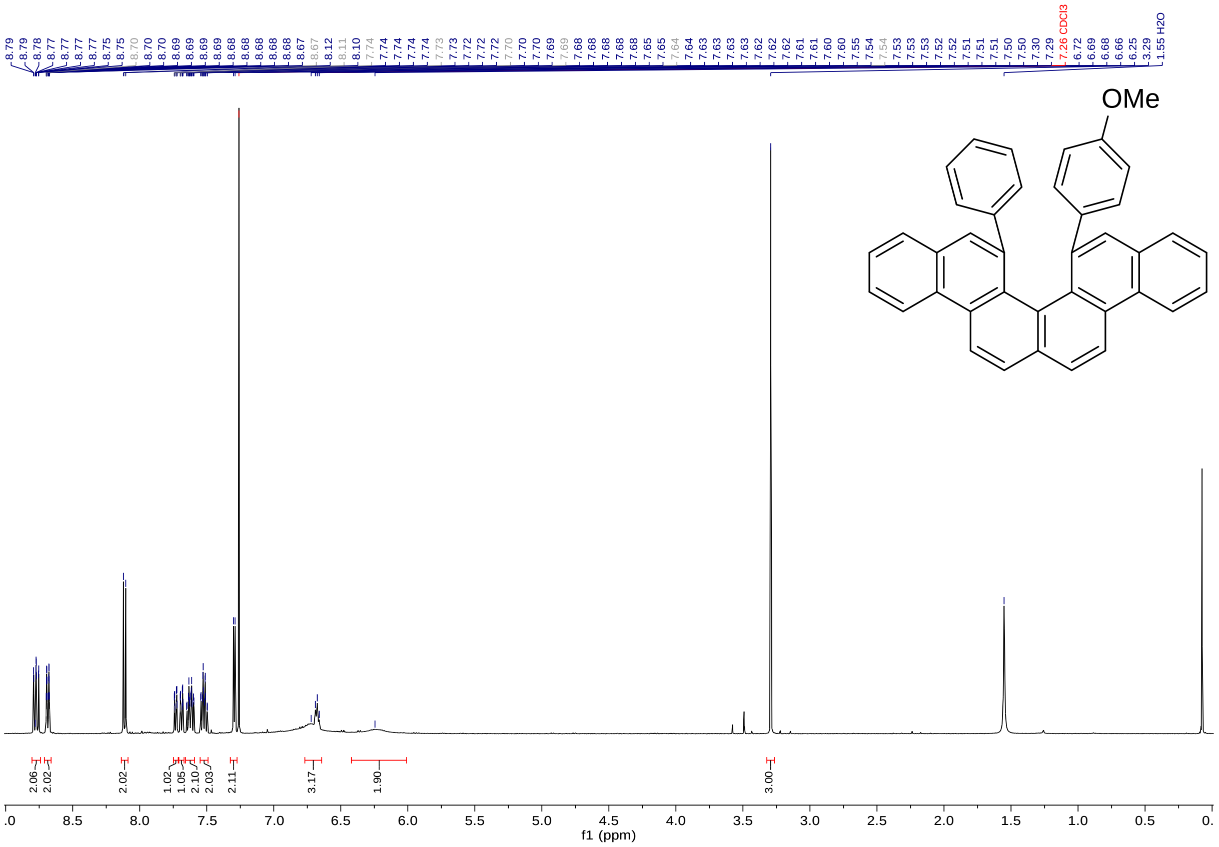

13C{H} NMR: (101 MHz, $CDCl_3$) **1e**

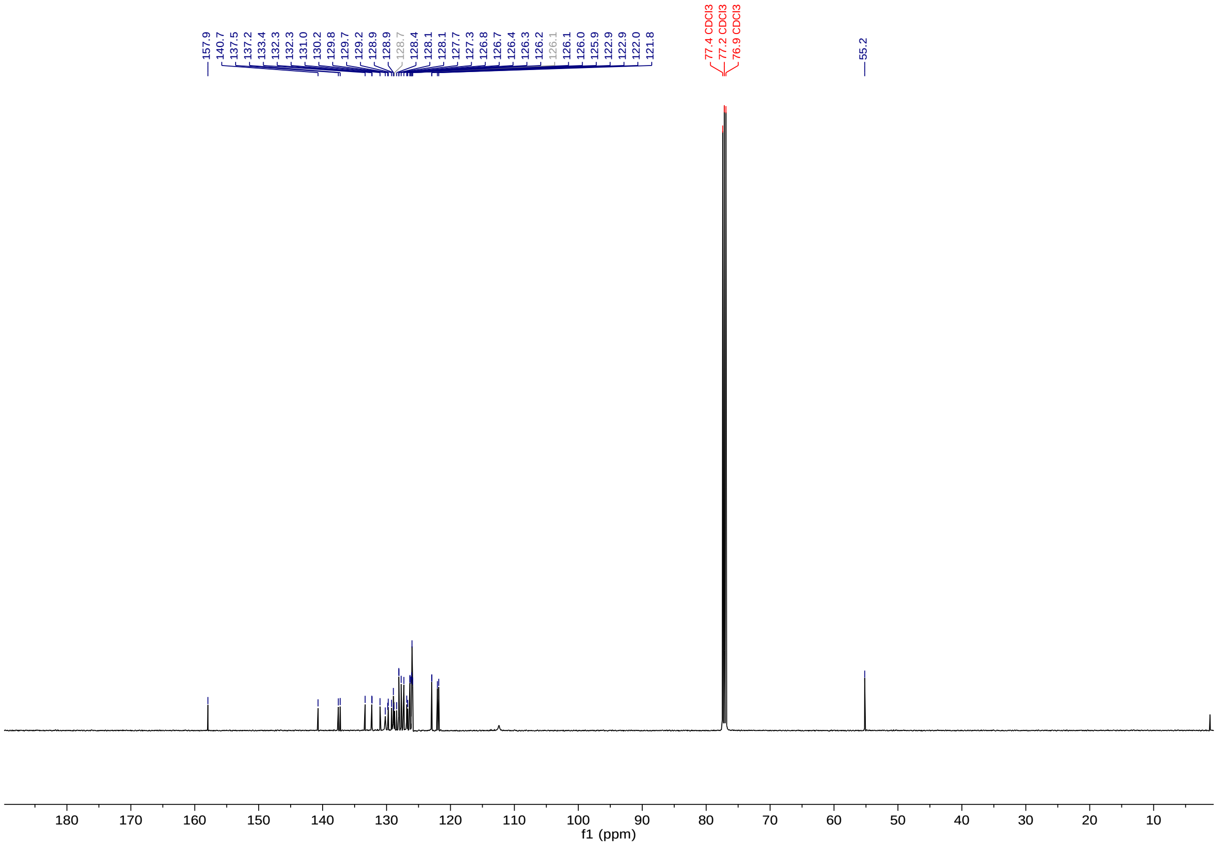

1H NMR: (300 MHz, $CDCl_3$) **12a**

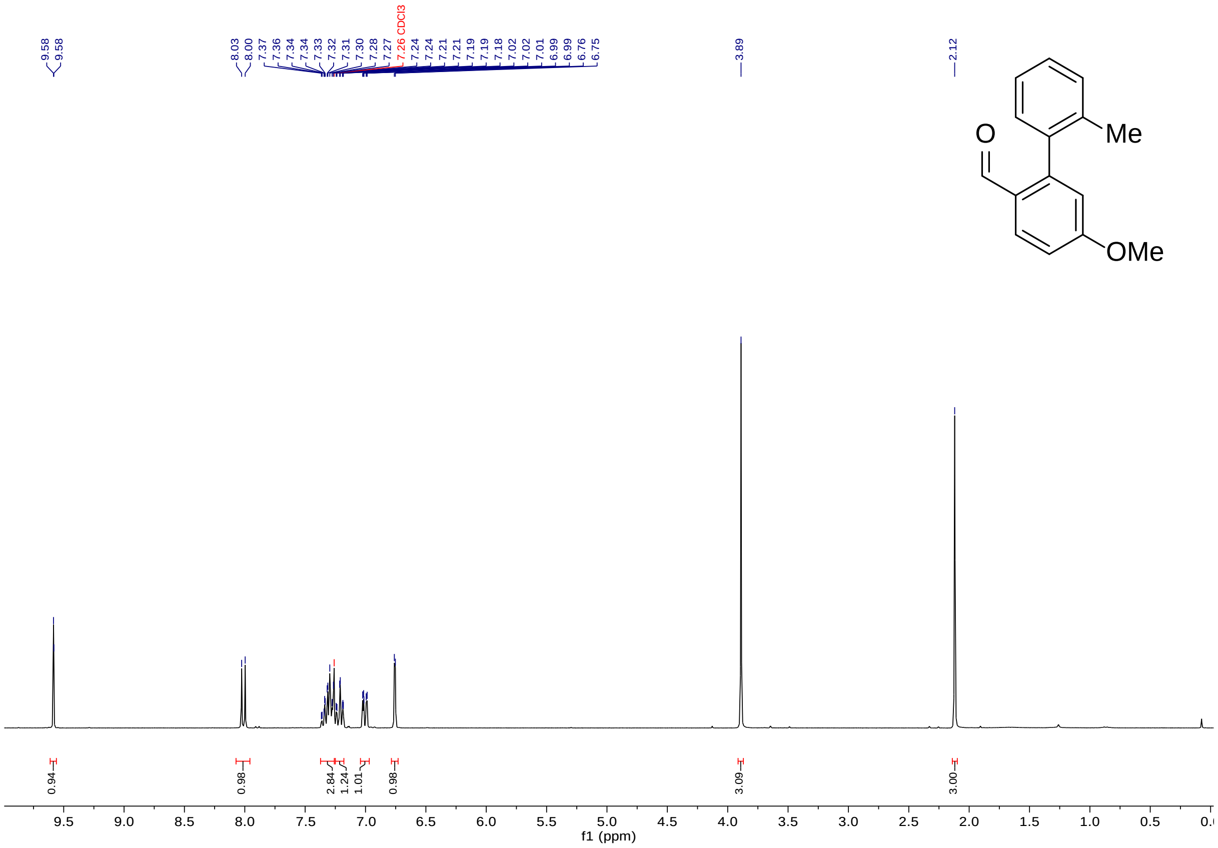

13C{H} NMR: (101 MHz, $CDCl_3$) **12a**

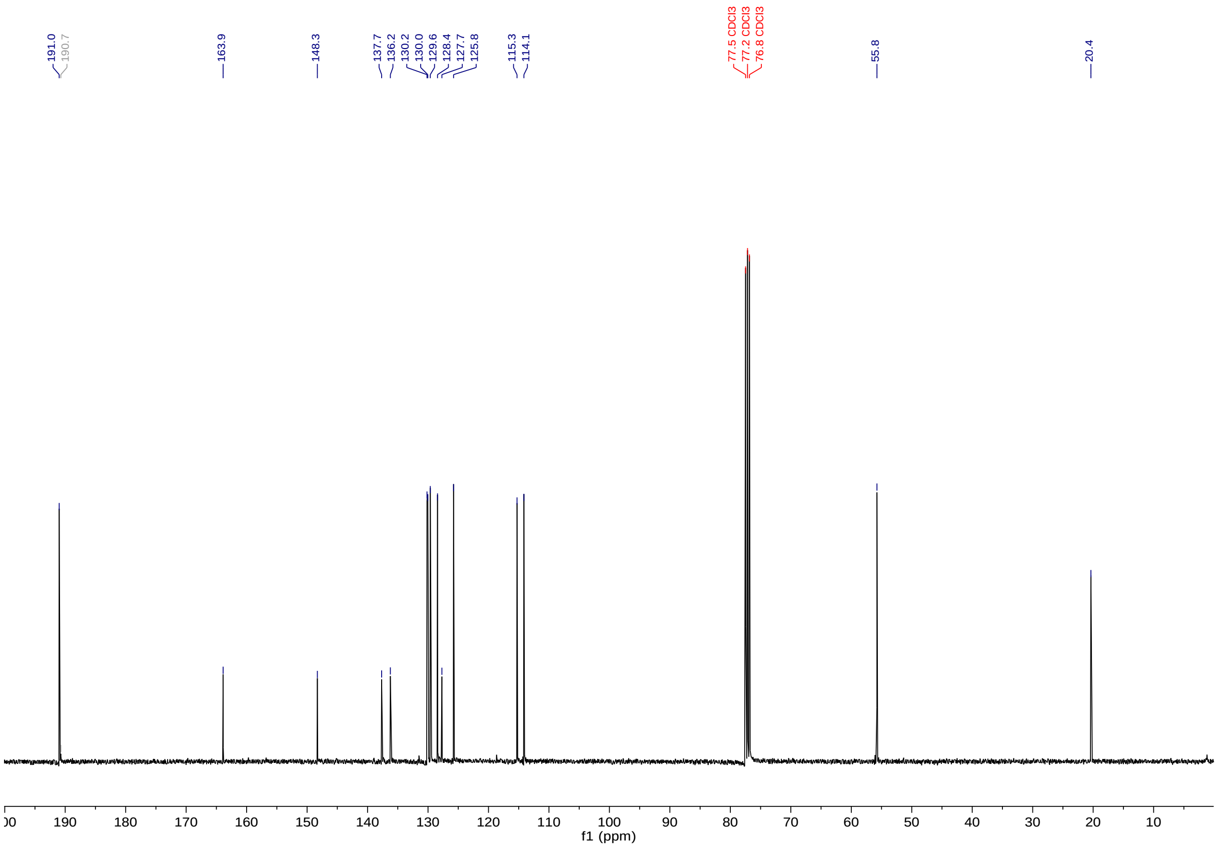

1H NMR: (300 MHz, $CDCl_3$) **12b**

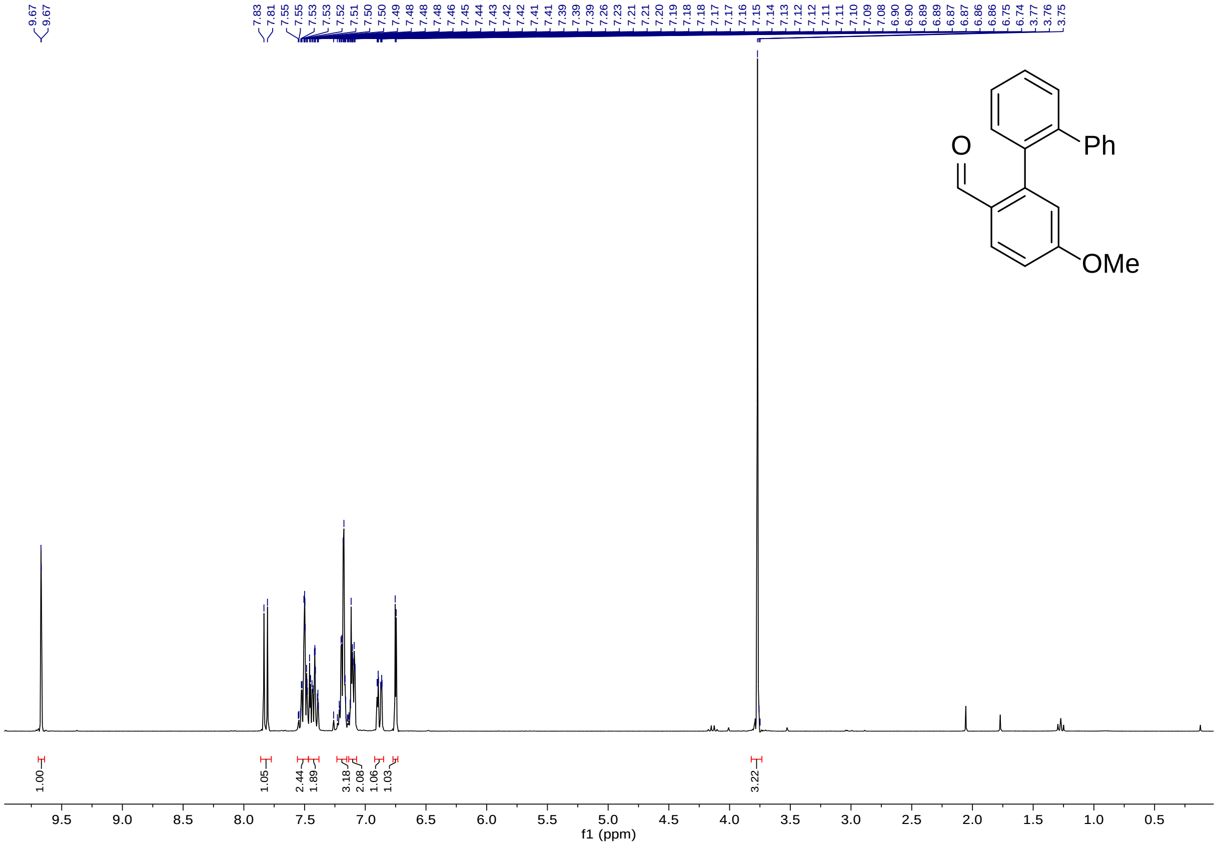

13C{H} NMR: (101 MHz, $CDCl_3$) **12b**

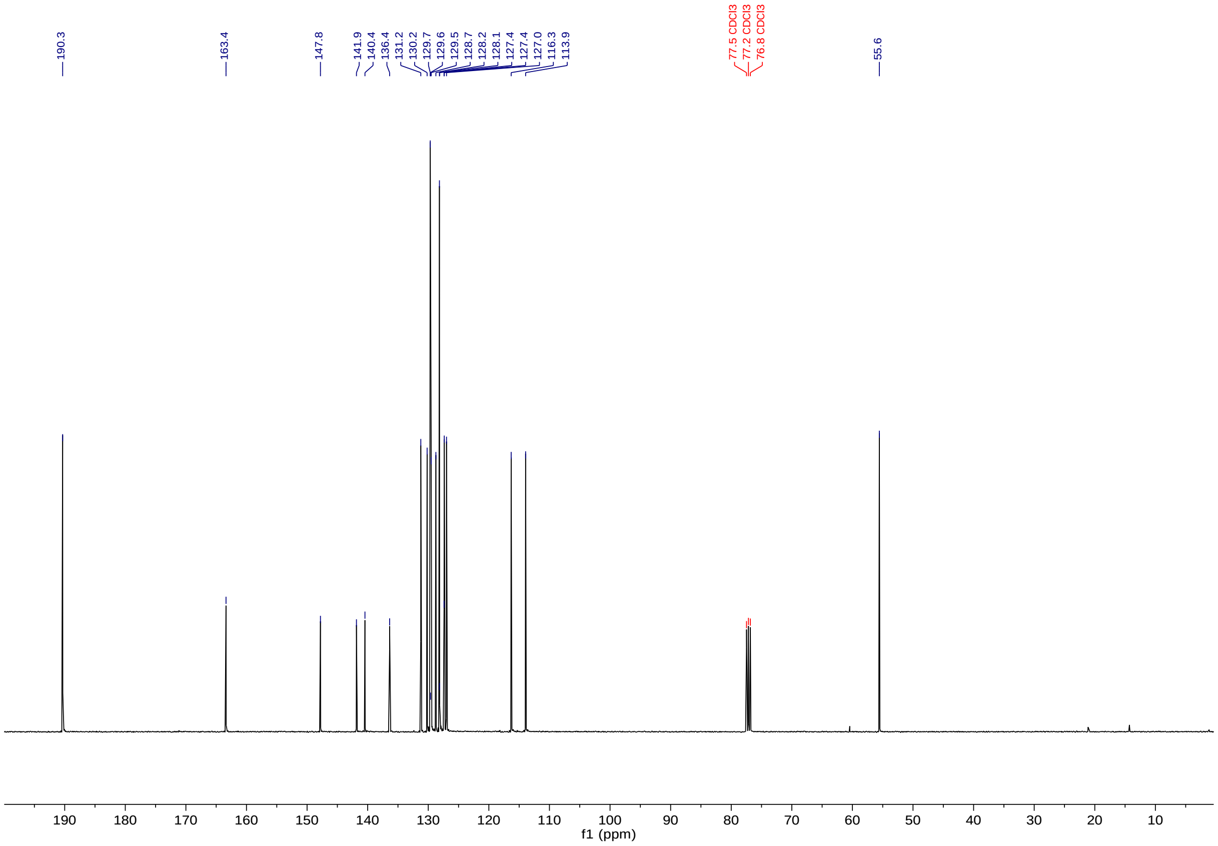

1H NMR: (300 MHz, $CDCl_3$) **12c**

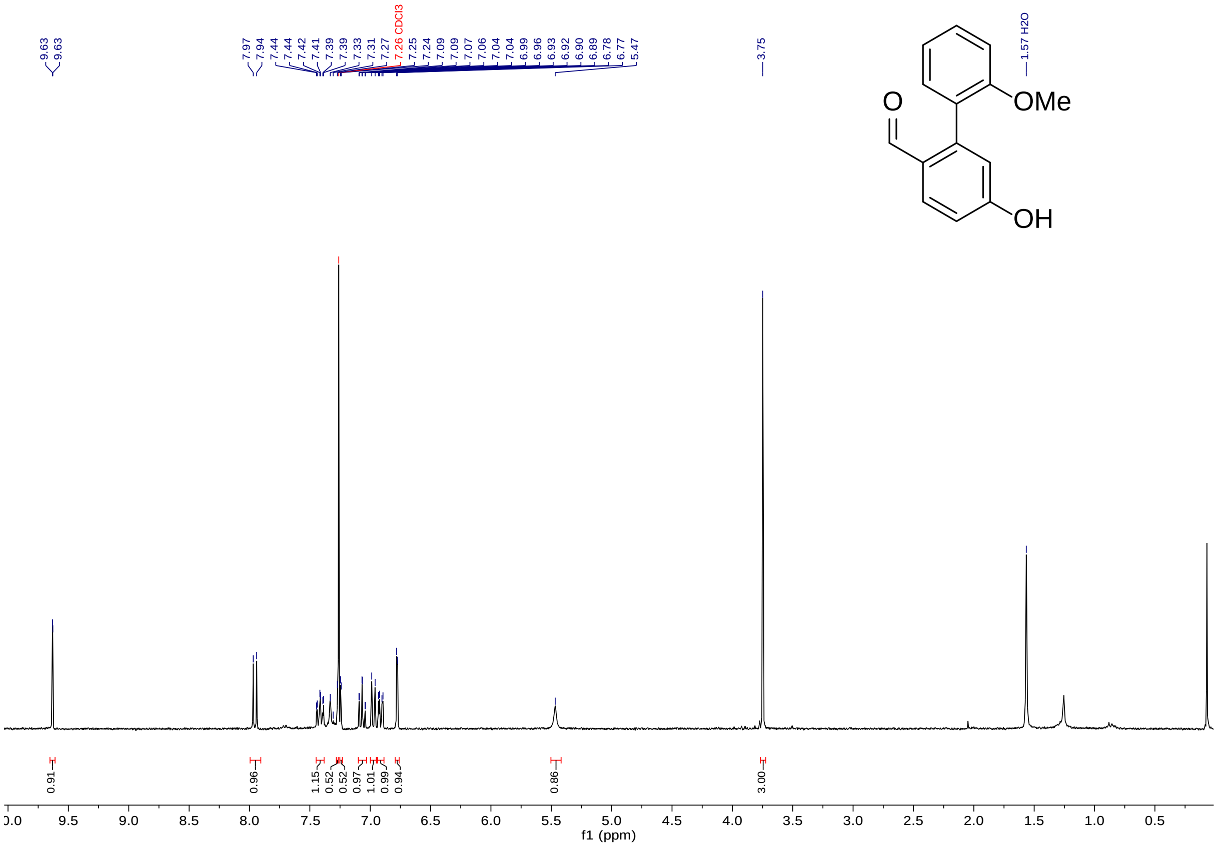

13C{H} NMR: (101 MHz, $CDCl_3$) **12c**

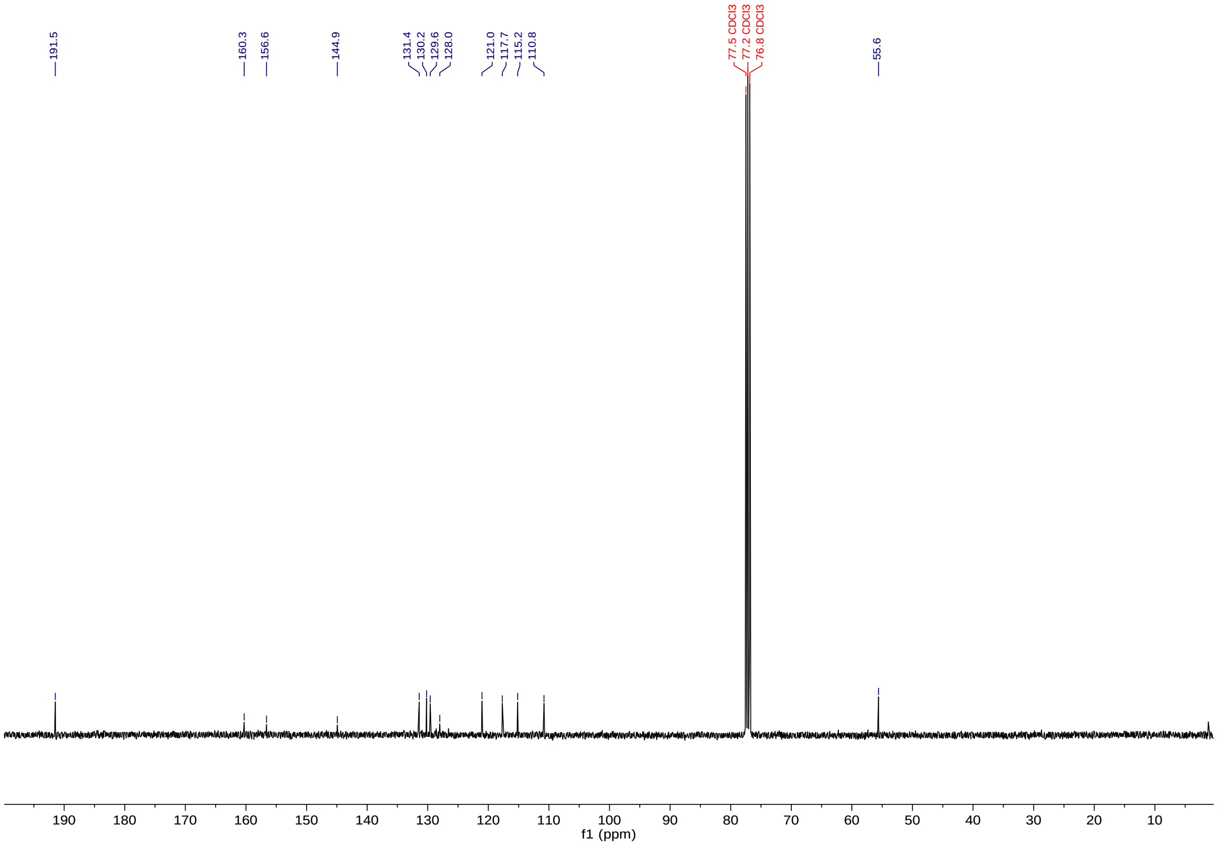

1H NMR: (300 MHz, $CDCl_3$) **12c**

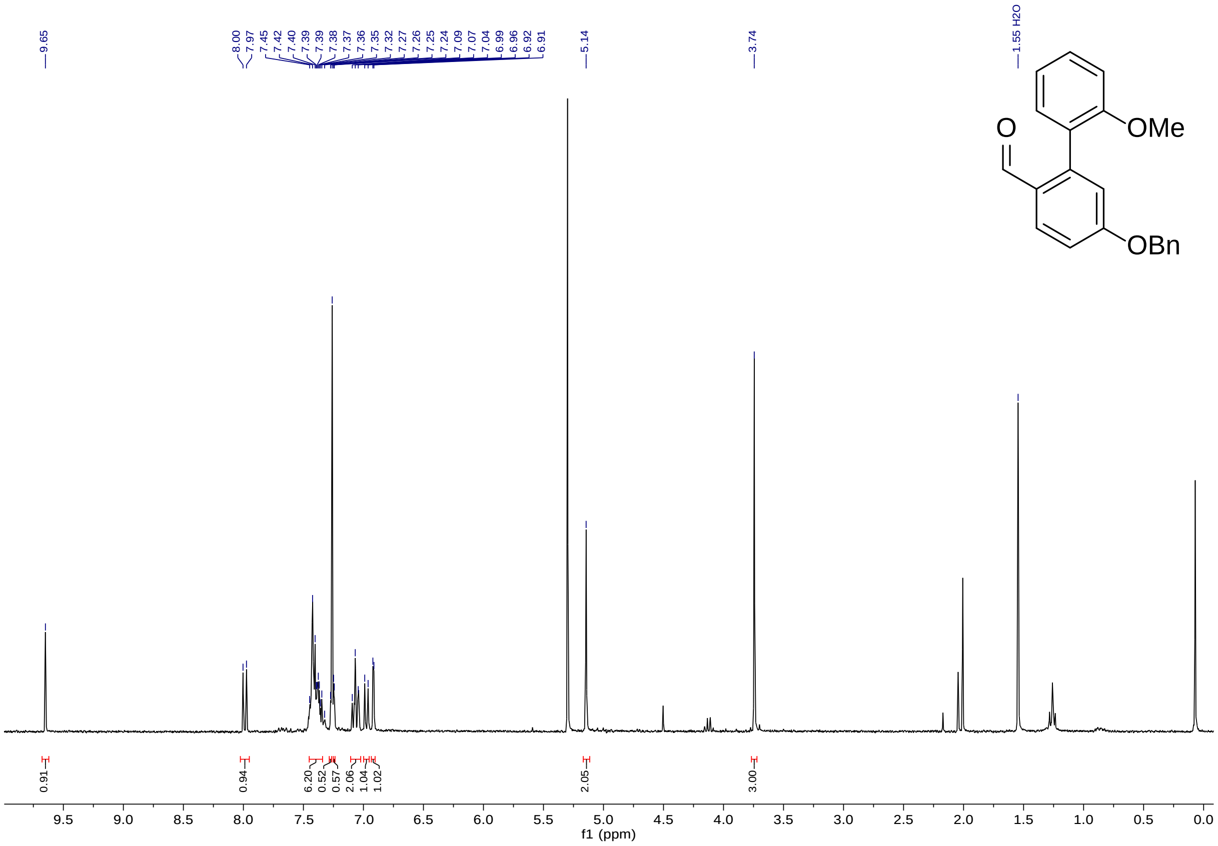

13C{H} NMR: (101 MHz, $CDCl_3$) **12c**

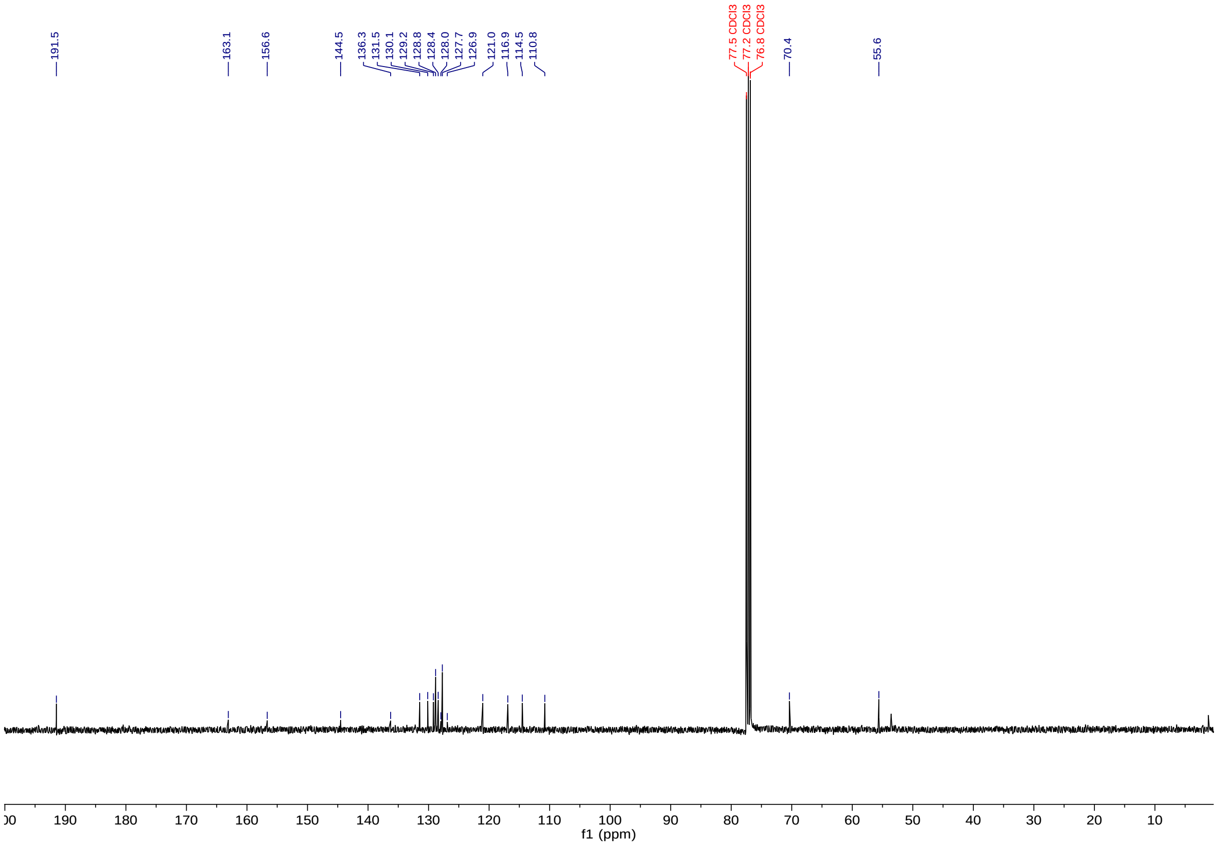

1H NMR: (300 MHz, $CDCl_3$) **13a**

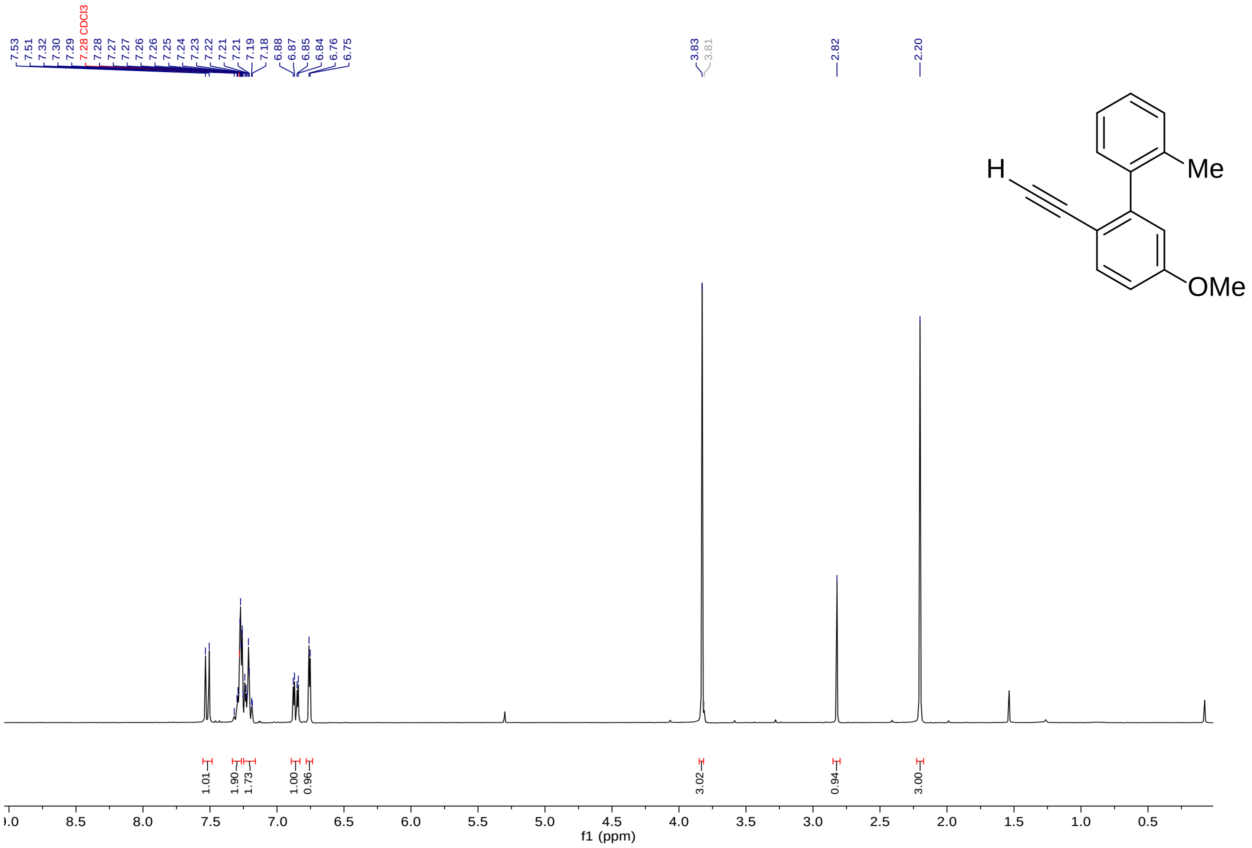

13C{H} NMR: (101 MHz, $CDCl_3$) **13a**

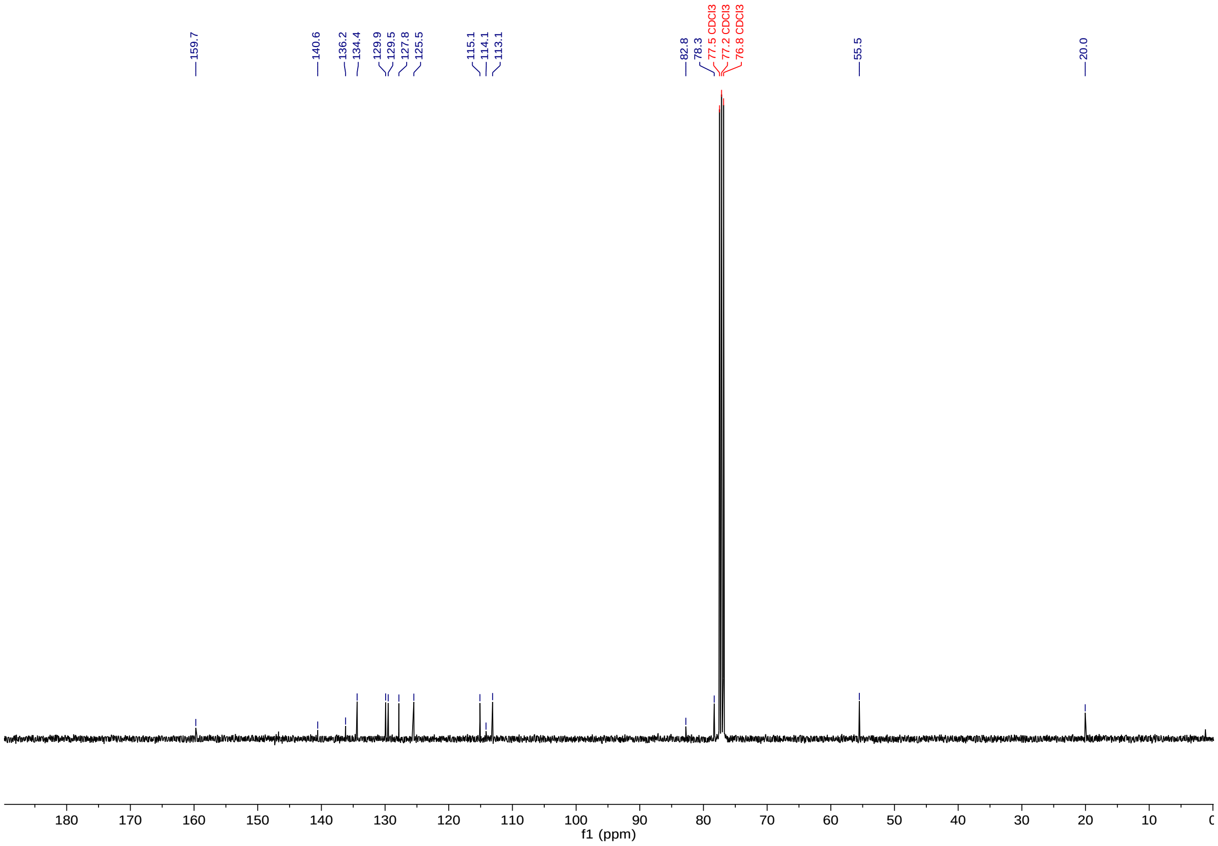

1H NMR: (400 MHz, $CDCl_3$) **13b**

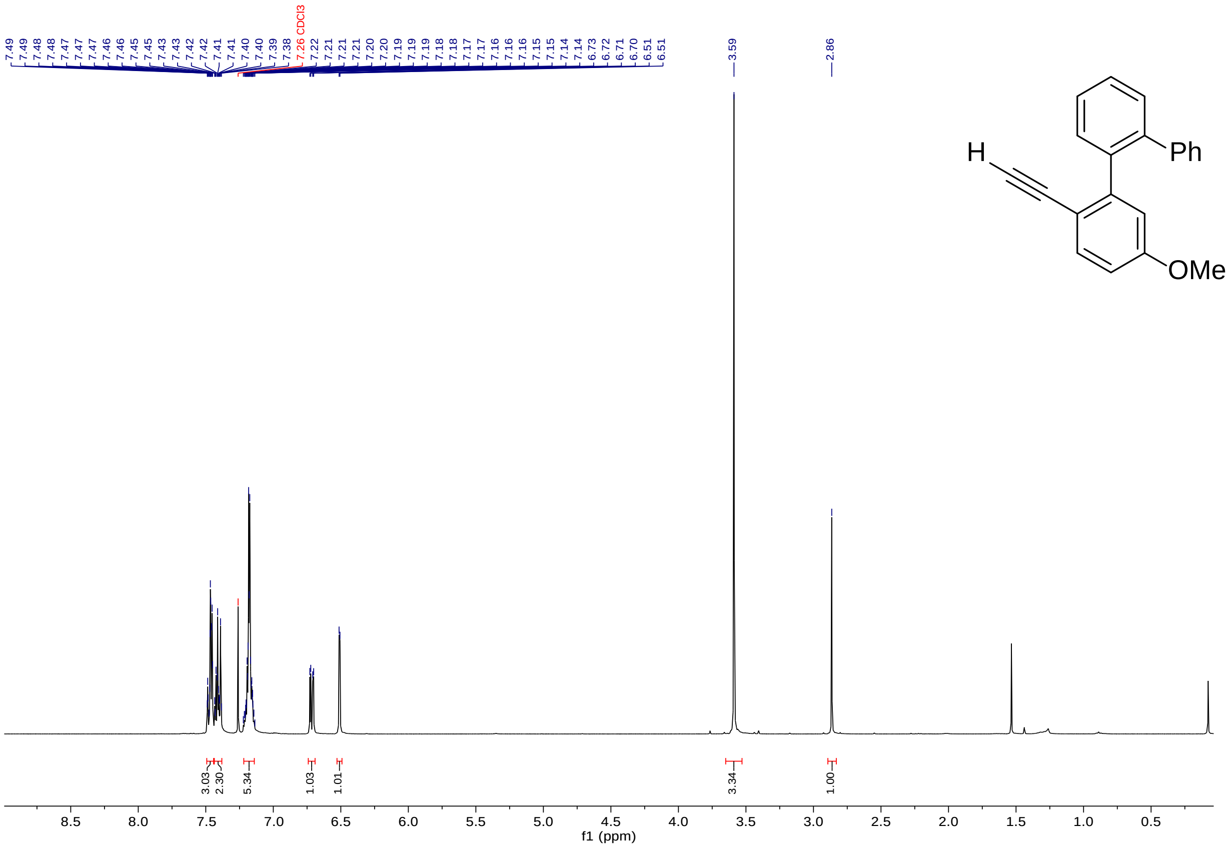

13C{H} NMR: (101 MHz, $CDCl_3$) **13b**

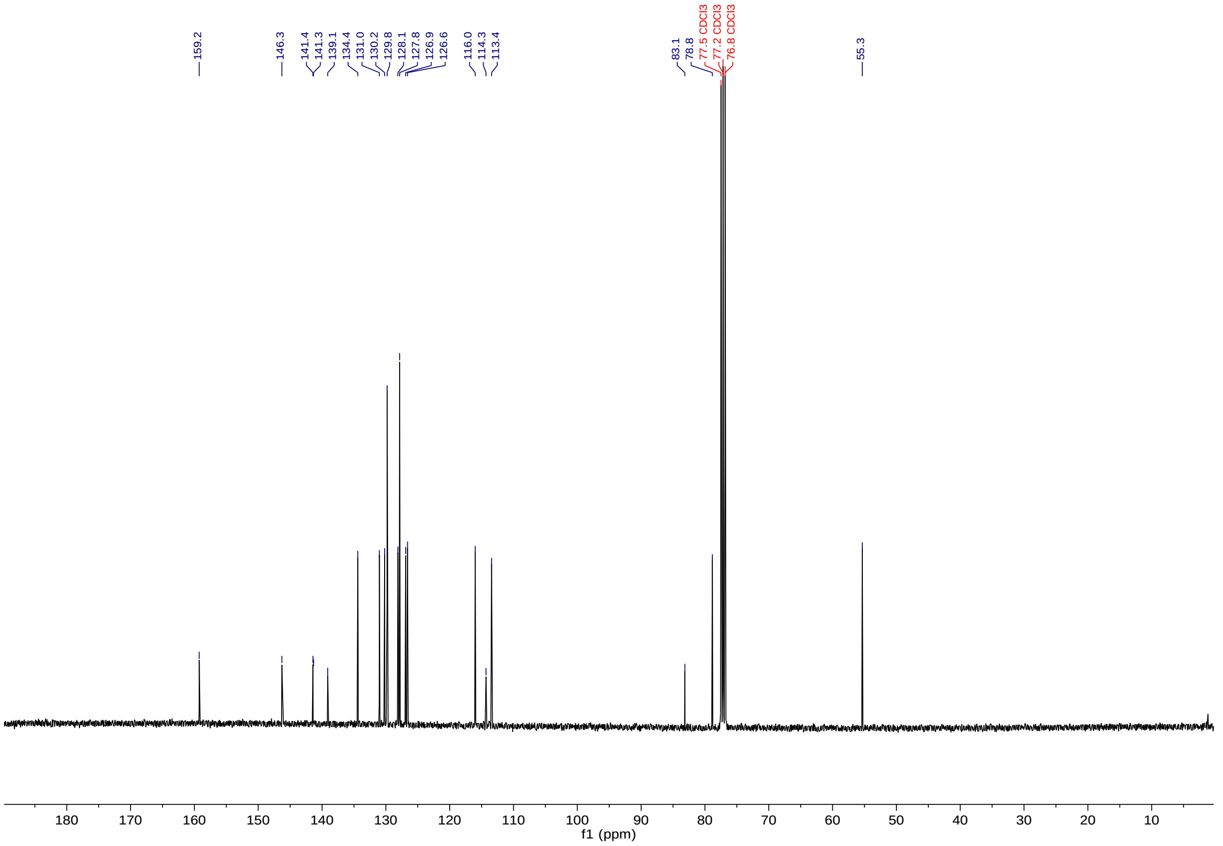

1H NMR: (400 MHz, $CDCl_3$) **13c**

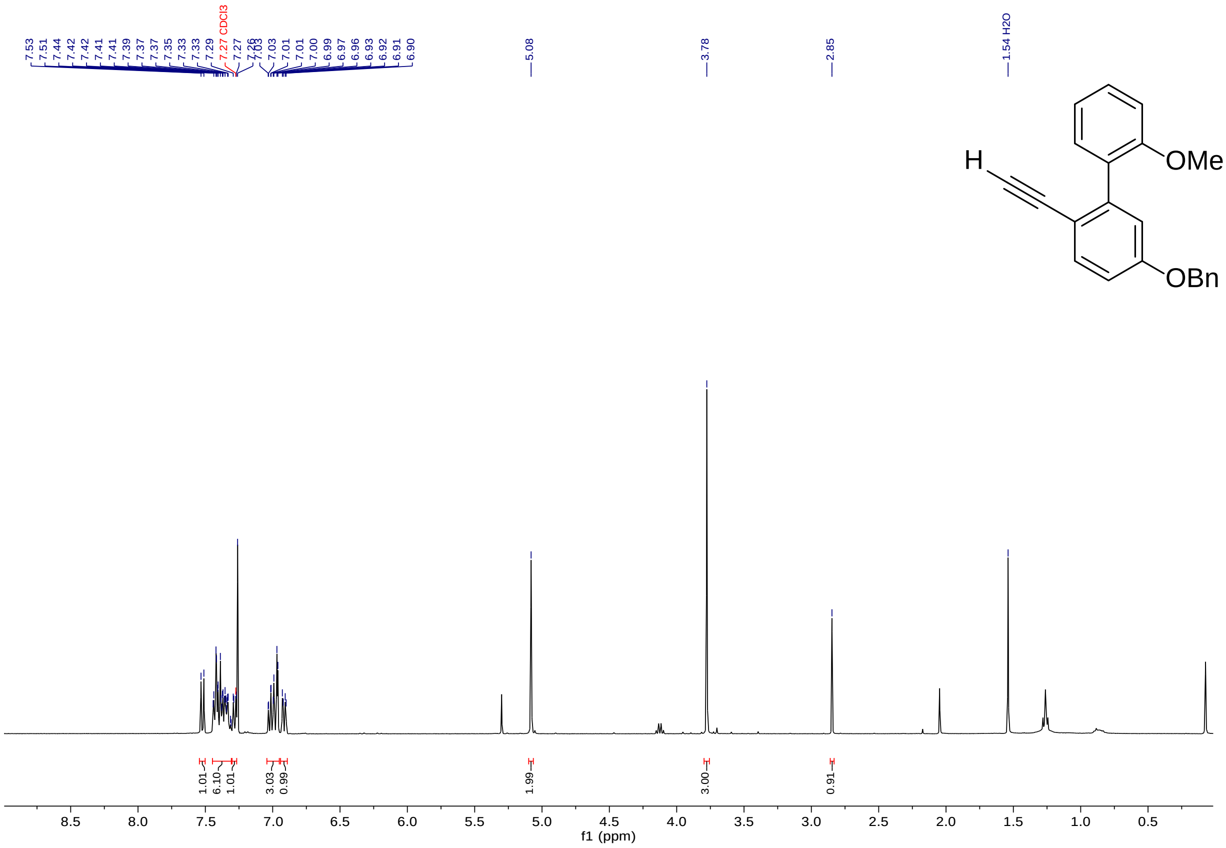

13C{H} NMR: (101 MHz, $CDCl_3$) **13c**

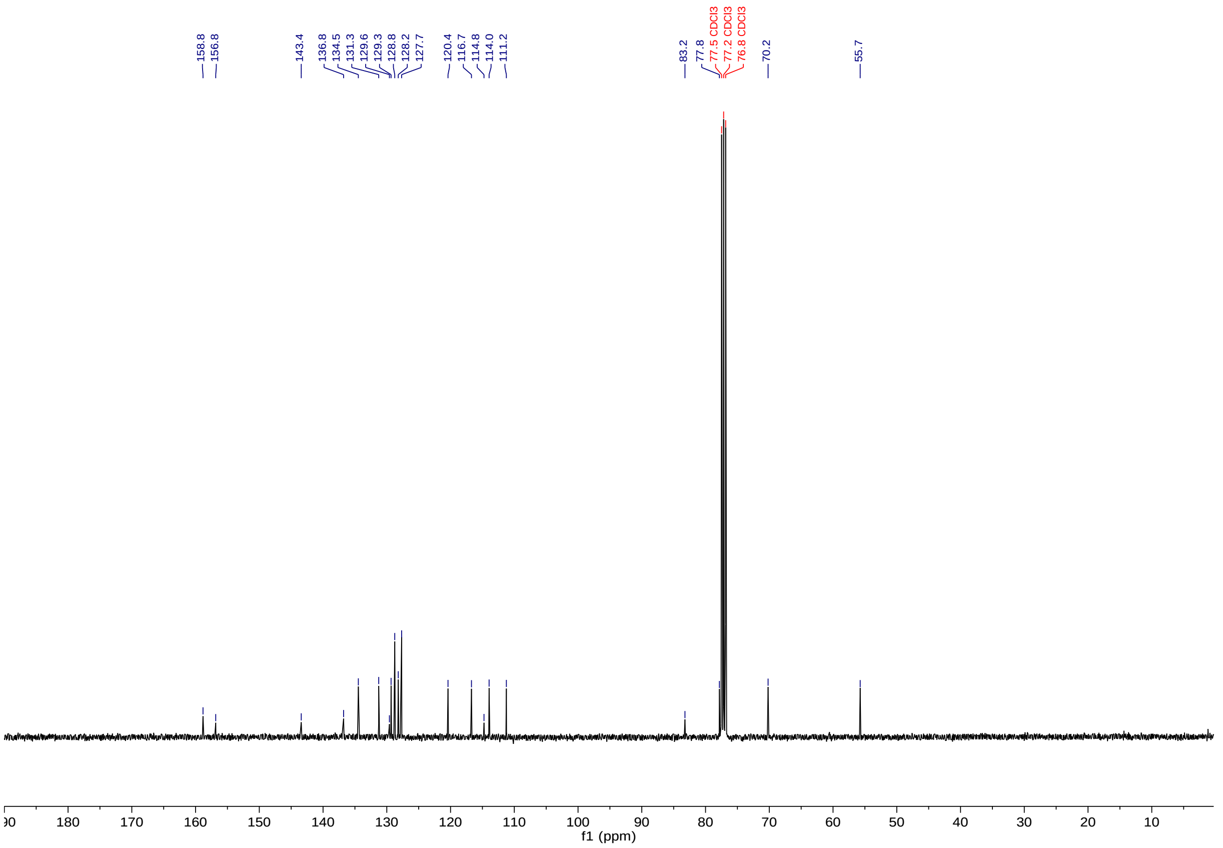

1H NMR: (400 MHz, $CDCl_3$) **14c**

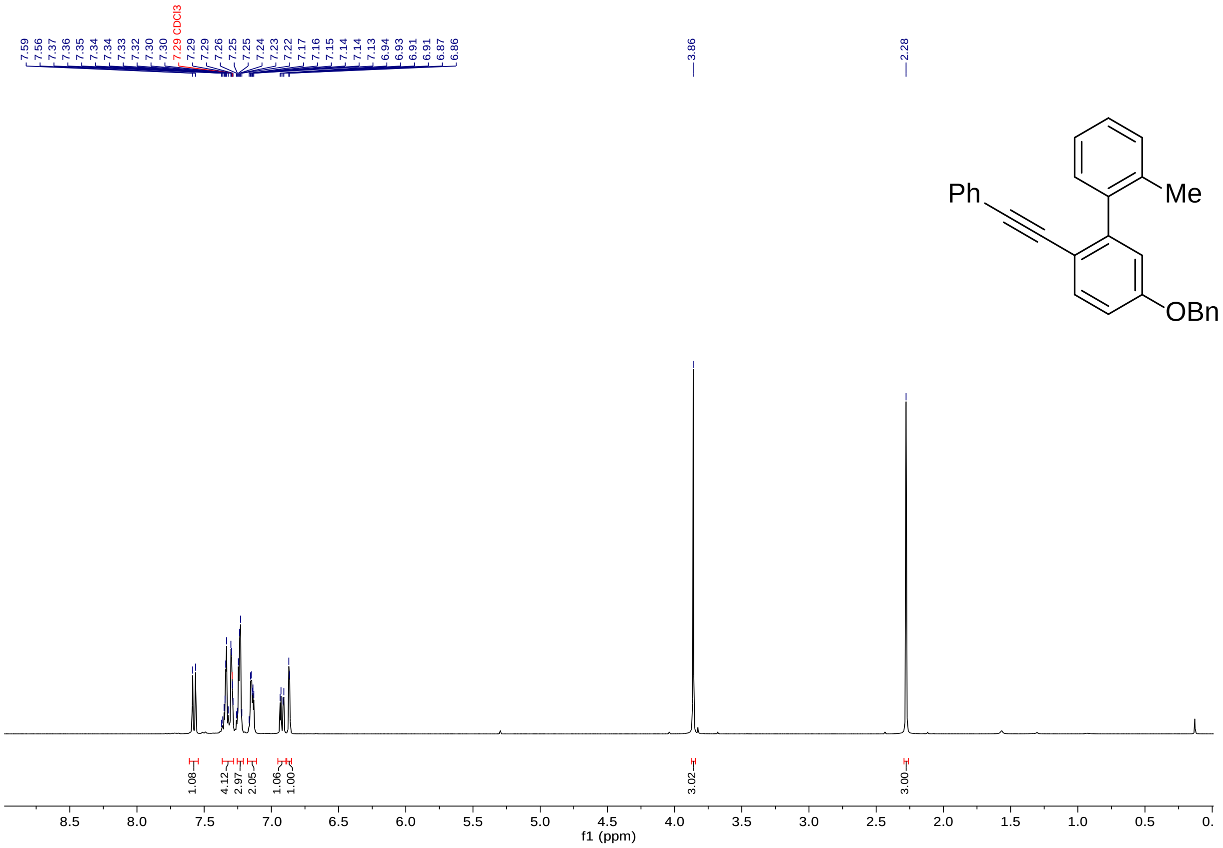

13C{H} NMR: (101 MHz, $CDCl_3$) **14c**

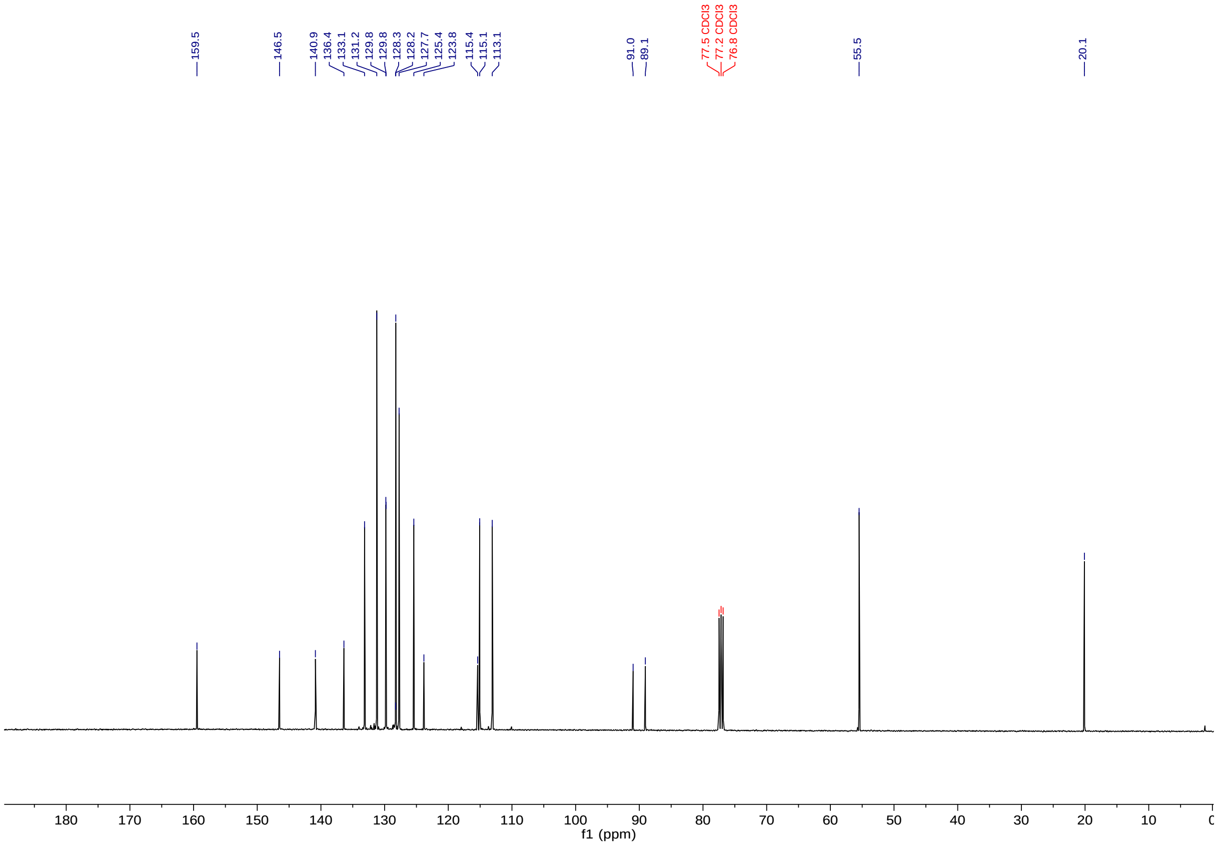

1H NMR: (400 MHz, $CDCl_3$) **14d**

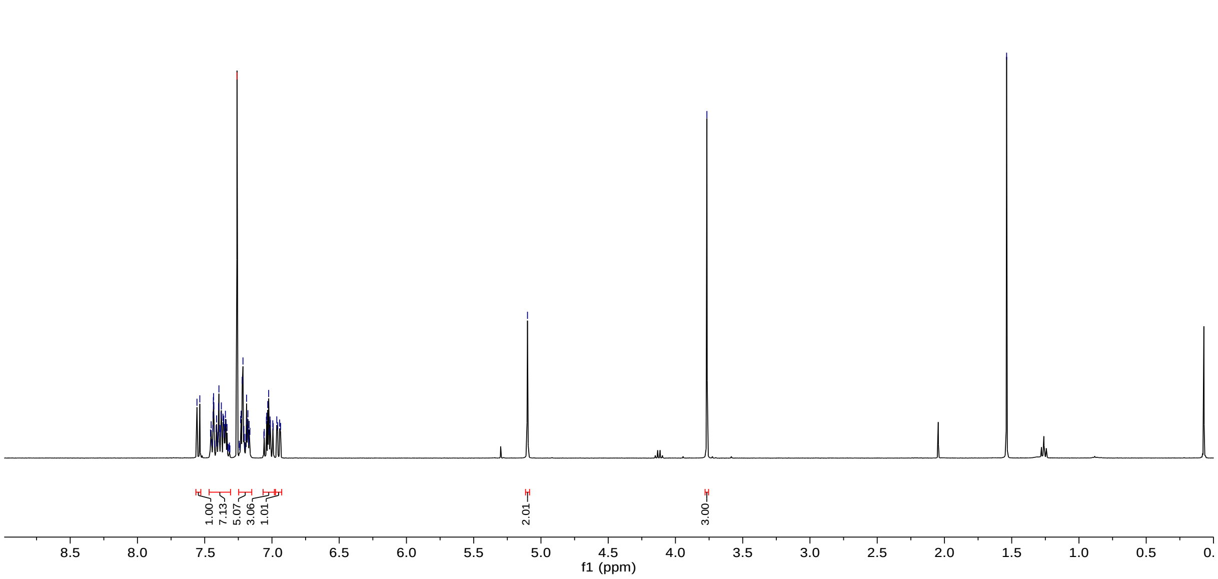

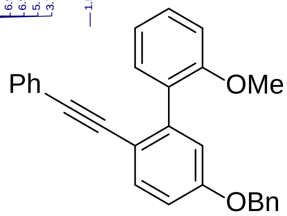

13C{H} NMR: (101 MHz, $CDCl_3$) **14d**

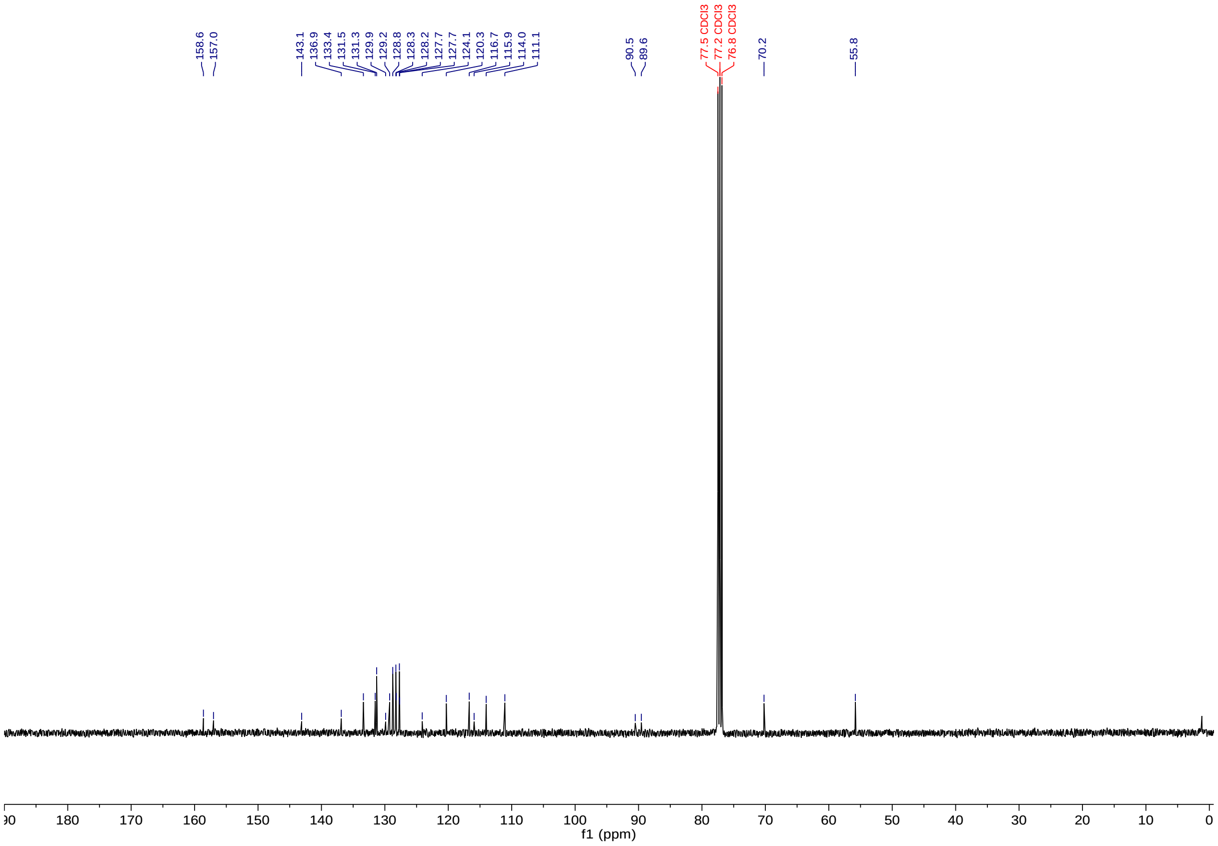

1H NMR: (300 MHz, $CDCl_3$) **15a**

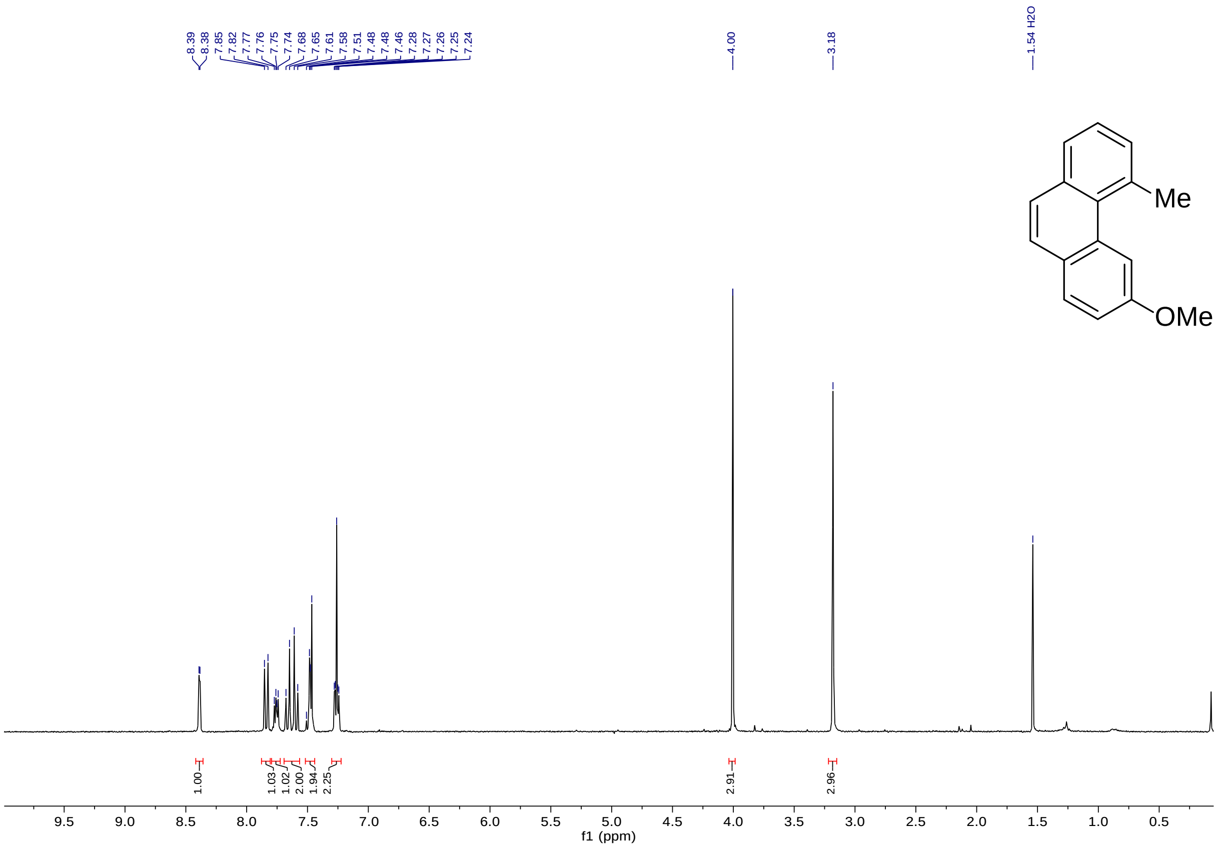

13C{H} NMR: (101 MHz, $CDCl_3$) **15a**

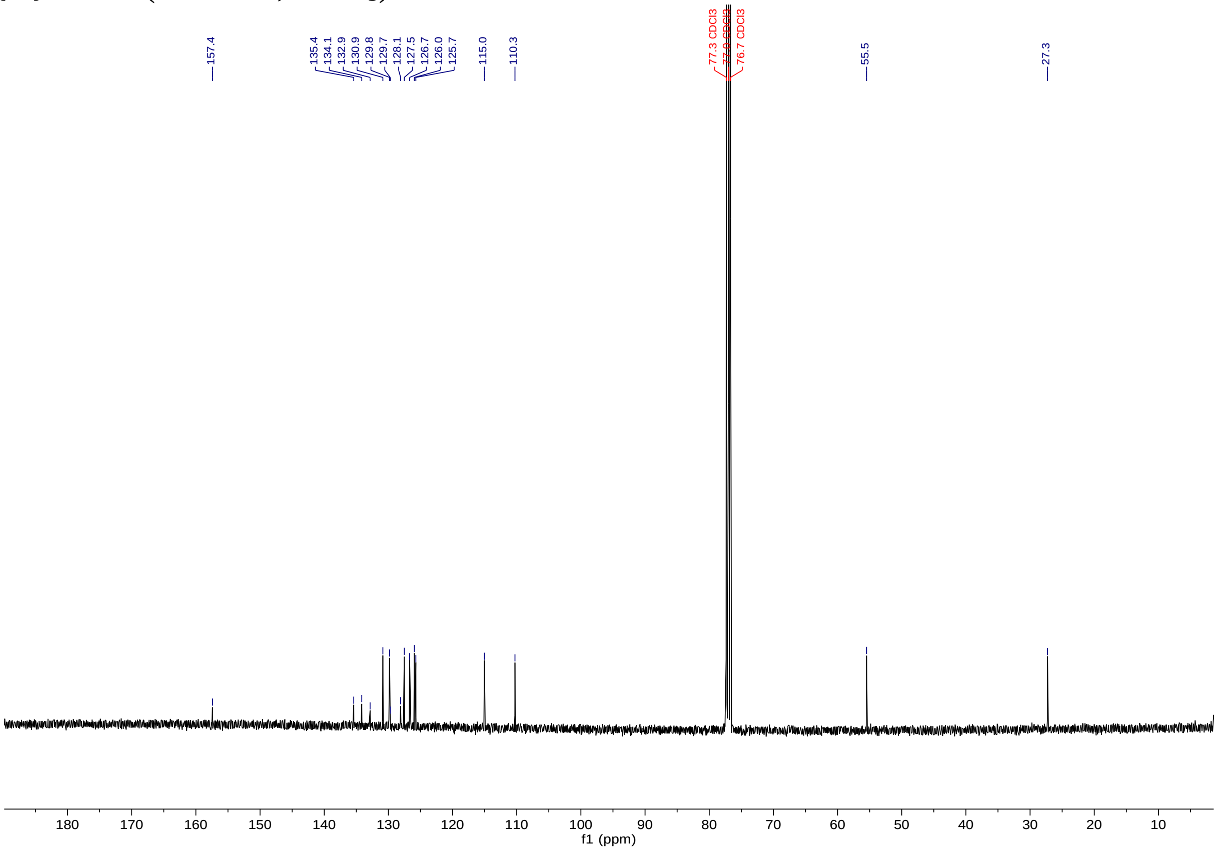

1H NMR: (400 MHz, $CDCl_3$) **15b**

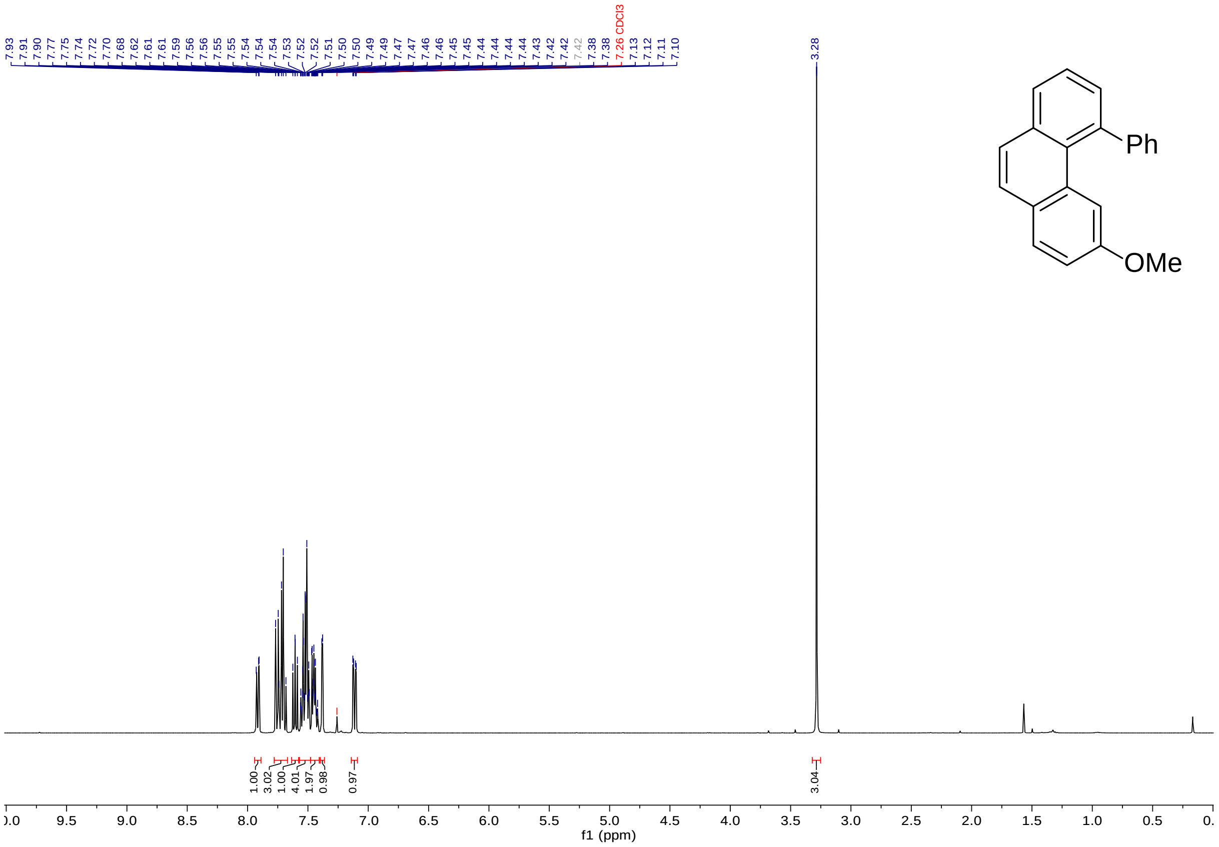

13C{H} NMR: (101 MHz, $CDCl_3$) **15b**

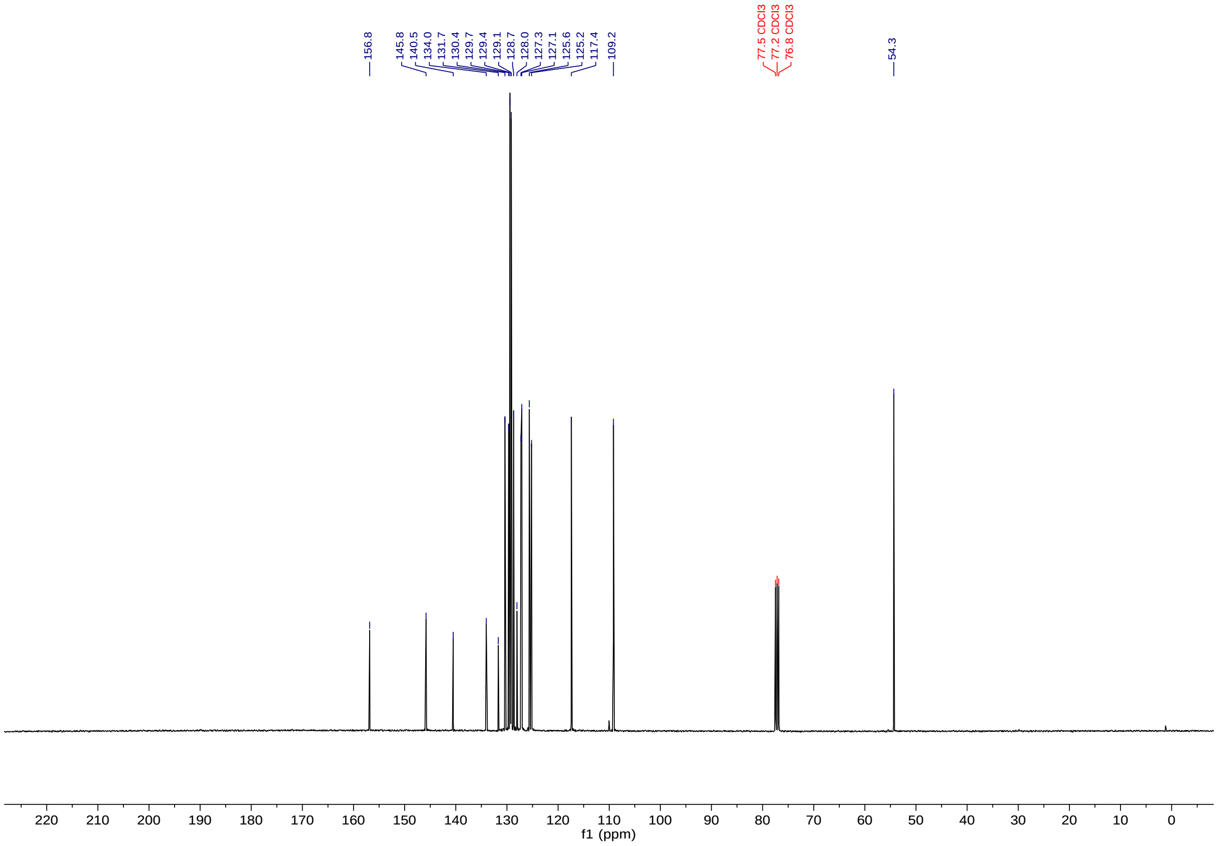

1H NMR: (500 MHz, $CDCl_3$) **15c**

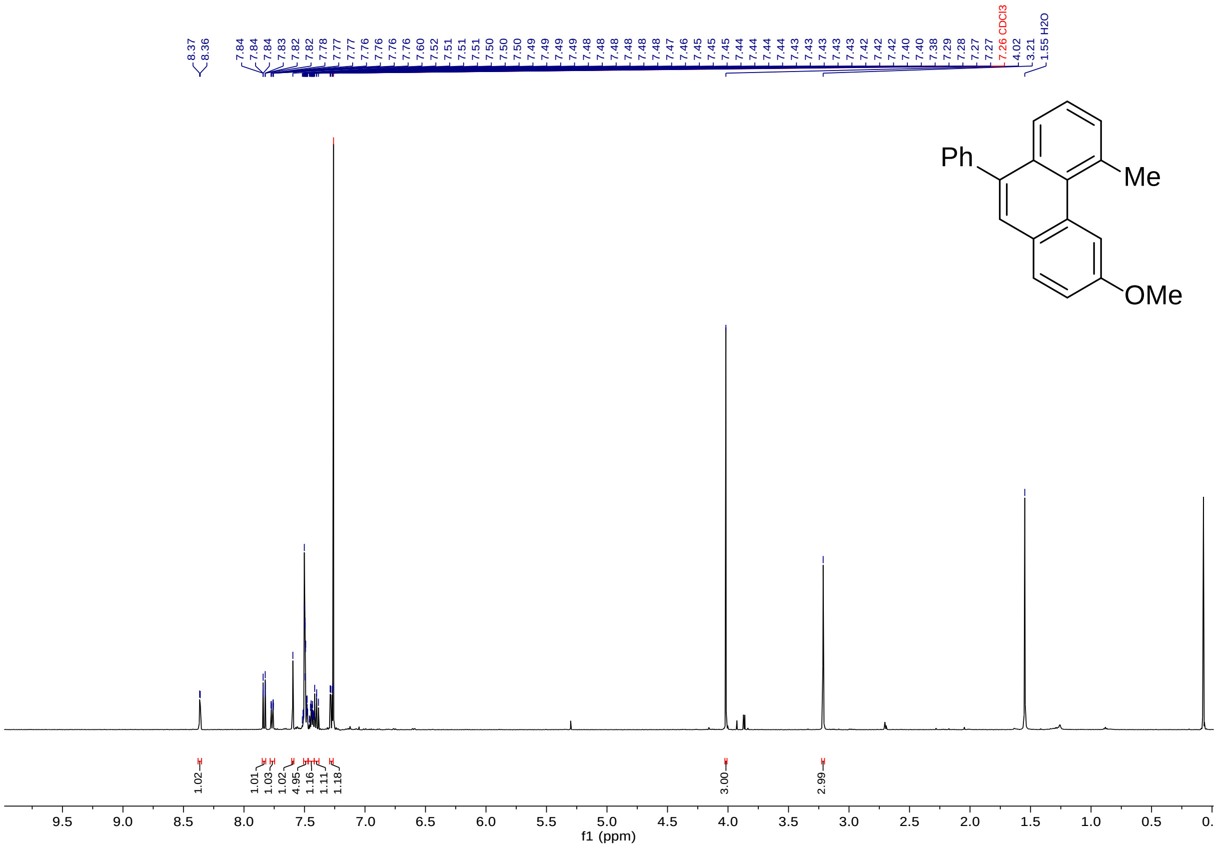

13C{H} NMR: (101 MHz, $CDCl_3$) **15c**

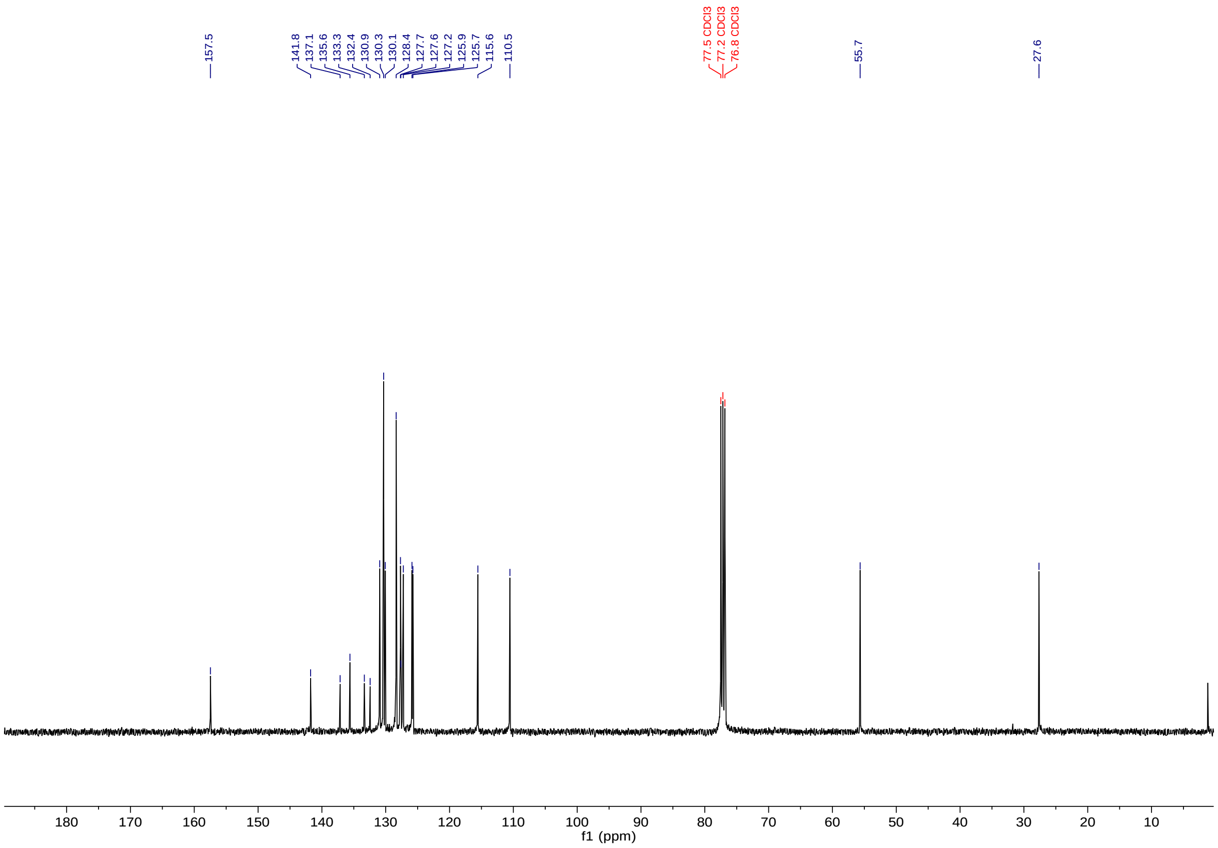

1H NMR: (400 MHz, $CDCl_3$) **16a**

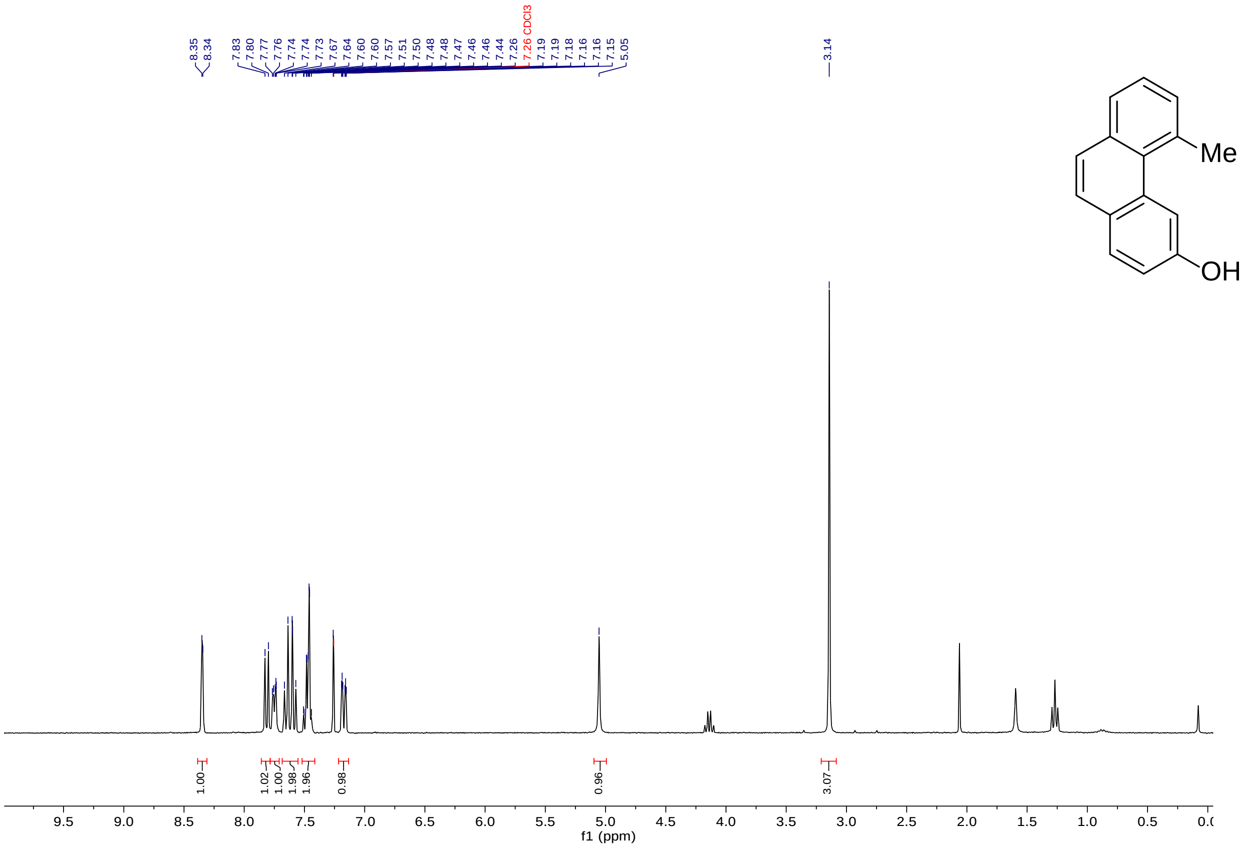

13C{H} NMR: (101 MHz, $CDCl_3$) **16b**

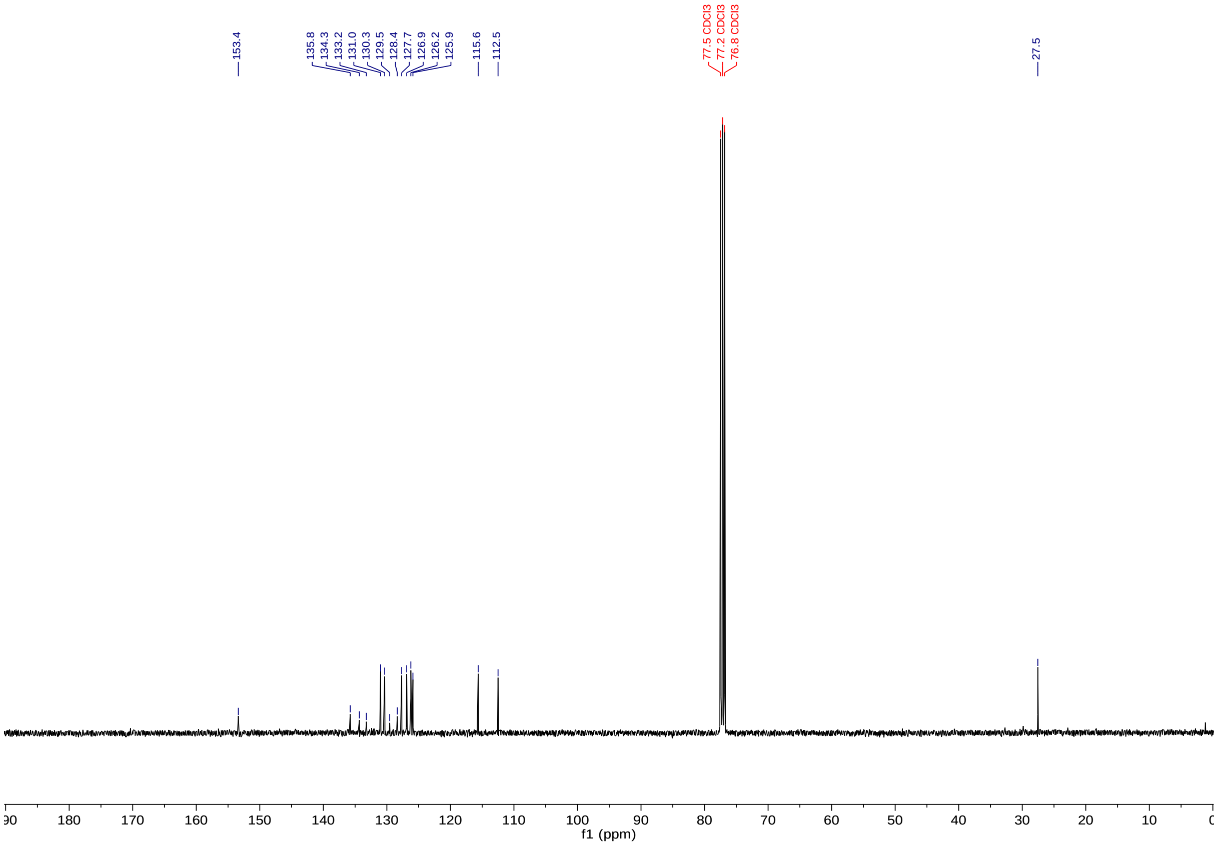

1H NMR: (400 MHz, $CDCl_3$) **16b**

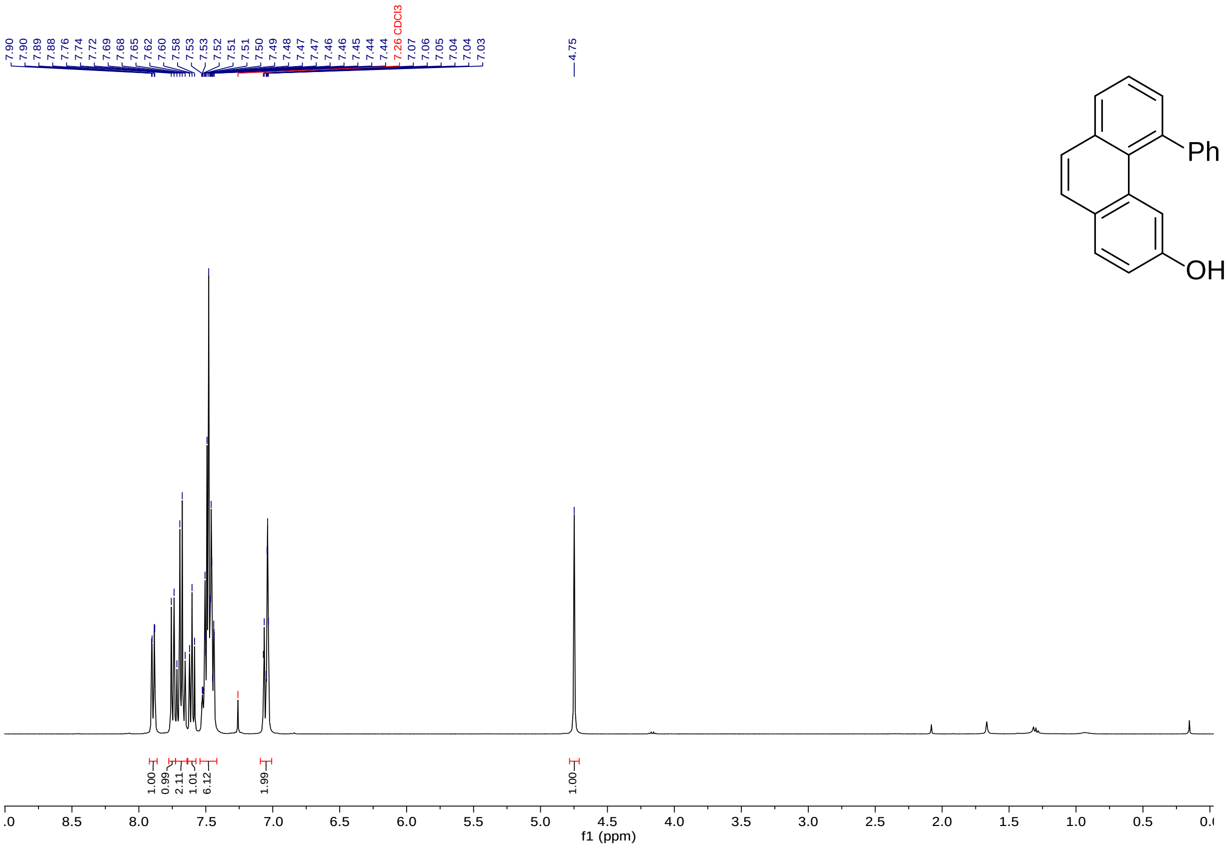

13C{H} NMR: (101 MHz, $CDCl_3$) **16b**

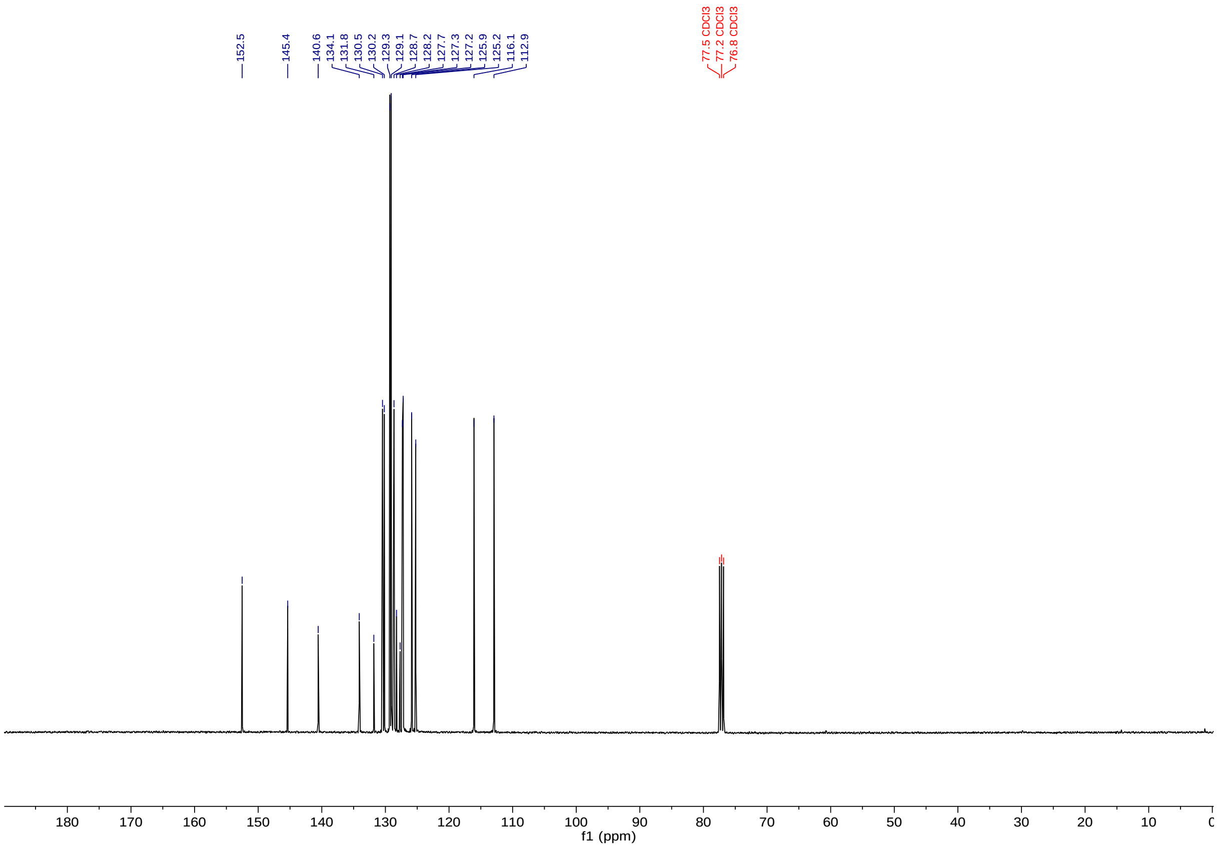

1H NMR: (500 MHz, $CDCl_3$) **16c**

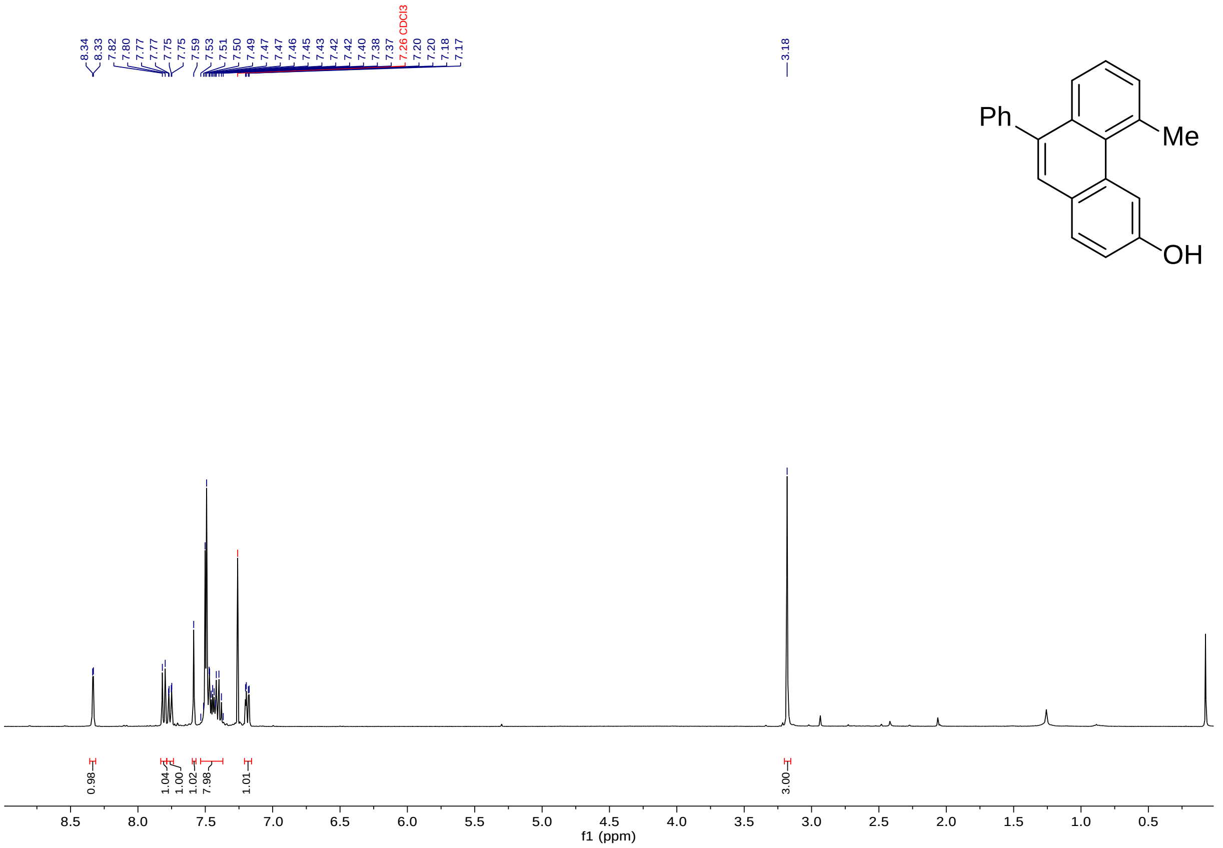

13C{H} NMR: (101 MHz, $CDCl_3$) **16c**

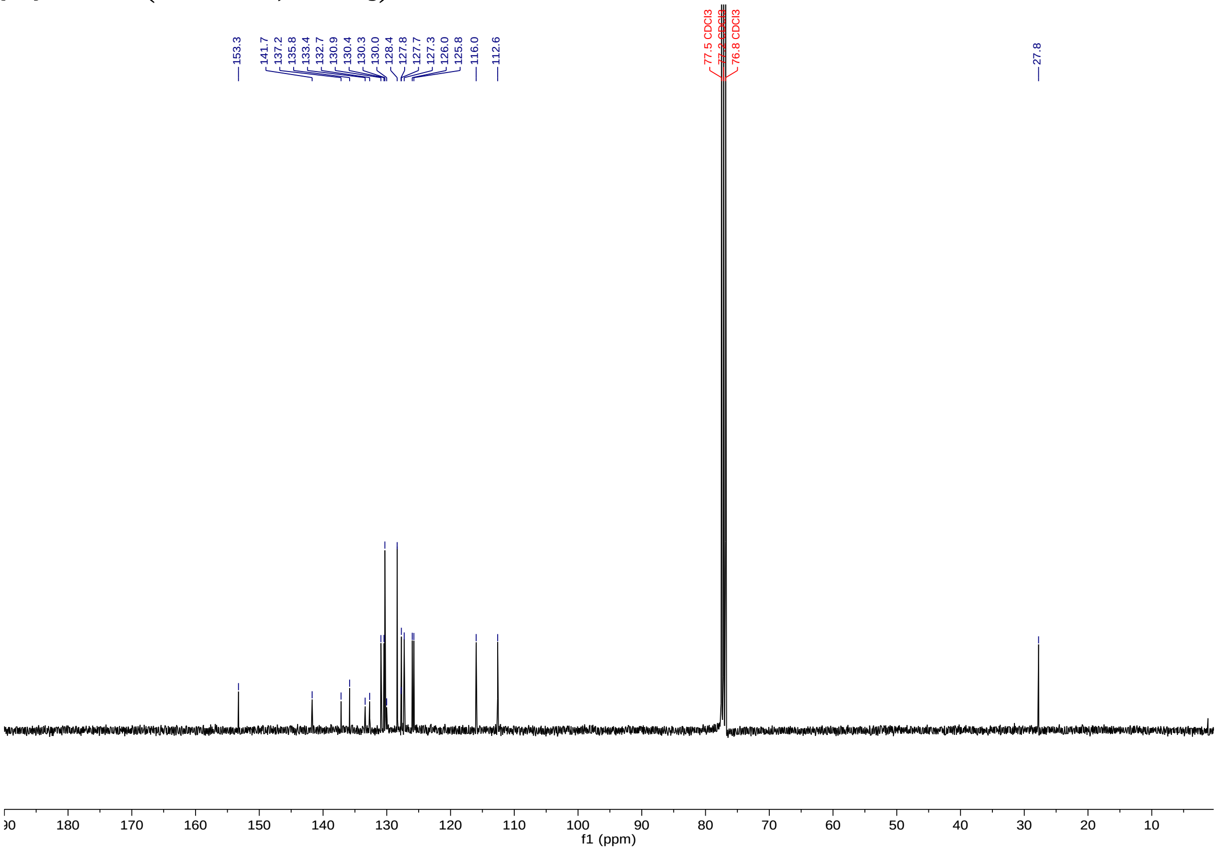

1H NMR: (500 MHz, $CDCl_3$) **16d**

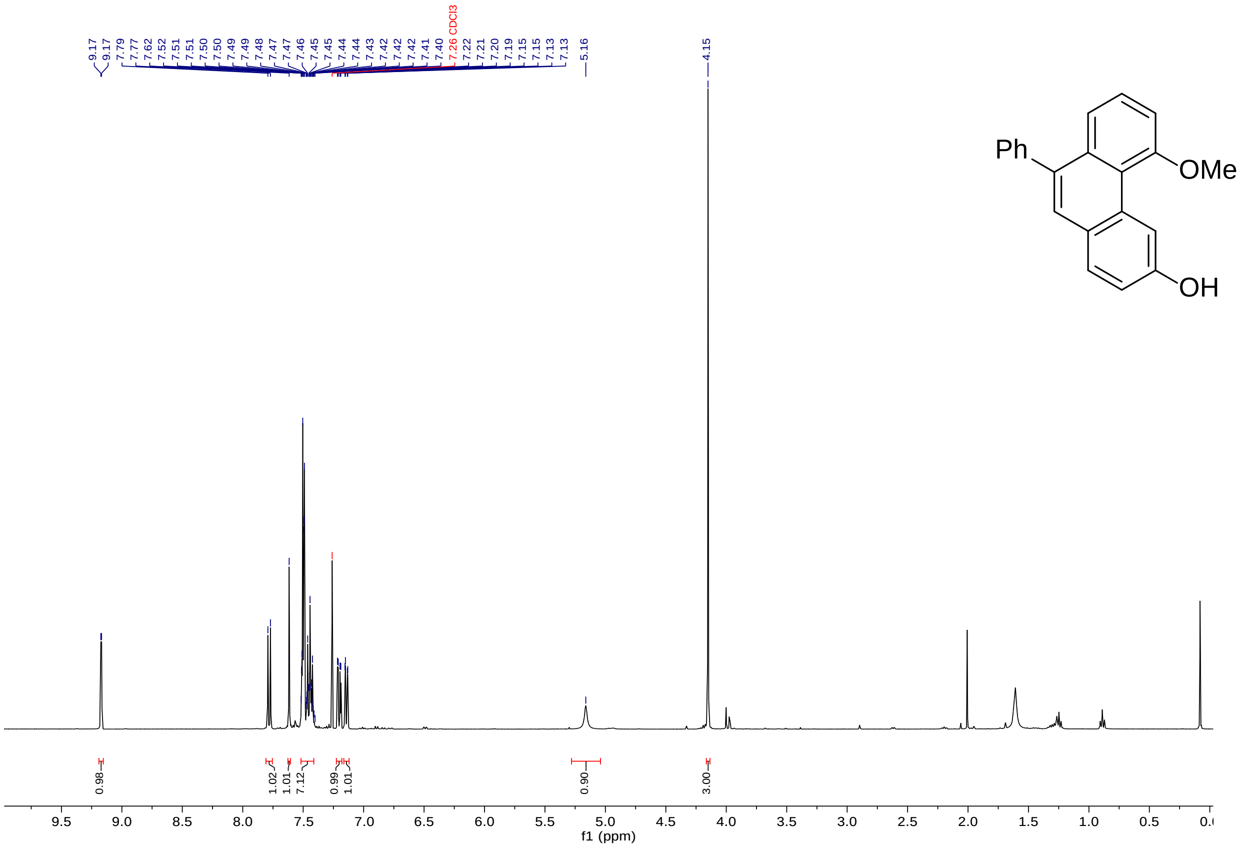

13C{H} NMR: (101 MHz, $CDCl_3$) **16d**

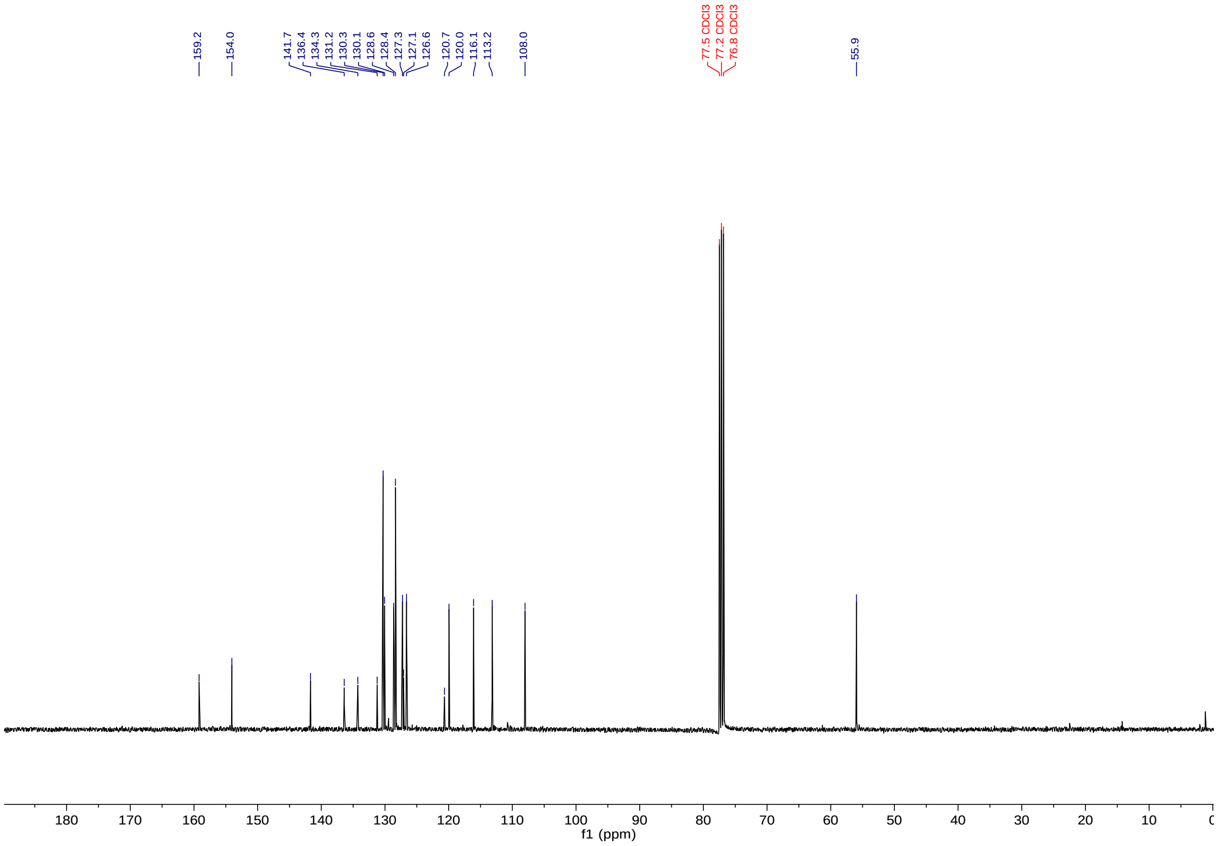

1H NMR: (400 MHz, $CDCl_3$) **17a**

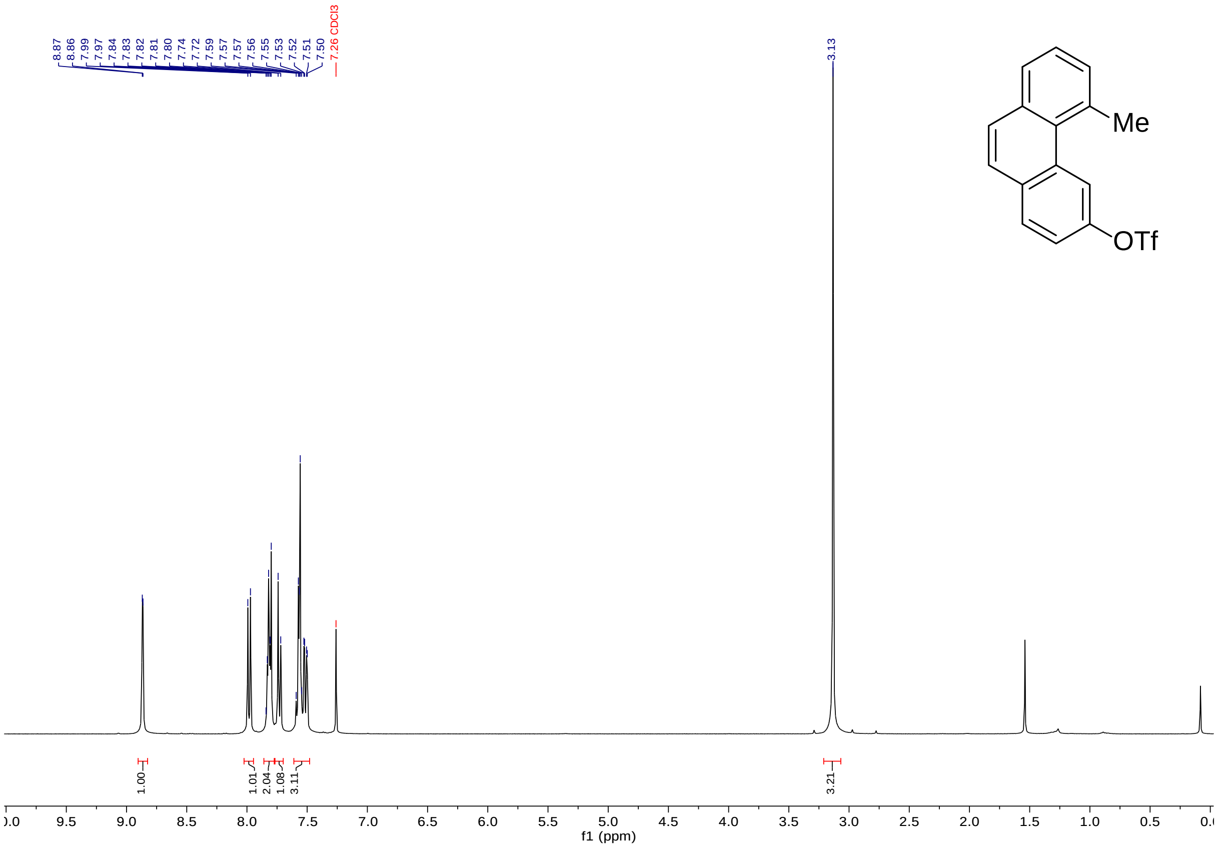

13C{H} NMR: (101 MHz, $CDCl_3$) **17a**

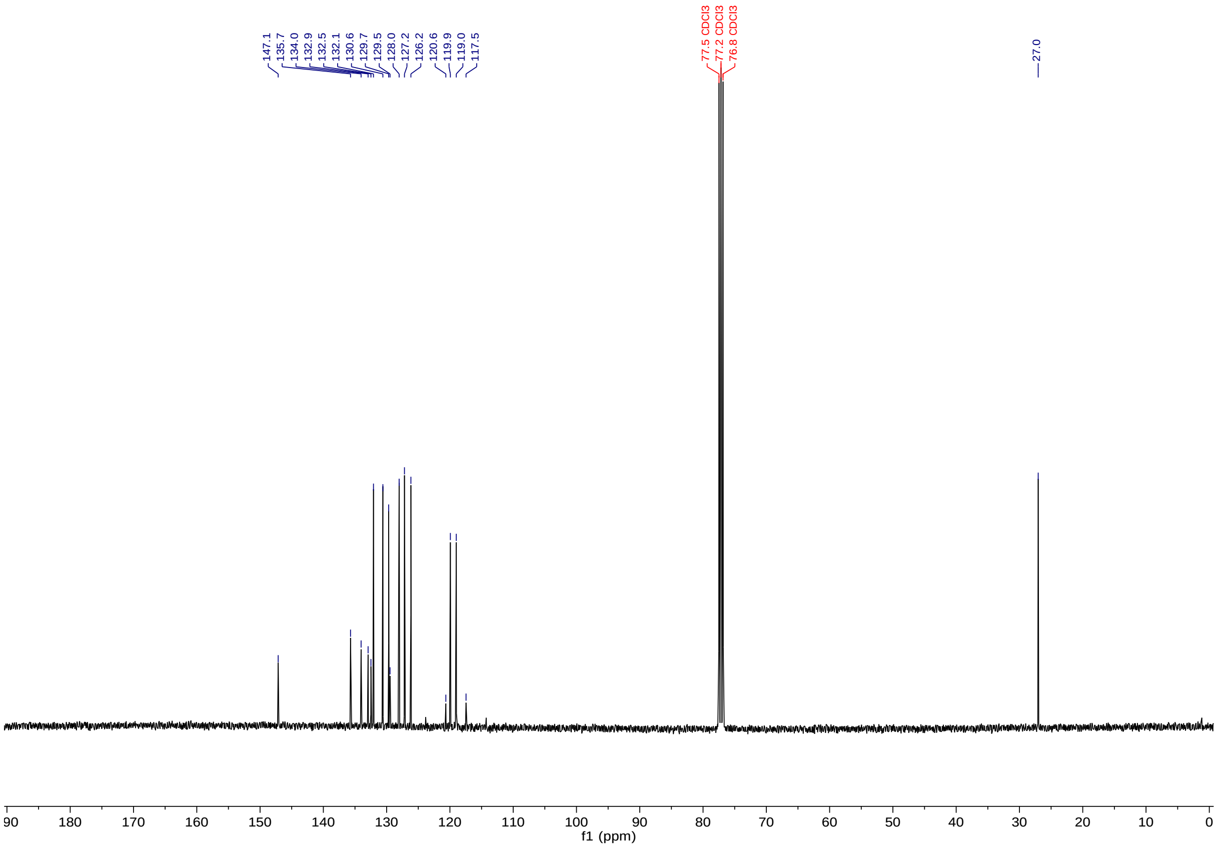

19F{H} NMR: (377 MHz, $CDCl_3$) **17a**

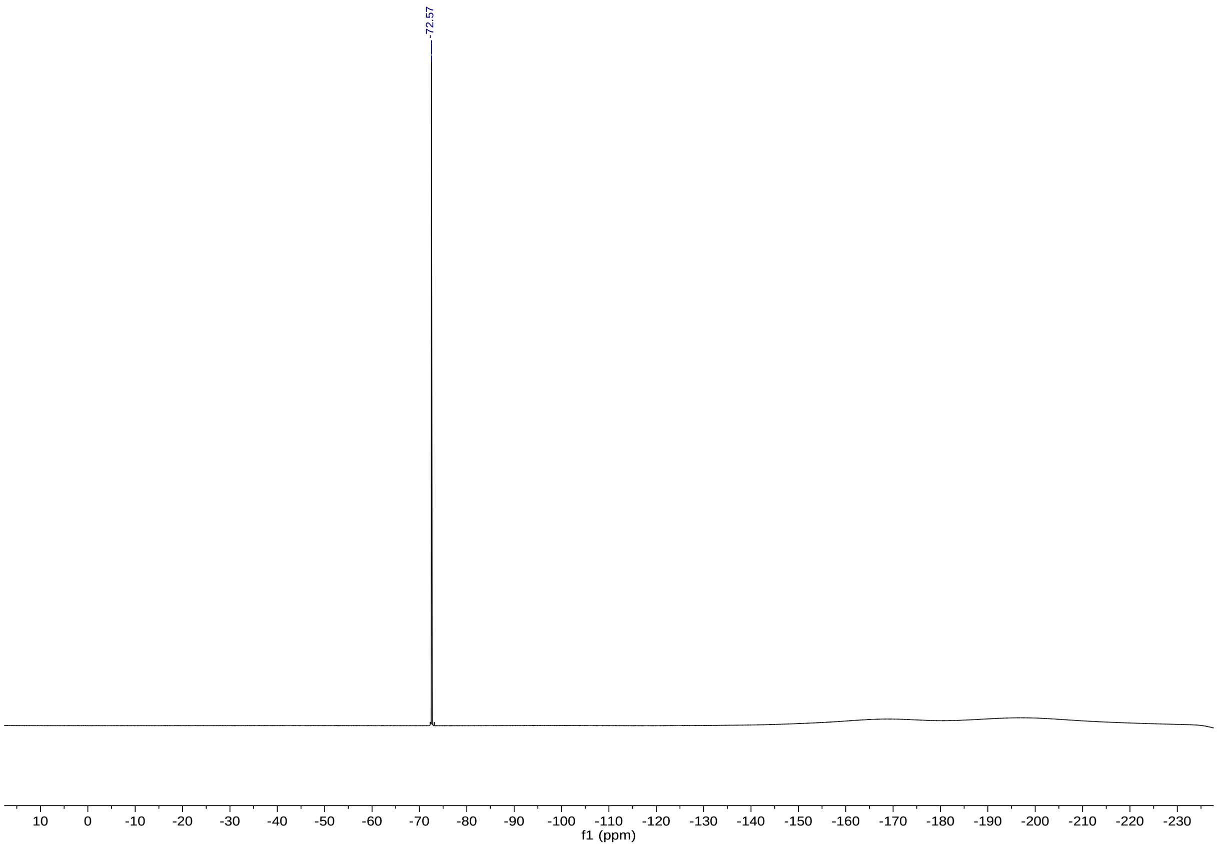

1H NMR: (400 MHz, $CDCl_3$) **17b**

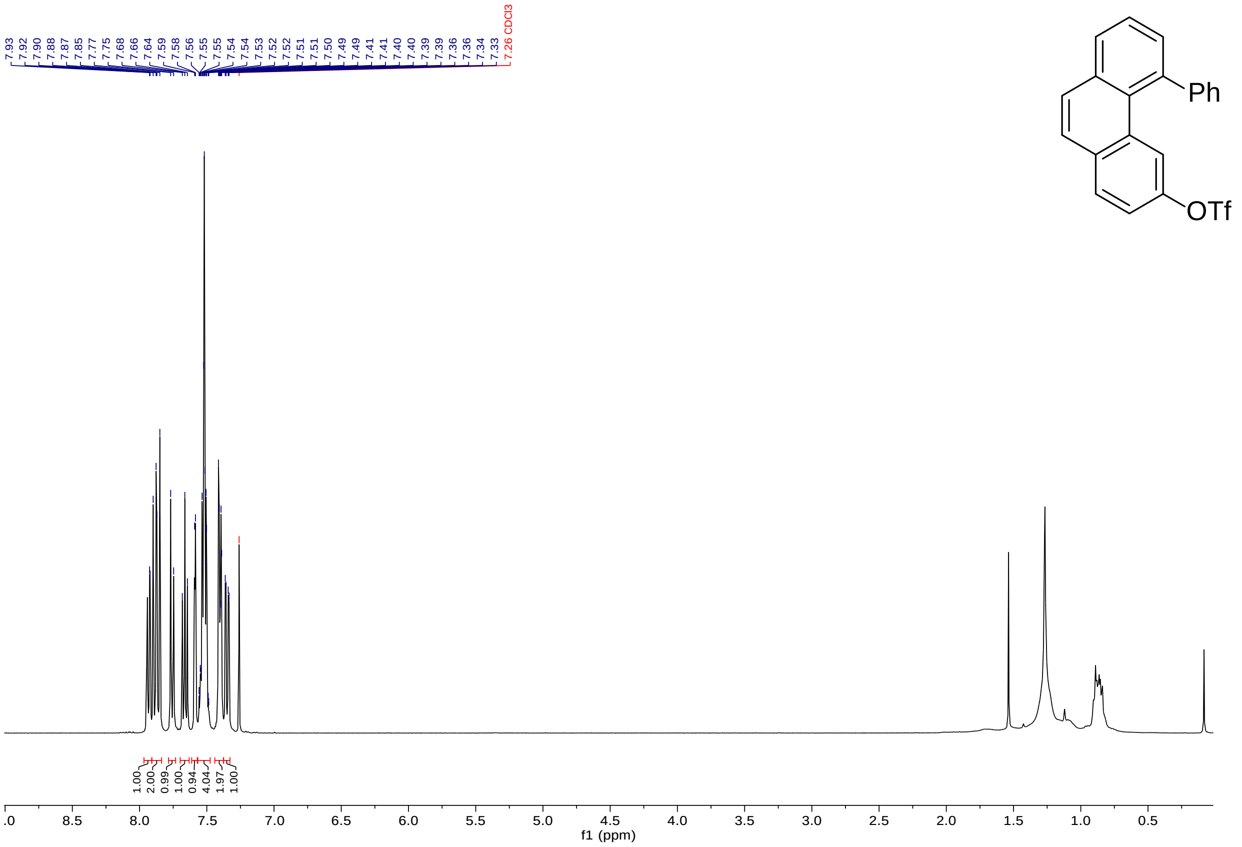

13C{H} NMR: (101 MHz, $CDCl_3$) **17b**

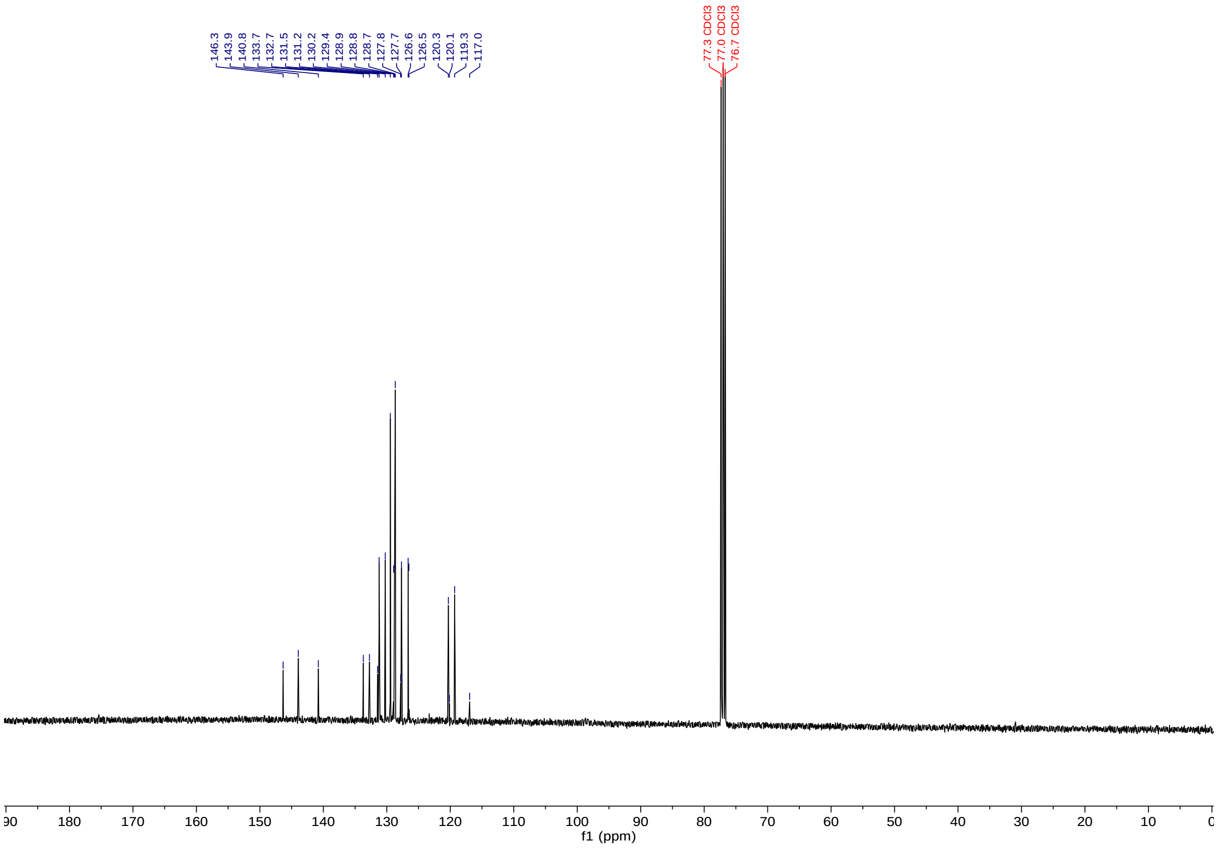

19F{H} NMR: (377 MHz, $CDCl_3$) **17b**

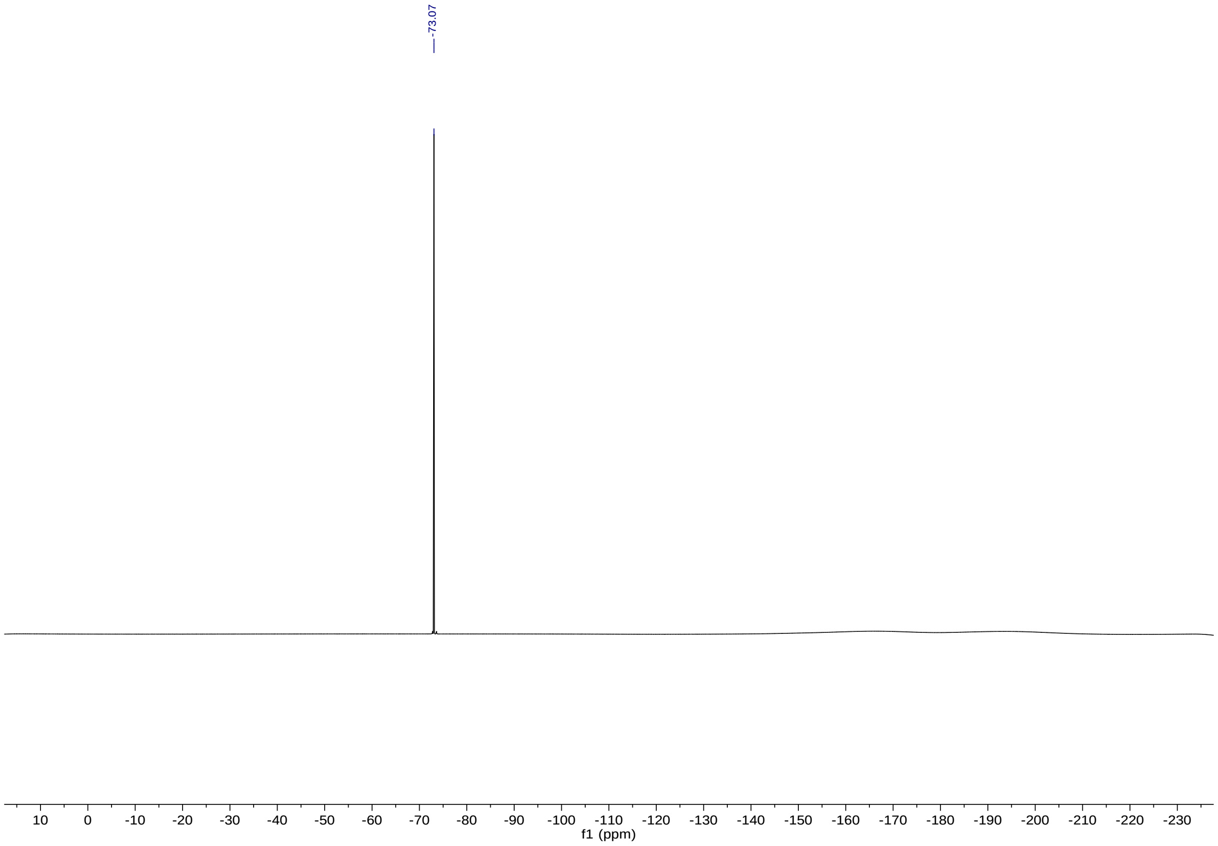

1H NMR: (400 MHz, $CDCl_3$) **17c**

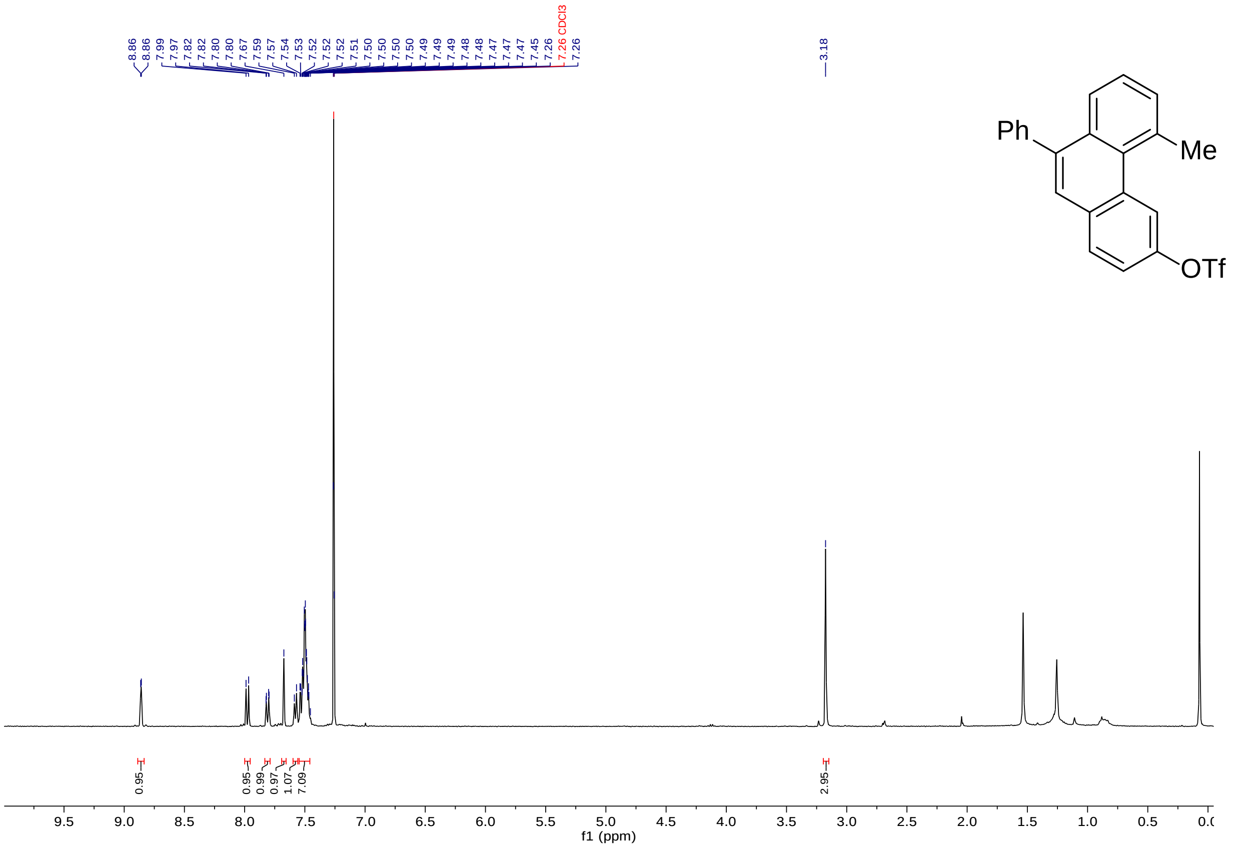

13C{H} NMR: (101 MHz, $CDCl_3$) **17c**

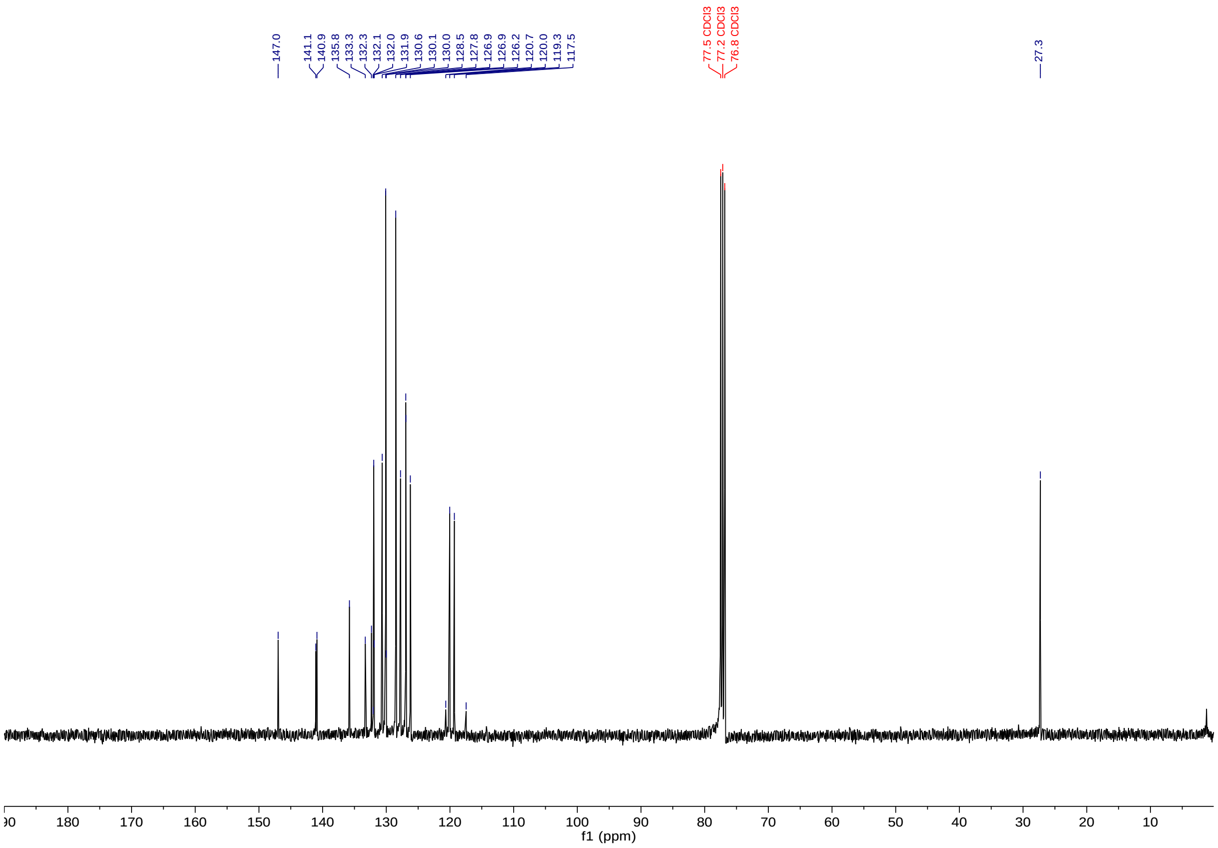

19F{H} NMR: (377 MHz, $CDCl_3$) **17c**

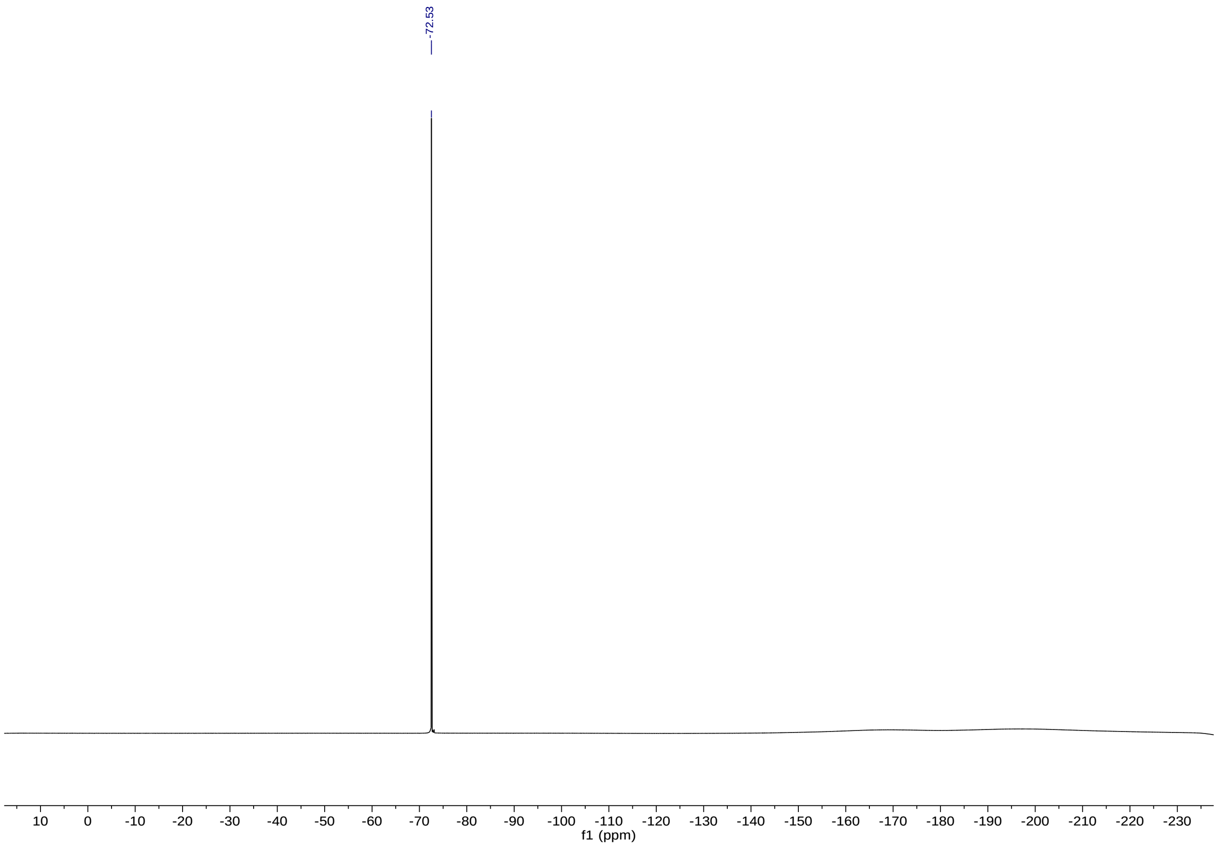

1H NMR: (300 MHz, $CDCl_3$) **17d**

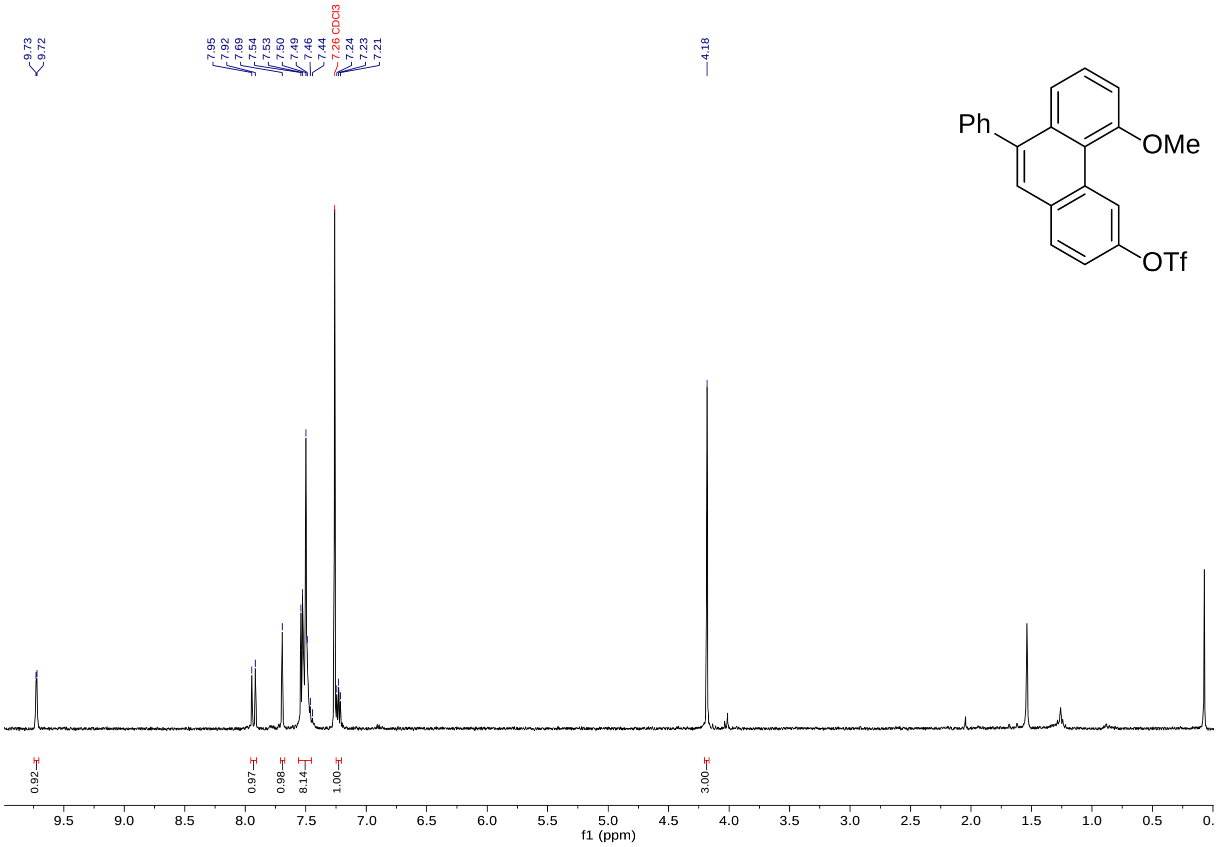

13C{H} NMR: (101 MHz, $CDCl_3$) **17d**

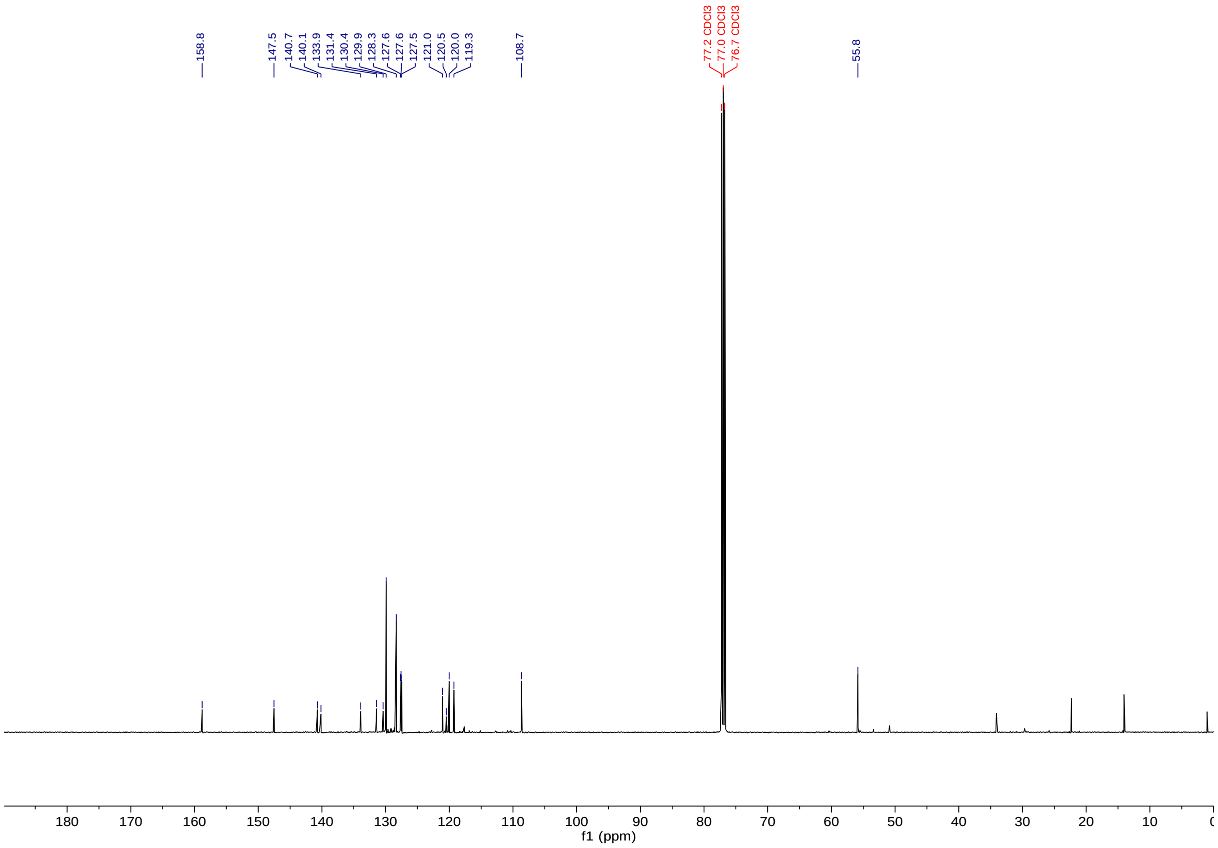

19F{H} NMR: (377 MHz, $CDCl_3$) **17d**

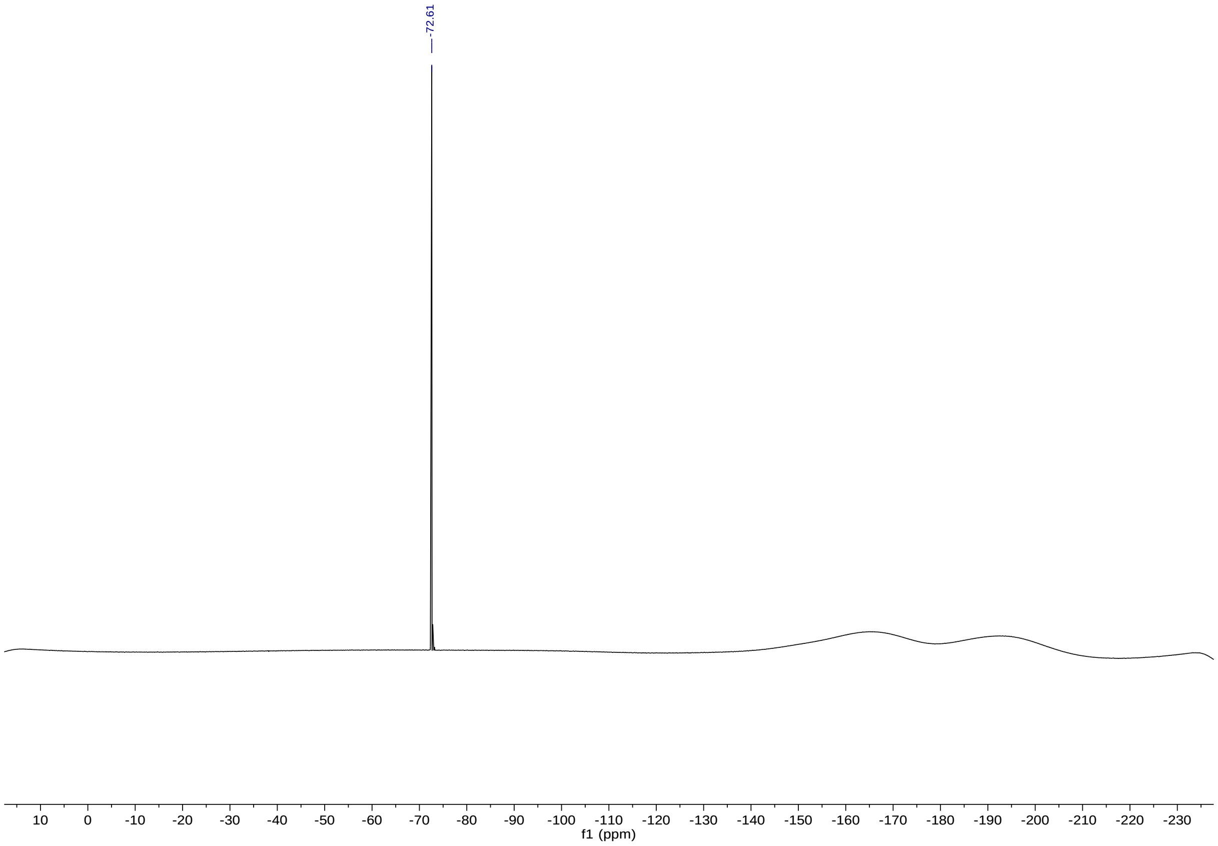

1H NMR: (400 MHz, $CDCl_3$) **18a**

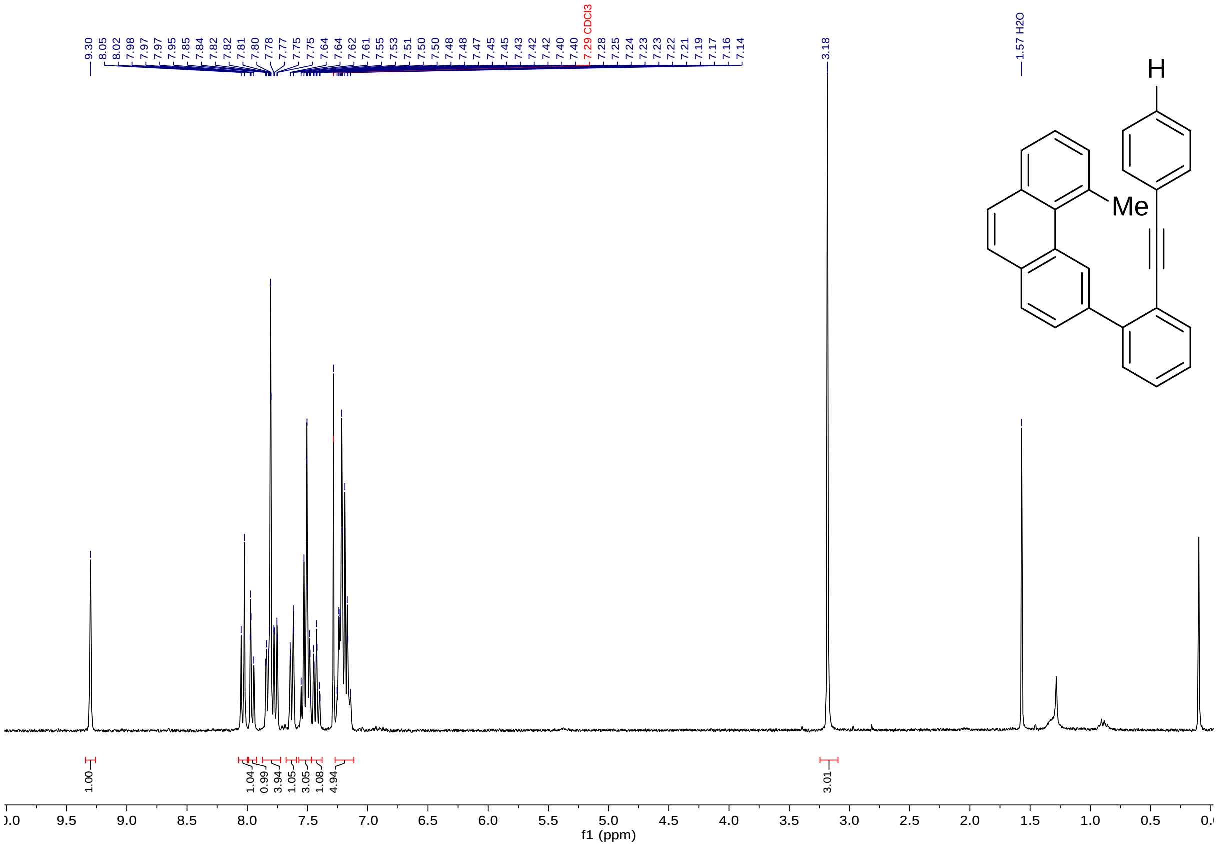

13C{H} NMR: (101 MHz, $CDCl_3$) **18a**

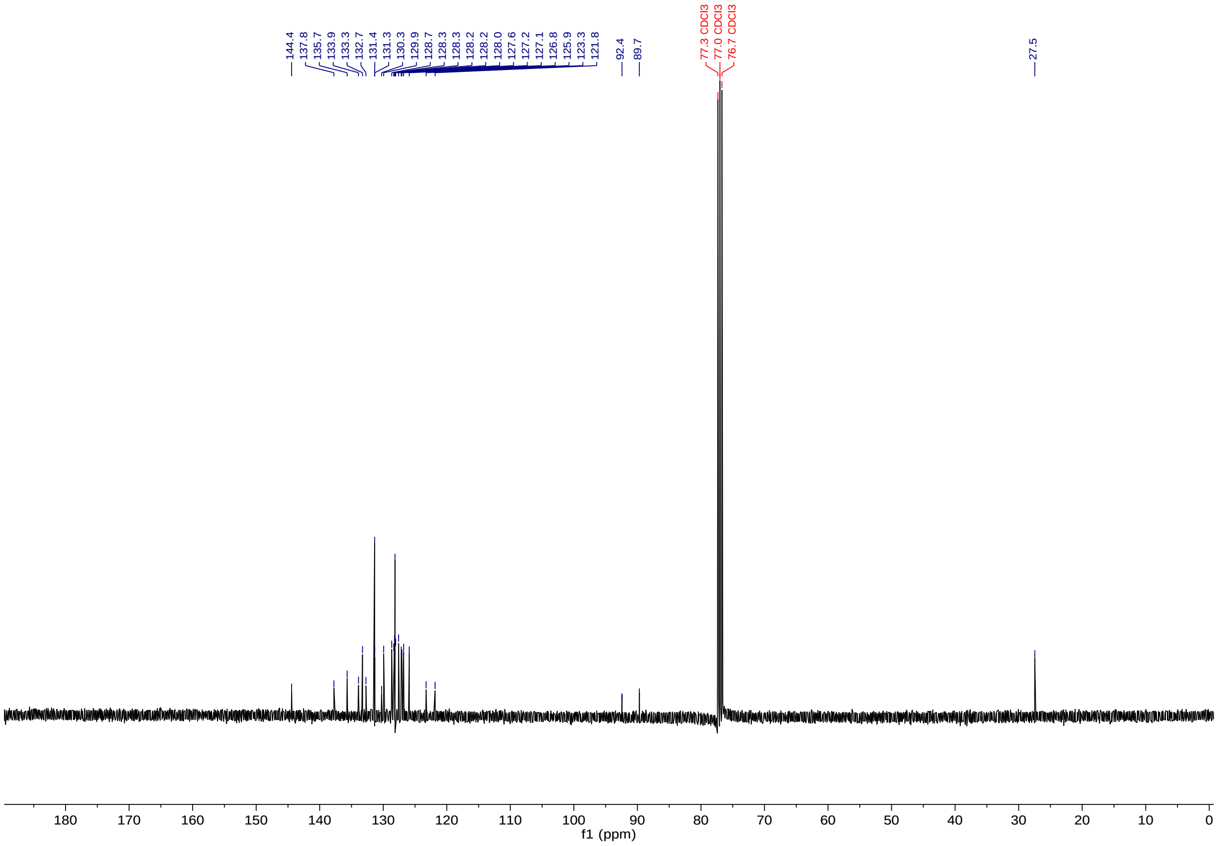

1H NMR: (400 MHz, $CDCl_3$) **18b**

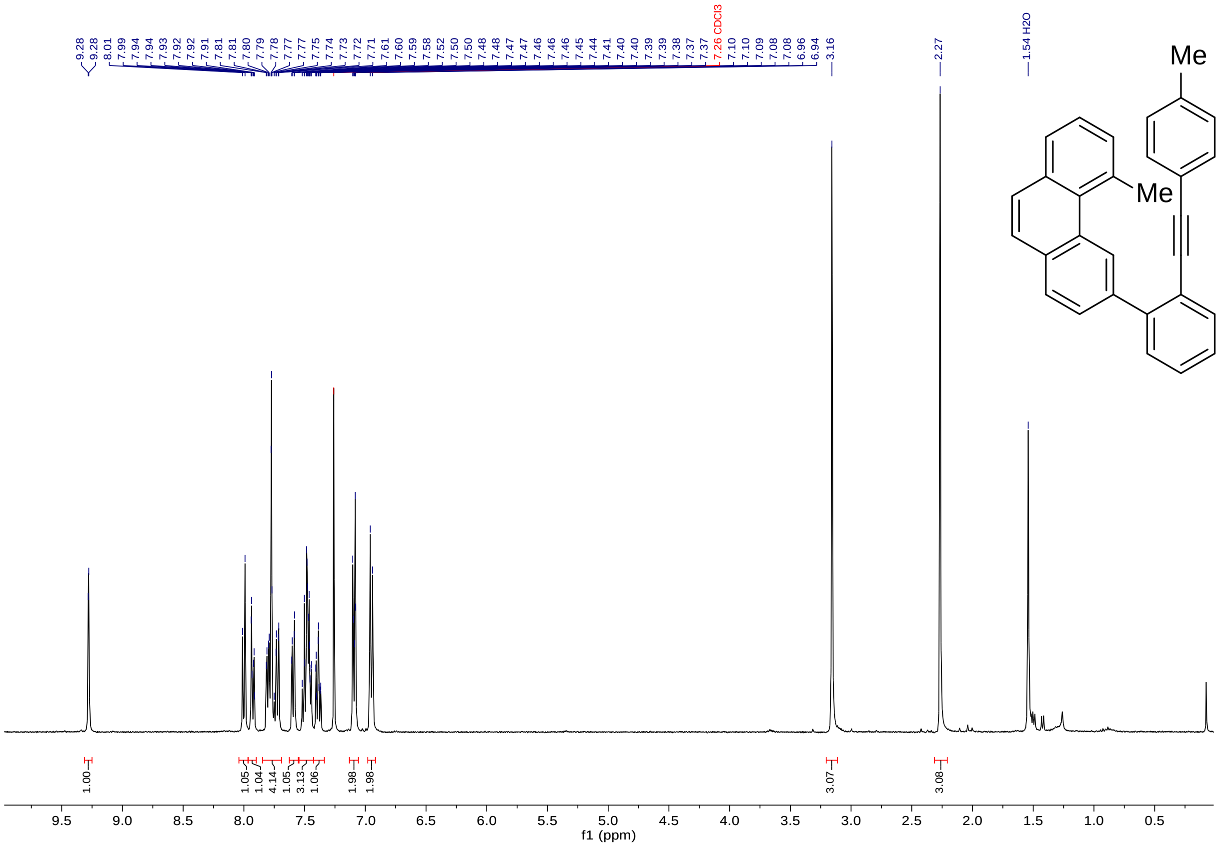

13C{H} NMR: (101 MHz, $CDCl_3$) **18c**

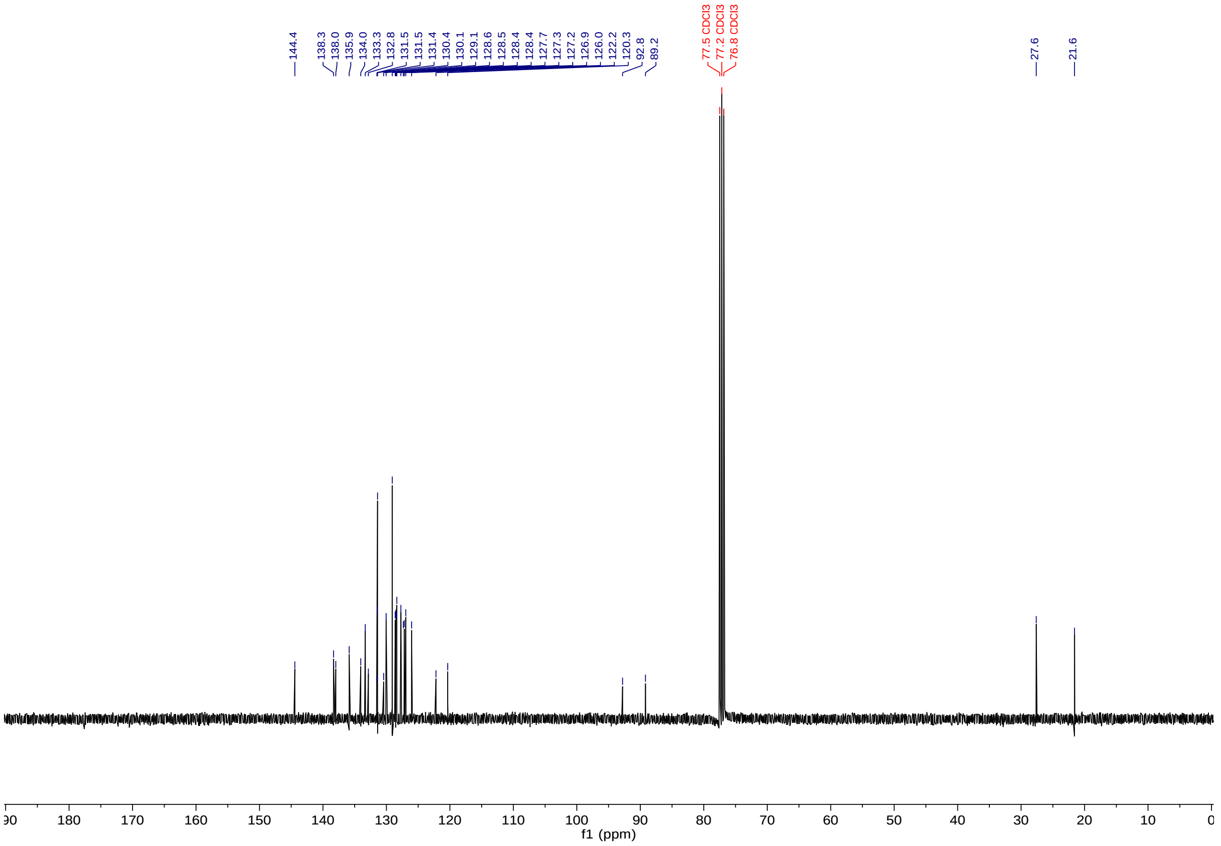

1H NMR: (400 MHz, $CDCl_3$) **18c**

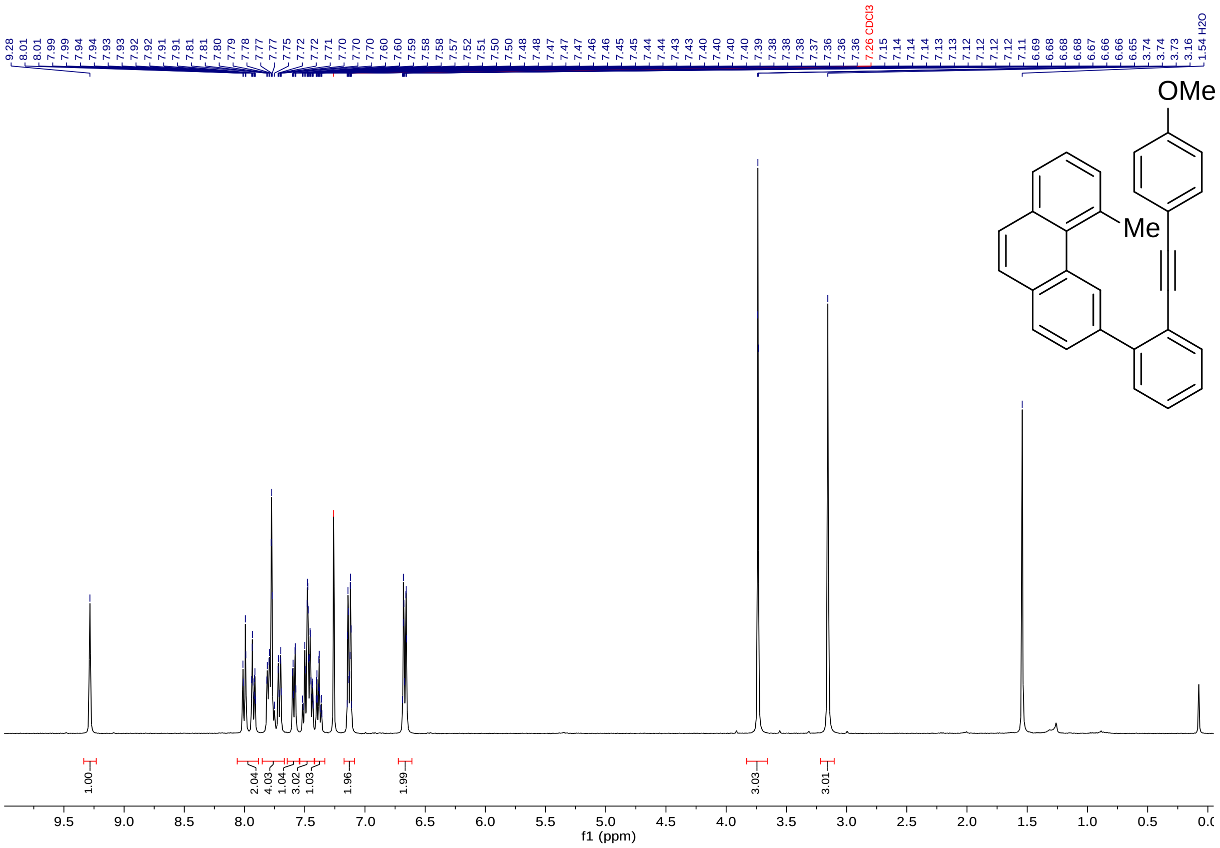

13C{H} NMR: (101 MHz, $CDCl_3$) **18c**

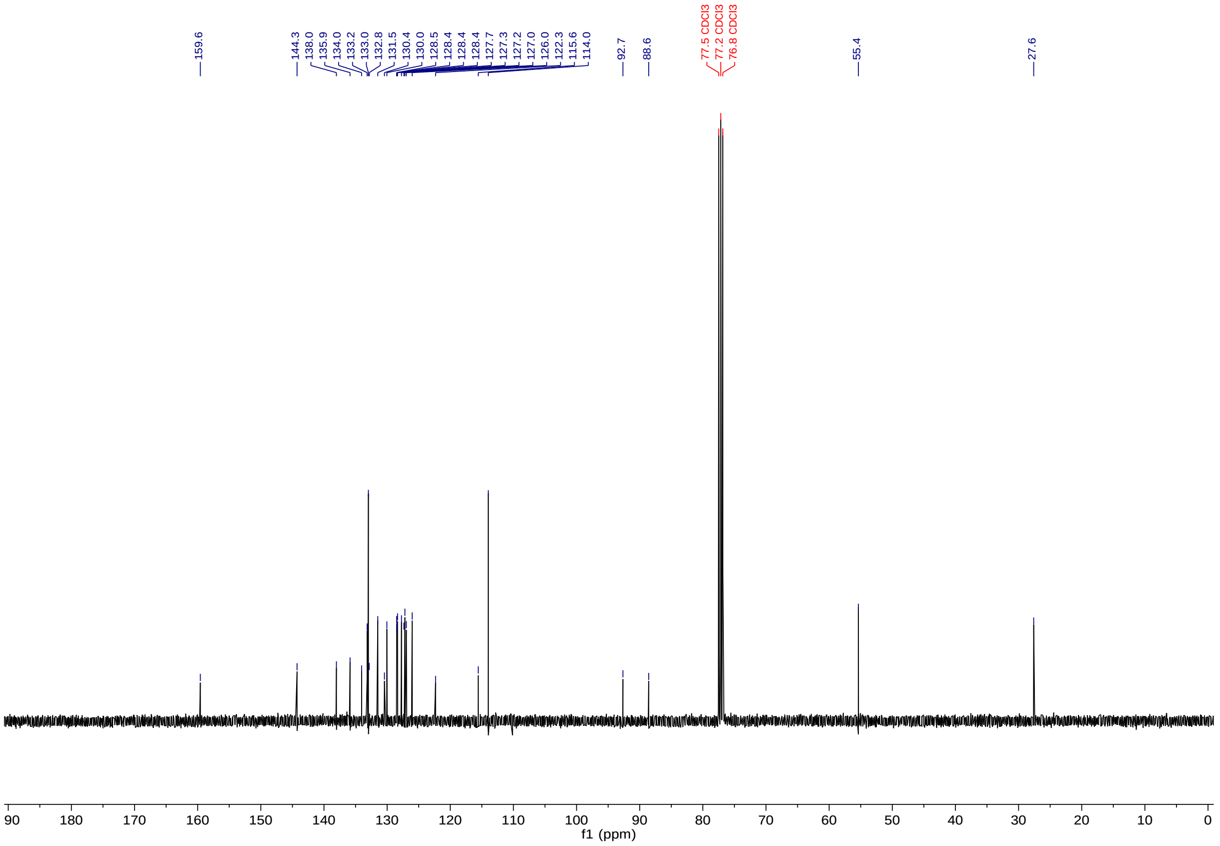

1H NMR: (400 MHz, $CDCl_3$) **18d**

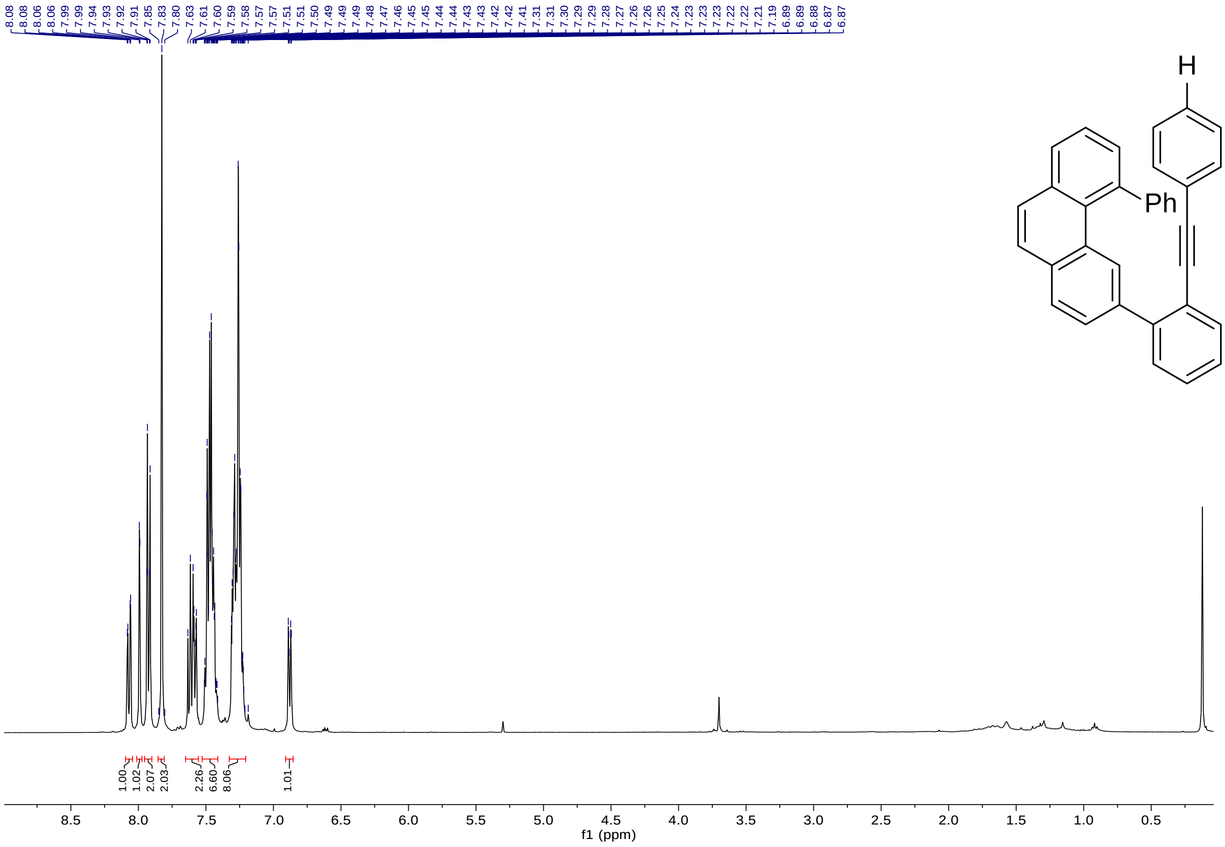

13C{H} NMR: (101 MHz, $CDCl_3$) **18d**

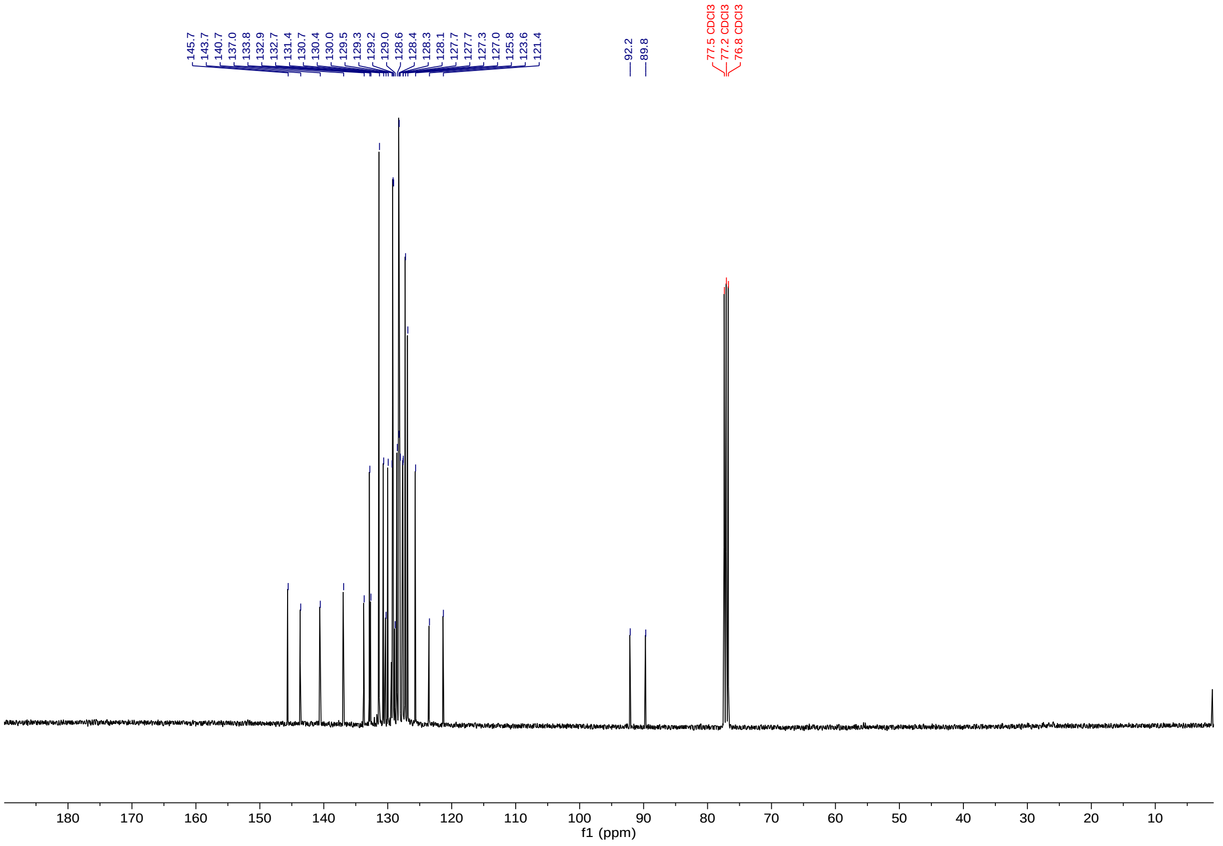

1H NMR: (400 MHz, $CDCl_3$) **18e**

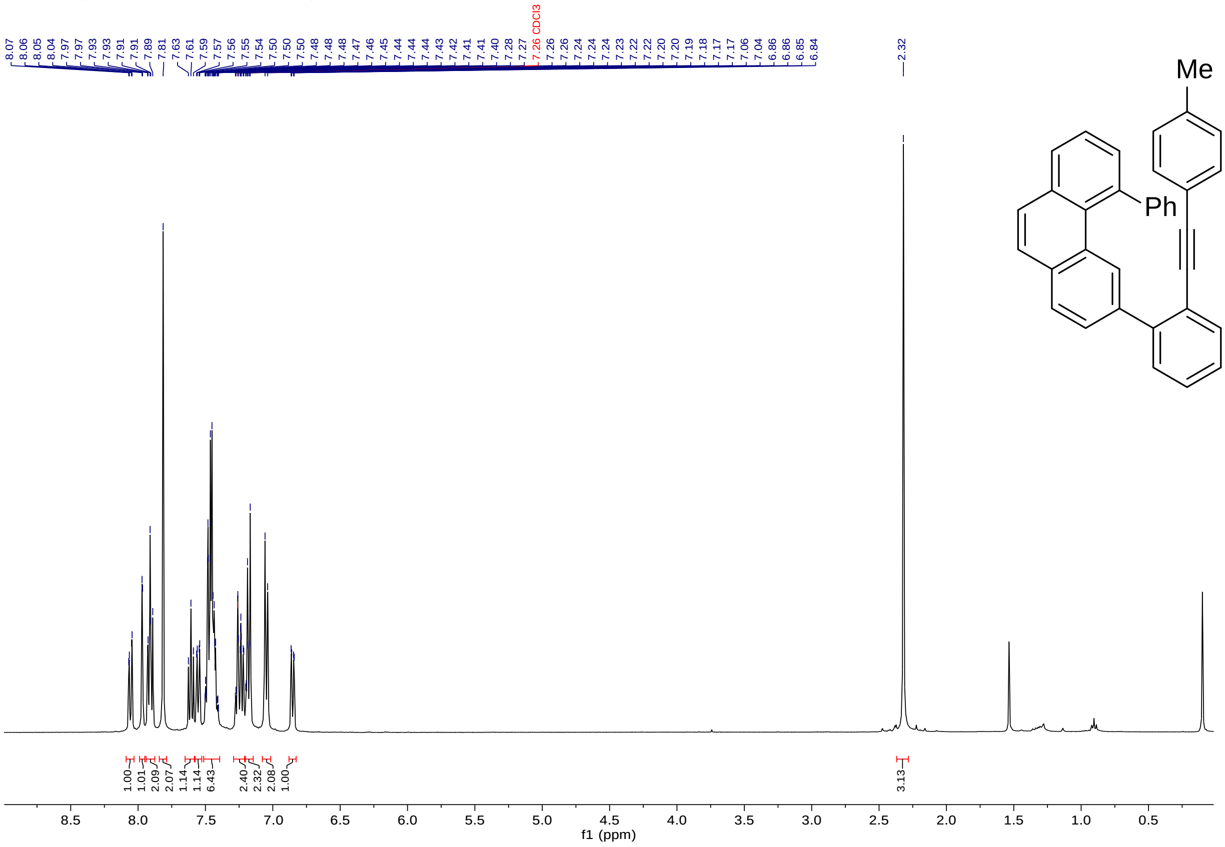

13C{H} NMR: (101 MHz, $CDCl_3$) **18e**

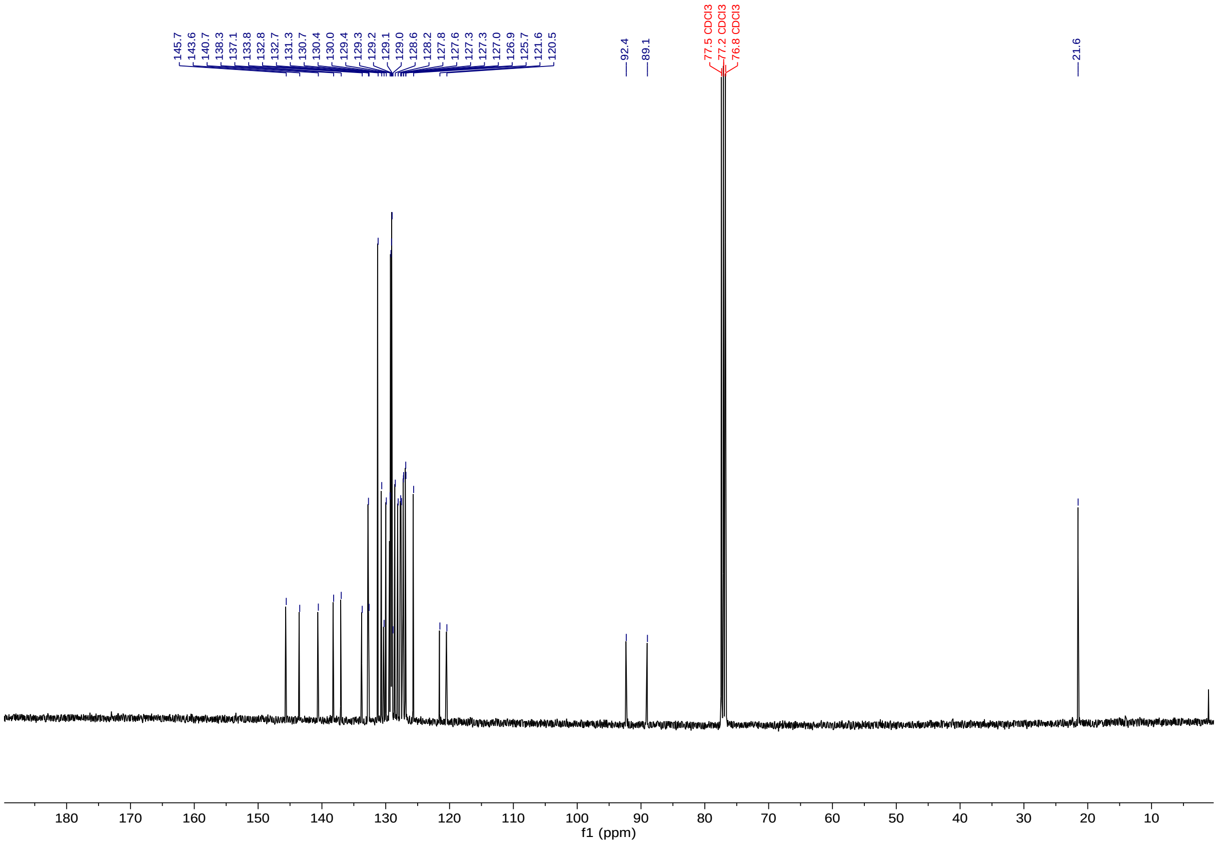

1H NMR: (400 MHz, $CDCl_3$) **18f**

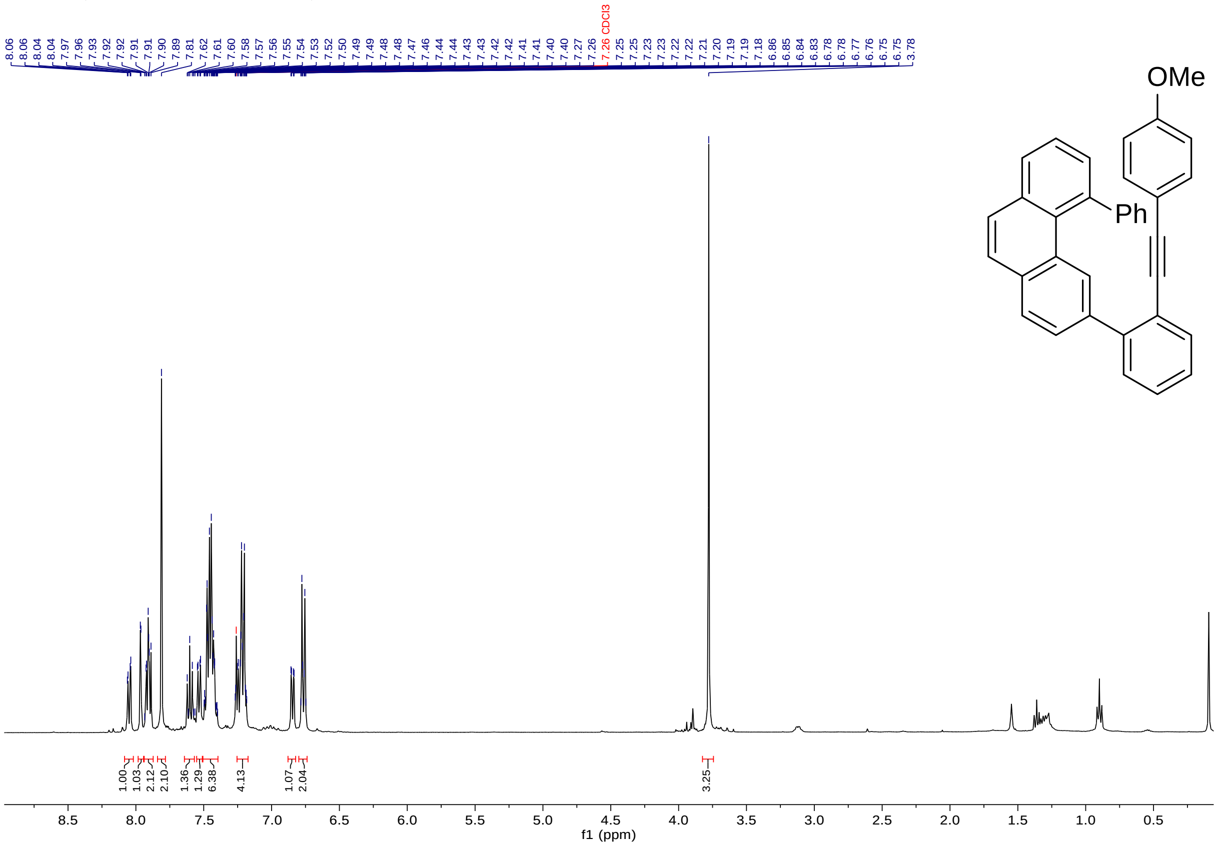

13C{H} NMR: (101 MHz, $CDCl_3$) **18f**

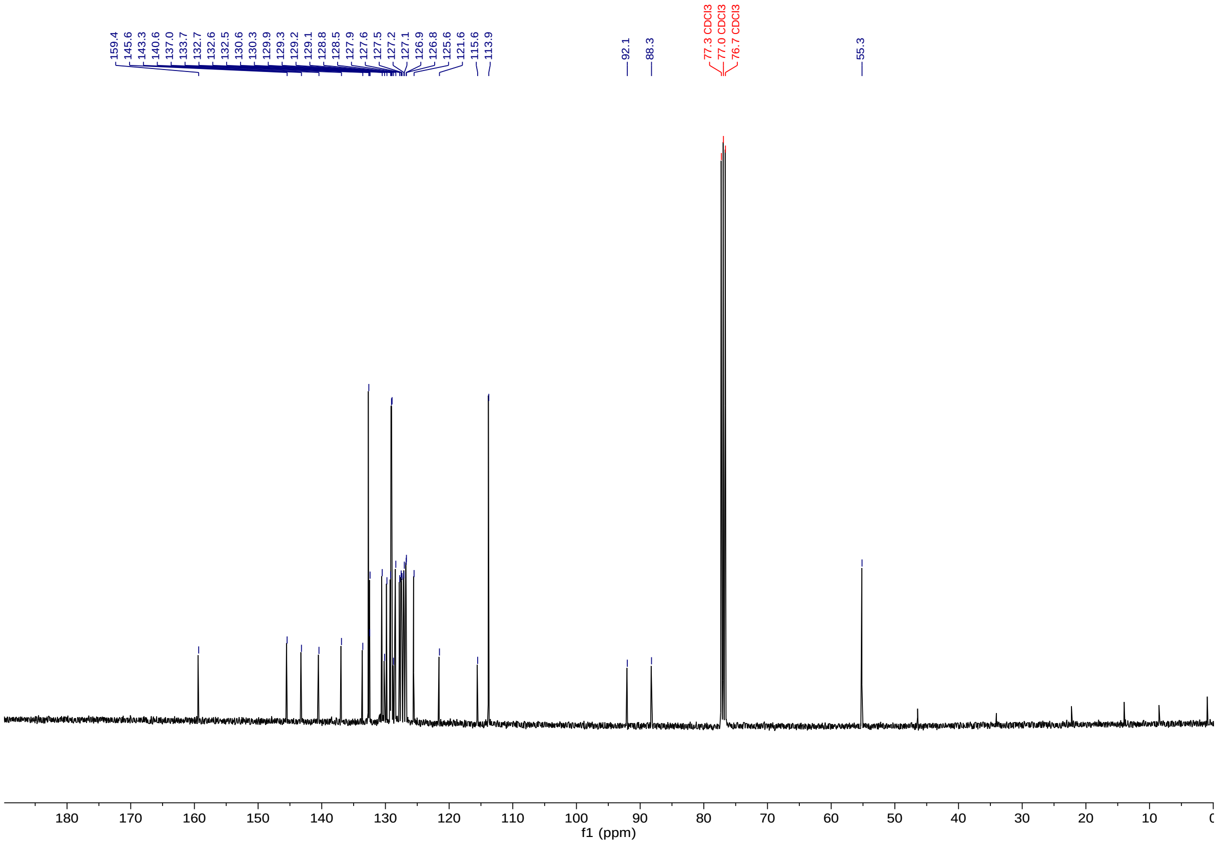

1H NMR: (500 MHz, $CDCl_3$) **18g**

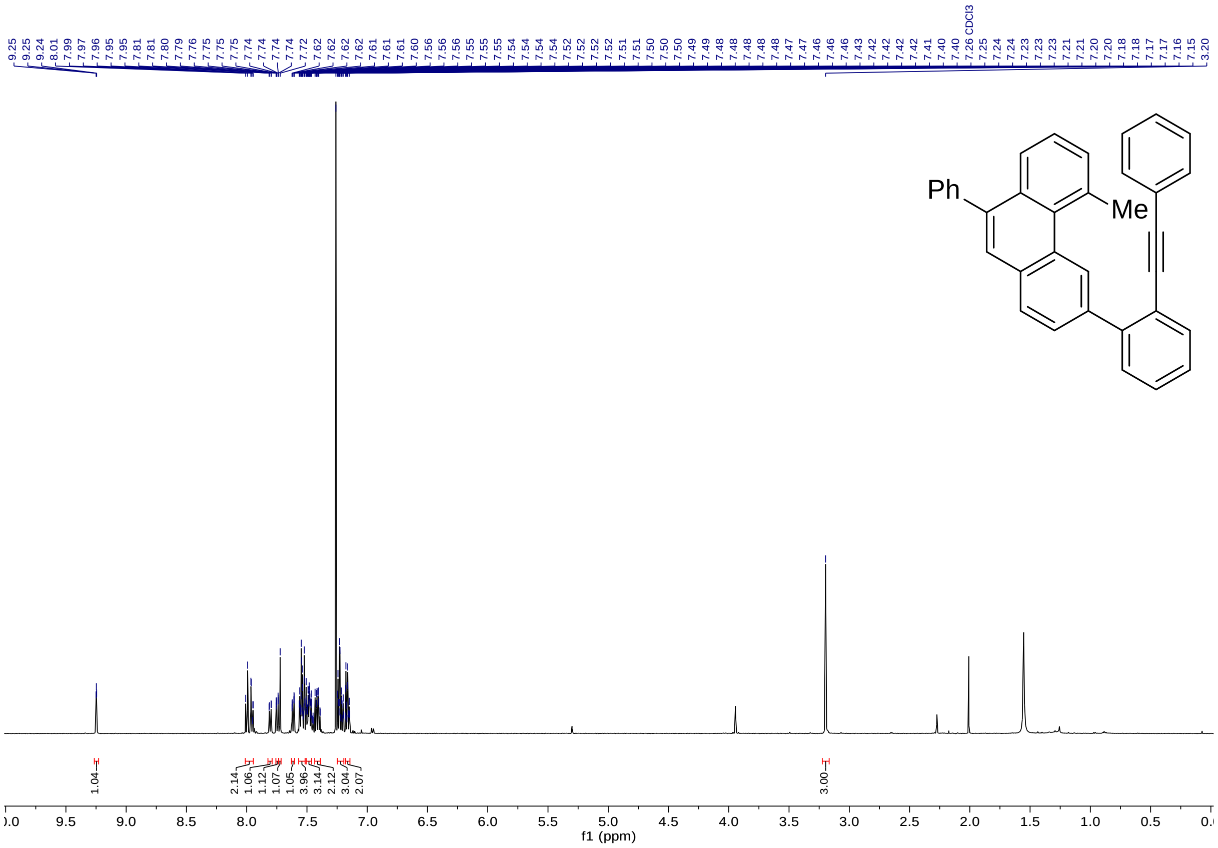

13C{H} NMR: (101 MHz, $CDCl_3$) **18g**

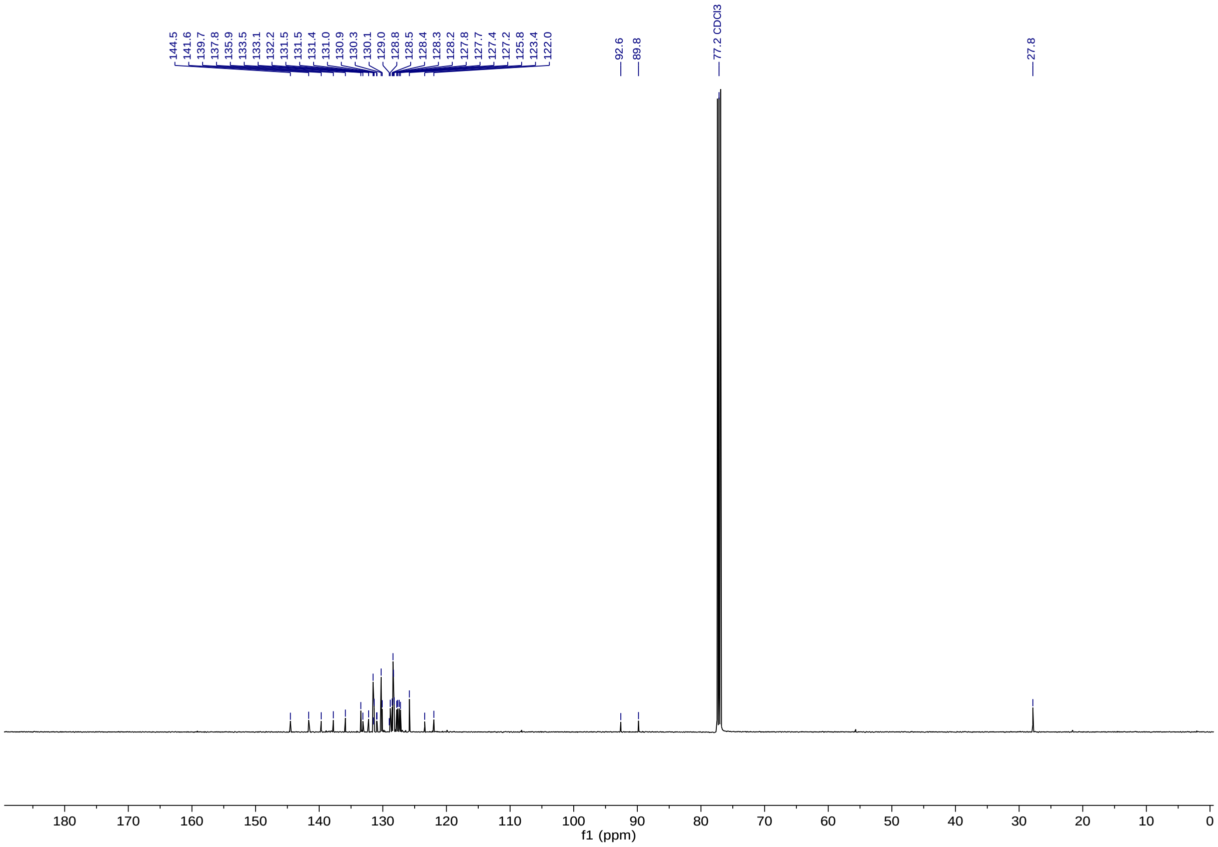

1H NMR: (400 MHz, $CDCl_3$) **18h**

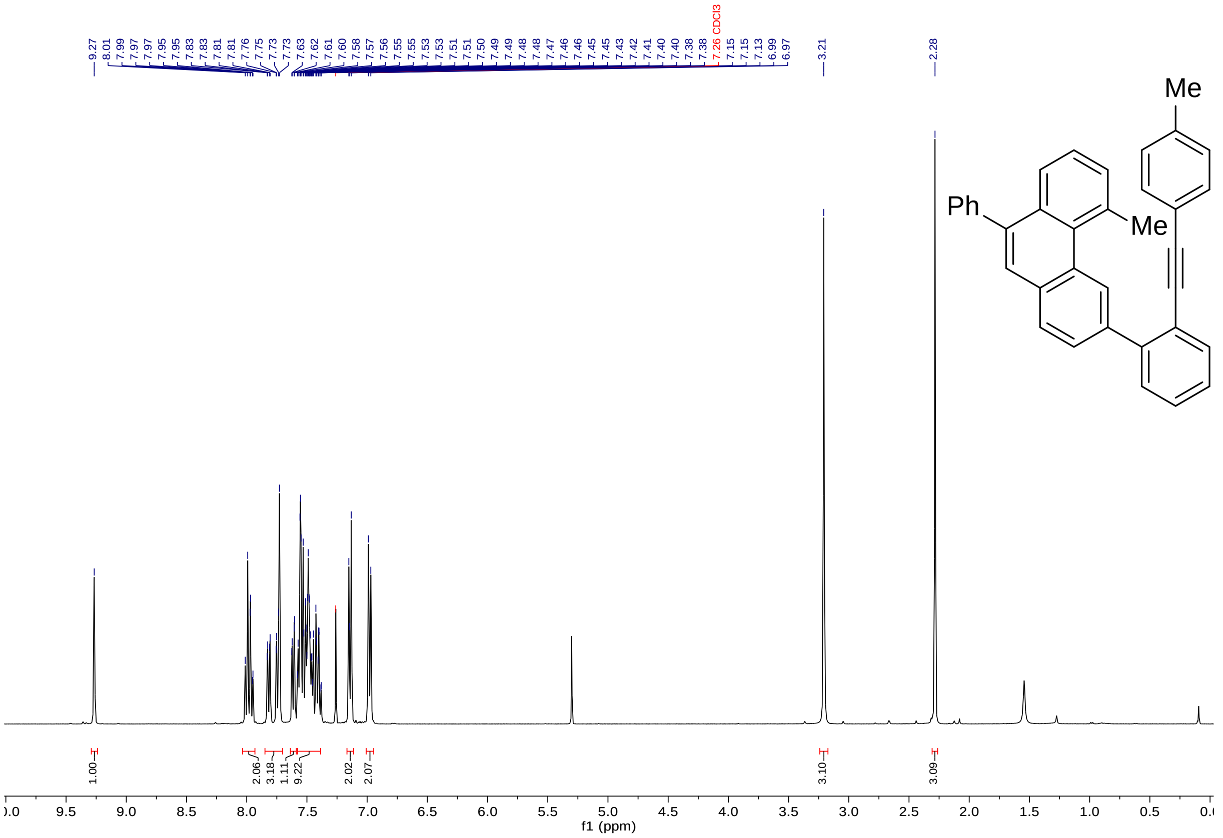

13C{H} NMR: (101 MHz, $CDCl_3$) **18h**

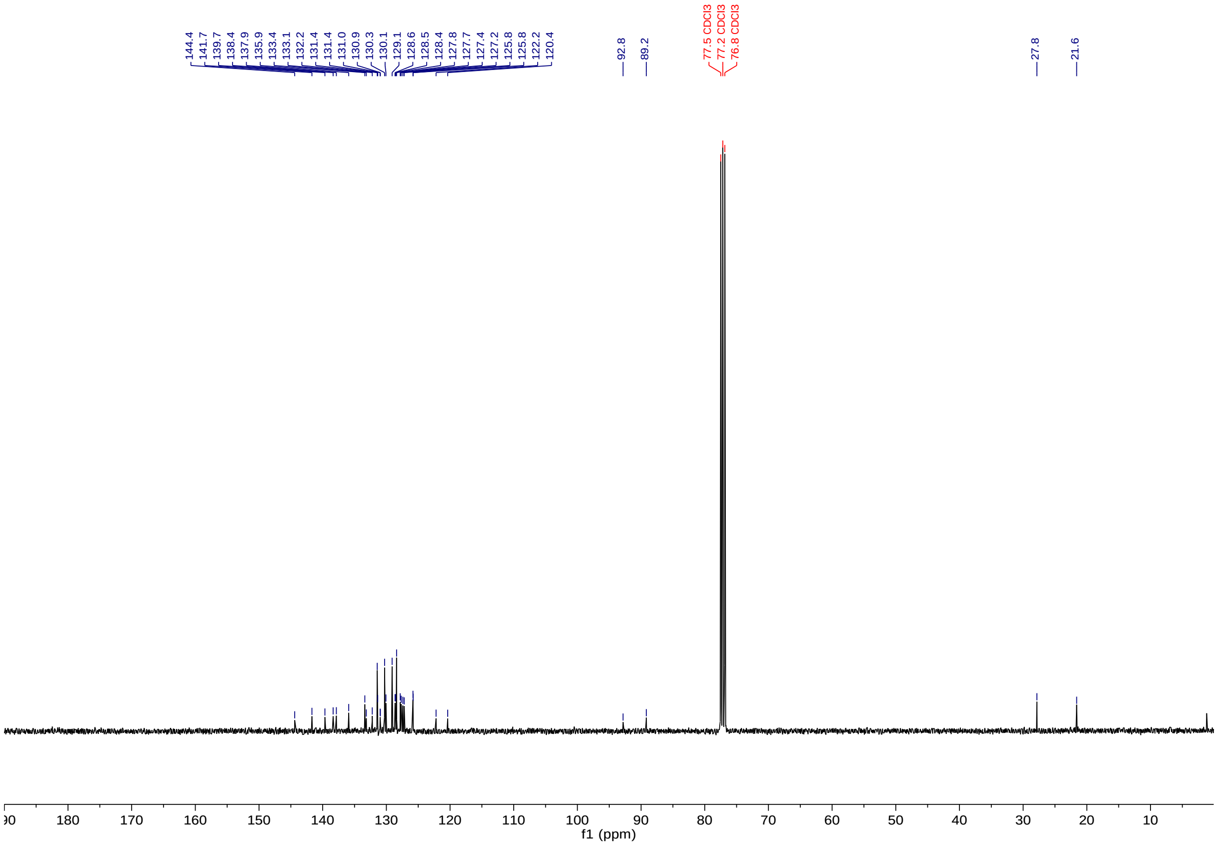

1H NMR: (400 MHz, $CDCl_3$) **18i**

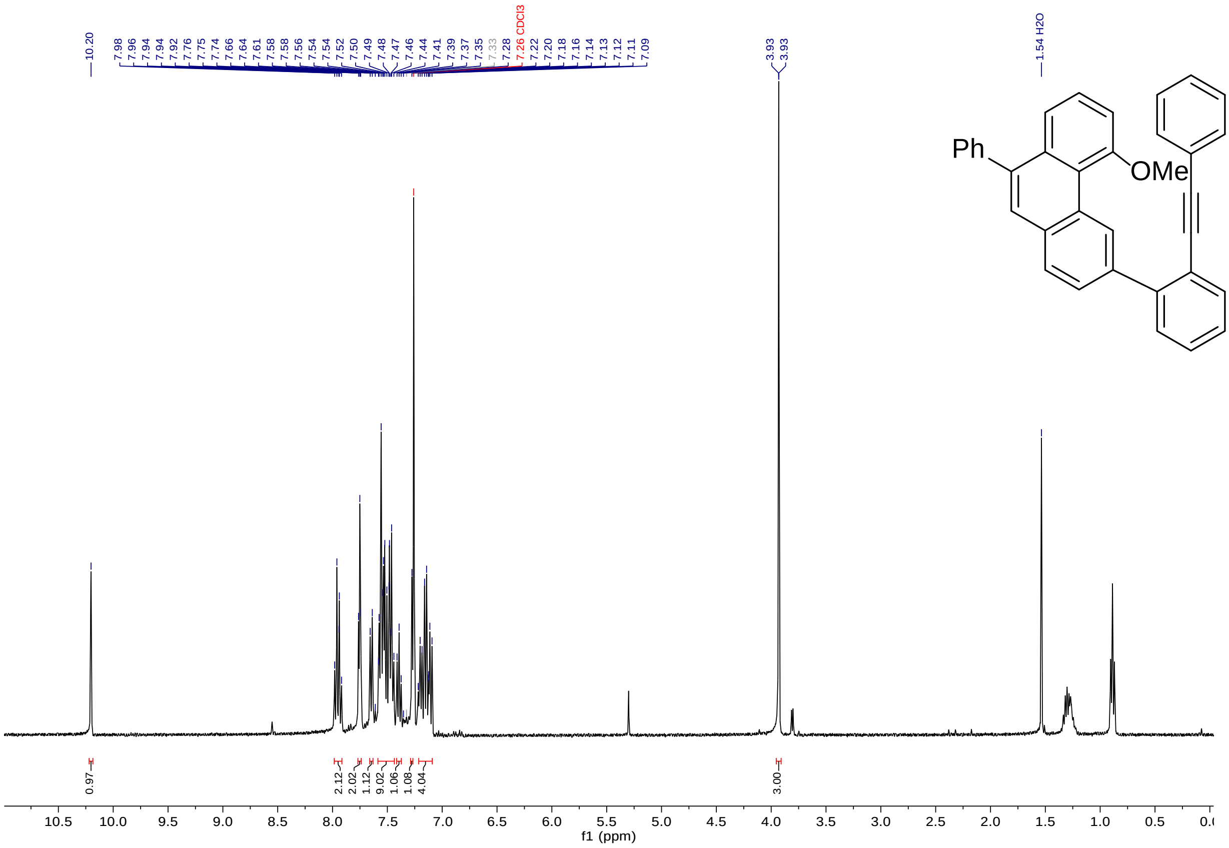

13C{H} NMR: (101 MHz, $CDCl_3$) **18i**

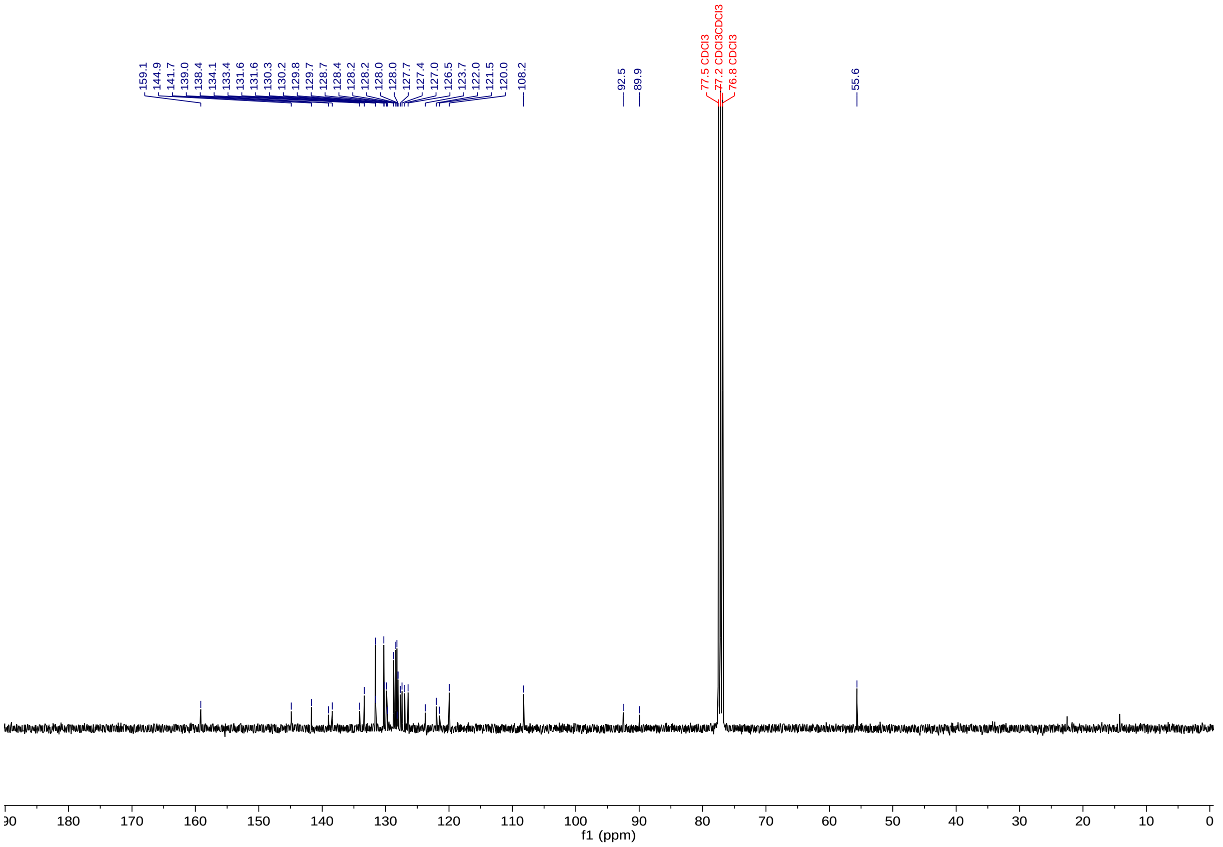

1H NMR: (400 MHz, $CDCl_3$) **18j**

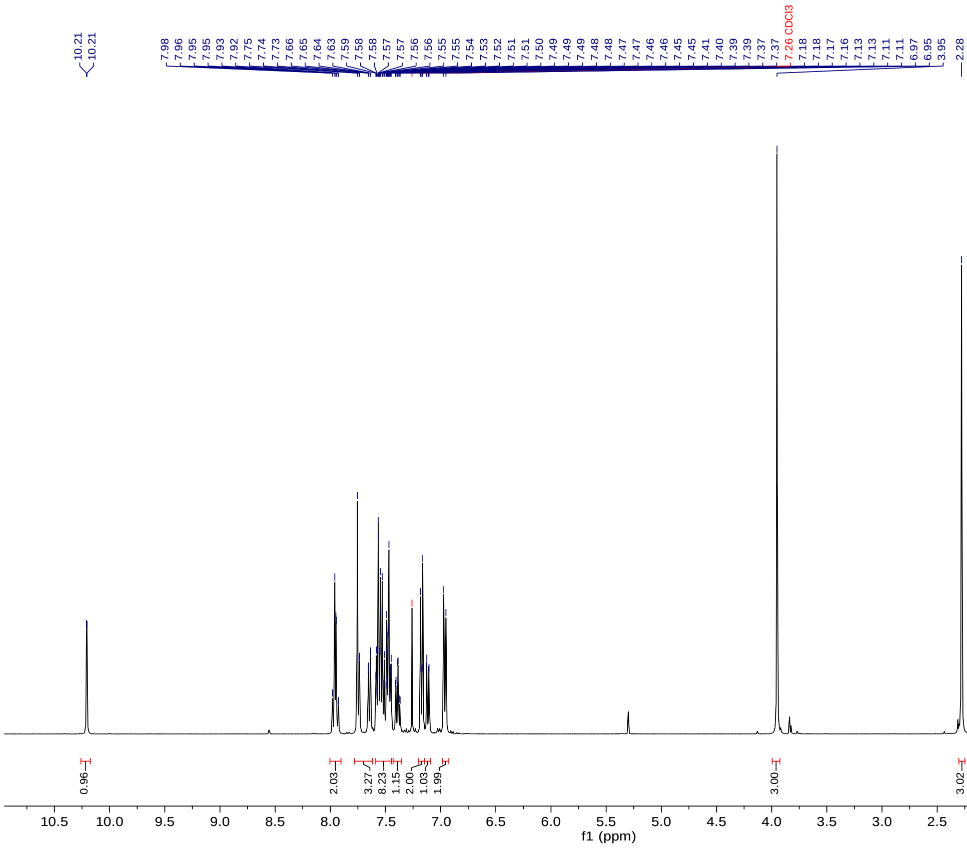

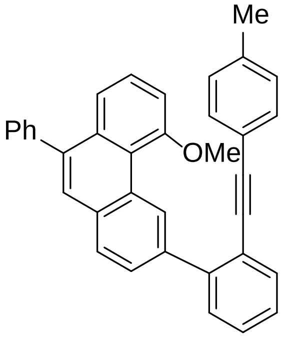

13C{H} NMR: (101 MHz, $CDCl_3$) **18i**

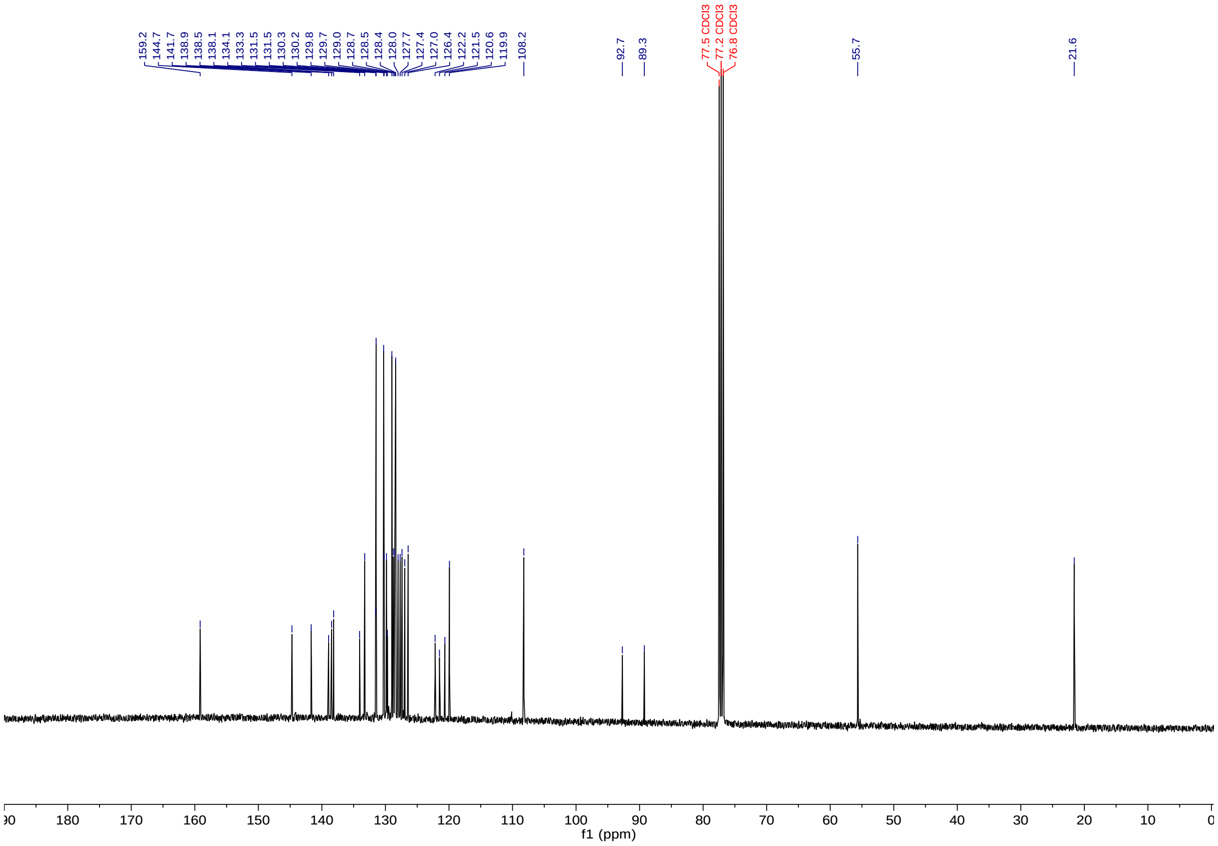

1H NMR: (400 MHz, $CDCl_3$) **20a**

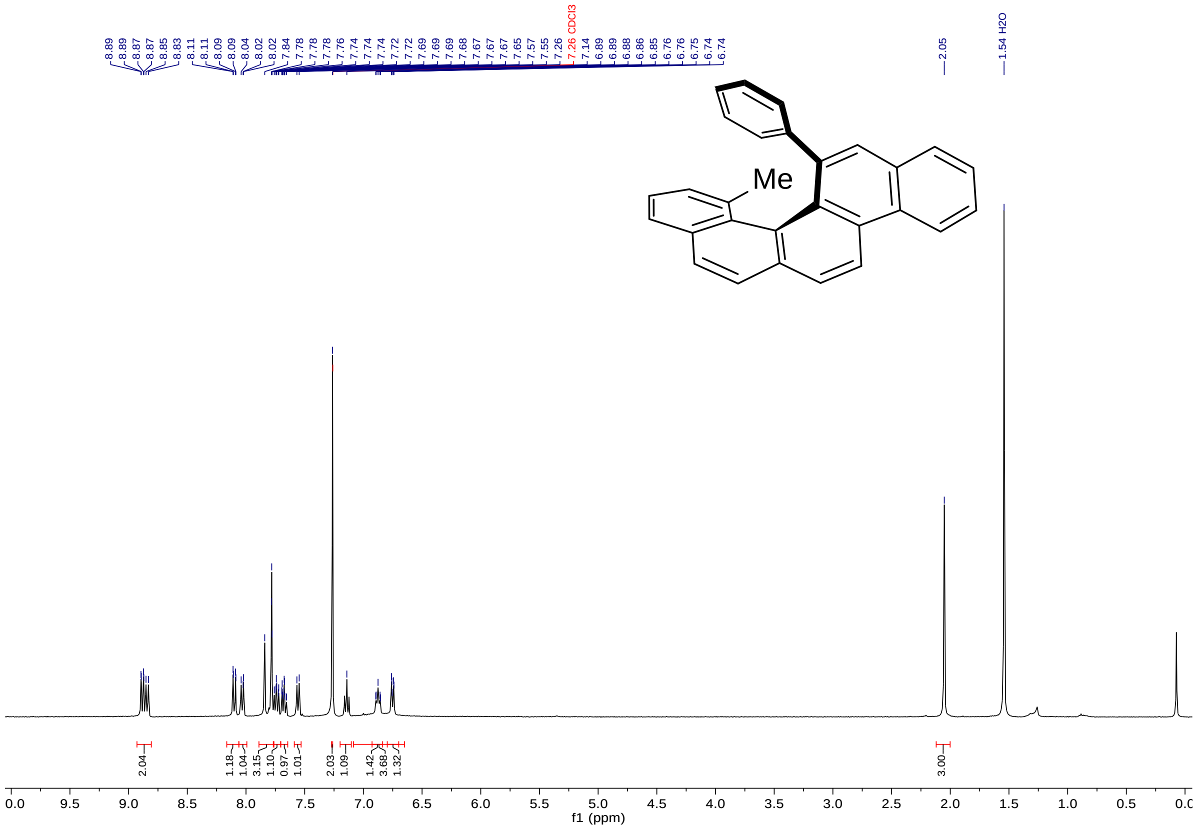

13C{H} NMR: (101 MHz, $CDCl_3$) **20a**

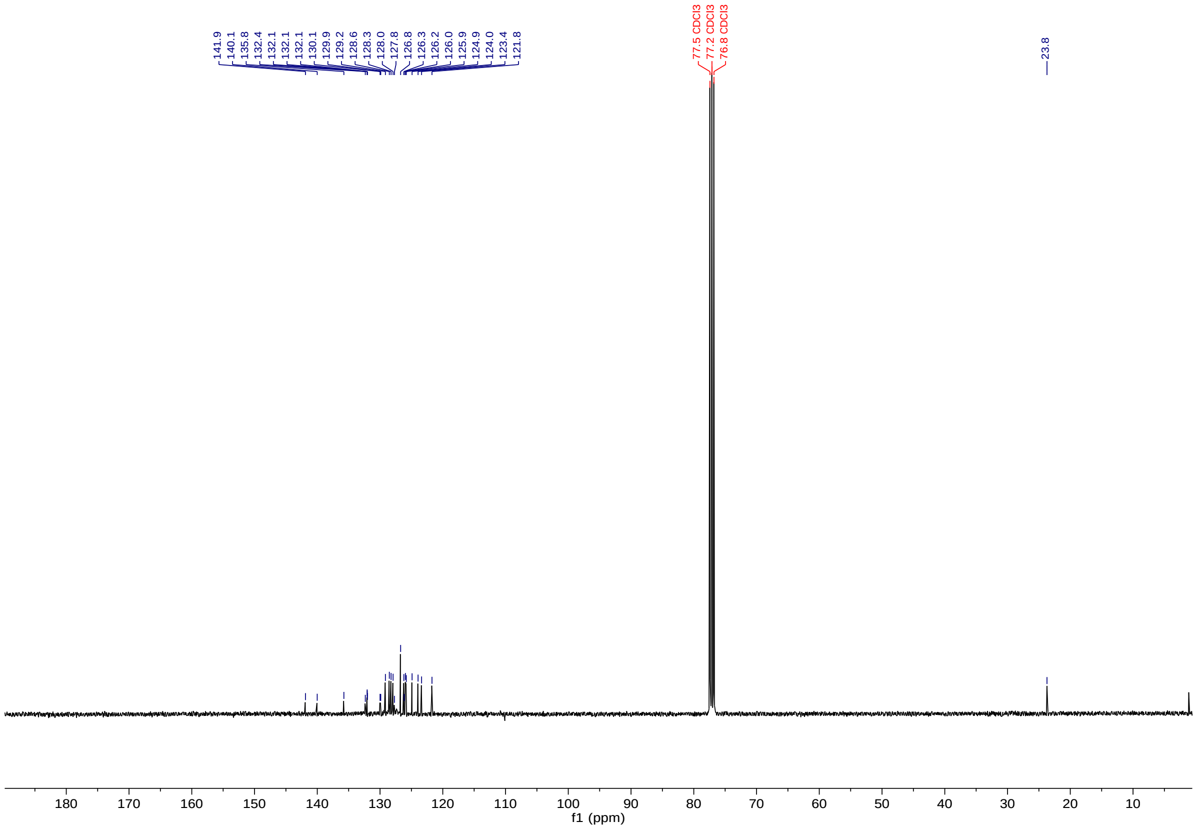

1H NMR: (400 MHz, $CDCl_3$) **20b**

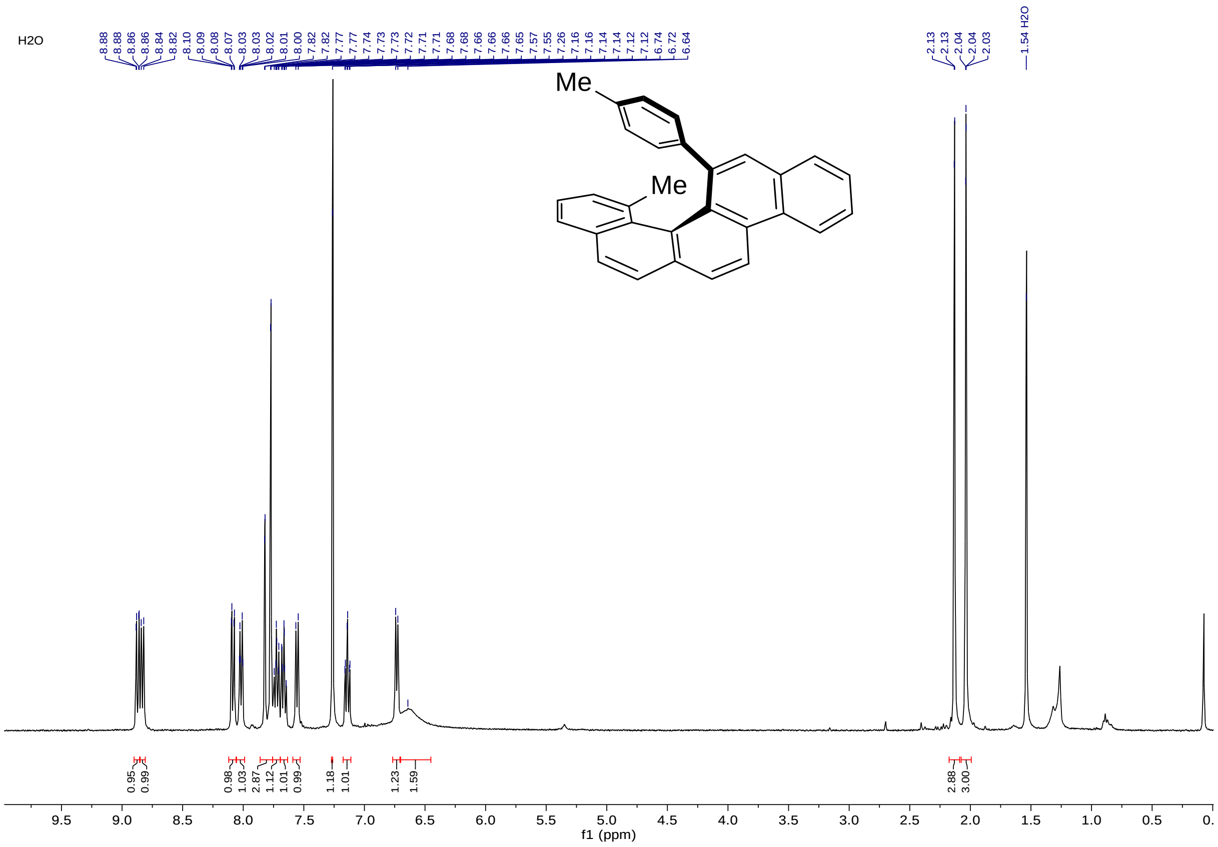

13C{H} NMR: (101 MHz, $CDCl_3$) **20b**

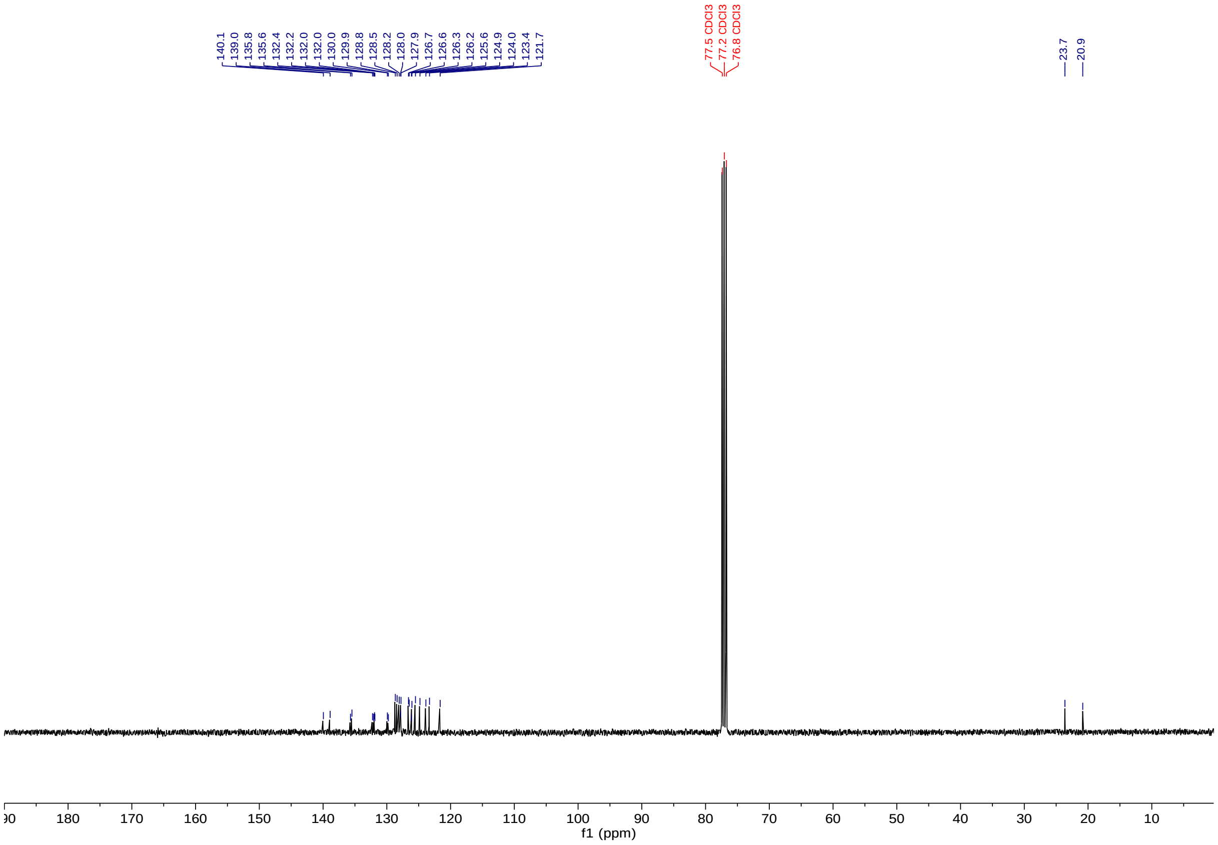

1H NMR: (400 MHz, $CDCl_3$) **20c**

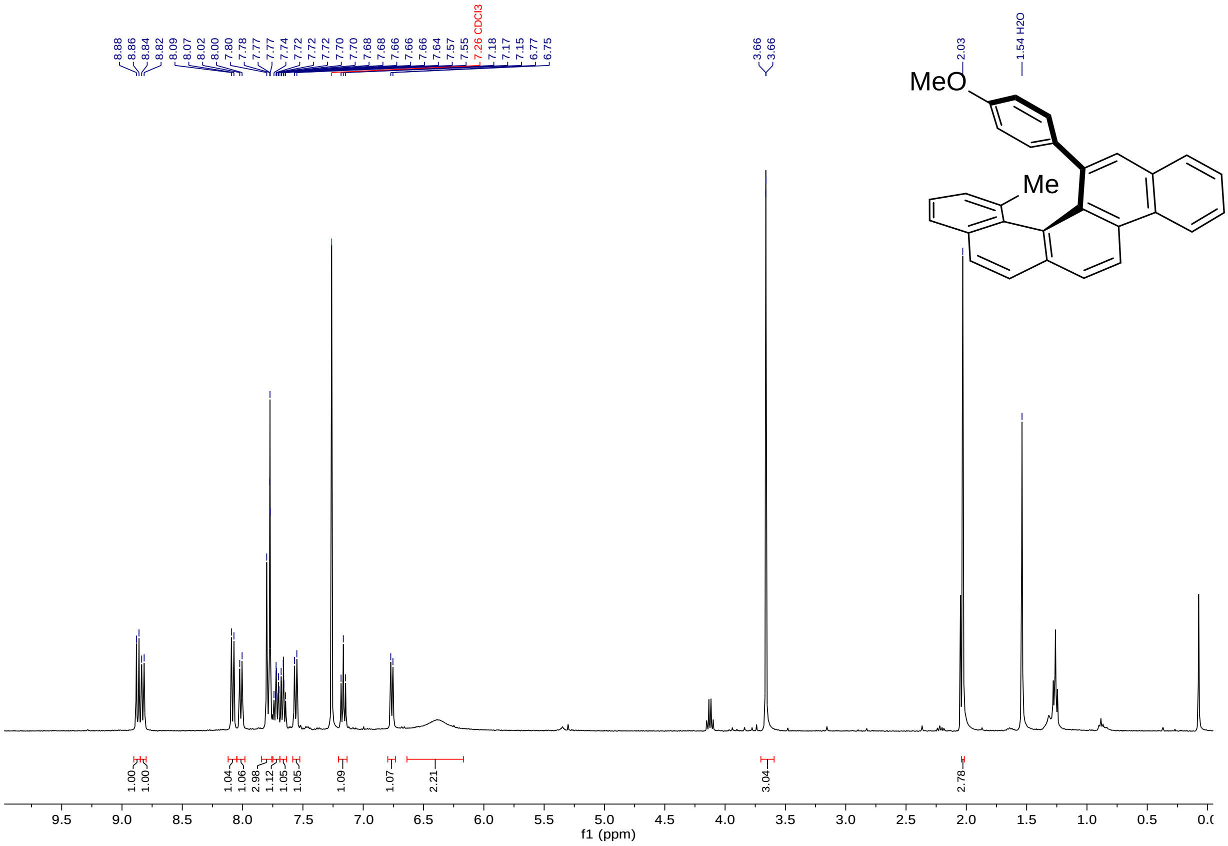

13C{H} NMR: (101 MHz, $CDCl_3$) **20c**

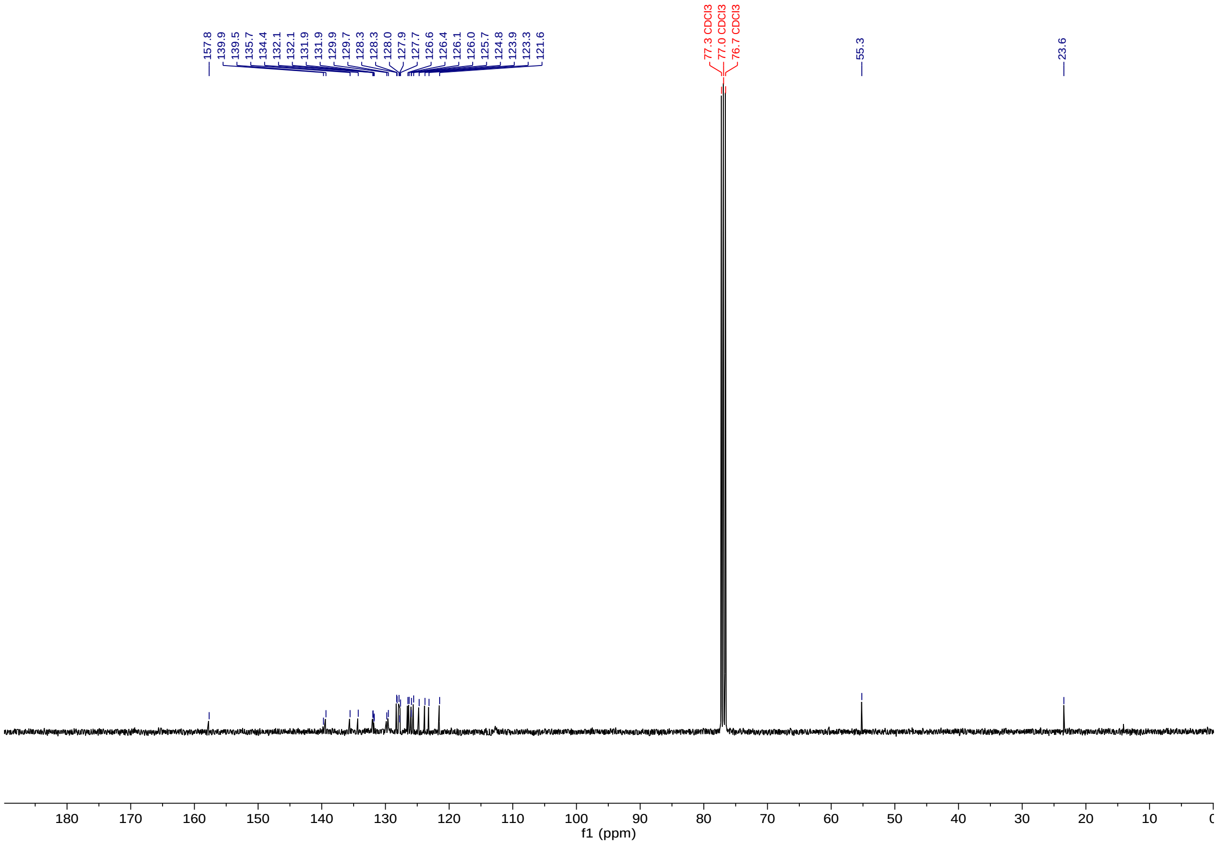

1H NMR: (400 MHz, $CDCl_3$) **20d**

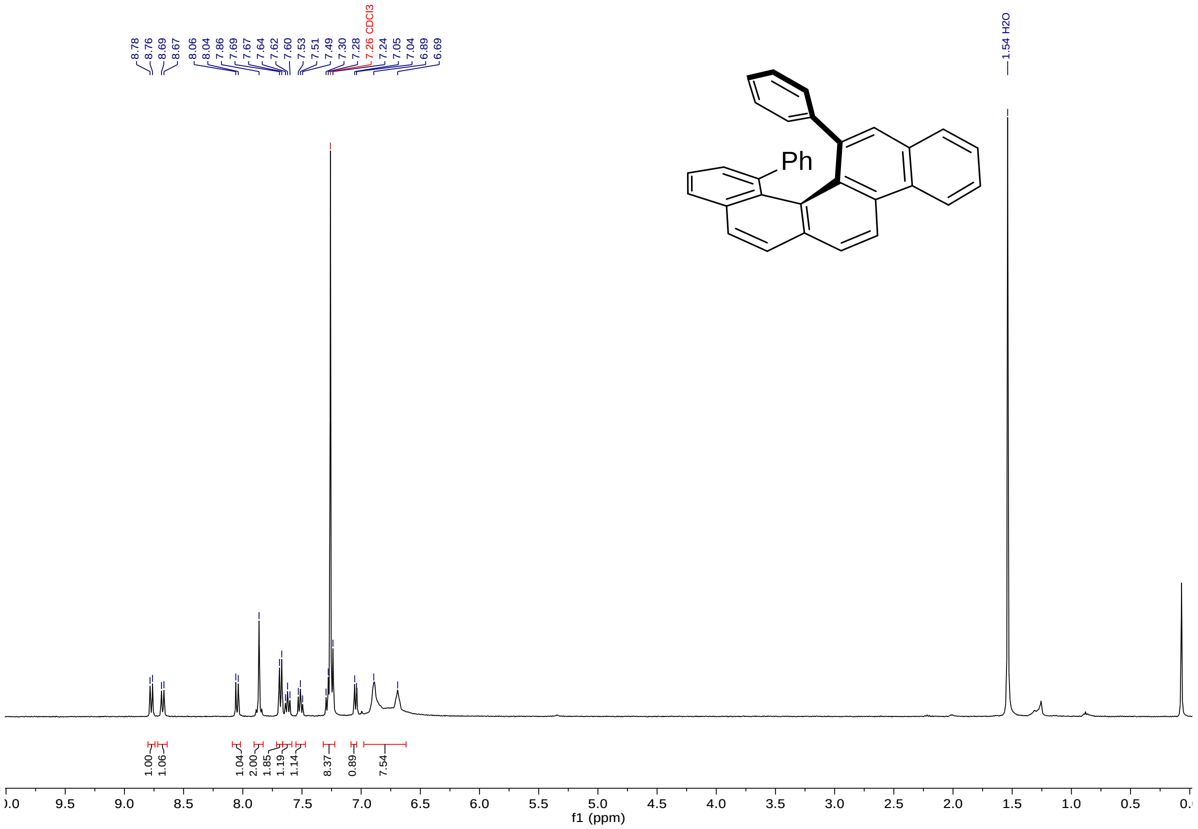

13C{H} NMR: (101 MHz, $CDCl_3$) **20d**

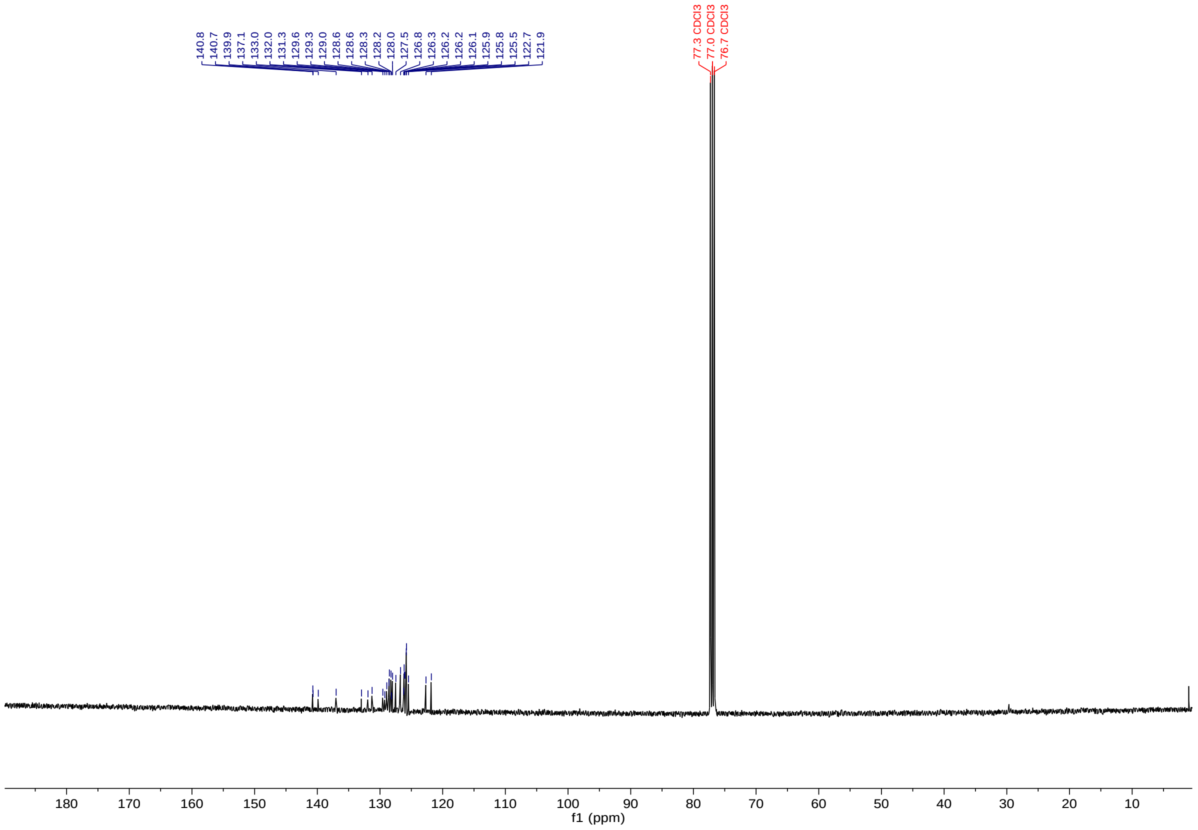

1H NMR: (400 MHz, $CDCl_3$) **20e**

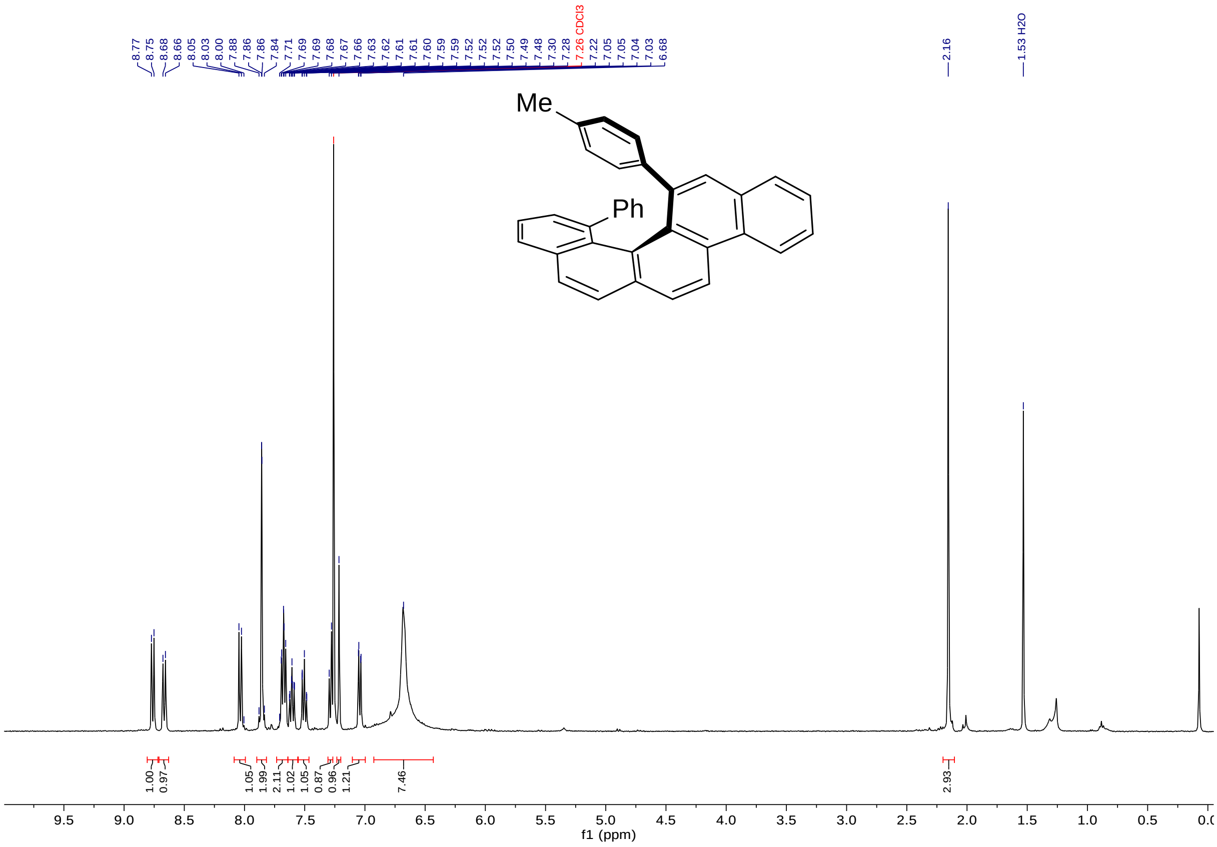

13C{H} NMR: (101 MHz, $CDCl_3$) **20e**

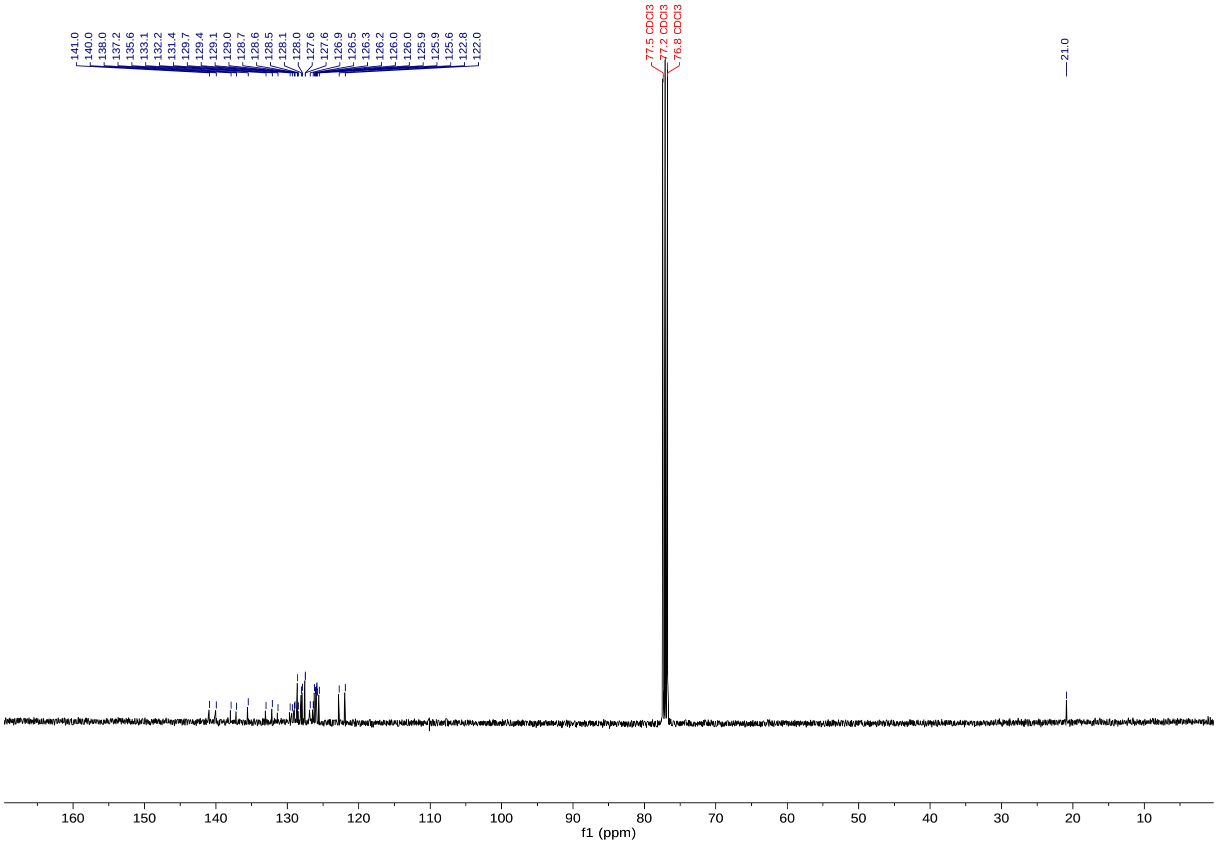

1H NMR: (400 MHz, $CDCl_3$) **20f**

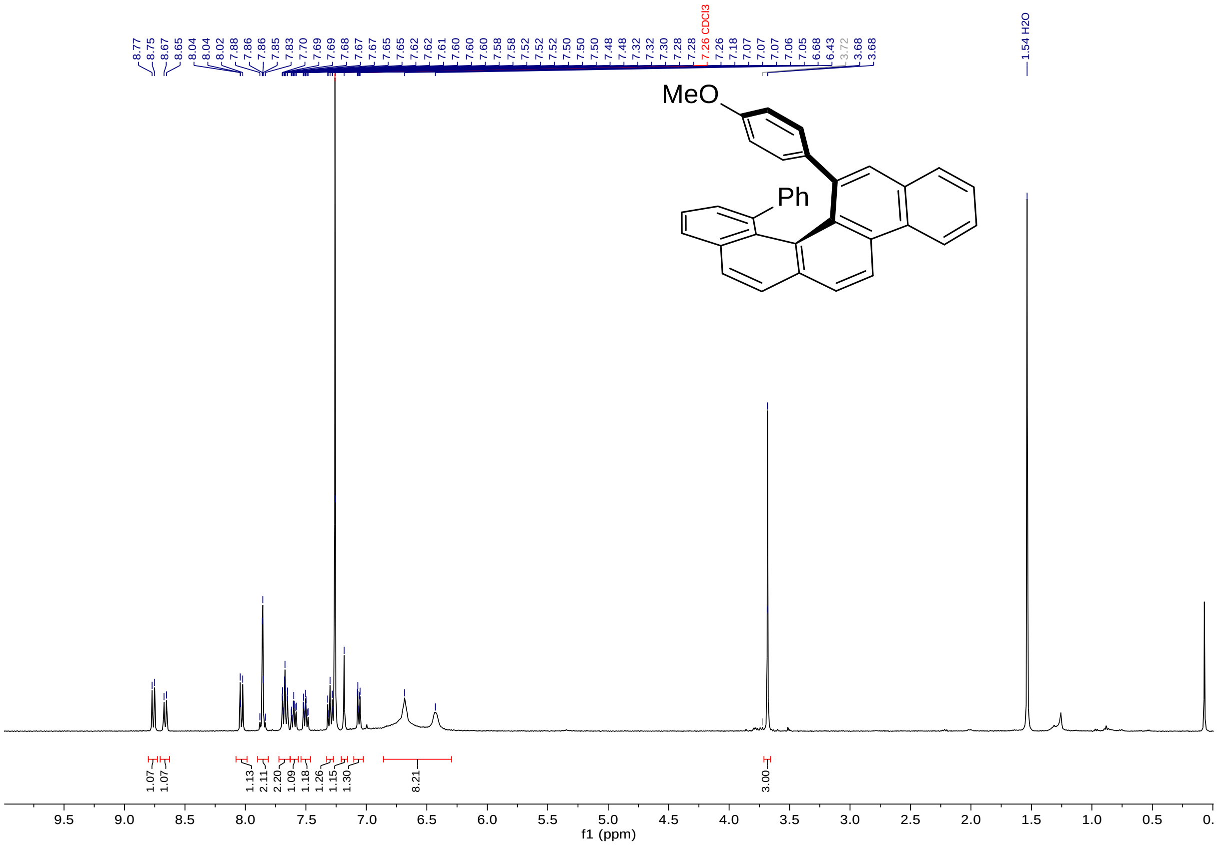

13C{H} NMR: (101 MHz, $CDCl_3$) **20f**

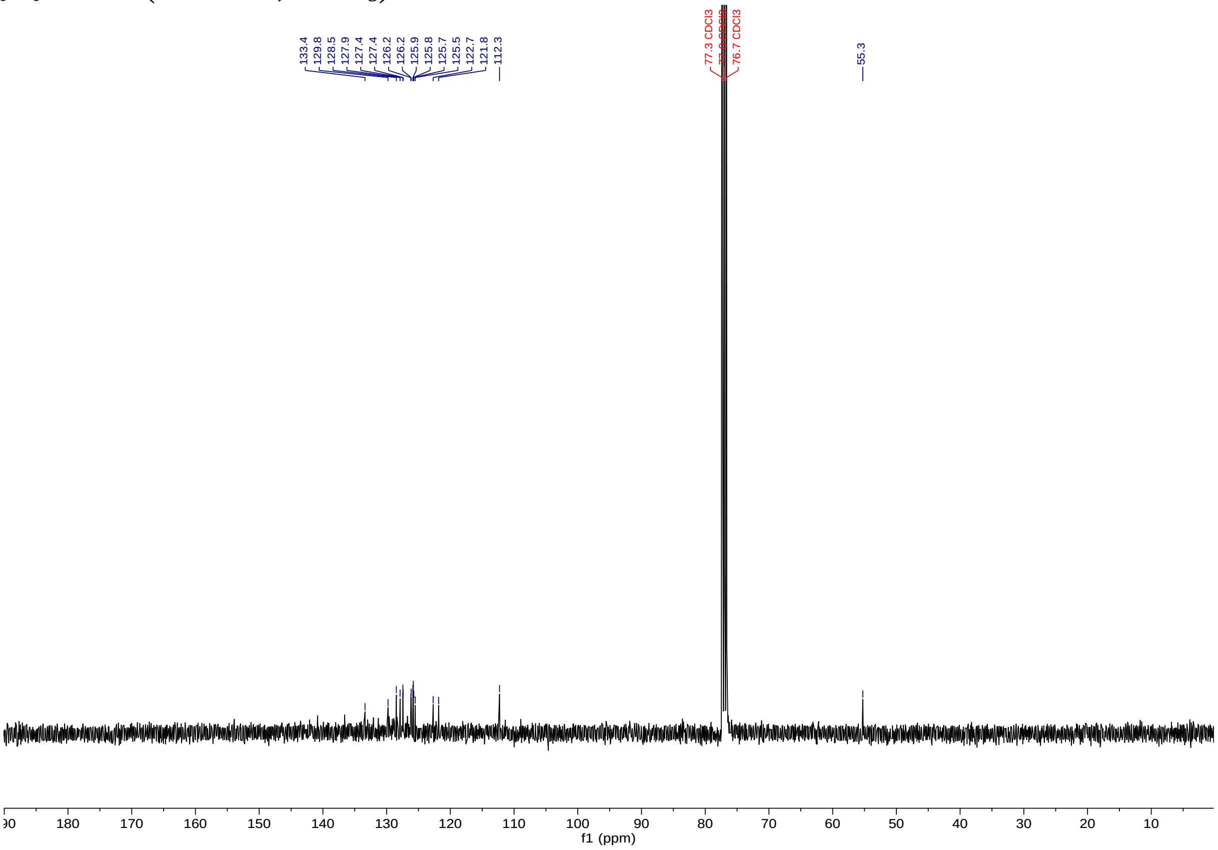

1H NMR: (500 MHz, $CDCl_3$) **20g,** expansion of crude spectrum.

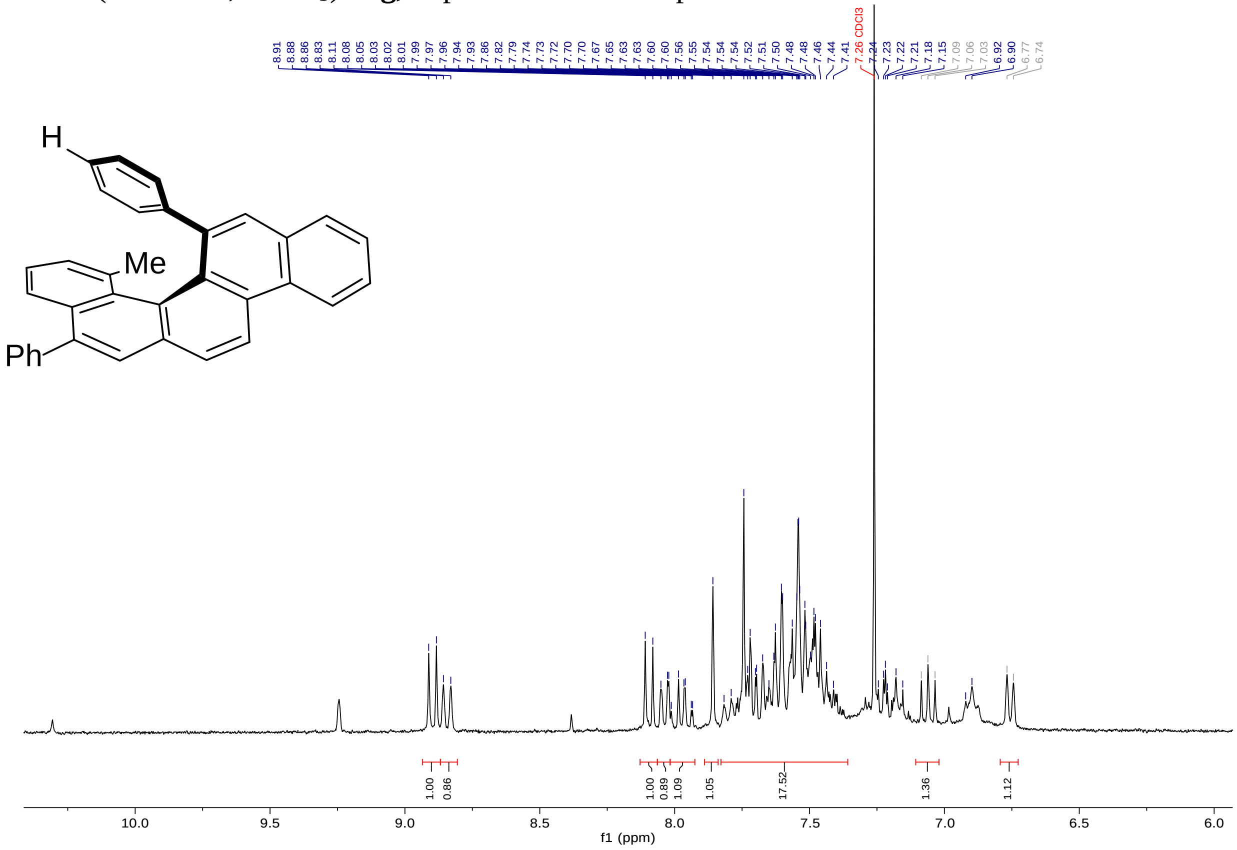

1H NMR: (500 MHz, $CDCl_3$) **20h**

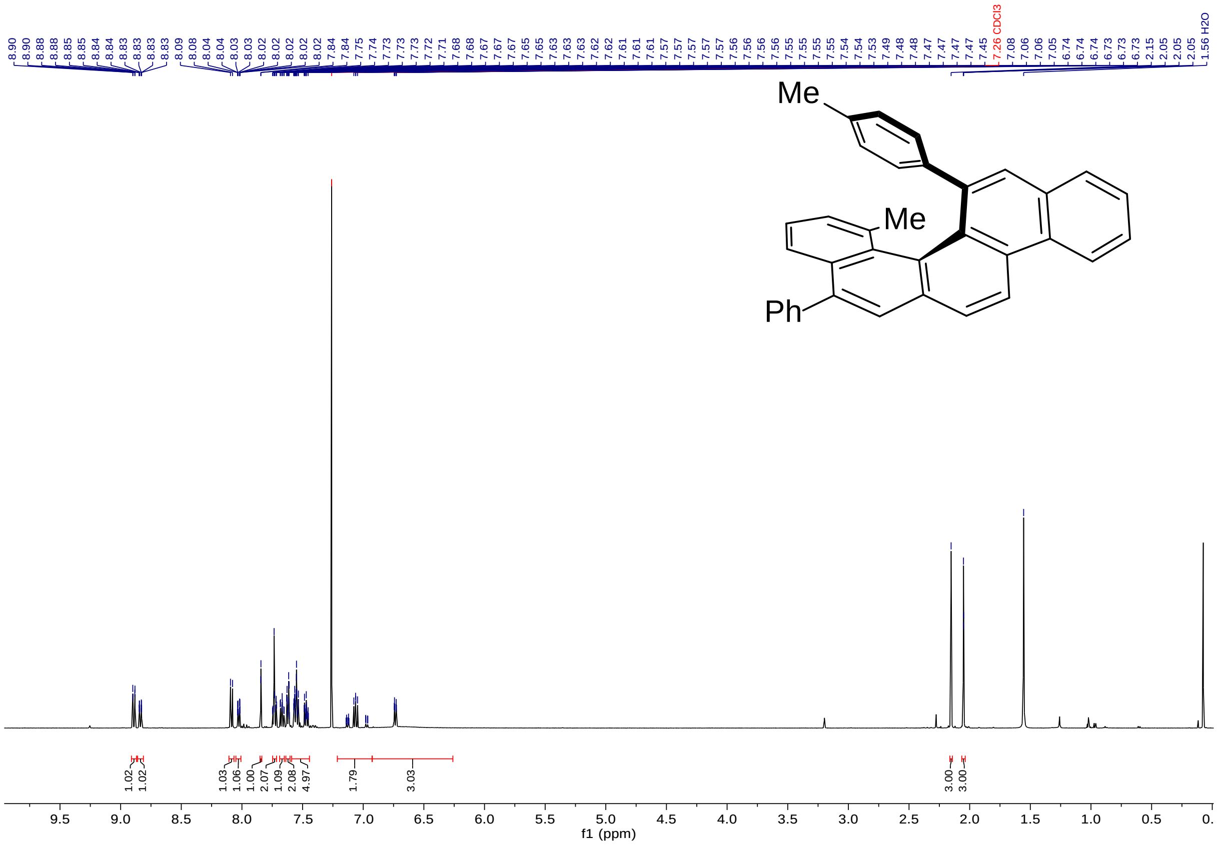

13C{H} NMR: (126 MHz, $CDCl_3$) **20h**

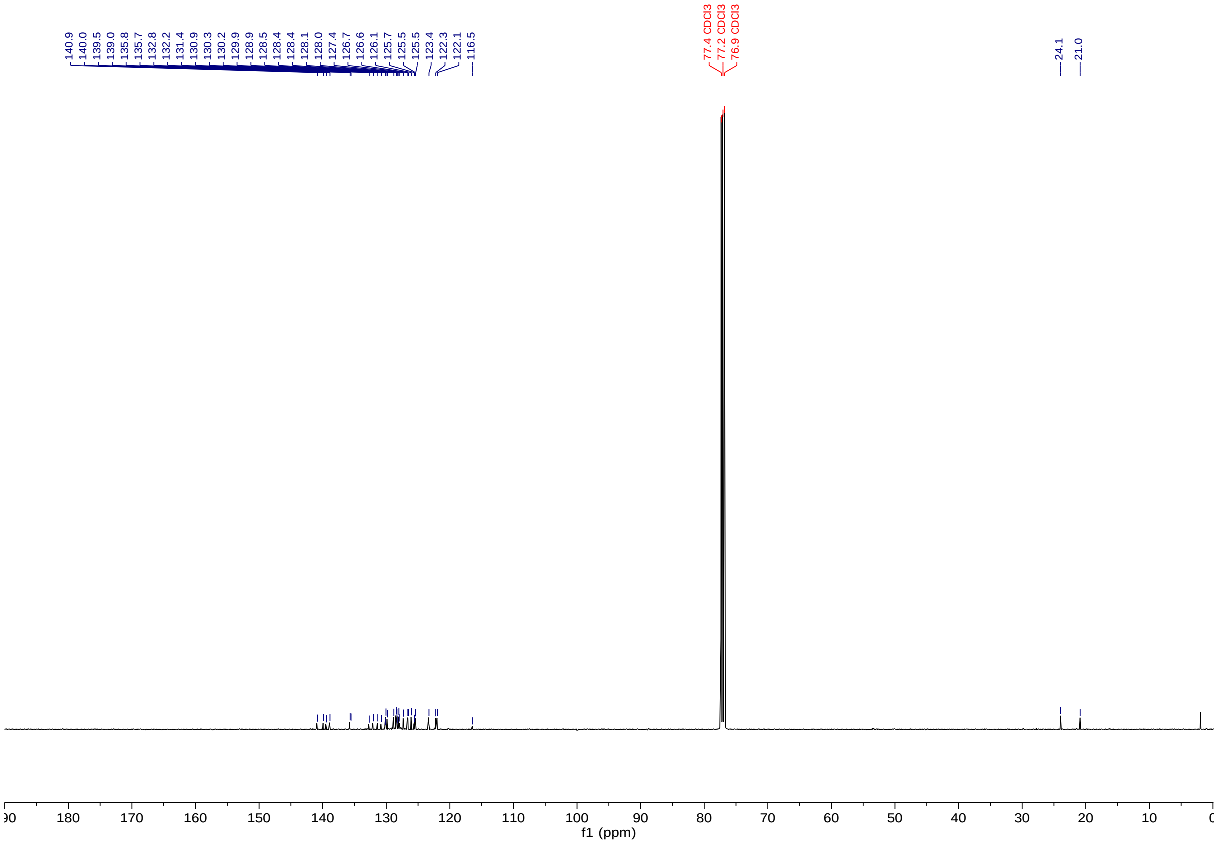

1H NMR: (500 MHz, $CDCl_3$) **20i**

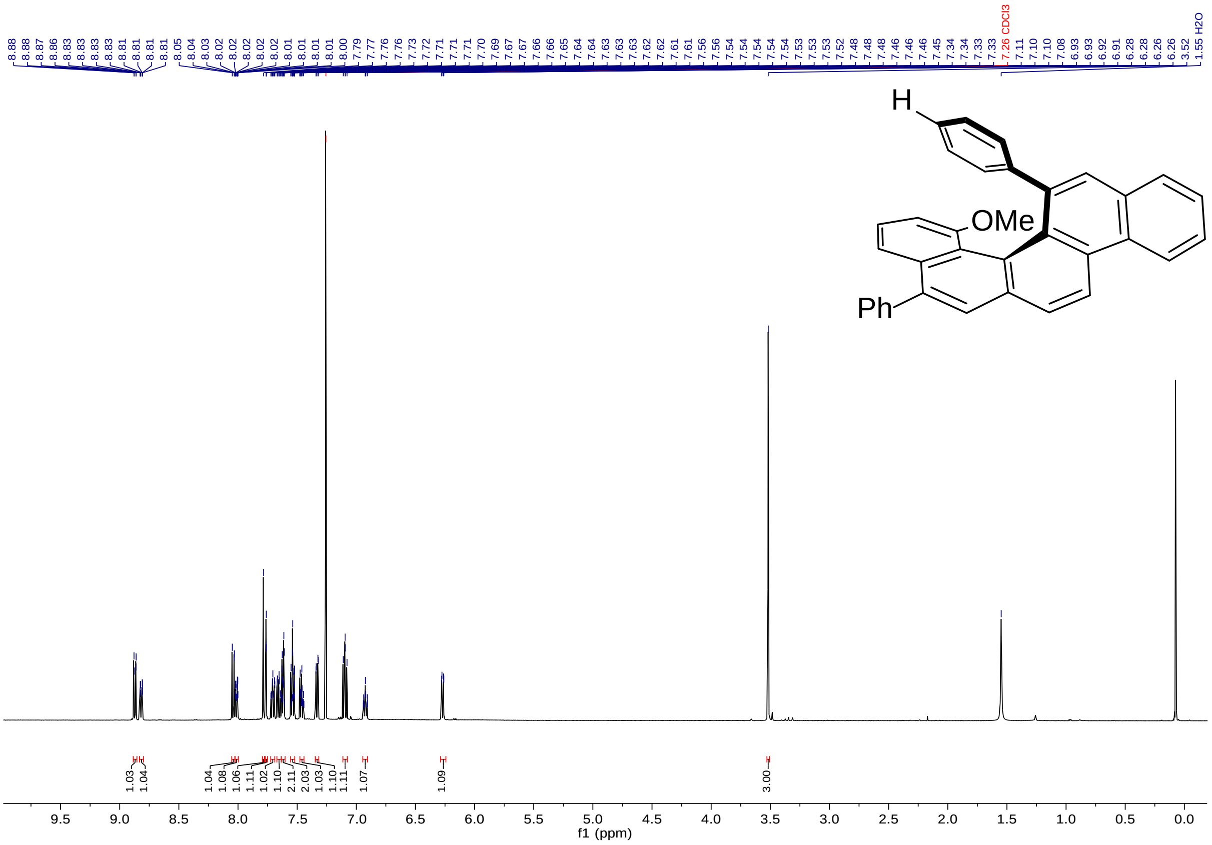

13C{H} NMR: (126 MHz, $CDCl_3$) **20i**

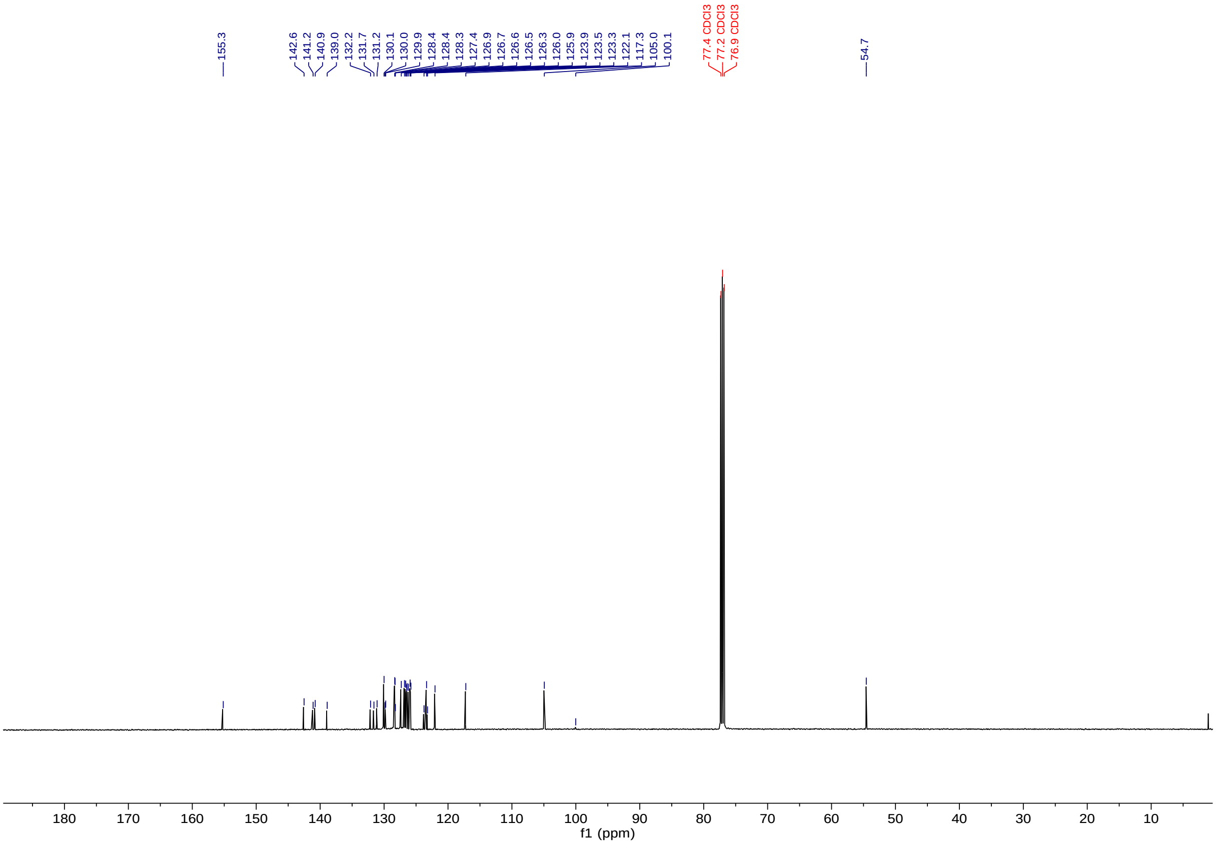

1H NMR: (500 MHz, $CDCl_3$) **20j**

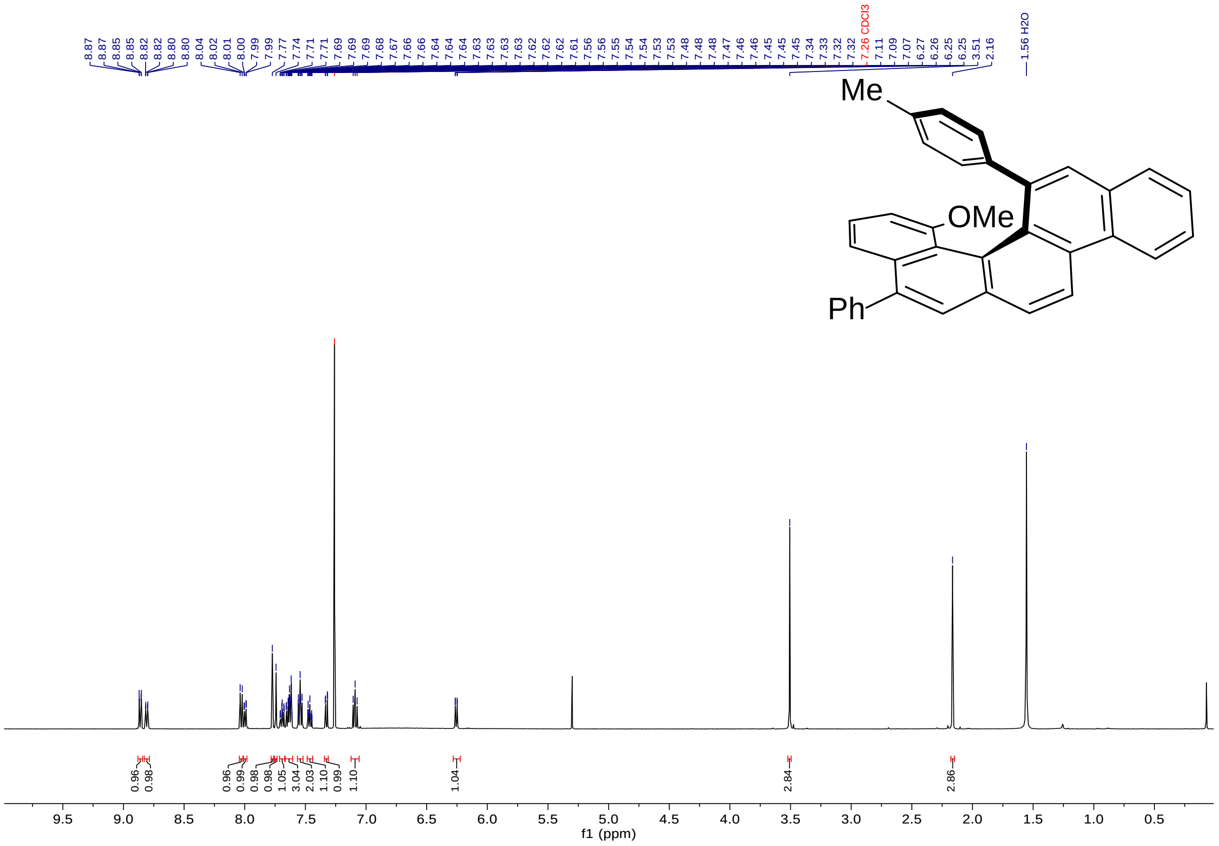

13C{H} NMR: (126 MHz, $CDCl_3$) **20j**

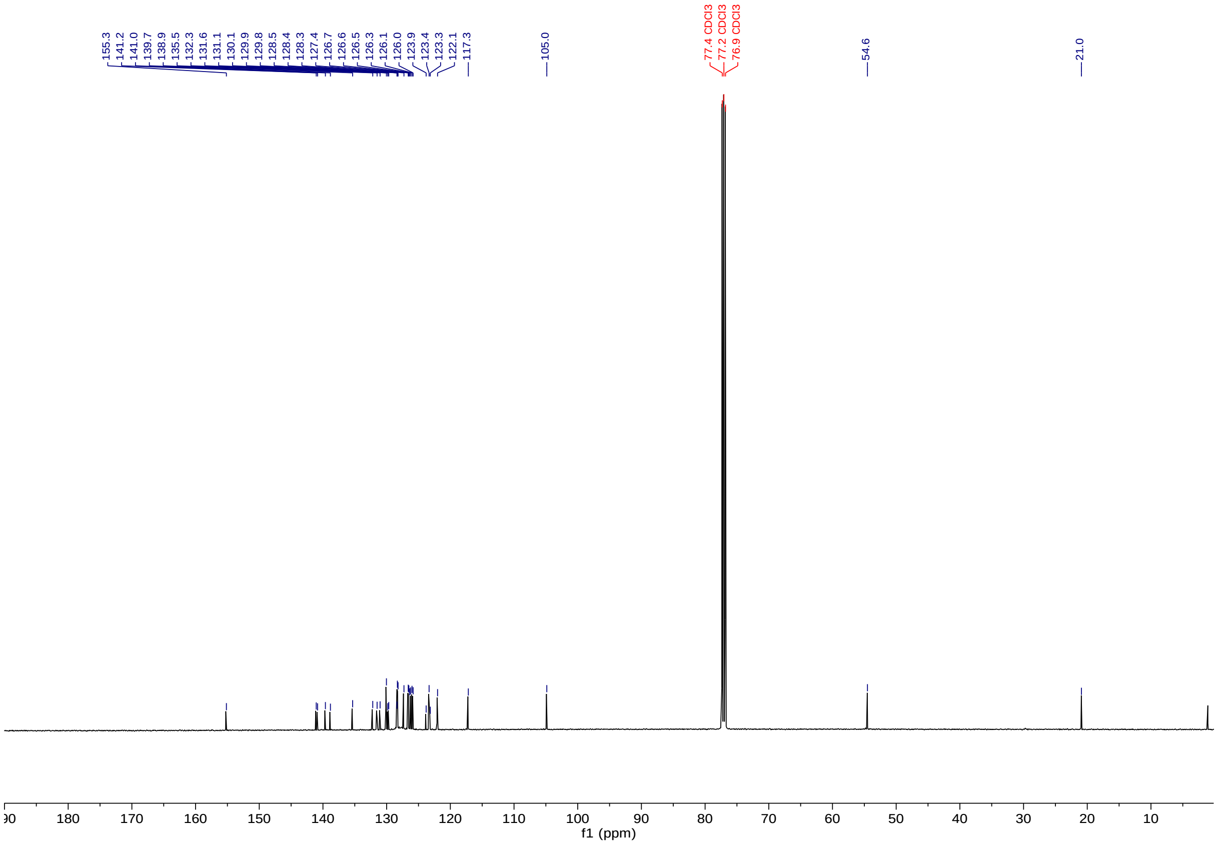

Single crystal X-ray diffraction analysis

Data collection was done on a *Bruker D8 Venture* four-circle-diffractometer from *Bruker AXS GmbH*; used detector: *Photon II* from *Bruker AXS GmbH*; used X-ray sources: microfocus *IµS* Cu/Mo from *Incoatec GmbH* with mirror optics *HELIOS* and single-hole collimator from *Bruker AXS GmbH*.
Used programs: *APEX3 Suite* (v2018.7-2) for data collection and therein integrated programs *SAINT* V8.38A (Integration) und *SADABS* 2016/2 (Absorption correction) from *Bruker AXS GmbH*; structure solution was done with *SHELXT*, refinement with *SHELXL*-2018/3 (Both: G.M. Sheldrick, *Acta Cryst*. **2008,** *A64*, 112-122.); $OLEX^2$ was used for data finalization (O.V. Dolomanov, L.J. Bourhis, R.J Gildea, J.A.K. Howard, H. Puschmann, *J. Appl. Cryst*. **2009,** *42*, 339-341.).
Special Utilities: *SMZ1270* stereomicroscope from *Nikon Metrology GmbH* was used for sample preparation; crystals were mounted on *MicroMounts* or *MicroLoops* from *MiTeGen* in NVH oil; for sensitive samples the *X-TEMP 2 System* was used for picking of crystals (T. Kottke, D. Stalke, *J. Appl. Cryst*. **1993**, *26*, 615-619.); crystals were cooled to given temperature with *Cryostream 800* from *Oxford Cryosystems*

Substance Identifier	CCDC-Nr.
**1a**	1958110
**2g**	1958111
**3c**	1958146
**7a**	1958107
**8a**	1958108
**15d**	1958109
**20j**	1958106
**20i**	1958105

X-Ray data of compound 1a

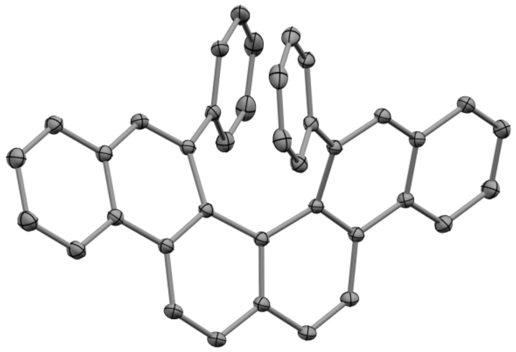

Empirical formula	$C_{38}H_{24}$
Formula weight	480.57
Temperature/K	100.01
Crystal system	orthorhombic
Space group	Pccn
a/Å	16.2522(6)
b/Å	32.6419(11)
c/Å	9.4624(4)
α/°	90
β/°	90
γ/°	90
Volume/Å^3	5019.8(3)
Z	8
ρ_{calc}g/cm^3	1.272
μ/mm^{-1}	0.072
F(000)	2016.0
Crystal size/mm^3	0.858 × 0.518 × 0.428
Radiation	MoKα (λ = 0.71073)
2Θ range for data collection/°	5.136 to 65.188
Index ranges	-24 ≤ h ≤ 24, -46 ≤ k ≤ 49, -13 ≤ l ≤ 14
Reflections collected	109653
Independent reflections	9142 [R_{int} = 0.0576, R_{sigma} = 0.0195]
Data/restraints/parameters	9142/0/343
Goodness-of-fit on F^2	1.046
Final R indexes [I>=2σ (I)]	R_1 = 0.0395, wR_2 = 0.1148
Final R indexes [all data]	R_1 = 0.0469, wR_2 = 0.1195
Largest diff. peak/hole / e Å^{-3}	0.46/-0.19

X-Ray data of compound 2g

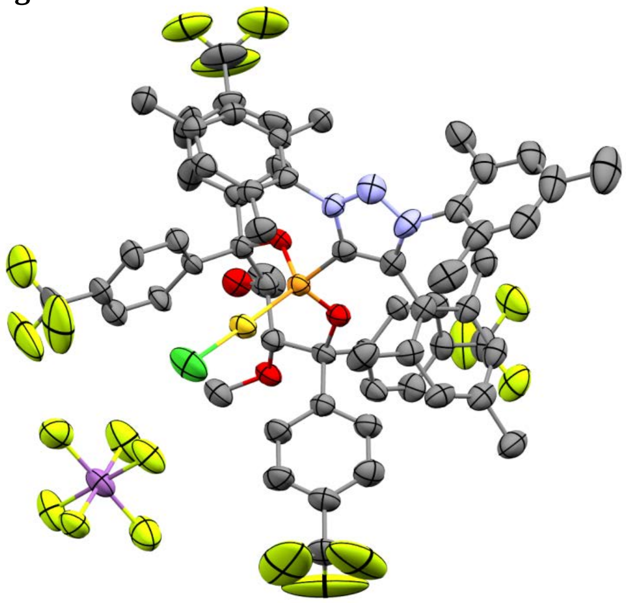

Empirical formula	C69H71AuCl3F18N3O4PSb
Formula weight	1804.32
Temperature/K	149.94
Crystal system	monoclinic
Space group	P21
a/Å	11.8820(7)
b/Å	24.5258(17)
c/Å	12.6105(8)
α/°	90
β/°	97.443(2)
γ/°	90
Volume/Å3	3643.9(4)
Z	2
ρcalcg/cm3	1.644
μ/mm-1	2.608
F(000)	1792.0
Crystal size/mm3	0.25 × 0.229 × 0.066
Radiation	MoKα (λ = 0.71073)
2Θ range for data collection/°	4.432 to 59.414
Index ranges	-16 ≤ h ≤ 15, -34 ≤ k ≤ 34, -17 ≤ l ≤ 16
Reflections collected	49543
Independent reflections	20178 [Rint = 0.0251, Rsigma = 0.0356]
Data/restraints/parameters	20178/127/905
Goodness-of-fit on F2	1.039
Final R indexes [I>=2σ (I)]	R1 = 0.0524, wR2 = 0.1384
Final R indexes [all data]	R1 = 0.0638, wR2 = 0.1457
Largest diff. peak/hole / e Å-3	1.88/-2.01
Flack parameter	-0.011(2)

X-Ray data of compound 3c

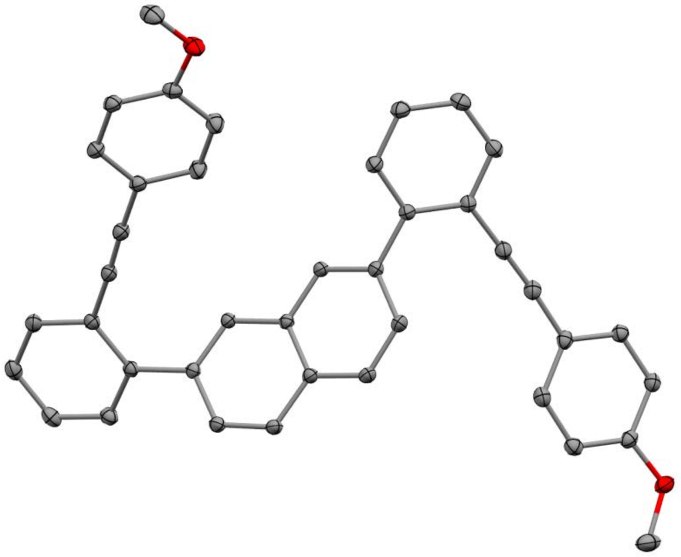

Empirical formula	$C_{42.5}H_{34}O_2$
Formula weight	576.70
Temperature/K	100.0
Crystal system	monoclinic
Space group	C2/c
a/Å	30.818(3)
b/Å	7.6358(6)
c/Å	27.269(2)
α/°	90
β/°	105.545(3)
γ/°	90
Volume/Å^3	6182.2(9)
Z	8
ρ_{calc}g/cm^3	1.239
μ/mm^{-1}	0.074
F(000)	2440.0
Crystal size/mm^3	0.274 × 0.266 × 0.078
Radiation	MoKα (λ = 0.71073)
2Θ range for data collection/°	4.658 to 65.15
Index ranges	$-46 \le h \le 46$, $-11 \le k \le 11$, $-40 \le l \le 41$
Reflections collected	46169
Independent reflections	11217 [R_{int} = 0.0229, R_{sigma} = 0.0214]
Data/restraints/parameters	11217/54/419
Goodness-of-fit on F^2	1.033
Final R indexes [I>=2σ (I)]	R_1 = 0.0414, wR_2 = 0.1153
Final R indexes [all data]	R_1 = 0.0477, wR_2 = 0.1208
Largest diff. peak/hole / e Å^{-3}	0.50/-0.23

X-Ray data of compound 7a

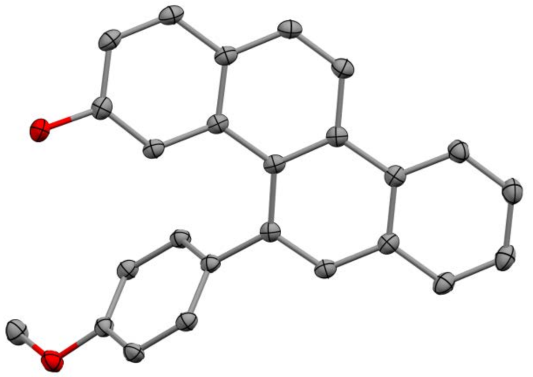

Empirical formula	$C_{25}H_{18}O_2$
Formula weight	350.39
Temperature/K	99.98
Crystal system	monoclinic
Space group	$P2_1/c$
a/Å	19.5663(14)
b/Å	5.9016(5)
c/Å	15.0828(11)
α/°	90
β/°	101.097(2)
γ/°	90
Volume/Å^3	1709.1(2)
Z	4
ρ_{calc}g/cm^3	1.362
μ/mm^{-1}	0.085
F(000)	736.0
Crystal size/mm^3	0.534 × 0.152 × 0.143
Radiation	MoKα (λ = 0.71073)
2Θ range for data collection/°	6.27 to 55.822
Index ranges	$-25 \le h \le 25$, $0 \le k \le 7$, $0 \le l \le 19$
Reflections collected	4324
Independent reflections	4324 [R_{int} = ?, R_{sigma} = 0.0206]
Data/restraints/parameters	4324/0/249
Goodness-of-fit on F^2	1.063
Final R indexes [I>=2σ (I)]	R_1 = 0.0428, wR_2 = 0.1183
Final R indexes [all data]	R_1 = 0.0457, wR_2 = 0.1217
Largest diff. peak/hole / e Å^{-3}	0.32/-0.23

X-Ray data of compound 8a

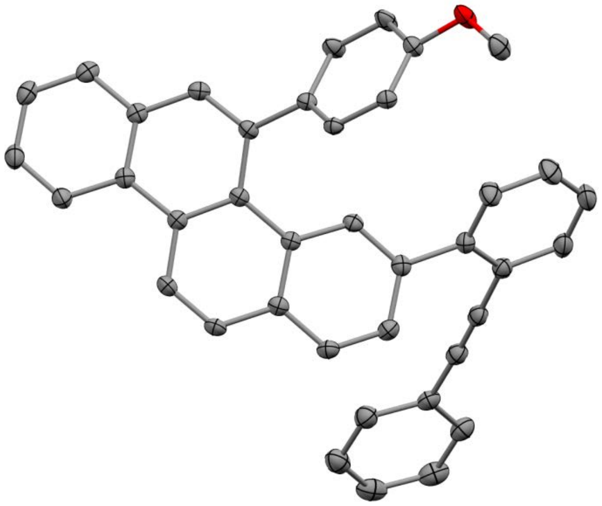

Empirical formula	$C_{39}H_{26}O$
Formula weight	510.60
Temperature/K	99.99
Crystal system	orthorhombic
Space group	Pbca
a/Å	12.9278(2)
b/Å	13.7658(2)
c/Å	31.1890(6)
α/°	90
β/°	90
γ/°	90
Volume/Å^3	5550.44(16)
Z	8
ρ_{calc}g/cm^3	1.222
μ/mm^{-1}	0.072
F(000)	2144.0
Crystal size/mm^3	0.427 × 0.192 × 0.17
Radiation	MoKα (λ = 0.71073)
2Θ range for data collection/°	4.516 to 57.524
Index ranges	$-17 \le h \le 17$, $-18 \le k \le 18$, $-42 \le l \le 42$
Reflections collected	103018
Independent reflections	7185 [R_{int} = 0.0396, R_{sigma} = 0.0173]
Data/restraints/parameters	7185/0/362
Goodness-of-fit on F^2	1.057
Final R indexes [I>=2σ (I)]	R_1 = 0.0434, wR_2 = 0.1056
Final R indexes [all data]	R_1 = 0.0509, wR_2 = 0.1114
Largest diff. peak/hole / e Å^{-3}	0.28/-0.24

X-Ray data of compound 15c

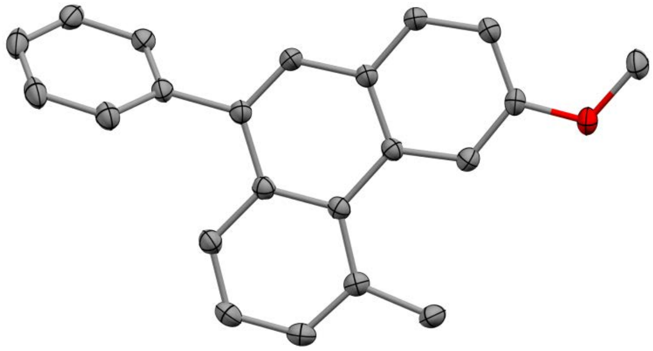

Empirical formula	$C_{22}H_{18}O$
Formula weight	298.36
Temperature/K	100.01
Crystal system	monoclinic
Space group	$P2_1/c$
a/Å	7.7672(9)
b/Å	9.2792(14)
c/Å	20.927(3)
α/°	90
β/°	92.834(6)
γ/°	90
Volume/Å^3	1506.4(4)
Z	4
ρ_{calc}g/cm^3	1.316
μ/mm^{-1}	0.079
F(000)	632.0
Crystal size/mm^3	0.522 × 0.391 × 0.108
Radiation	MoKα (λ = 0.71073)
2Θ range for data collection/°	4.802 to 57.456
Index ranges	$-10 \le h \le 9$, $-12 \le k \le 12$, $-28 \le l \le 28$
Reflections collected	47090
Independent reflections	3906 [R_{int} = 0.0262, R_{sigma} = 0.0132]
Data/restraints/parameters	3906/0/210
Goodness-of-fit on F^2	1.092
Final R indexes [I>=2σ (I)]	R_1 = 0.0418, wR_2 = 0.1207
Final R indexes [all data]	R_1 = 0.0447, wR_2 = 0.1234
Largest diff. peak/hole / e Å^{-3}	0.40/-0.18

X-Ray data of compound 20j

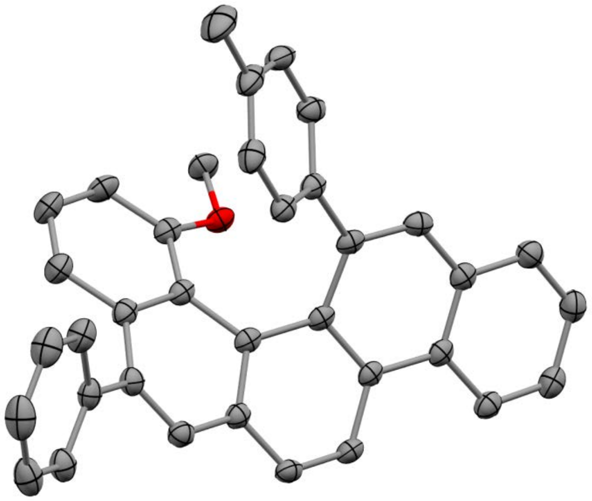

Empirical formula	$C_{36}H_{26}O$
Formula weight	474.57
Temperature/K	100.0
Crystal system	monoclinic
Space group	$P2_1$
a/Å	9.1839(9)
b/Å	7.5120(8)
c/Å	18.0480(18)
α/°	90
β/°	99.307(3)
γ/°	90
Volume/Å^3	1228.7(2)
Z	2
ρ_{calc}g/cm^3	1.283
μ/mm^{-1}	0.579
F(000)	500.0
Crystal size/mm^3	0.333 × 0.082 × 0.063
Radiation	CuKα (λ = 1.54178)
2Θ range for data collection/°	4.962 to 160.992
Index ranges	$-11 \leq h \leq 11$, $-9 \leq k \leq 8$, $-22 \leq l \leq 22$
Reflections collected	47880
Independent reflections	5164 [R_{int} = 0.0297, R_{sigma} = 0.0132]
Data/restraints/parameters	5164/1/402
Goodness-of-fit on F^2	1.024
Final R indexes [I>=2σ (I)]	R_1 = 0.0325, wR_2 = 0.0855
Final R indexes [all data]	R_1 = 0.0330, wR_2 = 0.0860
Largest diff. peak/hole / e Å^{-3}	0.66/-0.14
Flack parameter	0.02(12)

X-Ray data of compound 20i

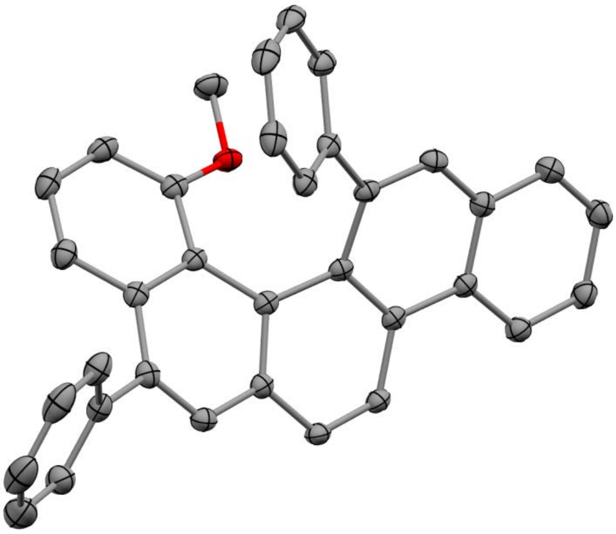

Empirical formula	$C_{35}H_{24}O$
Formula weight	460.54
Temperature/K	100.0
Crystal system	orthorhombic
Space group	$P2_12_12_1$
a/Å	7.4517(5)
b/Å	16.7581(8)
c/Å	19.7465(8)
α/°	90
β/°	90
γ/°	90
Volume/$Å^3$	2465.9(2)
Z	4
ρ_{calc}g/cm^3	1.241
μ/mm^{-1}	0.562
F(000)	968.0
Crystal size/mm^3	0.264 × 0.208 × 0.062
Radiation	CuKα (λ = 1.54178)
2Θ range for data collection/°	6.918 to 161.172
Index ranges	$-9 \leq h \leq 8$, $-21 \leq k \leq 21$, $-25 \leq l \leq 25$
Reflections collected	69907
Independent reflections	5379 [R_{int} = 0.0251, R_{sigma} = 0.0095]
Data/restraints/parameters	5379/0/395
Goodness-of-fit on F^2	1.048
Final R indexes [I>=2σ (I)]	R_1 = 0.0311, wR_2 = 0.0831
Final R indexes [all data]	R_1 = 0.0313, wR_2 = 0.0833
Largest diff. peak/hole / e $Å^{-3}$	0.78/-0.15
Flack parameter	0.01(4)

HPLC-Chromatograms of Helicenes

1a, chiral separation, HPLC

Data File : TH-277-QH-Ph-c_MeCN-H2O_75-25_IC-3.lcd
Comment : TH-277-QH-Ph in MeCN-DCM 90-10
MeCN-H2O 75-25, 45 min isocratic
1.0 mL/min, 298 K
IC-3, 4.6x150mm, 3µm

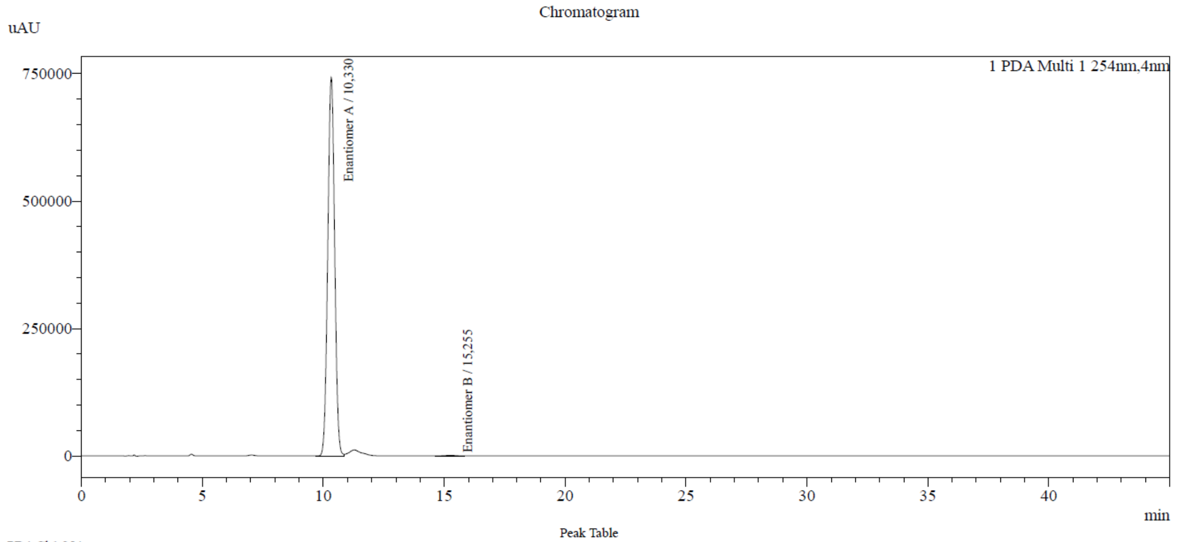

Peak Table

PDA Ch1 254nm

Peak#	Ret. Time	Area	Height	Area%
1	10,330	14695324	742656	99,815
2	15,255	27252	890	0,185
Total		14722576	743545	100,000

1a, achiral separation, HPLC

Comment : TH-277-QH-Ph_chir in MeCN-DCM 90-10
MeCN-H2O 90-10, gradient to 100-0 over 20 min
1.0 mL/min, 295 K
Zorbaxx SB-C18, 4.6x250mm, 3.5µm

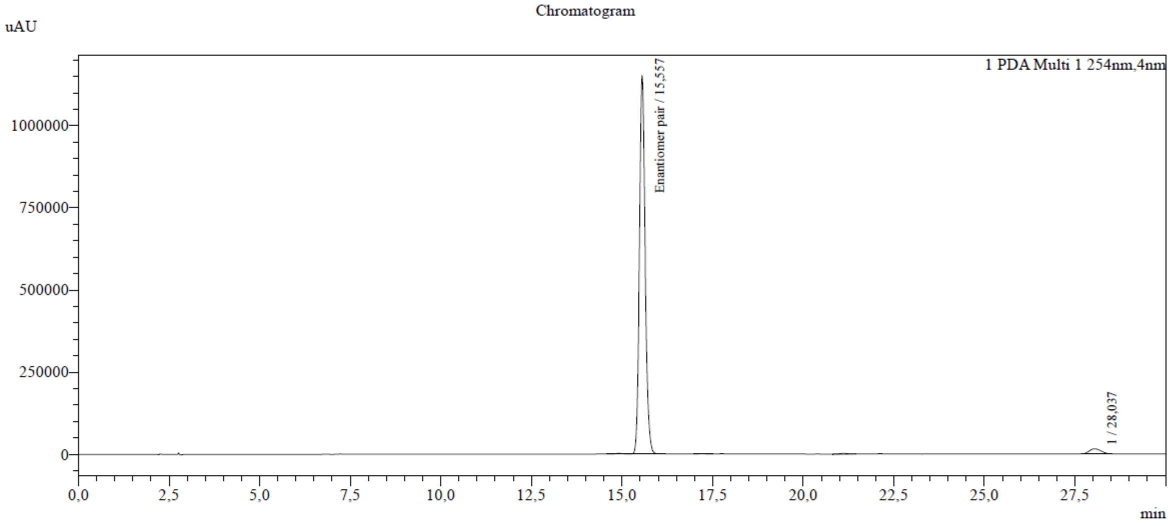

Peak Table

PDA Ch1 254nm

Peak#	Ret. Time	Area	Height	Area%
1	14,906	31357	2436	0,240
2	15,557	12652510	1150434	96,818
3	17,203	11623	989	0,089
4	21,116	27487	2227	0,210
5	28,037	345374	15558	2,643
Total		13068351	1171645	100,000

1b, chiral separation, HPLC

Data File : TH-278-QH-Me-c_MeCN-H2O_75-25_IC-3.lcd
Comment : TH-278-QH-Me in MeCN-DCM 90-10
MeCN-H2O 75-25, 45 min isocratic
1.0 mL/min, 298 K
IC-3, 4.6x150mm, 3µm

Chromatogram

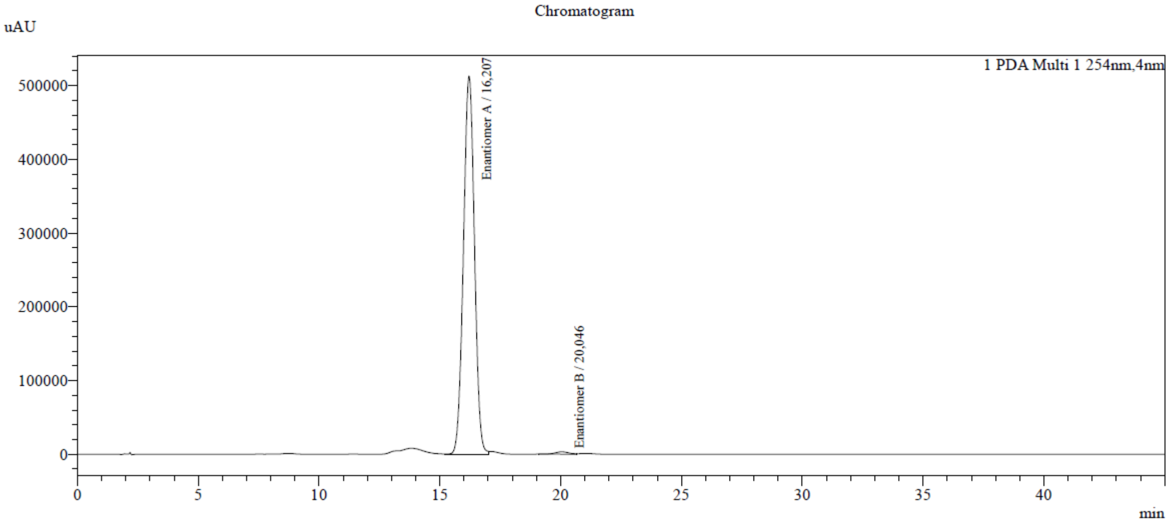

Peak Table

PDA Ch1 254nm

Peak#	Ret. Time	Area	Height	Area%
1	16,207	16195085	512411	99,237
2	20,046	124586	3172	0,763
Total		16319671	515583	100,000

1b, achiral separation, HPLC

Comment : TH-278-QH-Me_chir in MeCN-DCM 90-10
MeCN-H2O 90-10, gradient to 100-0 over 20 min
1.0 mL/min, 295 K
Zorbaxx SB-C18, 4.6x250mm, 3.5µm

Chromatogram

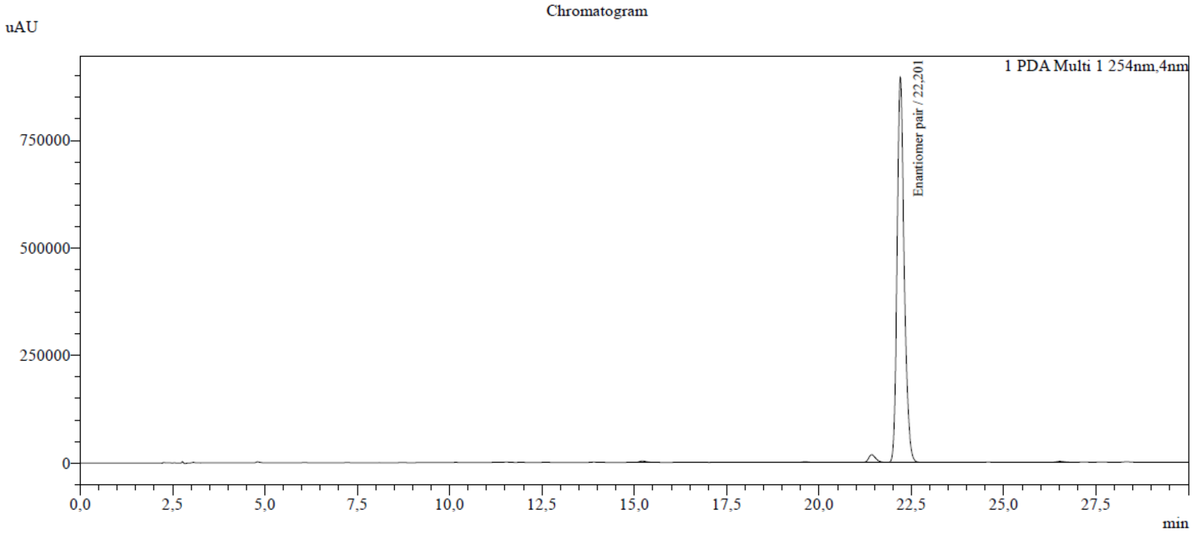

Peak Table

PDA Ch1 254nm

Peak#	Ret. Time	Area	Height	Area%
1	15,214	43296	3350	0,353
2	19,625	17948	1351	0,146
3	21,427	236524	17882	1,930
4	22,201	11933641	895247	97,354
5	26,525	26628	1803	0,217
Total		12258036	919633	100,000

1c, chiral separation, HPLC

Data File : TH-279-QH-OMe-c_MeCN-H2O_75-25_IC-3.lcd
Comment : TH-279-QH-OMe in MeCN-DCM 90-10
MeCN-H2O 75-25, 45 min isocratic
1.0 mL/min, 298 K
IC-3, 4.6x150mm, 3µm

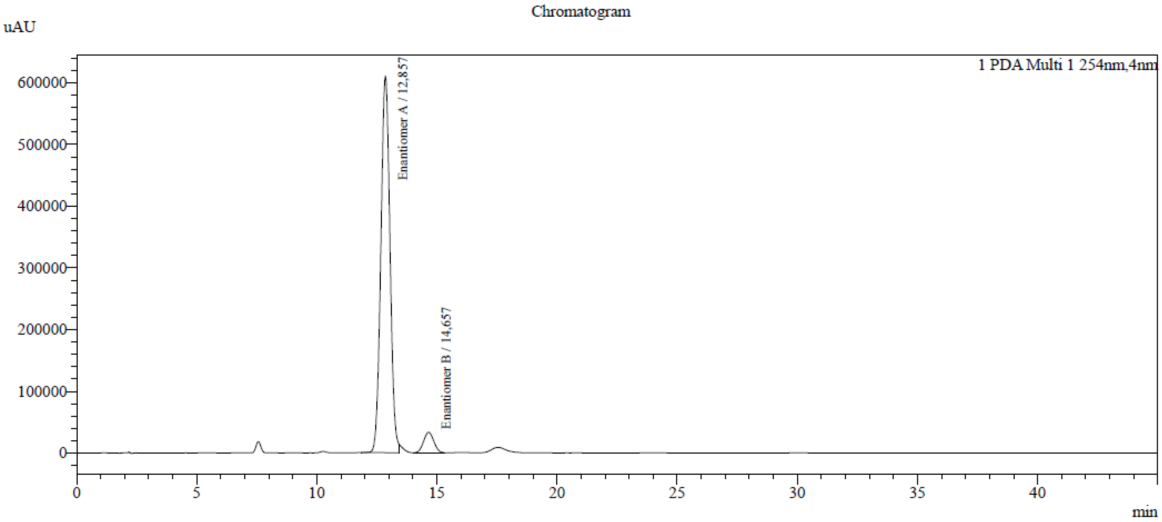

Peak Table

PDA Ch1 254nm

Peak#	Ret. Time	Area	Height	Area%
1	12,857	16074651	609290	94,255
2	14,657	979732	33103	5,745
Total		17054383	642393	100,000

1c, achiral separation, HPLC

Comment : TH-279-QH-OMe_chir in MeCN-DCM 90-10
MeCN-H2O 90-10, gradient to 100-0 over 20 min
1.0 mL/min, 295 K
Zorbaxx SB-C18, 4.6x250mm, 3.5µm

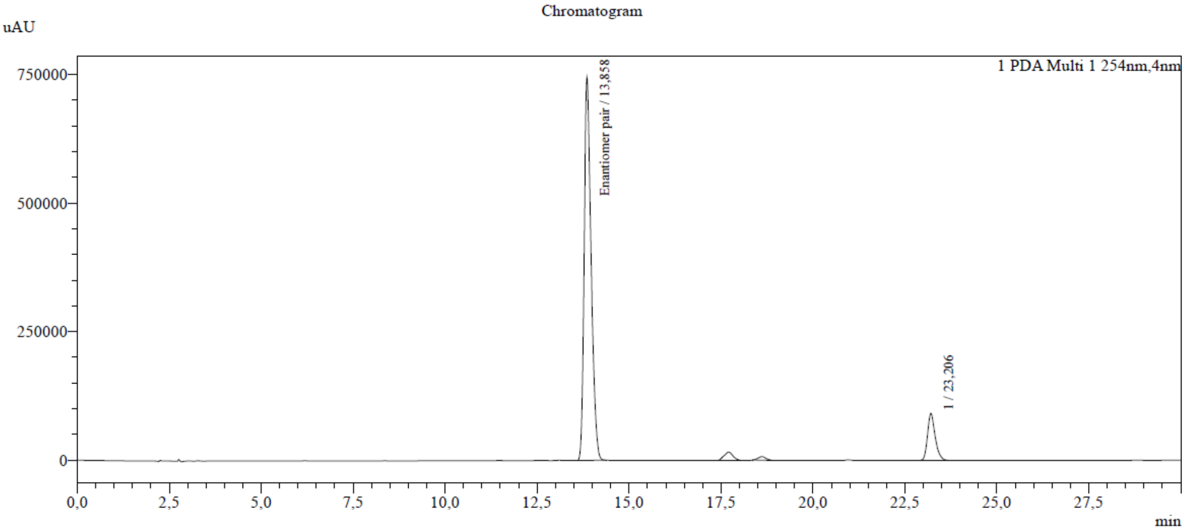

Peak Table

PDA Ch1 254nm

Peak#	Ret. Time	Area	Height	Area%
1	13,858	9763820	745462	85,146
2	17,710	284308	16516	2,479
3	18,611	107325	7091	0,936
4	23,206	1311716	91621	11,439
Total		11467169	860691	100,000

1d, chiral separation, HPLC

Data File : TH-280-QH-CF3_MeCN-H2O_75-25_IC-3.lcd
Comment : TH-280-QH-CF3 in MeCN-DCM 90-10
MeCN-H2O 75-25, 45 min isocratic
1.0 mL/min, 298 K
IC-3, 4.6x150mm, 3µm

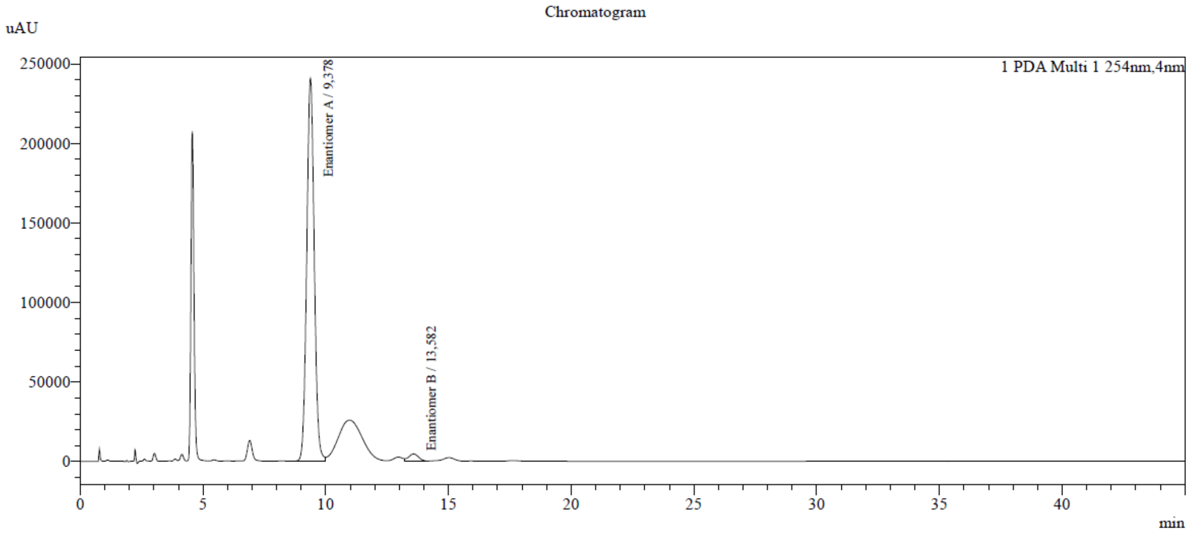

Peak Table

PDA Ch1 254nm

Peak#	Ret. Time	Area	Height	Area%
1	9,378	5114217	240667	97,719
2	13,582	119376	4408	2,281
Total		5233593	245074	100,000

1d, achiral separation, HPLC

Comment : TH-280-QH-CF3_chir in MeCN-DCM 90-10
MeCN-H2O 90-10, gradient to 100-0 over 20 min
1.0 mL/min, 295 K
Zorbaxx SB-C18, 4.6x250mm, 3.5µm

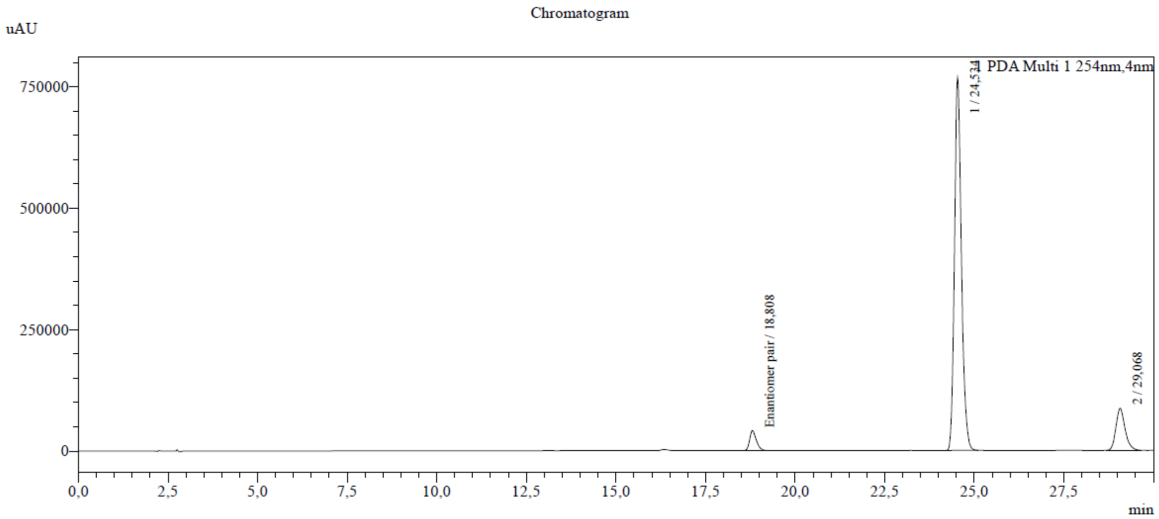

Peak Table

PDA Ch1 254nm

Peak#	Ret. Time	Area	Height	Area%
1	18,808	532092	41220	4,285
2	24,534	10353392	767596	83,378
3	29,068	1531885	86511	12,337
Total		12417368	895326	100,000

1e from **8a**, chiral separation, HPLC

Comment : TH-RMa-605-clean in MeCN-CDCl3 90-10
MeCN-H2O 70-30, 35 min
1.0 mL/min, 17.3 MPa, 303 K
IC-3, 4.6x150mm, 3µm

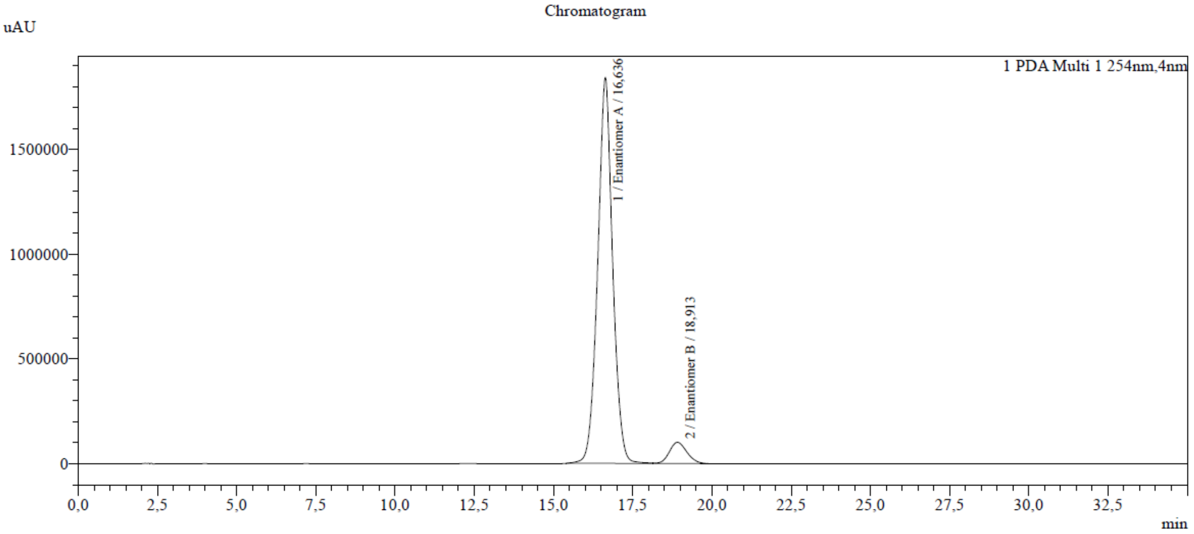

Peak Table

PDA Ch1 254nm

Peak#	Ret. Time	Area	Height	Area%
1	16,636	60542212	1841691	93,935
2	18,913	3908699	101519	6,065
Total		64450911	1943210	100,000

1e from **8a**, achiral separation, HPLC

Comment : TH-RMa-605 in MeCN-DCM 90-10
MeCN-H2O 90-10, gradient to 100-0 over 20 min
1.0 mL/min, 11.9 MPa, 295 K
Zorbax SB-C18, 4.6x250mm, 3.5µm

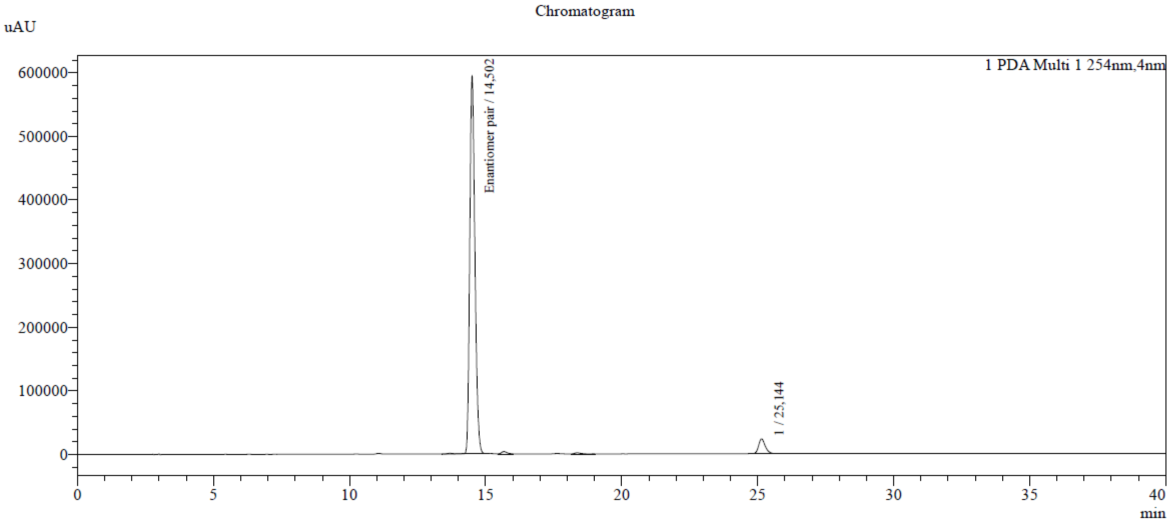

Peak Table

PDA Ch1 254nm

Peak#	Ret. Time	Area	Height	Area%
1	13,685	14124	1111	0,169
2	14,502	7880557	591234	94,278
3	15,673	48717	3597	0,583
4	18,367	24713	1748	0,296
5	25,144	390697	23086	4,674
Total		8358808	620776	100,000

1e from **8b**, chiral separation, HPLC

Comment : TH-295-chir in MeCN-DCM 90-10
MeCN-H2O 70-30, 45 min isocratic
1.0 mL/min, 298 K
IC-3, 4.6x150mm, 3µm

Chromatogram

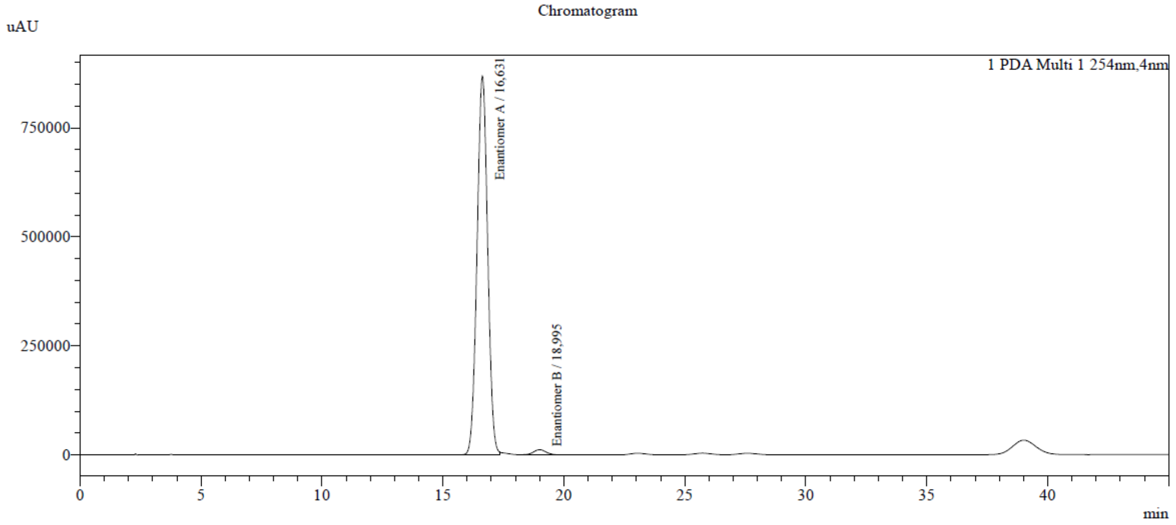

Peak Table

PDA Ch1 254nm

Peak#	Ret. Time	Area	Height	Area%
1	16,631	26468644	868756	98,488
2	18,995	406310	11619	1,512
Total		26874953	880375	100,000

1e from **8b**, achiral separation, HPLC

Comment : TH-295 in MeCN-DCM 90-10
MeCN-H2O 80-20 5 min, gradient to 100-0 over 10 min
1.0 mL/min, 15.5 MPa, 295 K
Zorbax SB-C18, 4.6x250mm, 3.5µm

Chromatogram

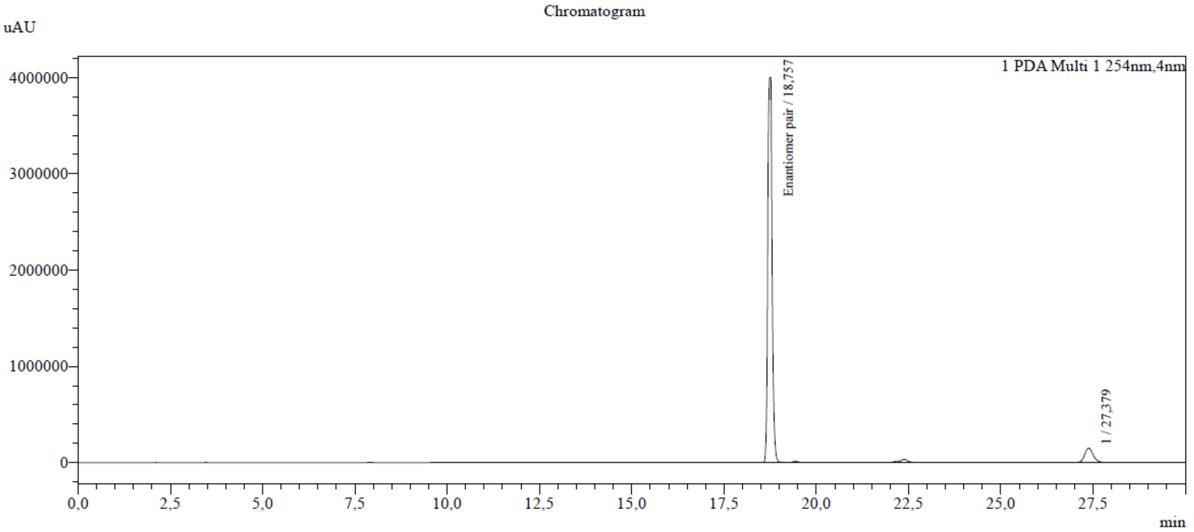

Peak Table

PDA Ch1 254nm

Peak#	Ret. Time	Area	Height	Area%
1	18,757	33018645	3996775	91,918
2	19,426	104303	13084	0,290
3	22,185	108606	13381	0,302
4	22,382	370094	32034	1,030
5	27,379	2320075	147978	6,459
Total		35921724	4203253	100,000

20a, chiral separation, HPLC

Comment : TH-304c in MeCN-DCM 90-10
MeCN-H2O 65-35, 40 min isocratic
1.0 mL/min, 19.8 MPa, 298 K
IC-3, 4.6x150mm, 3µm

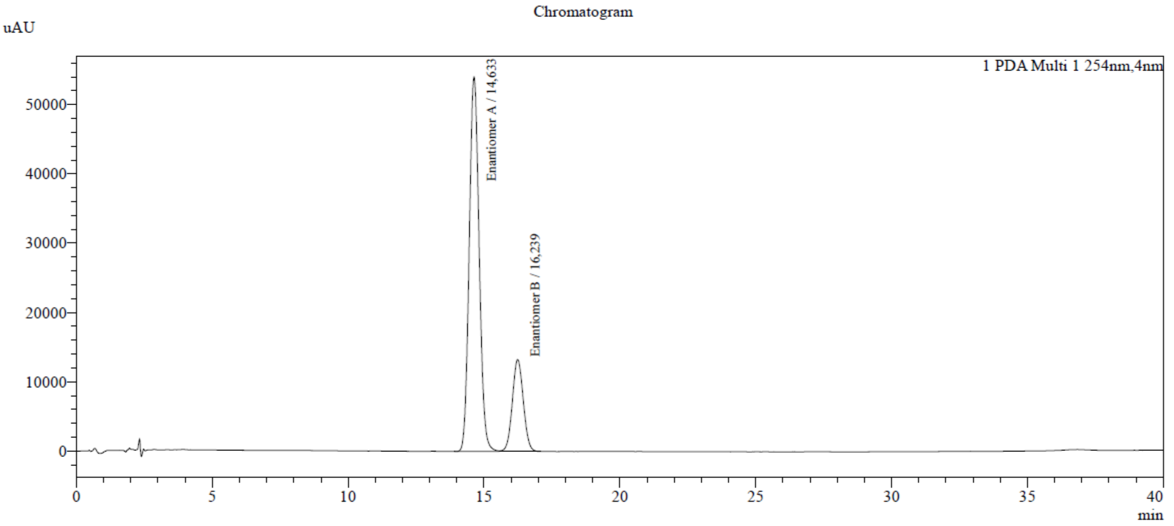

Peak Table

PDA Ch1 254nm

Peak#	Ret. Time	Area	Height	Area%
1	14,633	1371489	54033	78,665
2	16,239	371965	13265	21,335
Total		1743454	67297	100,000

20a, achiral separation, HPLC

Comment : TH-304-moreKat in MeCN-DCM 90-10
MeCN-H2O 90-10, 30 min isocratic
1.0 mL/min, 12.8 MPa, 295 K
Zorbax SB-C18, 4.6x250mm, 3.5µm

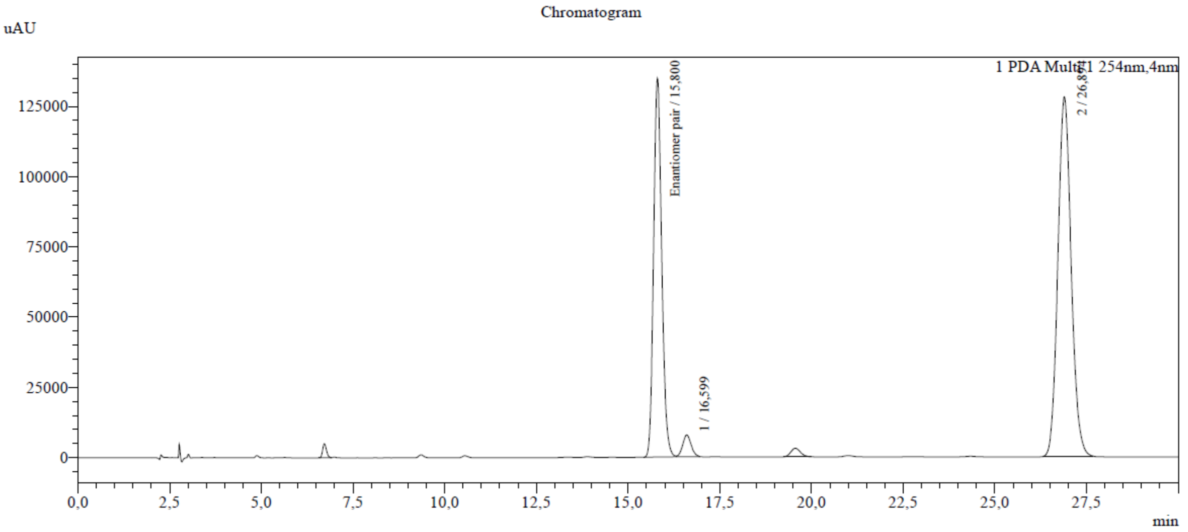

Peak Table

PDA Ch1 254nm

Peak#	Ret. Time	Area	Height	Area%
1	6,720	35477	5034	0,645
2	15,800	2069091	134818	37,630
3	16,599	123529	7742	2,247
4	19,559	55367	3050	1,007
5	26,894	3214993	127895	58,471
Total		5498456	278539	100,000

20a, chiral separation, SFC

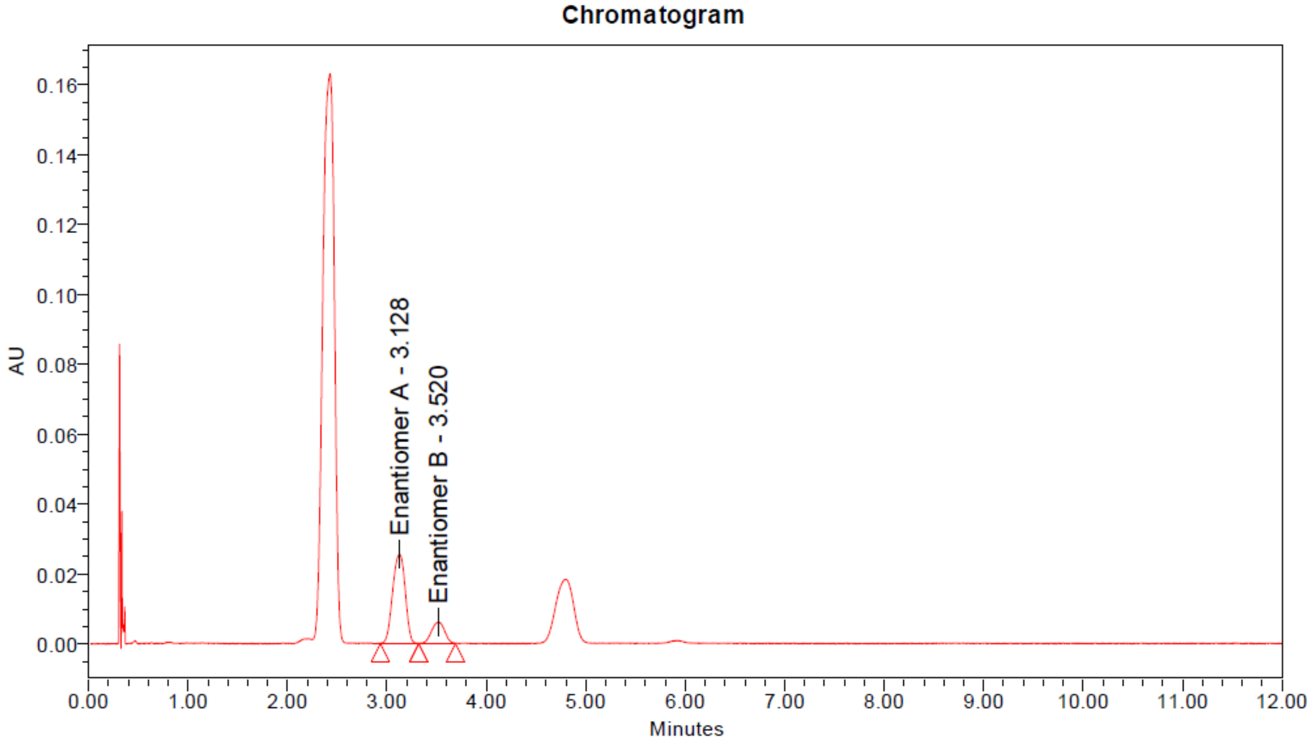

Component Results

	Name	RT	Area	Height	% Area
1	Enantiomer A	3.128	225239	25625	79.85
2	Enantiomer B	3.520	56829	6150	20.15

20b, chiral separation, HPLC

Comment : TH-303c in MeCN-DCM 90-10
MeCN-H2O 65-35, 40 min isocratic
1.0 mL/min, 298 K
IC-3, 4.6x150mm, 3μm

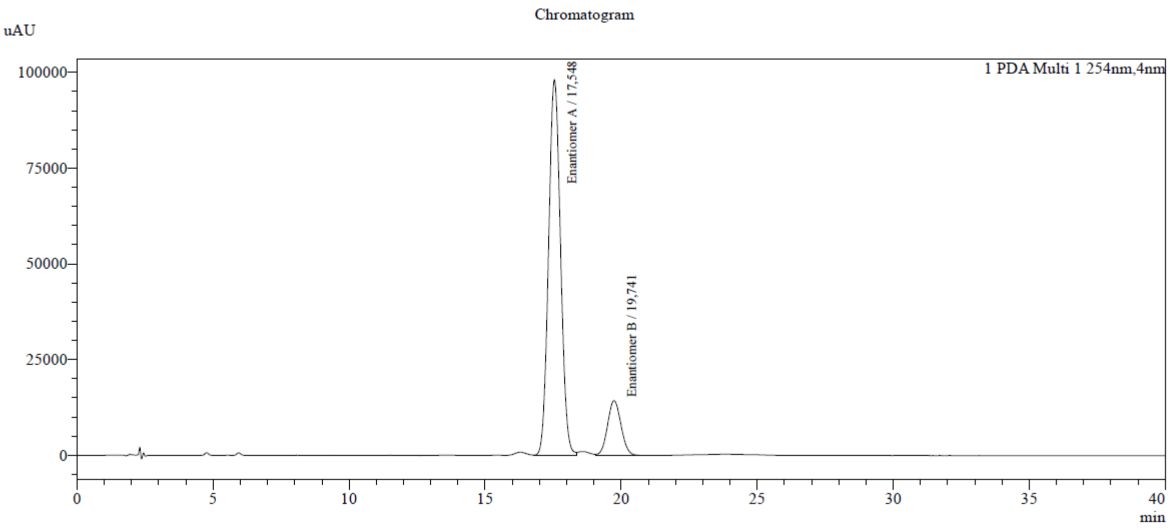

Peak Table

PDA Ch1 254nm

Peak#	Ret. Time	Area	Height	Area%
1	17,548	2997871	98054	85,922
2	19,741	491197	14287	14,078
Total		3489068	112341	100,000

20b, achiral separation, HPLC

Comment : TH-303-MeMe-chiral in MeCN-DCM 90-10
MeCN-H2O 90-10, 45 min isocratic
1.0 mL/min, 295 K
Zorbax SB-C18, 4.6x250mm, 3.5µm

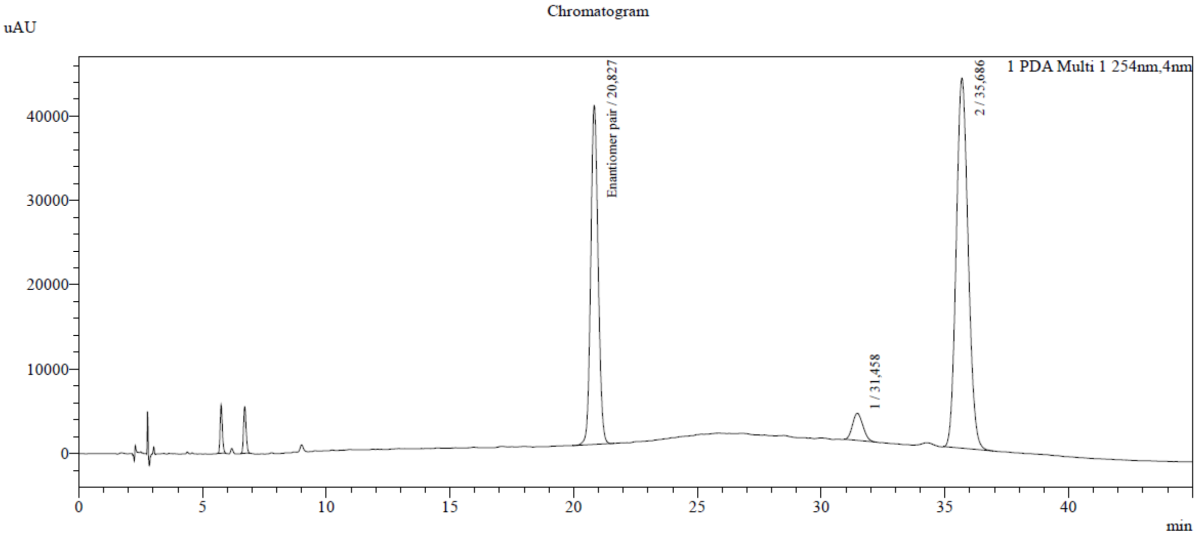

Peak Table

PDA Ch1 254nm

Peak#	Ret. Time	Area	Height	Area%
1	5,749	36002	5759	1,456
2	6,704	39707	5517	1,605
3	20,827	834375	40173	33,732
4	31,458	93824	3211	3,793
5	35,686	1469598	43877	59,414
Total		2473506	98537	100,000

20b, chiral separation, SFC

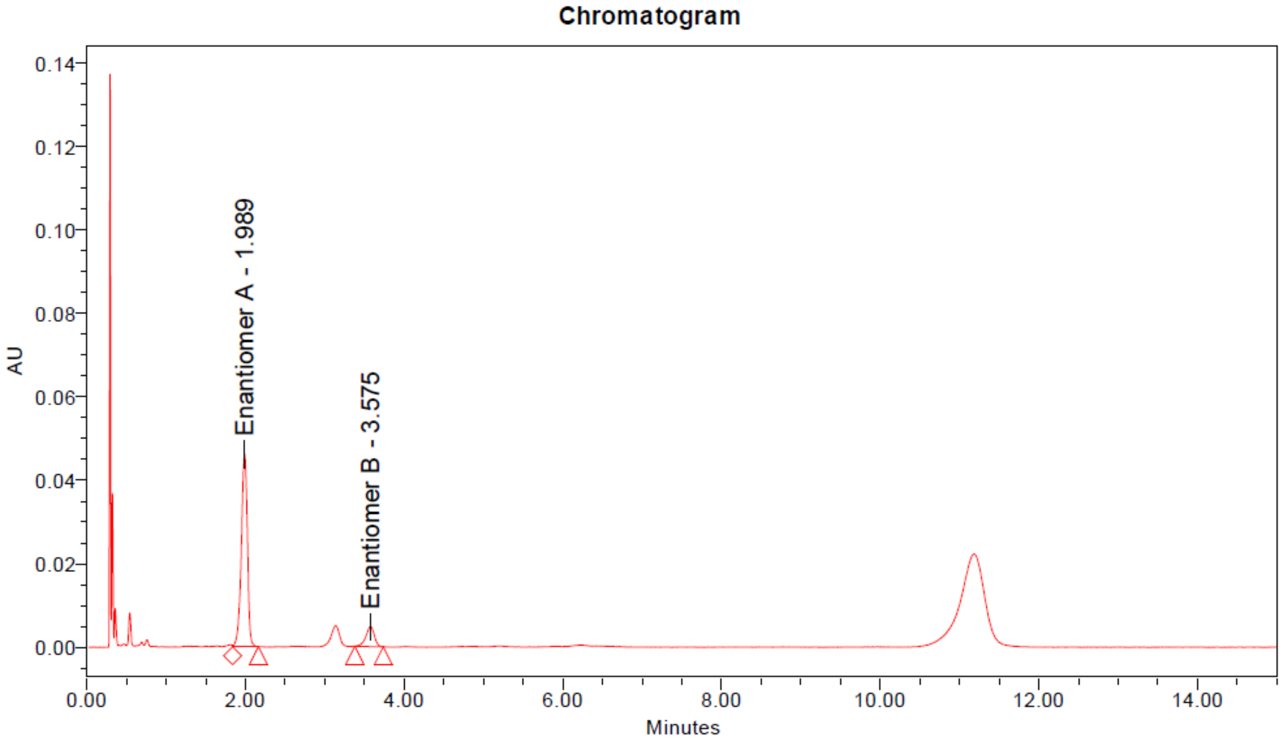

Component Results

	Name	RT	Area	Height	% Area
1	Enantiomer A	1.989	245724	46198	87.35
2	Enantiomer B	3.575	35585	4764	12.65

20c, chiral separation, HPLC

Comment : TH-302c in MeCN-DCM 90-10
MeCN-H2O 65-35, 40 min isocratic
1.0 mL/min, 298 K
IC-3, 4.6x150mm, 3µm

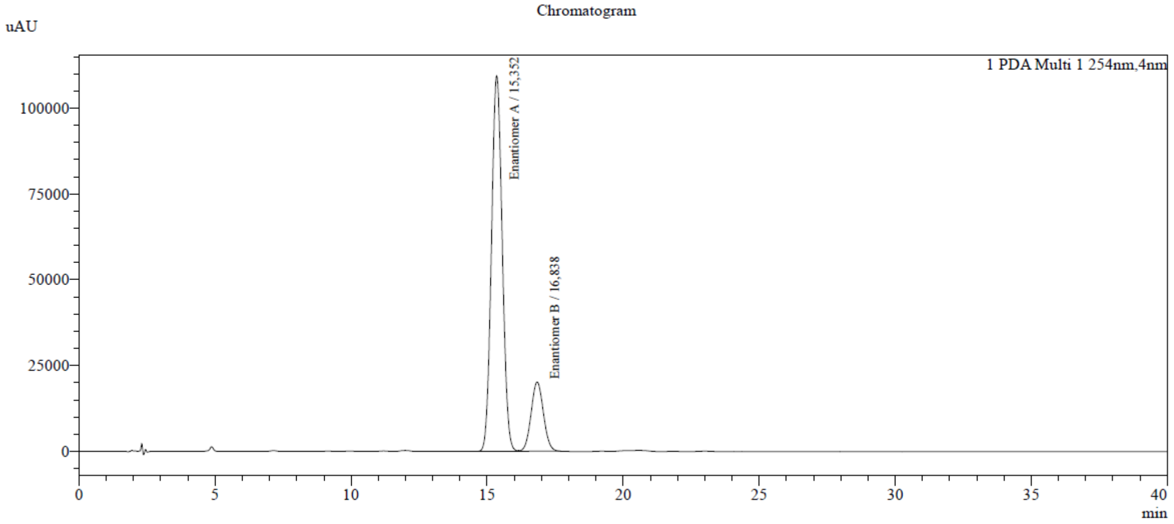

Peak Table

PDA Ch1 254nm

Peak#	Ret. Time	Area	Height	Area%
1	15,352	3013687	109378	82,591
2	16,838	635251	20177	17,409
Total		3648938	129555	100,000

20c, achiral separation, HPLC

Comment : TH-302-MeOMe-chiral in MeCN-DCM 90-10
MeCN-H2O 90-10, 45 min isocratic
1.0 mL/min, 295 K
Zorbax SB-C18, 4.6x250mm, 3.5µm

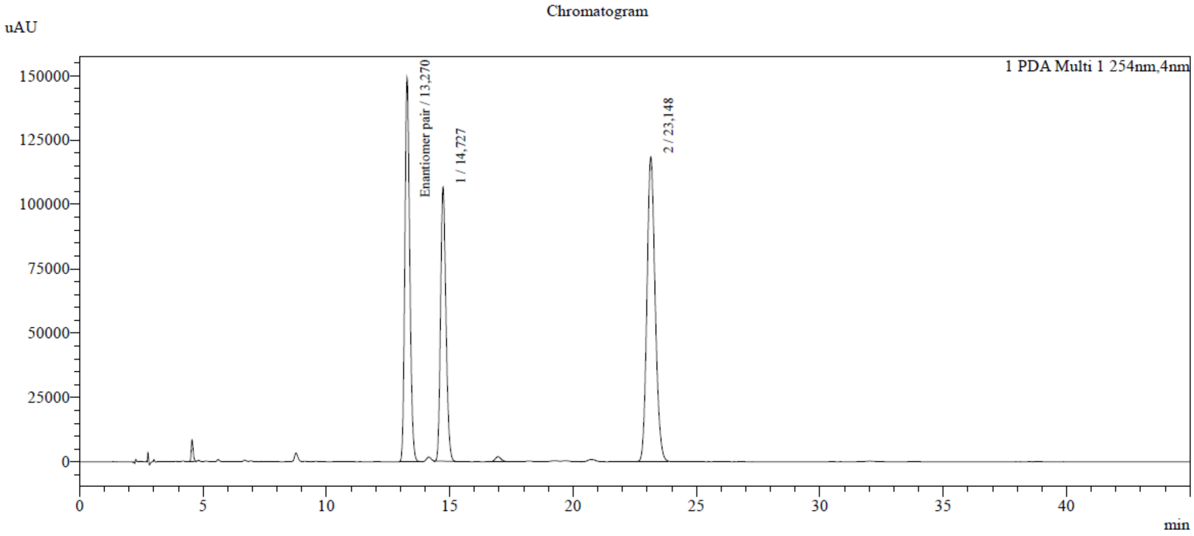

Peak Table

PDA Ch1 254nm

Peak#	Ret. Time	Area	Height	Area%
1	4,555	46161	8409	0,713
2	13,270	2051115	149083	31,701
3	14,727	1610442	106515	24,890
4	16,953	34082	1969	0,527
5	23,148	2728484	118149	42,169
Total		6470284	384124	100,000

20c, chiral separation, SFC

Chromatogram

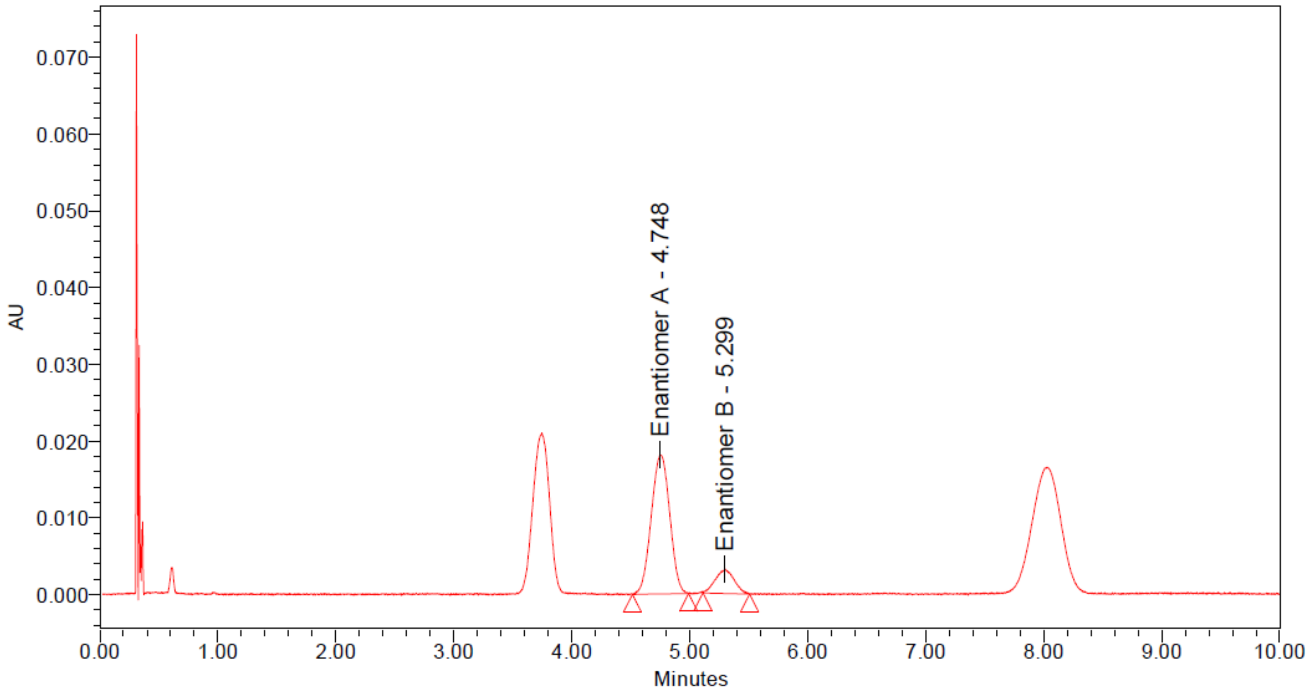

Component Results

	Name	RT	Area	Height	% Area
1	Enantiomer A	4.748	197630	18155	85.06
2	Enantiomer B	5.299	34711	3211	14.94

20d, chiral separation, HPLC

Comment : TH-285-1 in MeCN-DCM 90-10
MeCN-H2O 65-35, 40 min isocratic
1.0 mL/min, 298 K
IC-3, 4.6x150mm, 3μm

Chromatogram

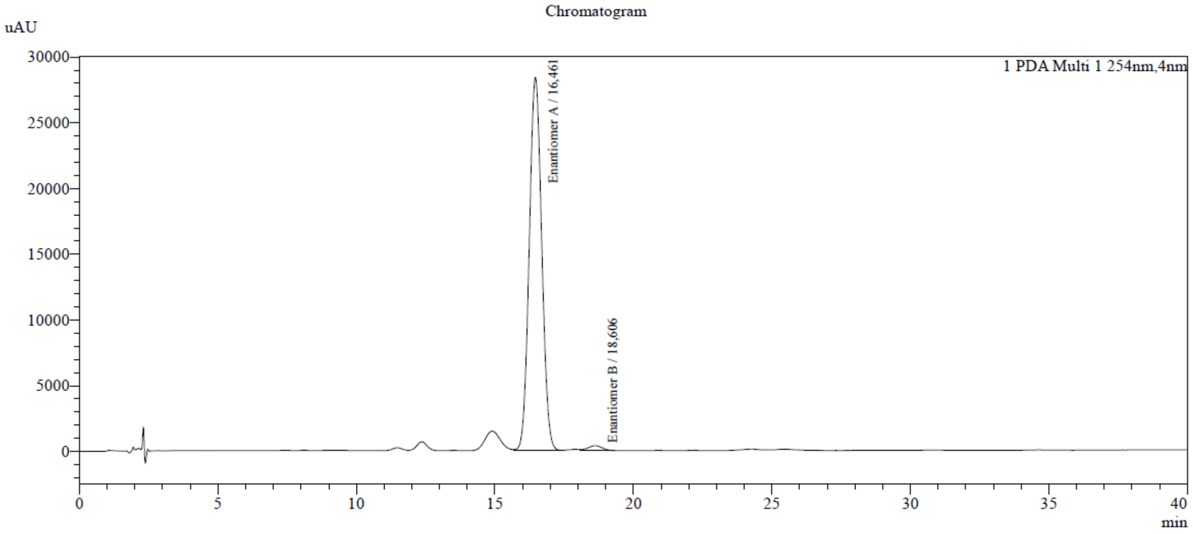

Peak Table

PDA Ch1 254nm

Peak#	Ret. Time	Area	Height	Area%
1	16,461	890320	28376	98,576
2	18,606	12865	361	1,424
Total		903185	28737	100,000

20d, achiral separation, HPLC

Comment : TH-285-PhPhPh-c in MeCN-DCM 90-10
MeCN-H2O 90-10, gradient to 100-0 over 10 min
1.0 mL/min, 295 K
Zorbax SB-C18, 4.6x250mm, 3.5µm

Chromatogram

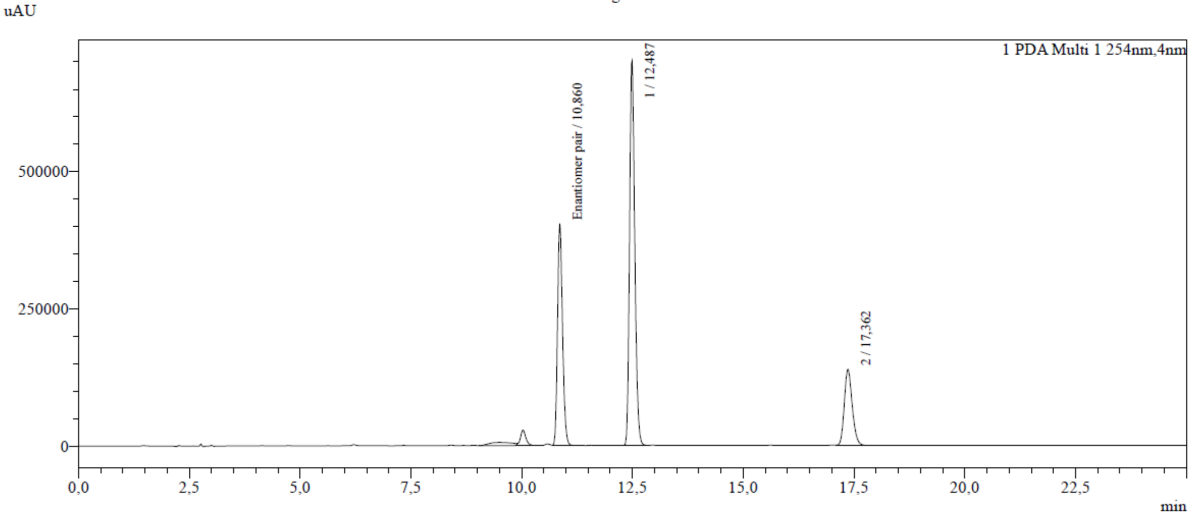

Peak Table

PDA Ch1 254nm

Peak#	Ret. Time	Area	Height	Area%
1	9,499	213049	5965	1,921
2	10,029	242027	28265	2,182
3	10,860	3171233	404623	28,595
4	12,487	5746960	700439	51,820
5	17,362	1716885	138726	15,481
Total		11090154	1278017	100,000

20e, chiral separation, HPLC

Comment : TH-305c in MeCN-DCM 90-10
MeCN-H2O 65-35, 40 min isocratic
1.0 mL/min, 298 K
IC-3, 4.6x150mm, 3µm

Chromatogram

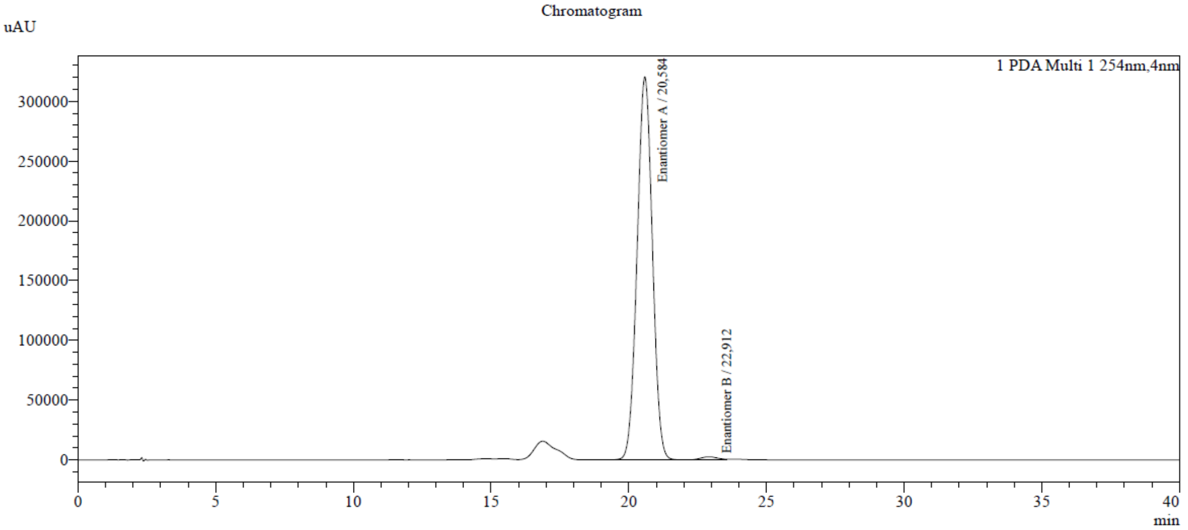

Peak Table

PDA Ch1 254nm

Peak#	Ret. Time	Area	Height	Area%
1	20,584	12366899	320238	99,123
2	22,912	109359	2564	0,877
Total		12476258	322802	100,000

20e, achiral separation, HPLC

Comment : TH-305-PhMe-chiral in MeCN-DCM 90-10
MeCN-H2O 90-10, gradient to 100-0 over 10 min
1.0 mL/min, 295 K
Zorbax SB-C18, 4.6x250mm, 3.5µm

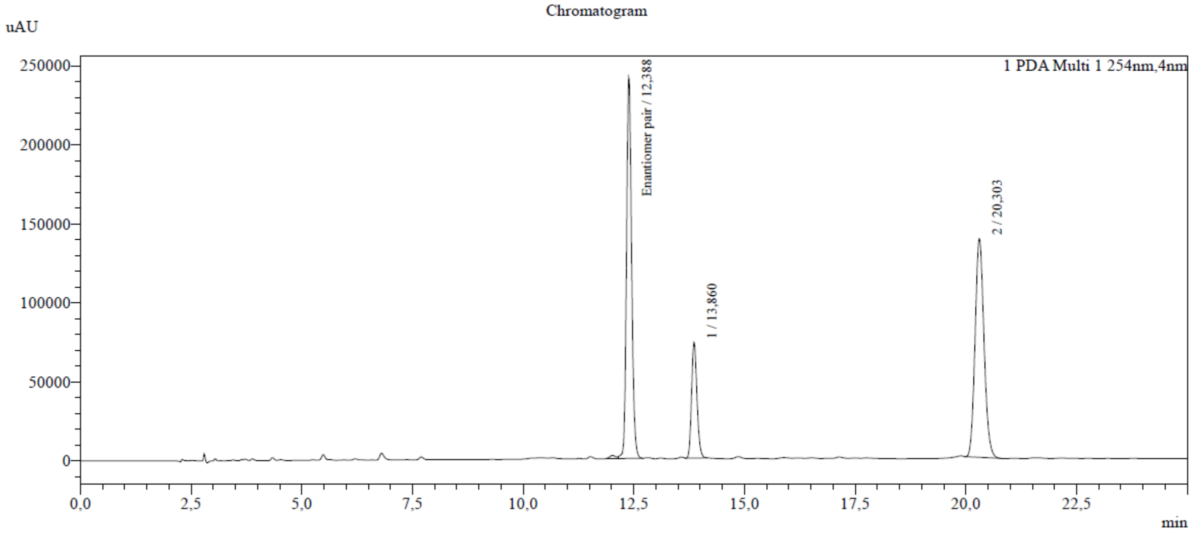

Peak Table

PDA Ch1 254nm

Peak#	Ret. Time	Area	Height	Area%
1	11,989	19718	1795	0,438
2	12,388	1921387	241161	42,728
3	13,860	617305	73141	13,728
4	20,303	1938362	138397	43,106
Total		4496771	454494	100,000

20e, chiral separation, SFC

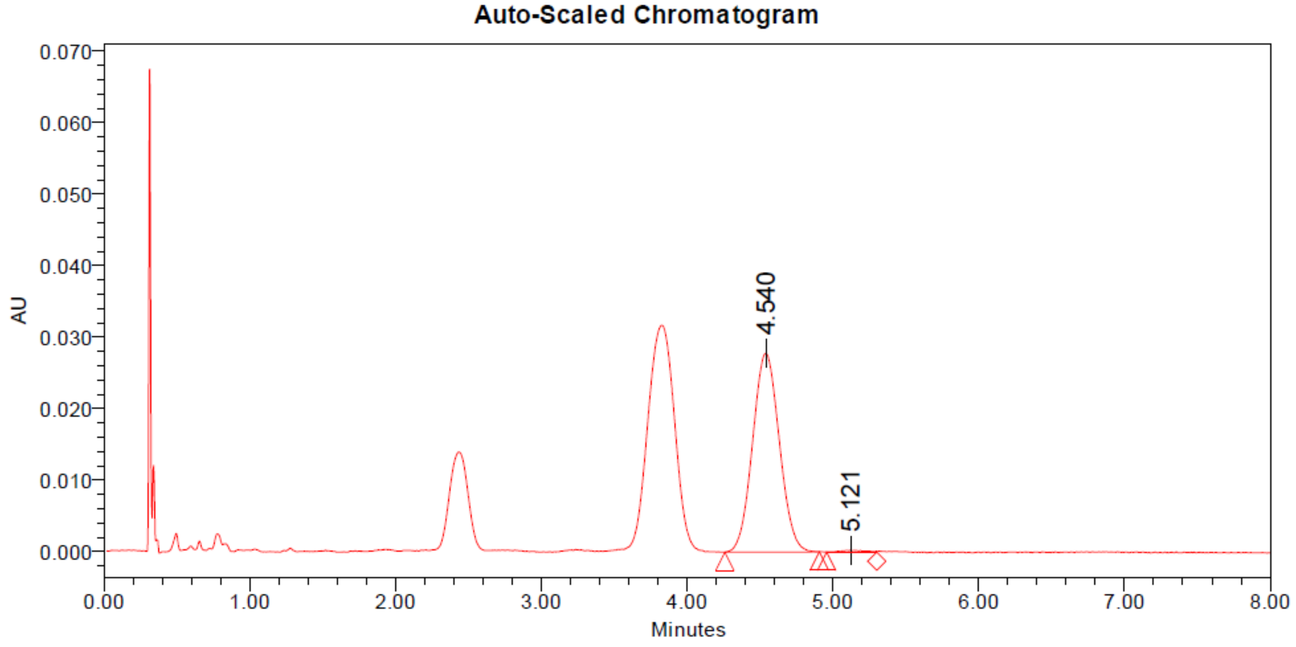

Peak Results

	Name	RT	Area	Height	Amount	Units
1		4.540	355096	27801		
2		5.121	2600	246		

20f, chiral separation, HPLC

Data File : TH-Kat-PhOMe_MeCN-H2O_60-40_IC-3.lcd
Comment : TH-Kat-PhOMe in MeCN-DCM 90-10
MeCN-H2O 60-40, 40 min isocratic
1.0 mL/min, 298 K
IC-3, 4.6x150mm, 3µm

Chromatogram

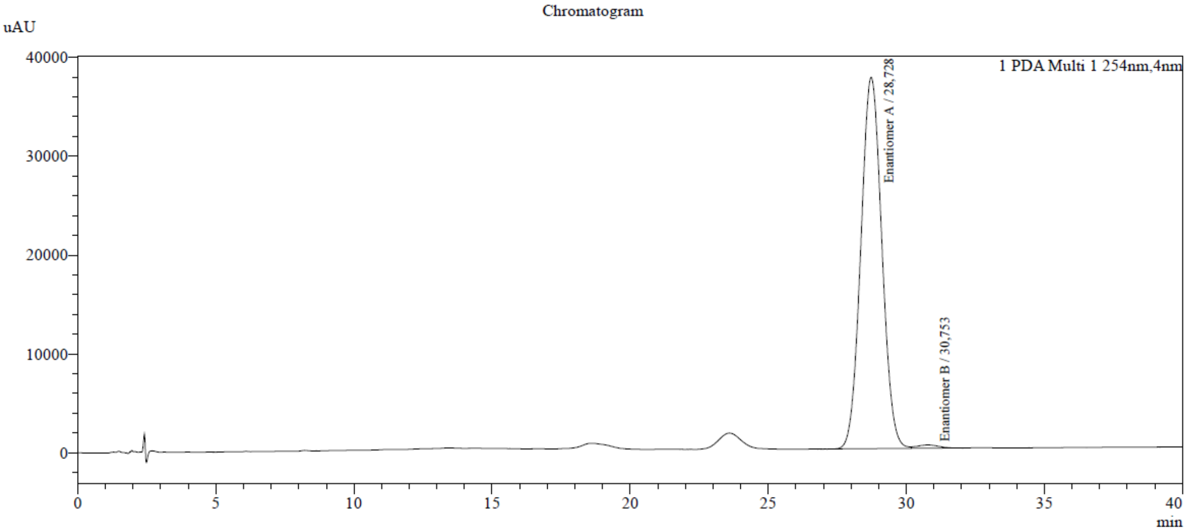

Peak Table

PDA Ch1 254nm

Peak#	Ret. Time	Area	Height	Area%
1	28,728	1981350	37570	99,081
2	30,753	18386	337	0,919
Total		1999736	37907	100,000

20f, achiral seperation, HPLC

Comment : TH-Kat-Phen-PhOMe in MeCN-DCM 90-10
MeCN-H2O 90-10, gradient to 100-0 over 20 min
1.0 mL/min, 295 K
Zorbax SB-C18, 4.6x250mm, 3.5µm

Chromatogram

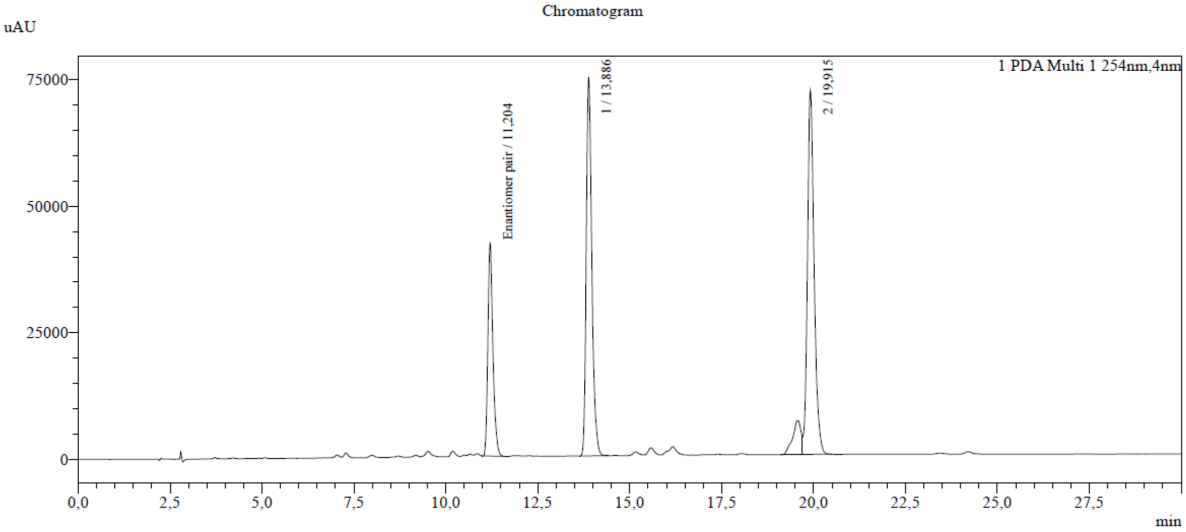

Peak Table

PDA Ch1 254nm

Peak#	Ret. Time	Area	Height	Area%
1	11,204	402818	42055	18,291
2	13,886	785874	74836	35,684
3	19,572	108202	6755	4,913
4	19,915	905436	71973	41,113
Total		2202329	195618	100,000

20g, chiral separation, HPLC

Comment : TH-RMa-2.5-clean in MeCN-DCM 90-10
MeCN-H2O 70-30, 35 min isocratic
1.0 mL/min, 17.3 MPa, 303 K
IC-3, 4.6x150mm, 3µm

Chromatogram

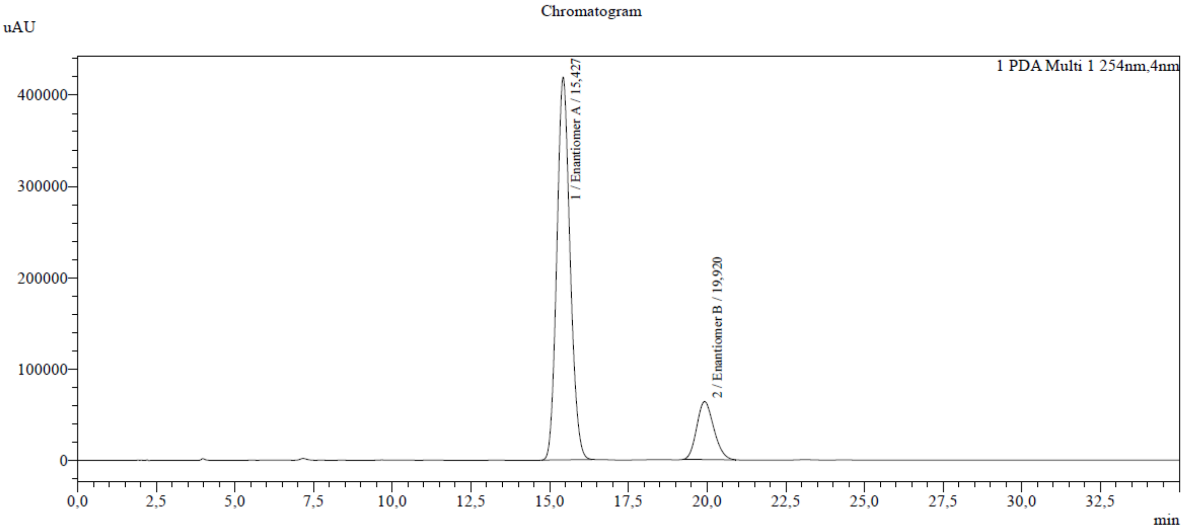

Peak Table

PDA Ch1 254nm

Peak#	Ret. Time	Area	Height	Area%
1	15,427	12255049	419177	83,672
2	19,920	2391546	63528	16,328
Total		14646595	482705	100,000

20g, achiral separation, HPLC

Comment : TH-RMa-2.5 in MeCN-DCM 90-10
MeCN-H2O 90-10, 60 min isocratic
1.0 mL/min, 295 K
Zorbax SB-C18, 4.6x250mm, 3.5µm

Chromatogram

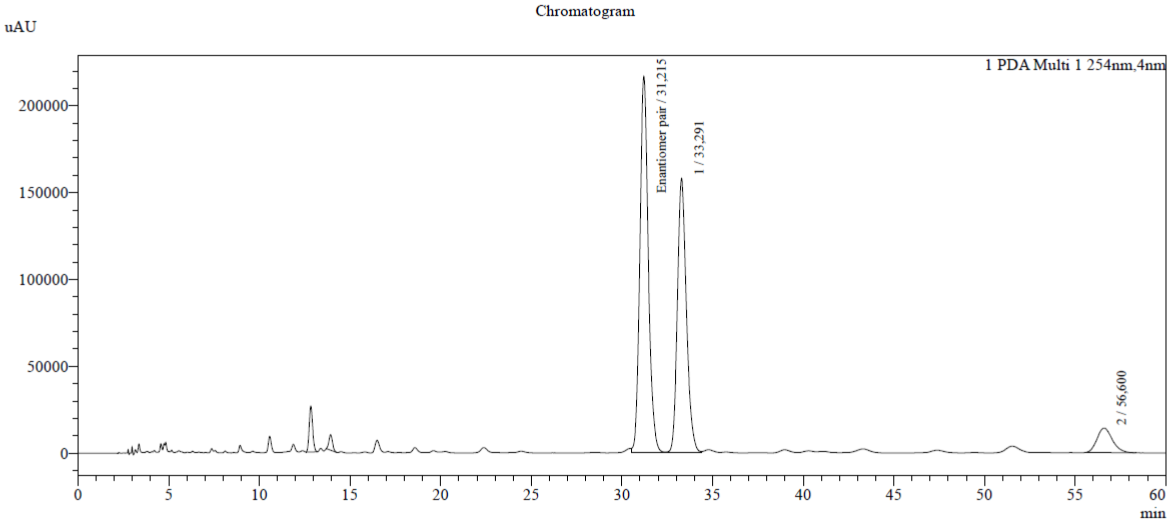

Peak Table

PDA Ch1 254nm

Peak#	Ret. Time	Area	Height	Area%
1	12,840	348274	26451	2,529
2	13,931	120584	8871	0,876
3	31,215	7008341	216788	50,887
4	33,291	5462572	158009	39,663
5	56,600	832594	14143	6,045
Total		13772364	424263	100,000

20h, chiral separation, HPLC

Comment : TH-RMa-2.3-clean in MeCN-DCM 90-10
MeCN-H2O 68-32, 45 min isocratic
0.8 mL/min, 13.8 MPa, 303 K
IC-3, 4.6x150mm, 3µm

Chromatogram

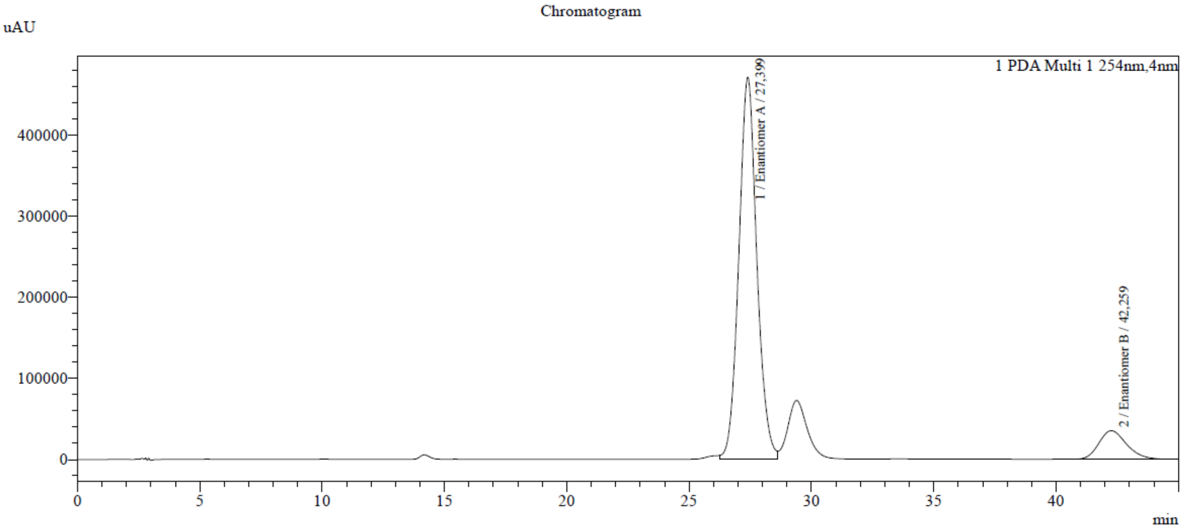

Peak Table

PDA Ch1 254nm

Peak#	Ret. Time	Area	Height	Area%
1	27,399	24144352	471094	89,696
2	42,259	2773511	35193	10,304
Total		26917863	506287	100,000

20h, achiral separation, HPLC

Comment : TH-RMa-2.3 in MeCN-DCM 90-10
MeCN-H2O 90-10, gradient to 100-0 over 20 min
1.0 mL/min, 11.9 MPa, 295 K
Zorbax SB-C18, 4.6x250mm, 3.5µm

Chromatogram

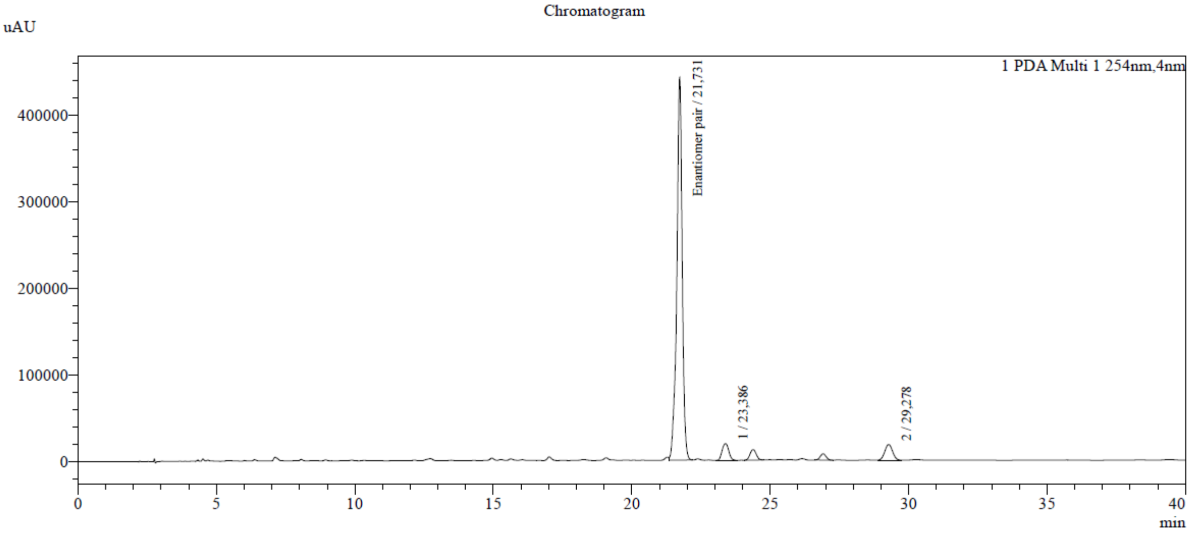

Peak Table

PDA Ch1 254nm

Peak#	Ret. Time	Area	Height	Area%
1	21,731	5992957	441021	86,063
2	23,386	321865	19282	4,622
3	24,380	189504	12096	2,721
4	26,912	107535	7243	1,544
5	29,278	351622	18090	5,050
Total		6963484	497732	100,000

20i, chiral separation, HPLC

Comment : TH-RMa-11-clean in MeCN-CDCl3 90-10
MeCN-H2O 70-30, 35 min
1.0 mL/min, 17.3 MPa, 303 K
IC-3, 4.6x150mm, 3µm

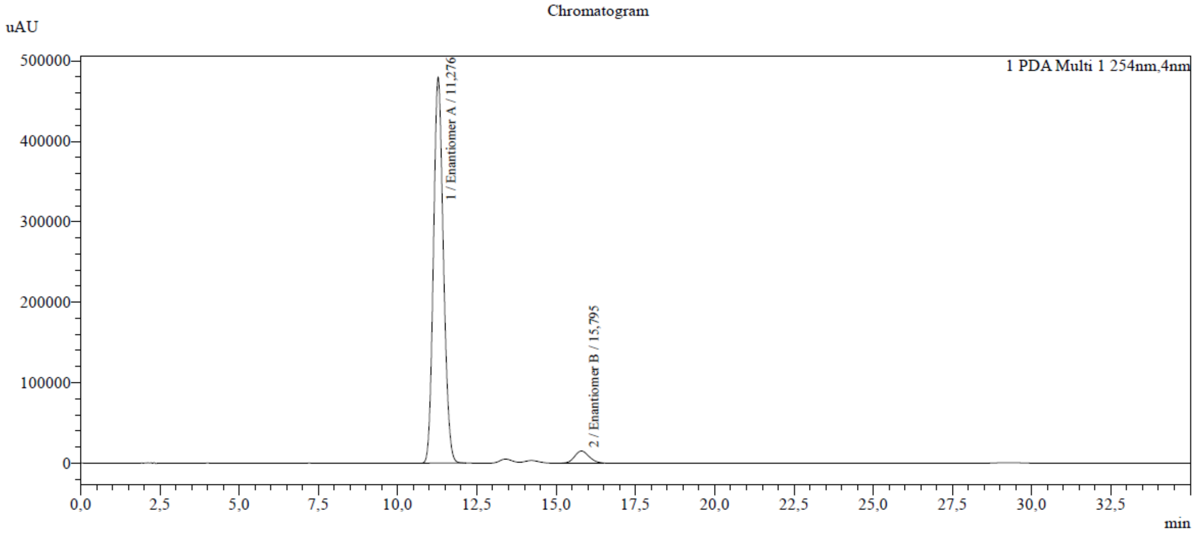

Peak Table

PDA Ch1 254nm

Peak#	Ret. Time	Area	Height	Area%
1	11,276	10475835	479659	95,759
2	15,795	463976	14915	4,241
Total		10939811	494574	100,000

20i, achiral separation, HPLC

Comment : TH-RMa-11 in MeCN-DCM 90-10
MeCN-H2O 90-10, gradient to 100-0 over 20 min
1.0 mL/min, 11.9 MPa, 295 K
Zorbax SB-C18, 4.6x250mm, 3.5µm

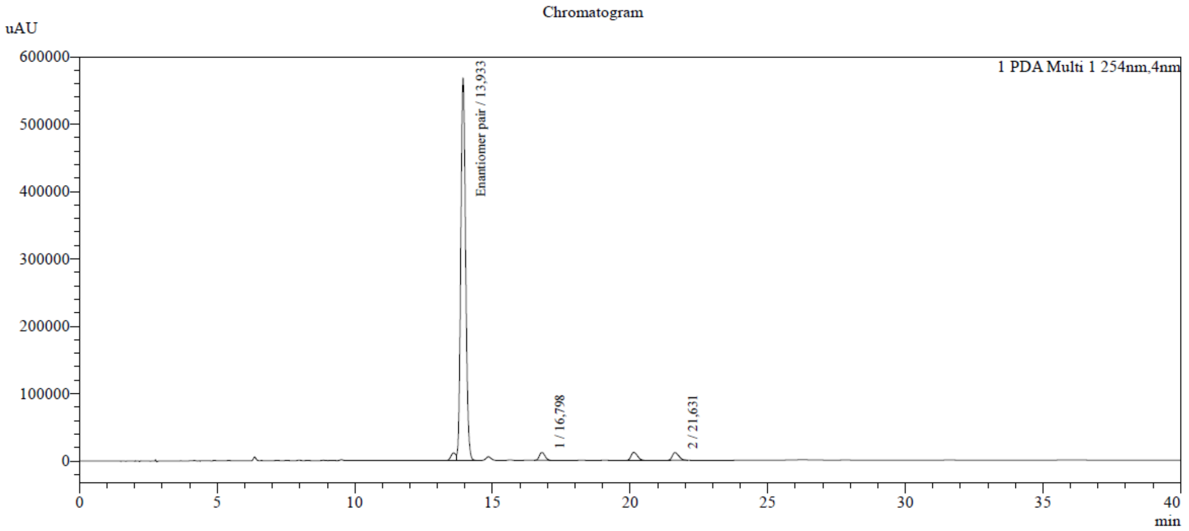

Peak Table

PDA Ch1 254nm

Peak#	Ret. Time	Area	Height	Area%
1	13,590	134410	11214	1,758
2	13,933	6975321	566107	91,240
3	16,798	162617	11556	2,127
4	20,133	186698	11802	2,442
5	21,631	185947	11475	2,432
Total		7644993	612154	100,000

20j, chiral separation, HPLC

Comment : TH-RMa-13-clean in MeCN-CDCl3 90-10
MeCN-H2O 70-30, 35 min
1.0 mL/min, 17.3 MPa, 303 K
IC-3, 4.6x150mm, 3µm

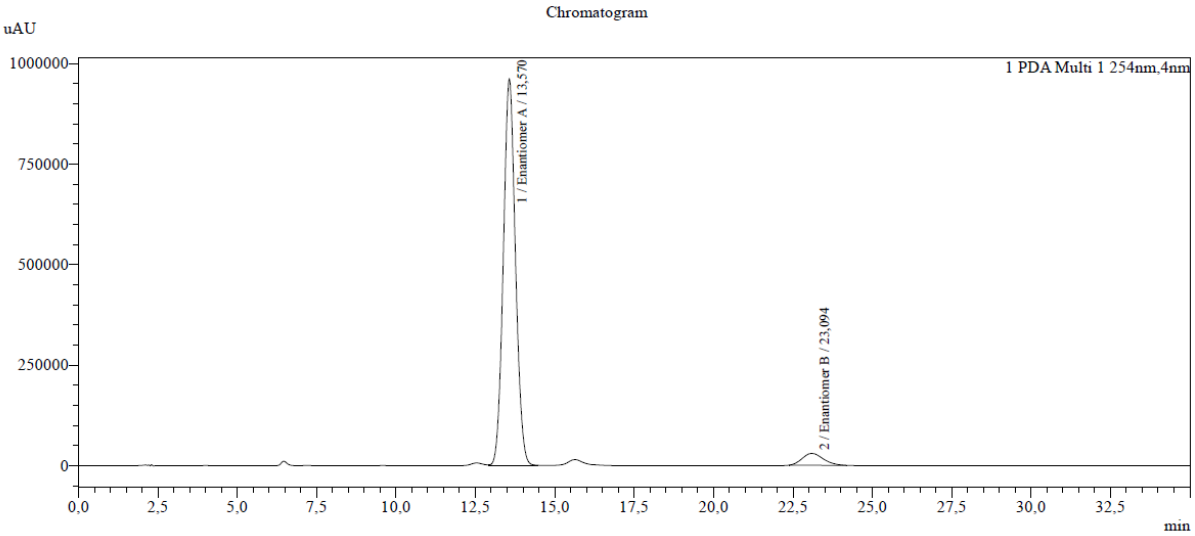

Peak Table

PDA Ch1 254nm

Peak#	Ret. Time	Area	Height	Area%
1	13,570	25496380	960972	94,985
2	23,094	1346015	29404	5,015
Total		26842394	990376	100,000

20j, achiral separation, HPLC

Comment : TH-RMa-13 in MeCN-DCM 90-10
MeCN-H2O 90-10, gradient to 100-0 over 20 min
1.0 mL/min, 11.9 MPa, 295 K
Zorbax SB-C18, 4.6x250mm, 3.5µm

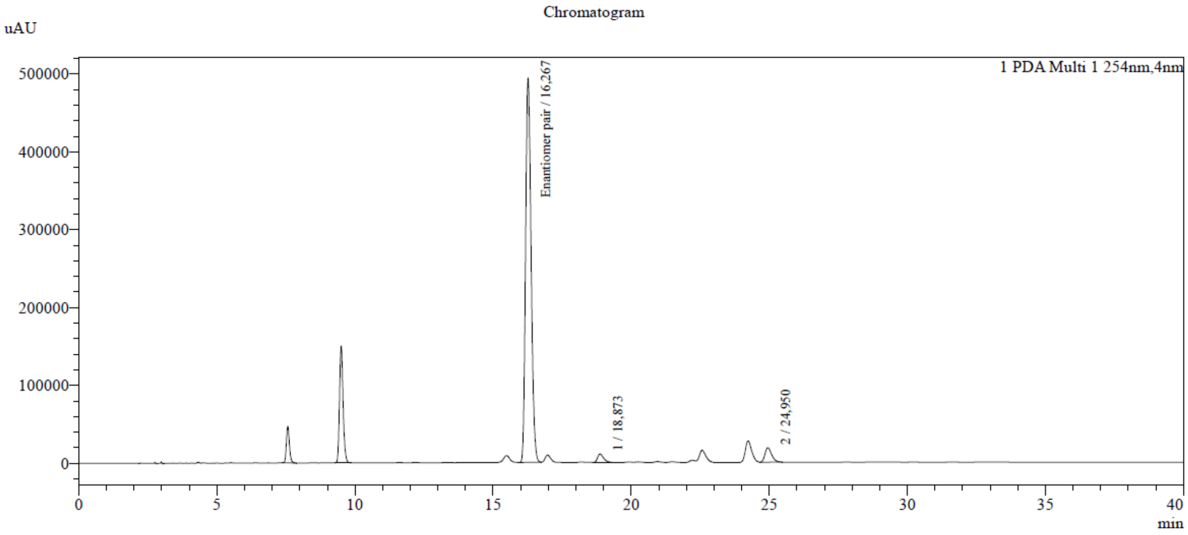

Peak Table

PDA Ch1 254nm

Peak#	Ret. Time	Area	Height	Area%
1	7,567	355006	46627	3,946
2	9,501	1350423	149311	15,010
3	16,267	6811760	491750	75,714
4	18,873	163701	10778	1,820
5	24,950	315760	18686	3,510
Total		8996651	717153	100,000

References

[1] A.S. Hacker, M. Pavano, J. E. Wood, II, C.E. Immoos, H. Hashimoto, S.P. Genis, D.K. Frantz, *J. Org. Chem.* **2018**, 83, 510−515.
[2] D. Pijper, M. G. M. Jongejan, A. Meetsma, B. L. Feringa, *J. Am. Chem. Soc.* **2008**, *130*, 4541.
[3] A.C. Shaikh, D.S. Ranade, P.R. Rajamohanan, P.P. Kulkarni, N.T. Patil, *Angew. Chem. Int. Ed.* **2017**, 56, 757.
[4] L.D.M. Nicholls, M. Marx, T. Hartung, E. González-Fernández, C. Golz, M. Alcarazo, *ACS Catal.* **2018**, *8*, 6079-6085.
[5] H. Tinnermann, L.D.M. Nicholls, T. Johannsen, C.Wille, C. Golz, R. Goddard, M. Alcrarazo *ACS Catal.* **2018**, *8*, 10457-10463.
[6] S. Rousseaux, J. García-Fortanet, M. Angel Del Aguila Sanchez, S. L. Buchwald, *J. Am. Chem. Soc*. **2011**, 133, 9282–9285.
[7] PCT Int. Appl. (2011), WO 2011159297 A1 Dec 22, 2011
[8] S. Ullah, D. Kang, S. Lee, M. Ikram, C. Park, Y. Park, S. Yoon, P. Chun, H.R. Moon, *Eur. J. Med. Chem* **2019**, 161, 78-92.
[9] S. Müller, B. Liepold, G.J. Roth, H.J. Bestmann, *SYNLETT* **1996**, *1996*, 521.
[10] T.L. Wu, H.H. Chou, P.Y. Huang, C.H. Cheng, and R.S. Liu, *J. Org. Chem*. **2014**, 79, 267−274.
[11] J. E. Donello, PCT/US2012/025731.
[12] K. Okano, K. Okuyama, T. Fukuyama, H. Tokuyama, *SYNLETT* **2008**, 13, 1977–1980.

Author Contributions

Synthesis and characterization of previously listed compounds, determination of photophysical properties of helicenes: Thierry Hartung (PhD student), Rafael Machleid (Bachelor Student).
Method development and application of those methods for all HPLC- and SFC-separations: Martin Simon.
X-ray diffraction and structure elucidation of all relevant compounds and DFT-calculations: Christopher Golz.
Funding acquisition, project design and coordination, and writing of the manuscript: Manuel Alcarazo.
